# Efficient One-Pot Functionalization of Pyrroles via Dearomative Chlorination–Thiocyanation Strategy

**DOI:** 10.3390/ijms27125442

**Published:** 2026-06-16

**Authors:** Jingrui Zhang, Alexander S. Aldoshin, Victoria E. Shambalova, Valentine G. Nenajdenko

**Affiliations:** Department of Chemistry, M. V. Lomonosov Moscow State University, Leninskie Gory 1, Building 3, Moscow 119991, Russia; jingrui0821@mail.ru (J.Z.); aldon2258@mail.ru (A.S.A.); vshambalova00@gmail.com (V.E.S.)

**Keywords:** pyrrole, fluorine, dearomatization, umpolung, thiocyanation, trifluoromethylation

## Abstract

Reactivity of non-aromatic 2,5-dichloro-2*H*-pyrroles toward S-nucleophiles was investigated. It was found that these non-aromatic derivatives exhibit both oxidative and electrophilic properties. Their reaction with thiols and xanthates proceeds as redox process to form disulfides and 5-chlorinated pyrroles as a result of 2,5-dichloro-2*H*-pyrroles reduction. However, the reaction with ammonium thiocyanate afforded the corresponding 5-thiocyanated 1*H*-pyrroles. Based on these findings, a novel one-pot method for the thiocyanation of 2,3,4-trisubstituted pyrroles was developed. The protocol involves the in situ generation of highly reactive 2,5-dichloro-2*H*-pyrroles via dearomative chlorination of the corresponding pyrroles using trichloroisocyanuric acid (TCCA). Subsequent addition of ammonium thiocyanate leads to regioselective incorporation of a thiocyanate group at the C5 position and rearomatization of the pyrrole core. A broad scope of pyrrole-5-thiocyanates was obtained in yields up to 82%. Furthermore, these derivatives were efficiently transformed into 5-trifluoromethylthiolated pyrroles using Ruppert’s reagent in up to 94% yield. This reaction sequence provides a cost-effective way to obtain 5-trifluoromethylthiolated pyrroles, avoiding the need for high-cost electrophilic reagents. The synthetic utility of these novel sulfur-containing pyrrole derivatives was also demonstrated.

## 1. Introduction

Pyrroles represent one of the most fundamental classes of nitrogen-containing heterocycles, serving as essential building blocks in natural products [1,2,3,4], pharmaceuticals [5,6,7,8,9,10,11], and materials science [12,13,14,15]. Traditionally, the pyrrole ring is characterized as a “π-excessive” system, exhibiting high nucleophilicity particularly at the C-2 and C-5 positions. Consequently, the vast majority of pyrrole functionalization methods rely on electrophilic aromatic substitution [16]. However, the inherent electronic nature of pyrrole imposes significant limitations on the scope of accessible derivatives.

Inversion of the natural reactivity of the pyrrole ring from a nucleophile to an electrophile can overcome these synthetic limitations. While the nucleophilic nature of pyrroles is well-documented, the umpolung of pyrroles into electrophilic synthons remains an understudied area. Indeed, only few strategies have been developed to achieve this inversion. For example, the use of hypervalent iodine-based reagents allows the generation of electrophilic equivalents of pyrrole for subsequent reaction with nucleophiles [17,18,19,20].

On the other hand, organofluorine compounds are of great importance in organic [21], agricultural [22], medicinal chemistry [23,24,25] and in material science [26,27,28,29,30]. Currently, about a quarter of all commercial agrochemicals and pharmaceuticals incorporate at least one fluorine atom [31]. The introduction of fluorine into a molecular framework serves as a potent strategy for modulating the pharmacokinetic and physicochemical profiles of biologically active substances [32,33]. An important task in these fields is to create structures with fluorine atoms in precisely defined positions. Utilizing fluorine-containing building blocks often represents a more efficient and in many cases indispensable alternative to late-stage fluorination for accessing these structures [34,35].

**Scheme 1:**
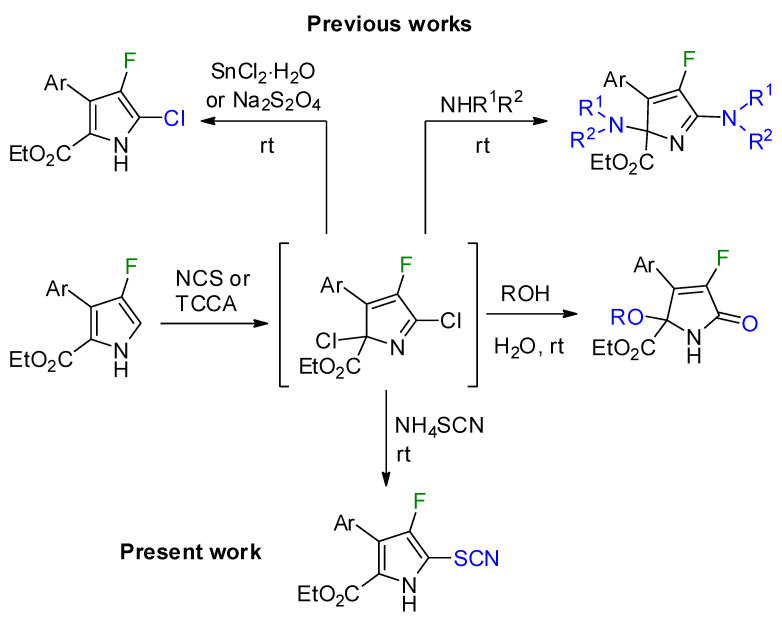
Nucleophilic modification of pyrroles via dearomative chlorination [38,39,40].

Having recently established a versatile synthesis of 4-fluoro-1*H*-pyrrole-2-carboxylates **1** via the Barton–Zard reaction [36], we subsequently expanded their synthetic utility through a novel oxidative umpolung strategy. By employing trichloroisocyanuric acid (TCCA) or N-chlorosuccinimide (NCS) [37], these electron-rich pyrroles can be converted into highly electrophilic 2,5-dichloro-2*H*-pyrroles (Scheme 1). This double chlorination effectively inverts the inherent polarity of the pyrrole core, enabling direct nucleophilic modification. Using this approach, we have accessed novel monofluorinated non-aromatic scaffolds, such as 2,5-diamino-2*H*-pyrroles [38] and 3-pyrrolin-2-ones [39], through reactions with amines and wet alcohols, respectively. Furthermore, they can undergo reduction to the corresponding 5-chloro-1H-pyrroles [40].

In the present work, we explore the reactivity of 2,5-dichloro-2*H*-pyrroles toward various S-nucleophiles. We revealed their unique dual reactivity as electrophiles and oxidants. The reaction with the thiocyanate anion opens an efficient access to 5-thiocyanated pyrroles as versatile precursors for 5-trifluoromethylthiolated derivatives and other functionalized derivatives.

## 2. Results and Discussion

**Scheme 2:**
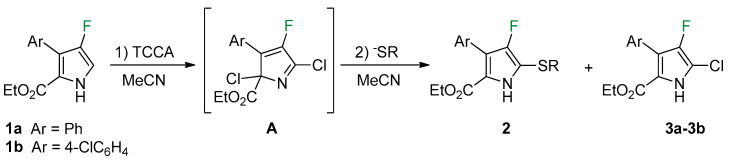
Reactions of 2,5-dichloropyrroles **A** with S-nucleophiles.

We started our study from a model reaction of electrophilic species **A** derived from pyrroles **1a**,**b** toward various S-nucleophiles. The key electrophilic intermediate, 2,5-dichloro-2*H*-pyrrole **A** was generated in situ upon treatment with TCCA, which simultaneously acts as a chlorinating agent and an oxidant to induce dearomatization of the starting material. Using this approach, the behavior of intermediate **A** was evaluated in subsequent reactions with various thiols, potassium ethyl xanthate and ammonium thiocyanate (Scheme 2).

It was found that all thiols act exclusively as reducing agents in this transformation, regardless of the nature of the substituent on the SH-group. Despite the use of various bases to generate more nucleophilic thiolate anions, all reactions consistently afforded the monochlorinated pyrroles **3** in 49–94% yields (Table 1, entries 1–10). The ethyl xanthate anion exhibited analogous reactivity (Table 1, entry 12). Consequently, in all these cases, the rearomatization of the non-aromatic 2*H*-pyrrole core proceeds via reduction, mirroring the behavior previously observed with inorganic reducing agents (Scheme 2).

**Scheme 3:**
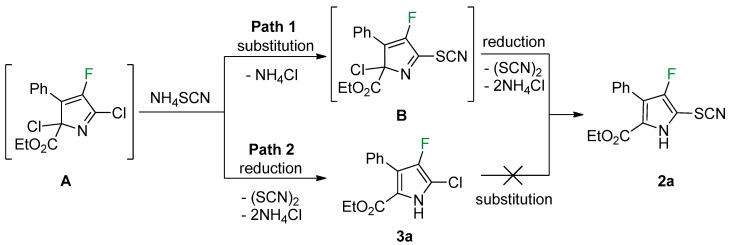
The mechanistic pathway for the reaction of intermediate **A** with ammonium thiocyanate.

In contrast, the reaction with ammonium thiocyanate yielded the nucleophilic substitution product **2a** and traces of reduced pyrrole **3a** (Table 1, entry 13). This observation suggests that the thiocyanate anion acts mainly as a nucleophile under these conditions. The transformation of intermediate **A** likely proceeds via two competing pathways (Scheme 3). While the reduction of **A** leads to the formation of **3a**, consistent with the behavior of other sulfur nucleophiles (Scheme 3, path 2), the process is dominated by the regioselective nucleophilic substitution of the C2–Cl bond by the thiocyanate anion (Scheme 3, path 1), affording intermediate **B**. Subsequent reduction of **B** by another equivalent of ammonium thiocyanate resulted in the desired pyrrole. Optimization revealed that utilizing 2.0–3.0 equivalents of NH_4_SCN ensures high selectivity toward the 2-thiocyanopyrrole **2a** (Table 1, entries 14 and 15). In terms of methodology, converting pyrroles into the 2,5-dichloro-2*H*-pyrrole intermediate changes their reactivity, allowing them to selectively react with the thiocyanate anion. The subsequent driving force of rearomatization then ensures the clean formation of the final products without the need for any additional reagents.

We reasoned that the selenocyanate anion could facilitate the similar introduction of a selenocyanato group. However, the reaction yielded only 5-chloropyrrole **2a** (Table 1, entry 16). The distinct reactivity of the thiocyanate anion compared to selenocyanate and thiols can be rationalized by their oxidation potentials. While the thiocyanate ion is weak reducing agent relatively resistant to oxidation (E_1/2_ ≈ 0.37 V vs. Fc/Fc+), the selenocyanate ion is a much more potent reductant (E_pa_ = −0.064 ÷ −0.18 V vs. Fc/Fc+) [41]. This latter value is comparable to the anodic peak potentials reported for various thiolate anions, which typically fall in the range of −1.1 to −0.5 V (vs. Fc/Fc+) in non-aqueous environments [42,43]. These electrochemical data support our observation that SeCN^−^ and thiols act exclusively as reducing agents toward intermediate **A**, leading to the rearomatized product **3**. We found also that the chlorine atom in pyrrole **3** is inert toward S_N_Ar substitution even at elevated temperatures.

**Scheme 4:**
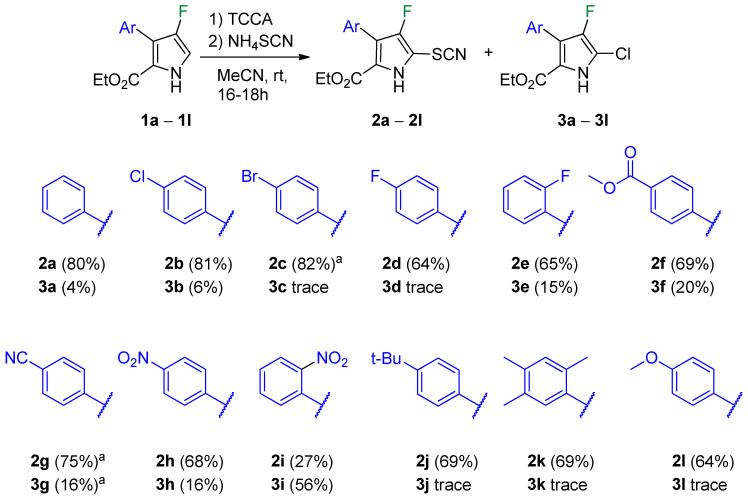
Scope of pyrroles **1** in one-pot thiocyanation; ^a^—thiocyanation step was conducted at 60 °C for 6 h.

Next, we studied the scope of the reaction. To our delight, the reaction with ammonium thiocyanate exhibited a broad substrate scope regarding pyrrole-2-carboxylates **1** (Scheme 4). Derivatives bearing both electron-donating (EDG) and electron-withdrawing groups (EWG) on the aryl ring were successfully converted into the corresponding pyrrole-2-thiocyanates **2**. It was noted, however, that substrates with EWGs showed slightly reduced selectivity. Nevertheless, the desired thiocyanated pyrroles **2** remained the major products in all cases, with the sole exception of the **2**-nitrophenyl-substituted derivative **1i**. The latter exhibited reversed selectivity favoring formation of the reduction side-product **3i**.

Interestingly, the pronounced shift toward the reduction pathway was uniquely observed for the *ortho*-nitro derivative **1i**, whereas the para-substituted **1h** analogs maintained high selectivity for thiocyanation. This difference can be explained by the *ortho*-effect: the proximity of the nitro group to the reactive center may cause steric distortion of the biaryl system, hindering the approach of the thiocyanate nucleophile [44,45]. Additionally, the strong field effect of the *ortho*-substituent likely lowers the energy of the transition state for the electron transfer, specifically promoting the reduction pathway [46,47]. In most instances, the yield of side-product **3** did not exceed 20%, while target pyrroles **2** were isolated in up to 82% yield. While the protocol typically proceeded smoothly at room temperature, the thiocyanation of pyrroles **1c** and **1g** required heating to 60 °C to achieve optimal efficiency (Scheme 4). The reaction proved scalable up to gram quantities without significant decrease in the yield. Specifically, the reaction of pyrrole **1a** (2.02 g, 8.56 mmol) afforded product **2a** in 74% yield (1.87 g).

**Scheme 5:**
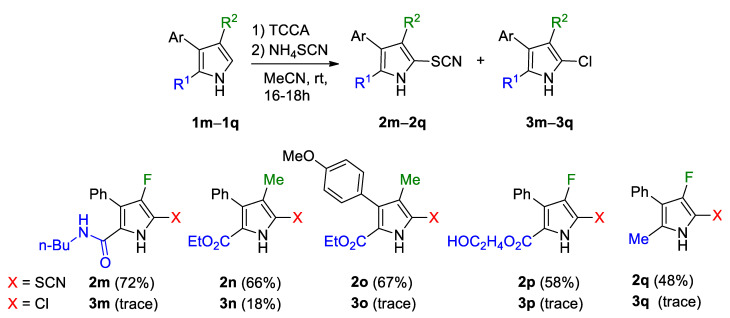
Scope of structurally diverse pyrroles **1m**–**1q** in one-pot thiocyanation.

The developed one-pot thiocyanation protocol proved applicable to a diverse range of trisubstituted pyrroles (Scheme 5). The corresponding thiocyanated products were obtained from pyrrole-2-butylamide **1m**, 4-methylated pyrrole-2-carboxylates **1n**–**1o**, 2-hydroxyethyl pyrrole-2-carboxylate **1p**, and 2-methylpyrrole **1q** in yields ranging from 48% to 72%. The lower yield of **2q** (48%) is attributed to the inherent instability of the C5-unsubstituted 2-methylpyrrole **1q**, which was generated via exhaustive reduction of the corresponding ester **1a**. However, the chlorination of pyrroles with multiple free positions occurs non-selectively and, after treatment with ammonium thiocyanate, leads to the formation of a complex, inseparable mixtures.

The achieved yields for pyrrole-5-thiocyanates highlight the efficiency of this one-pot sequence, demonstrating that despite the thermodynamic instability of the non-aromatic intermediate and a competitive reduction pathway, nucleophilic attack by the thiocyanate anion remains the dominant pathway.

**Scheme 6:**
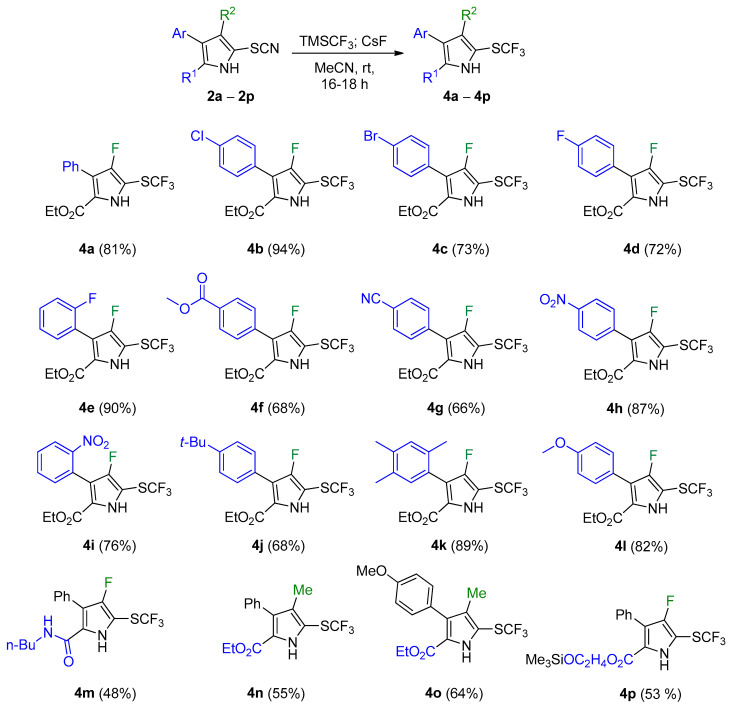
Scope of pyrrole-2-thiocyanates **2** in trifluromethylation with Ruppert’s reagent.

We proposed that the synthesized pyrrole-2-thiocyanates **2** are versatile intermediates for the synthesis of the trifluoromethylthio (SCF_3_)-substituted pyrroles. Treatment of **2** with trifluoromethyltrimethylsilane (Ruppert’s reagent) in the presence of cesium fluoride (CsF) facilitated the chemoselective nucleophilic substitution of the cyano group (Scheme 6). Remarkably, the reaction proceeded highly efficiently, leaving the pyrrole core and other functional groups intact.

**Figure 1:**
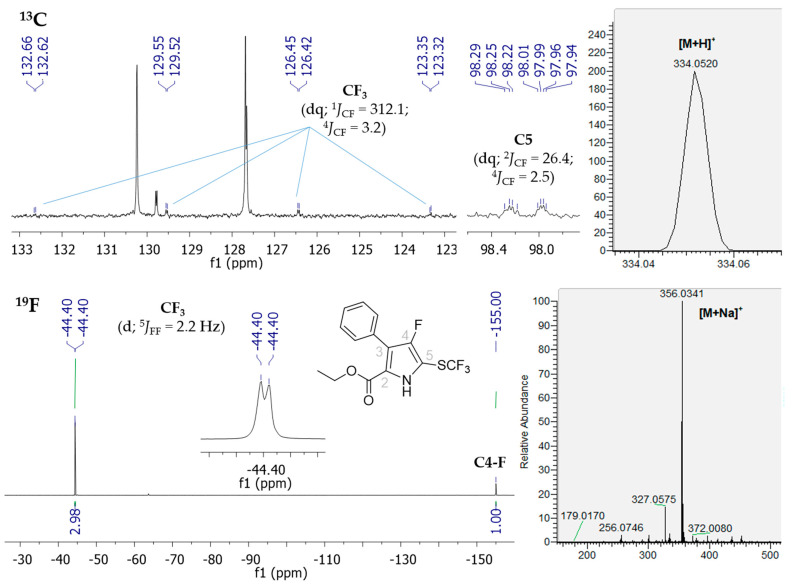
Key regions of the ^13^C and ^19^F NMR and mass spectra of compound **4a**, confirming CF_3_ functionalization.

The spectral data provide unambiguous evidence for the successful introduction of the CF_3_ group (Figure 1). For instance, the ^13^C NMR spectrum of product **4a** exhibits two distinct doublet of quartets (dq) signals at 128.0 and 98.1 ppm, resulting from heteronuclear coupling with both the three fluorine atoms of the CF_3_ group and the fluorine atom at the C4 position. Furthermore, the ^19^F NMR spectrum displays a new doublet at −44.4 ppm with an integration of 3F, which falls within the region characteristic of the SCF_3_ group. This doublet arises from the long-range coupling between the CF_3_ fluorine atoms and the fluorine atom at the C4 position. Additionally, the ESI-HRMS spectrum displays a base peak corresponding to the sodium adduct ([M + Na]^+^), alongside a low-intensity peak assigned to the protonated molecular ion ([M + H]^+^).

An exception was noted for **2p**, which underwent double functionalization: the thiocyanate group was converted to the SCF_3_ moiety, while the primary alcohol was simultaneously transformed into a trimethylsilyl (TMS) ether, affording **4p** in 53% yield as the only isolated product. The concomitant silylation of the hydroxyl group is attributed to the high electrophilicity of the silicon center in Ruppert’s reagent, combined with the strong thermodynamic driving force of Si–O bond formation [48,49].

The protocol demonstrated broad applicability, providing a diverse range of 5-trifluoromethylthiolated pyrroles **4** in yields up to 90% (Scheme 6). Moreover, it offers a cost-effective and efficient alternative to traditional methods that rely on expensive electrophilic trifluoromethylthiolating reagents [50,51,52].

**Scheme 7:**
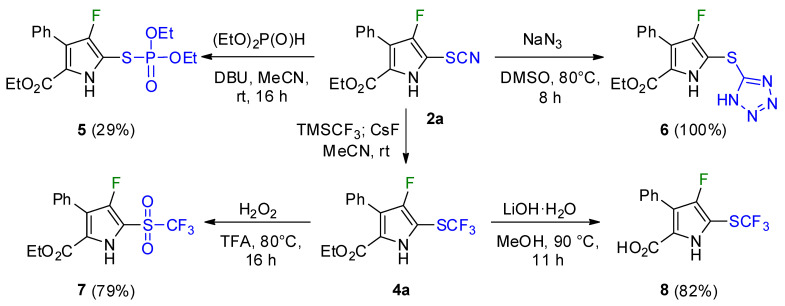
Synthetic utility of pyrrole derivatives **2** and **4**.

The synthetic utility of the obtained pyrrole derivatives **2** and **4** was further demonstrated through several downstream transformations (Scheme 7). For instance, the reaction of **2a** with diethyl phosphite in the presence of DBU facilitated the nucleophilic substitution of the cyano group, yielding the phosphonylated derivative **5** (29%). Furthermore, the [3+2]-cycloaddition of the azide anion afforded the corresponding sulfenyl tetrazole **6** in quantitative yield. Such sulfur-linked tetrazoles are of significant interest as potentially biologically active scaffolds and versatile pharmacophores in medicinal chemistry, exhibiting a wide range of biological activities [53,54,55,56].

Additionally, the oxidation of trifluoromethylthio-pyrrole **4a** with hydrogen peroxide in trifluoroacetic acid (TFA) smoothly provided sulfone **7** in 79% yield. Subsequent hydrolysis of the ester group of pyrrole **4a** using lithium hydroxide afforded the corresponding carboxylic acid **8** in 82% yield.

These pyrrole derivatives may be potentially useful in both medicinal chemistry and materials science. In drug discovery, the incorporation of the highly lipophilic SCF_3_ group or its strongly electron-withdrawing trifluoromethylsulfonyl (SO_2_CF_3_) analog alongside a fluorine atom can enhance metabolic stability and membrane permeability [57,58], while the tetrazolyl moiety serves as a classic bioisostere for carboxylic acids to tune binding profiles [59]. Concurrently, attaching these electron-withdrawing substituents to the electron-rich pyrrole core can modulate frontier molecular orbital (HOMO–LUMO) energy levels for organic electronics [60].

The structures of all newly synthesized compounds were unambiguously confirmed by ^1^H, ^13^C, and ^19^F NMR spectroscopy, as well as high-resolution mass spectrometry (HRMS).

In summary, we developed an efficient one-pot protocol for the C5-thiocyanation of pyrroles, mediated by dearomative oxidative chlorination with TCCA. This approach exploits the umpolung reactivity of in situ generated 2,5-dichloro-2*H*-pyrroles. While thiols and xanthates were found to act exclusively as reducing agents, the reaction with thiocyanate anion enabled regioselective nucleophilic substitution accompanied by rearomatization. A wide scope of 5-thiocyanato-1*H*-pyrroles **2** was obtained in yields up to 82%. These compounds were successfully transformed into 5-trifluoromethylthiolated pyrroles **4** using Ruppert’s reagent (up to 94% yield). This strategy offers a practical, cost-effective alternative to expensive electrophilic SCF_3_ reagents, providing a straightforward route to functionalized pyrroles while ensuring scalability for industrial applications. Crucially, the use of fluorinated pyrroles as starting building blocks allows for the selective installation of both the fluorine atom and the SCF_3_ group strictly at the C4 and C5 positions, respectively. However, the limitation of this methodology is its substrate dependency, as highly selective nucleophilic thiocyanation remains restricted primarily to 2,3,4-trisubstituted pyrroles. The synthetic utility of the obtained derivatives was further demonstrated through their transformation into phosphonyl, tetrazolyl, and sulfonyl pyrroles, as well as the corresponding carboxylic acid. These results highlight the potential of our method for the synthesis of complex, biologically relevant pyrrole-based scaffolds.

## 3. Materials and Methods

### 3.1. Materials

The starting pyrroles **1** [36,61] and potassium ethyl xanthate [62] were synthesized according to previously reported procedures. All other reagents were obtained from commercial sources (Sigma-Aldrich, St. Louis, MO, USA; Macklin Inc., Shanghai, China) and used without further purification. Solvents were dried and distilled using standard protocols. Melting points were determined on a Büchi B-545 apparatus (BUCHI Labortechnik AG, Flawil, Switzerland). ^1^H, ^13^C{^1^H}, ^19^F, and ^31^P{^1^H} NMR NMR spectra were recorded on a Bruker Avance-400 spectrometer (Bruker Corporation, Karlsruhe, Germany) at 400, 100, 376, and 162 MHz, respectively. Chemical shifts (δ) are reported in ppm relative to residual solvent signals as internal standards (^1^H, ^13^C). For ^19^F NMR, α*,*α,α-trifluorotoluene was used as an internal standard (δ = −63.72 ppm). Coupling constants (*J*) are given in Hertz (Hz). The following abbreviations are used to describe multiplicity: s (singlet), br s (broad singlet), d (doublet), t (triplet), q (quartet), m (multiplet), dd (doublet of doublets), dq (doublet of quartets), and tt (triplet of triplets). HRMS analysis was performed using an Orbitrap Q Exactive Plus high-resolution mass spectrometer (Thermo Scientific, Bremen, Germany) equipped with an orbital ion trap mass analyzer. Mass spectra were recorded in the *m*/*z* range of 100–2500 with a spectral resolution of 70,000 in positive and negative ion mode using electrospray ionization (ESI).

### 3.2. Synthesis and Characterization

**Preparation of potassium ethyl xanthate** (EtOCS_2_K). KOH (76.7 mg, 1.37 mmol, 1.0 mol equiv) was dissolved in absolute EtOH (10 mL). The solution was stirred at room temperature and then cooled to 0 °C. Carbon disulfide (CS_2_), (0.83 mL, 13.7 mmol, 10.0 mol equiv) was added dropwise, and the resulting mixture was stirred at room temperature for 1 h. The formed precipitate was collected by filtration, washed with diethyl ether (2 × 5 mL), and dried under vacuum to afford EtOCS_2_K (199.1 mg, 91%) as off-white solid. The analytical data were in agreement with those previously reported [62].
**2-Hydroxyethyl 4-fluoro-3-phenyl-1*H*-pyrrole-2-carboxylate** (**1p**). A vial was charged with pyrrole **1a** (0.506 g, 2.14 mmol, 1.0 mol equiv), potassium carbonate (0.148 g, 1.07 mmol, 0.5 mol equiv), and ethylene glycol (10 mL). The reaction mixture was stirred at 100 °C in an open vessel for 20 min until the starting material was consumed (monitored by TLC). Upon completion, the mixture was poured into ethyl acetate (50 mL) and washed with water (3 × 30 mL). The organic phase was dried over anhydrous, filtered, and concentrated under reduced pressure. The residue was purified by column chromatography on silica gel (eluent: CH_2_Cl_2_/EtOAc (1:5)) to afford **1p** (0.441 g, 82%) as a yellowish oil. ^1^H NMR (400 MHz, CDCl_3_) δ 9.98 (br s, 1H), 7.52 (d, *J* = 7.7 Hz, 2H), 7.46–7.31 (m, 3H), 6.67 (t, *J* = 3.4 Hz, 1H), 4.27–4.20 (m, 2H), 3.73–3.65 (m, 2H), 3.14 (br s, 1H). ^13^C NMR (100 MHz, CDCl_3_) δ 161.0 (d, ^4^*J*_CF_ = 2.9 Hz), 149.8 (d, ^1^*J*_CF_ = 243.5 Hz), 130.7 (d, ^4^*J*_CF_ = 2.2 Hz), 130.2, 127.8, 127.6, 118.6 (d, ^2^*J*_CF_ = 11.5 Hz), 114.7 (d, ^3^*J*_CF_ = 3.8 Hz), 107.6 (d, ^2^*J*_CF_ = 27.5 Hz), 65.7, 60.7. ^19^F NMR (376 MHz, CDCl_3_) δ −167.94. HRMS (ESI) *m*/*z*: [M − H]^−^ calcd. for C_13_H_11_FNO_3_^−^ 248.0728; found 248.0728.
**General procedure for the one-pot thiocyanation of pyrroles 1**. A solution of pyrroles **1** (0.2 mmol, 1.0 mol equiv) in acetonitrile (2 mL) was placed in a vial, and TCCA (31.1 mg, 0.134 mmol, 0.67 mol equiv) was added. The mixture was stirred at room temperature for 5 min until the oxidative chlorination was complete (monitored by TLC). Subsequently, NH_4_SCN (45.7 mg, 0.6 mmol, 3.0 mol equiv) was added, and the reaction was stirred overnight at room temperature (or at 60 °C for 6 h for **1c** and **1g**). Upon completion, the solvent was removed under reduced pressure. The residue was purified by column chromatography on silica gel using an appropriate mixture of solvents to afford the target product **2** and the side-product **3**.
**Ethyl 4-fluoro-3-phenyl-5-thiocyanato-1*H*-pyrrole-2-carboxylate** (**2a**). Eluents: Hex/DCM (5:1, 3:1, 1:1, 1:5, 1:10). Yield: 44.0 mg (80%) obtained from 43.7 mg (0.187 mmol) of pyrrole **1a**. Yield: 1.87 g (74%) obtained from 2.02 g (8.56 mmol) of pyrrole **1a**. Pale orange solid; mp 153–157 °C. ^1^H NMR (400 MHz, CDCl_3_) δ 10.14 (br s, 1H), 7.51–7.34 (m, 5H), 4.33 (q, *J* = 7.1 Hz, 2H), 1.23 (t, *J* = 7.1 Hz, 3H). ^13^C NMR (100 MHz, CDCl_3_) δ 160.5 (d, ^4^*J*_CF_ = 2.6 Hz), 152.7 (d, ^1^*J*_CF_ = 256.3 Hz), 130.4, 129.1 (d, ^3^*J*_CF_ = 2.6 Hz), 128.3, 128.0, 121.0 (d, ^3^*J*_CF_ = 2.9 Hz), 118.8 (d, ^2^*J*_CF_ = 11.2 Hz), 108.3, 95.0 (d, ^2^*J*_CF_ = 26.9 Hz), 62.0, 14.0. 19F NMR (376 MHz, CDCl3) δ −154.79 (s). HRMS (ESI) *m*/*z*: [M + H]^+^ calcd. for C_14_H_12_FN_2_O_2_S^+^ 291.0598; found 291.0552.
**Ethyl 5-chloro-4-fluoro-3-phenyl-1*H*-pyrrole-2-carboxylate** (**3a**). Eluents: Hex/DCM (5:1, 3:1, 1:1). Yield: 2.1 mg (4%). Colorless solid. The analytical data were in agreement with those previously reported [40].
**Ethyl 3-(4-chlorophenyl)-4-fluoro-5-thiocyanato-1*H*-pyrrole-2-carboxylate** (**2b**). Eluents: (Hex/DCM (1:1, 1:3), DCM) Yield: 492.5 mg (81%) obtained from 501.9 mg pyrrole **1b**. Pale yellow solid; mp 150–153 °C. ^1^H NMR (400 MHz, CDCl_3_) δ 10.08 (br s, 1H), 7.39 (s, 4H), 4.34 (q, *J* = 7.1 Hz, 2H), 1.25 (t, *J* = 7.1 Hz, 3H). ^13^C NMR (100 MHz, CDCl_3_) δ 160.2 (d, ^4^*J*_CF_ = 2.9 Hz), 152.6 (d, ^1^*J*_CF_ = 256.5 Hz), 134.4, 131.7, 128.3, 127.5 (d, ^3^*J*_CF_ = 2.7 Hz), 120.9 (d, ^3^*J*_CF_ = 2.7 Hz), 117.6 (d, ^2^*J*_CF_ = 11.1 Hz), 108.1 (d, ^4^*J*_CF_ = 2.2 Hz), 95.3 (d, ^2^*J*_CF_ = 27.0 Hz), 62.2, 14.1 ppm. 19F NMR (376 MHz, CDCl_3_) δ −153.96 (d, ^4^*J*_HF_ = 1.9 Hz). HRMS (ESI) *m*/*z*: [M + H]^+^ calcd. for C_14_H_11_ClFN_2_O_2_S 325.0214; found 325.0212.
**Ethyl 5-chloro-3-(4-chlorophenyl)-4-fluoro-1*H*-pyrrole-2-carboxylate** (**3b**). Eluents: Hex/DCM (1:1, 1:3). Yield: 32.4 mg (6%). Colorless solid. The analytical data were in agreement with those previously reported [40].
**Ethyl 3-(4-bromophenyl)-4-fluoro-5-thiocyanato-1*H*-pyrrole-2-carboxylate** (**2c**). Eluent: DCM. Yield: 21.7 mg (82%) obtained from 22.5 mg (0.072 mmol) of pyrrole **1c**. Pale yellow solid, mp 150–160 °C. ^1^H NMR (400 MHz, CDCl_3_) δ 10.07 (br s, 1H), 7.58–7.52 (m, 2H), 7.36–7.30 (m, 2H), 4.34 (q, *J* = 7.1 Hz, 2H), 1.26 (t, *J* = 7.1 Hz, 3H). ^13^C NMR (100 MHz, CDCl_3_) δ 160.2 (d, ^4^*J*_CF_ = 2.8 Hz), 152.6 (d, ^1^*J*_CF_ = 256.5 Hz), 132.0, 131.3, 128.0 (d, ^3^*J*_CF_ = 2.6 Hz), 122.6, 120.9 (d, ^3^*J*_CF_ = 2.5 Hz), 117.6 (d, ^2^*J*_CF_ = 11.2 Hz), 108.1 (d, ^4^*J*_CF_ = 2.3 Hz), 95.29 (d, ^2^*J*_CF_ = 27.1 Hz), 62.1, 14.2. 19F NMR (376 MHz, CDCl_3_) δ −154.29 (s). HRMS (ESI) *m*/*z*: [M + Na]^+^ calcd. for C_14_H_10_BrFN_2_NaO_2_S^+^ 390.9523; found 390.9576.
**Ethyl 4-fluoro-3-(4-fluorophenyl)-5-thiocyanato-1*H*-pyrrole-2-carboxylate** (**2d**). Eluent: Hex/DCM (1:5). Yield: 39.4 mg (64%). Pale yellow solid, mp 140–145 °C. ^1^H NMR (400 MHz, CDCl_3_) δ 10.01 (br s, 1H), 7.43 (dd, *J* = 8.2, 5.6 Hz, 2H), 7.15–7.06 (m, 2H), 4.33 (q, *J* = 7.1 Hz, 2H), 1.24 (t, *J* = 7.1 Hz, 3H). ^13^C NMR (100 MHz, CDCl_3_) δ 162.8 (d, ^1^*J*_CF_ = 247.9 Hz), 160.2 (d, ^4^*J*_CF_ = 2.9 Hz), 152.7 (d, ^1^*J*_CF_ = 256.3 Hz), 132.2 (d, ^3^*J*_CF_ = 8.3 Hz), 125.02 (pseudo-t, ^3^*J*_CF_ = ^4^*J*_CF_ = 3.0 Hz), 121.0 (d, ^3^*J*_CF_ = 2.6 Hz), 117.9 (d, ^2^*J*_CF_ = 11.2 Hz), 115.1 (d, ^2^*J*_CF_ = 21.8 Hz), 108.1, 95.2 (d, ^2^*J*_CF_ = 26.9 Hz), 62.0, 14.1. ^19^F NMR (376 MHz, CDCl_3_) δ −114.46–−114.54 (m, 1F), −154.17 (s, 1F). HRMS (ESI) *m*/*z*: [M + Na]^+^ calcd. for C_14_H_10_F_2_N_2_NaO_2_S^+^ 331.0323; found 331.0326.
**Ethyl 4-fluoro-3-(2-fluorophenyl)-5-thiocyanato-1*H*-pyrrole-2-carboxylate** (**2e**). Eluents: Hex/DCM (3:1, 1:1, 1:3, 1:5). Yield: 112 mg (65%) obtained from 141.3 mg (0.561 mmol) of pyrrole **1e**. Pale yellow solid, mp 133–138 °C. ^1^H NMR (400 MHz, CDCl_3_) δ 10.45 (br s, 1H), 7.43–7.35 (m, 2H), 7.24–7.17 (m, 1H), 7.17–7.10 (m, 1H), 4.32 (q, *J* = 7.1 Hz, 2H), 1.18 (t, *J* = 7.1 Hz, 3H). ^13^C NMR (100 MHz, CDCl_3_) 160.0 (d, ^4^*J*_CF_ = 2.8 Hz), 159.7 (d, ^1^*J*_CF_ = 248.5 Hz), 152.3 (d, ^1^*J*_CF_ = 257.2 Hz), 131.8, 129.9 (d, ^3^*J*_CF_ = 8.3 Hz), 123.2 (d, ^3^*J*_CF_ = 3.6 Hz), 121.6 (d, ^3^*J*_CF_ = 3.1 Hz), 116.9 (dd, ^2^*J*_CF_ = 15.7, ^3^*J*_CF_ = 2.6 Hz), 115.0 (d, ^2^*J*_CF_ = 22.0 Hz), 111.3 (d, ^2^*J*_CF_ = 12.6 Hz), 107.7 (d, ^4^*J*_CF_ = 2.2 Hz), 94.7 (d, ^2^*J*_CF_ = 26.7 Hz), 61.6, 13.3. ^19^F NMR (376 MHz, CDCl_3_) −114.14–−114.25 (m, 1F), −152.29 (d, ^5^*J*_FF_ = 6.2 Hz, 1F). HRMS (ESI-TOF) *m*/*z*: [M − C_2_H_6_O + H]^+^ calcd. for C_12_H_5_F_2_N_2_OS^+^ 263.0085; found 263.0093.
**Ethyl 5-chloro-4-fluoro-3-(2-fluorophenyl)-1*H*-pyrrole-2-carboxylate** (**3e**). Eluents: Hex/DCM (3:1, 1:1, 1:3). Yield: 24.5 mg (15%). Colorless solid. The analytical data were in agreement with those previously reported [40].
**Ethyl 4-fluoro-3-(4-(methoxycarbonyl)phenyl)-5-thiocyanato-1*H*-pyrrole-2-carboxylate** (**2f**). Eluents: Hex/DCM (3:1, 1:1, 1:3, 1:5). Yield: 48.8 mg (69%). Pale yellow solid, mp 145–150 °C. ^1^H NMR (400 MHz, CDCl_3_) δ 10.36 (br s, 1H), 8.09 (d, *J* = 8.4 Hz, 2H), 7.53 (d, *J* = 8.0 Hz, 2H), 4.33 (q, *J* = 7.1 Hz, 2H), 3.95 (s, 3H), 1.22 (t, *J* = 7.1 Hz, 3H). ^13^C NMR (100 MHz, CDCl_3_) δ 167.0, 160.2 (d, ^4^*J*_CF_ = 2.5 Hz), 152.6 (d, ^1^*J*_CF_ = 257.0 Hz), 133.9 (d, ^3^*J*_CF_ = 2.5 Hz), 130.4, 129.8, 129.3, 121.1 (d, ^3^*J*_CF_ = 2.3 Hz), 117.6 (d, ^2^*J*_CF_ = 10.9 Hz), 108.1, 95.5 (d, ^2^*J*_CF_ = 27.0 Hz), 62.2, 52.4, 14.1. ^19^F NMR (376 MHz, CDCl_3_) δ −153.61 (s). HRMS (ESI) *m*/*z*: [M + H]^+^ calcd. for C_16_H_14_FN_2_O_4_S^+^ 349.0653; found 349.0621.
**Ethyl 5-chloro-4-fluoro-3-(4-(methoxycarbonyl)phenyl)-1*H*-pyrrole-2-carboxylate** (**3f**). Eluents: Hex/DCM (3:1, 1:1, 1:3). Yield: 13 mg (20%). Colorless solid. The analytical data were in agreement with those previously reported [40].
**Ethyl 3-(4-cyanophenyl)-4-fluoro-5-thiocyanato-1*H*-pyrrole-2-carboxylate** (**2g**). Eluents: Hex/DCM (1:1, 1:5), DCM. Yield: 47.4 mg (75%). Off-white solid, mp 193–208 °C. ^1^H NMR (400 MHz, CDCl_3_) δ 12.38 (br s, 1H), 7.67–7.61 (m, 2H), 7.51 (d, *J* = 7.9 Hz, 2H), 4.20 (q, *J* = 7.1 Hz, 2H), 1.16 (t, *J* = 7.1 Hz, 3H). ^13^C NMR (100 MHz, CDCl_3_) δ 159.1 (d, ^4^*J*_CF_ = 2.8 Hz), 151.2 (d, ^1^*J*_CF_ = 255.2 Hz), 134.4 (d, ^3^*J*_CF_ = 2.9 Hz), 130.9, 130.5, 120.5 (d, ^3^*J*_CF_ = 3.3 Hz), 118.3, 115.0 (d, ^2^*J*_CF_ = 10.7 Hz), 110.6, 108.3 (d, ^4^*J*_CF_ = 2.0 Hz), 95.0 (d, ^2^*J*_CF_ = 25.7 Hz), 60.6, 13.5. ^19^F NMR (376 MHz, CDCl_3_) δ −155.24 (s). HRMS (ESI) *m*/*z*: [M + Na]^+^ calcd. for C_15_H_10_FN_3_NaO_2_S^+^ 338.0370; found 338.0370.
**Ethyl 5-chloro-3-(4-cyanophenyl)-4-fluoro-1*H*-pyrrole-2-carboxylate** (**3g**). Eluents: Hex/DCM (1:1, 1:5). Yield: 9.2 mg (16%). Colorless solid. The analytical data were in agreement with those previously reported [40].
**Ethyl 4-fluoro-3-(4-nitrophenyl)-5-thiocyanato-1*H*-pyrrole-2-carboxylate** (**2h**). Eluents: Hex/DCM (1:1, 1:3, 1:5). Yield: 250.8 mg (68%) obtained from 305.1 mg (1.08 mmol) of pyrrole **1h**. Pale yellow solid, mp 163–168 °C. ^1^H NMR (400 MHz, CDCl_3_) δ 10.03 (br s, 1H), 8.33–8.23 (m, 2H), 7.66 (d, *J* = 8.4 Hz, 2H), 4.35 (q, *J* = 7.1 Hz, 2H), 1.26 (t, *J* = 7.1 Hz, 3H). ^13^C NMR (100 MHz, CDCl_3_) δ 159.7 (d, ^4^*J*_CF_ = 2.8 Hz), 152.5 (d, ^1^*J*_CF_ = 257.7 Hz), 147.6, 135.9 (d, ^3^*J*_CF_ = 2.7 Hz), 131.3, 123.3, 121.2 (d, ^3^*J*_CF_ = 2.4 Hz), 116.43 (d, ^2^*J*_CF_ = 10.6 Hz), 107.83 (d, ^4^*J*_CF_ = 2.2 Hz), 95.8, 62.4, 14.2. ^19^F NMR (376 MHz, CDCl_3_) δ −153.97 (s). HRMS (ESI) *m*/*z*: [M + H]^+^ calcd. for C_14_H_11_FN_3_O_4_S^+^ 336.0449; found 336.0449.
**Ethyl 5-chloro-4-fluoro-3-(4-nitrophenyl)-1*H*-pyrrole-2-carboxylate** (**3h**). Eluents: Hex/DCM (1:1, 1:3). Yield: 56.3 mg (16%). Yellowish solid. The analytical data were in agreement with those previously reported [40].
**Ethyl 4-fluoro-3-(2-nitrophenyl)-5-thiocyanato-1*H*-pyrrole-2-carboxylate** (**2i**). Eluents: Hex/DCM (1:1), DCM, DCM/EtOAc (10:1, 5:1). Yield: 15.2 mg (27%). Pale orange solid, mp 163–170 °C. ^1^H NMR (400 MHz, CDCl_3_) δ 10.17 (br s, 1H), 8.12 (dd, *J* = 8.2, 1.2 Hz, 1H), 7.68 (td, *J* = 7.6, 1.3 Hz, 1H), 7.62–7.54 (m, 1H), 7.46 (d, *J* = 7.6 Hz, 1H), 4.20 (q, *J* = 7.1 Hz, 2H), 1.10 (t, *J* = 7.1 Hz, 3H). ^13^C NMR (100 MHz, CDCl_3_) δ 159.7 (d, ^4^*J*_CF_ = 2.9 Hz), 152.31 (d, ^1^*J*_CF_ = 256.7 Hz), 149.2, 133.4, 133.0, 129.6, 124.8 (^3^*J*_CF_ = 2.3 Hz), 124.8, 121.2 (d, ^3^*J*_CF_ = 2.5 Hz), 114.1 (d, ^2^*J*_CF_ = 12.1 Hz), 108.09 (d, ^4^*J*_CF_ = 2.9 Hz), 95.6 (d, ^2^*J*_CF_ = 26.5 Hz), 62.3, 13.7. ^19^F NMR (376 MHz, CDCl_3_) δ −153.26 (s). HRMS (ESI) *m*/*z*: [M + Na]^+^ calcd. for C_14_H_10_FN_3_O_4_SNa^+^ 358.0268; found 358.0258.
**Ethyl 5-chloro-4-fluoro-3-(2-nitrophenyl)-1*H*-pyrrole-2-carboxylate** (**3i**). Eluents: Hex/DCM (1:1), DCM, DCM/EtOAc (10:1, 5:1). Yield: 35.1 mg (56%). Yellowish solid. The analytical data were in agreement with those previously reported [40].
**Ethyl 3-(4-(tert-butyl)phenyl)-4-fluoro-5-thiocyanato-1*H*-pyrrole-2-carboxylate** (**2j**). Eluent: Hex/DCM (1:3). Yield: 47.1 mg (69%). Pale yellow solid, mp 143–153 °C. ^1^H NMR (400 MHz, CDCl_3_) δ 10.20 (br s, 1H), 7.47–7.38 (m, 4H), 4.34 (q, *J* = 7.1 Hz, 2H), 1.36 (s, 9H), 1.24 (t, *J* = 7.1 Hz, 3H). ^13^C NMR (100 MHz, CDCl_3_) δ 160.5 (d, ^4^*J*_CF_ = 2.7 Hz), 152.8 (d, ^1^*J*_CF_ = 255.9 Hz), 151.3, 130.0, 126.0 (d, ^3^*J*_CF_ = 2.7 Hz), 125.0, 120.9 (d, ^3^*J*_CF_ = 3.0 Hz), 118.9 (d, ^2^*J*_CF_ = 11.3 Hz), 108.3 (d, ^4^*J*_CF_ = 2.0 Hz), 94.9 (d, ^2^*J*_CF_ = 27.1 Hz), 61.9, 34.8, 31.4, 14.0. ^19^F NMR (376 MHz, CDCl_3_) δ −154.91–−154.98 (m). HRMS (ESI) *m*/*z*: [M + Na]^+^ calcd. for C_18_H_19_FN_2_O_2_SNa^+^ 369.1043; found 369.1077.
**Ethyl 4-fluoro-5-thiocyanato-3-(2,4,5-trimethylphenyl)-1*H*-pyrrole-2-carboxylate** (**2k**). Eluent: Hex/DCM (1:3). Yield: 46 mg (69%). Pink solid, mp 143–148 °C. ^1^H NMR (400 MHz, CDCl_3_) δ 10.30 (br s, 1H), 7.05 (s, 1H), 6.98 (s, 1H), 4.36–4.19 (m, 2H), 2.28 (s, 3H), 2.24 (s, 3H), 2.14 (s, 3H), 1.14 (t, *J* = 7.1 Hz, 3H). ^13^C NMR (100 MHz, CDCl_3_) δ 160.6 (d, ^4^*J*_CF_ = 2.9 Hz), 152.8 (d, ^1^*J*_CF_ = 255.1 Hz), 136.9, 134.5, 133.3, 132.1, 131.2, 126.2 (d, ^3^*J*_CF_ = 2.2 Hz), 121.7 (d, ^3^*J*_CF_ = 3.7 Hz), 118.3 (d, ^2^*J*_CF_ = 13.3 Hz), 108.4 (d, ^4^*J*_CF_ = 2.3 Hz), 94.7 (d, ^2^*J*_CF_ = 27.2 Hz), 61.7, 19.6, 19.5, 19.2, 13.9. ^19^F NMR (376 MHz, CDCl_3_) δ −151.82 (s). HRMS (ESI) *m*/*z*: [M + Na]^+^ calcd. for C_17_H_17_FN_2_O_2_SNa^+^ 355.0887; found 355.0914.
**Ethyl 4-fluoro-3-(4-methoxyphenyl)-5-thiocyanato-1*H*-pyrrole-2-carboxylate** (**2l**). Eluent: hexane/CH_2_Cl_2_ (1:3). Yield: 40.9 mg (64%). Pale yellow solid, mp 153–160 °C ^1^H NMR (400 MHz, CDCl_3_) δ 10.04 (br s, 1H), 7.39 (dd, *J* = 8.8, 0.9 Hz, 2H), 6.98–6.92 (m, 2H), 4.33 (q, *J* = 7.1 Hz, 2H), 3.85 (d, *J* = 4.3 Hz, 3H), 1.26 (t, *J* = 7.1 Hz, 3H). ^13^C NMR (100 MHz, CDCl_3_) δ 160.4 (d, ^4^*J*_CF_ = 2.8 Hz), 159.6, 152.8 (d, ^1^*J*_CF_ = 255.5 Hz), 131.6, 121.2 (d, ^3^*J*_CF_ = 2.5 Hz), 120.8 (d, ^3^*J*_CF_ = 2.9 Hz), 118.7 (d, ^2^*J*_CF_ = 11.2 Hz), 113.5, 108.3 (d, ^4^*J*_CF_ = 2.0 Hz), 94.8 (d, ^2^*J*_CF_ = 27.3 Hz), 61.9, 55.4, 14.2. ^19^F NMR (376 MHz, CDCl_3_) δ −155.28 (s). HRMS (ESI) *m*/*z*: [M + Na]^+^ calcd. for C_15_H_13_FN_2_O_3_SNa^+^ 343.0523; found 343.0530.
**N-butyl-4-fluoro-3-phenyl-5-thiocyanato-1*H*-pyrrole-2-carboxamide** (**2m**). Eluent: CH_2_Cl_2_/EtOAc (20:1). Yield: 34.2 mg (72%) obtained from 39.0 mg (0.15 mmol) pyrrole **1m**. Yellowish solid, mp 142–145 °C. ^1^H NMR (400 MHz, CDCl_3_) δ 12.00 (br s, 1H), 7.57–7.41 (m, 5H), 5.82 (t, *J* = 5.3 Hz, 1H), 3.37 (dd, *J* = 12.6, 6.8 Hz, 2H), 1.40–1.25 (m, 2H), 11.21–1.08 (m, 2H), 0.84 (t, *J* = 7.3 Hz, 3H). ^13^C NMR (100 MHz, CDCl_3_) δ 160.0 (d, ^4^*J*_CF_ = 2.4 Hz), 152.5 (d, ^1^*J*_CF_ = 255.4 Hz), 130.5, 129.6, 129.5 (d, ^3^*J*_CF_ = 2.2 Hz), 129.2, 124.2 (d, ^3^*J*_CF_ = 1.4 Hz), 113.8 (d, ^2^*J*_CF_ = 12.5 Hz), 108.8 (d, ^4^*J*_CF_ = 2.3 Hz), 93.3 (d, ^2^*J*_CF_ = 26.2 Hz), 39.5, 31.1, 20.0, 13.7. ^19^F NMR (376 MHz, CDCl_3_) −155.38 (d, ^4^*J*_HF_ = 2.2 Hz). HRMS (ESI) *m*/*z*: [M + H]^+^ calcd. for C_16_H_17_FN_3_OS^+^ 318.1071; found 318.1119.
**Ethyl 4-methyl-3-phenyl-5-thiocyanato-1*H*-pyrrole-2-carboxylate** (**2n**). Eluents: hexane/CH_2_Cl_2_ (5:1, 3:1, 1:1, 1:3). Yield: 37.8 mg (66%). Pale yellow solid, mp 173–178 °C. ^1^H NMR (400 MHz, CDCl_3_) δ 10.15 (s, 1H), 7.42–7.32 (m, 3H), 7.30–7.26 (m, 2H), 4.24 (q, *J* = 7.1 Hz, 2H), 2.11 (s, 3H), 1.13 (t, *J* = 7.1 Hz, 3H). ^13^C NMR (101 MHz, CDCl_3_) δ 160.7, 133.6, 132.0, 130.2, 129.3, 127.9, 127.5, 123.7, 109.0, 107.3, 61.2, 14.0, 10.4. HRMS (ESI) *m*/*z*: [M + H]^+^ calcd. for C_15_H_15_N_2_O_2_S^+^ 287.0849; found 287.0857.
**Ethyl 5-chloro-4-methyl-3-phenyl-1*H*-pyrrole-2-carboxylate** (**3n**). Eluents: hexane/CH_2_Cl_2_ (5:1, 3:1, 1:1). Yield: 9.7 mg (18%). Colorless solid. The analytical data were in agreement with those previously reported [40].
**Ethyl 3-(4-methoxyphenyl)-4-methyl-5-thiocyanato-1*H*-pyrrole-2-carboxylate** (**2o**). Eluents: hexane/CH_2_Cl_2_ (3:1, 1:1, 1:3). Yield: 42 mg (67%). Pale yellow solid, mp 150–155 °C. ^1^H NMR (400 MHz, CDCl_3_) δ 10.07 (br s, 1H), 7.24–7.19 (m, 2H), 6.96–6.90 (m, 2H), 4.25 (q, *J* = 7.1 Hz, 2H), 3.85 (s, 3H), 2.11 (s, 3H), 1.18 (t, *J* = 7.1 Hz, 3H). ^13^C NMR (100 MHz, CDCl_3_) δ 160.7, 159.0, 131.7, 131.4, 129.4, 125.8, 123.6, 113.3, 109.1, 107.1, 61.1, 55.4, 14.2, 10.5. HRMS (ESI) *m*/*z*: [M + Na]^+^ calcd. for C_16_H_16_N_2_NaO_3_S^+^ 339.0774; found 339.0804.
**3-Hydroxypropyl 4-fluoro-3-phenyl-5-thiocyanato-1*H*-pyrrole-2-carboxylate** (**2p**). Eluents: CH_2_Cl_2_/EtOAc (10:1, 5:1, 3:1). Yield: 35 mg (58%). Pale yellow solid, mp 138–141 °C. ^1^H NMR (400 MHz, CDCl_3_) δ 10.81 (br s, 1H), 7.50–7.35 (m, 5H), 4.37–4.31 (m, 2H), 3.88–3.73 (m, 2H), 2.60 (br s, 1H). ^13^C NMR (100 MHz, CDCl_3_) δ 159.8 (d, ^4^*J*_CF_ = 2.9 Hz), 152.6 (d, ^1^*J*_CF_ = 256.1 Hz), 130.2, 129.2 (d, ^3^*J*_CF_ = 2.6 Hz), 128.5, 128.2, 120.5 (d, ^3^*J*_CF_ = 3.2 Hz), 119.2 (d, ^2^*J*_CF_ = 11.5 Hz), 108.6 (d, ^4^*J*_CF_ = 2.2 Hz), 95.2 (d, ^2^*J*_CF_ = 26.8 Hz), 66.6, 60.8. ^19^F NMR (376 MHz, CDCl_3_) δ −155.68 (s). HRMS (ESI) *m*/*z*: [M + Na]^+^ calcd. for C_14_H_11_FN_2_NaO_3_S^+^ 329.0367; found 329.0368.
**Methyl 3-ethyl-2,4-dimethyl-5-thiocyanato-1*H*-pyrrole-2-carboxylate** (**2q**). Eluent: hexane/CH_2_Cl_2_ (1:3). Yield: 55.3 mg (48%) obtained from 86.2 mg (0.492 mmol) of pyrrole **1q**. Dark yellow solid, mp 97–101 °C. ^1^H NMR (400 MHz, CDCl_3_) δ 8.24 (br s, 1H), 7.46–7.39 (m, 2H), 7.36 (d, *J* = 8.0 Hz, 2H), 7.3–7.28 (m, 1H), 2.34 (s, 3H). ^13^C NMR (100 MHz, CDCl_3_) δ 153.6 (d, ^1^*J*_CF_ = 255.0 Hz), 130.7 (d, ^3^*J*_CF_ = 3.0 Hz), 129.5 (d, ^3^*J*_CF_ = 3.5 Hz), 128.2 (d, ^4^*J*_CF_ = 2.0 Hz), 128.2, 126.4, 111.5 (d, ^2^*J*_CF_ = 10.9 Hz), 109.8 (d, ^4^*J*_CF_ = 2.9 Hz), 84.5 (d, ^2^*J*_CF_ = 27.4 Hz), 12.9. ^19^F NMR (376 MHz, CDCl_3_) δ −155.34 (s). HRMS (ESI) *m*/*z*: [M + H]^+^ calcd. for C_12_H_10_FN_2_S^+^ 233.0543; found 233.05444
**General procedure for the synthesis of 5-trifluoromethylthiolated pyrroles 4 from thiocyanates 2**. To a solution of pyrrole-2-thiocyanate **2** (0.2 mmol, 1.0 mol equiv) in acetonitrile (2 mL), TMSCF_3_ (85.3 mg, 0.6 mmol, 3.0 mol equiv) and CsF (91.1 mg, 0.6 mmol, 3.0 mol equiv) were added. The reaction mixture was stirred at room temperature overnight until the starting material was consumed (monitored by TLC). The solvent was removed under reduced pressure, and the residue was purified by column chromatography on silica gel using an appropriate mixture of solvents to afford the corresponding 5-trifluoromethylthiolated product **4**.
**Ethyl 4-fluoro-3-phenyl-5-((trifluoromethyl)thio)-1*H*-pyrrole-2-carboxylate** (**4a**). Eluent: hexane/CH_2_Cl_2_ (1:3). Yield: 933 mg (81%) obtained from 1.004 g (3.445 mmol) of pyrrole **2a**. Off-white solid, mp 163–167 °C. ^1^H NMR (400 MHz, CDCl_3_) δ 9.51 (br s, 1H), 7.48 (d, *J* = 7.9 Hz, 2H), 7.44–7.33 (m, 3H), 4.29 (q, *J* = 7.1 Hz, 2H), 1.23 (t, *J* = 7.1 Hz, 3H). ^13^C NMR (100 MHz, CDCl_3_) δ 160.3 (d, ^4^*J*_CF_ = 3.1 Hz), 153.5 (d, ^1^*J*_CF_ = 254.2 Hz), 130.2, 129.8 (d, ^3^*J*_CF_ = 2.8 Hz), 128.0 (dq, ^1^*J*_CF_ = 312.1, ^4^*J*_CF_ = 3.2 Hz), 127.7, 127.6, 120.5 (d, ^3^*J*_CF_ = 3.3 Hz), 117.6 (d, ^2^*J*_CF_ = 11.3 Hz), 98.1 (dq, ^2^*J*_CF_ = 26.4, ^3^*J*_CF_ = 2.5 Hz), 61.1, 13.9. ^19^F NMR (376 MHz, CDCl_3_) δ −44.40 (d, ^5^*J*_FF_ = 2.3 Hz, 3F), −155.00 (s, 1F). HRMS (ESI) *m*/*z*: [M + Na]^+^ calcd. for C_14_H_11_F_4_NNaO_2_S^+^ 356.0339; found 356.0341.
**Ethyl 3-(4-chlorophenyl)-4-fluoro-5-((trifluoromethyl)thio)-1*H*-pyrrole-2-carboxylate** (**4b**). Eluents: hexane/CH_2_Cl_2_ (1:1, 1:3, 1:5). Yield: 107.5 mg (94%) obtained from 101.1 mg (0.308 mmol) of pyrrole **2b**. Off-white solid, mp 170–172 °C. ^1^H NMR (400 MHz, CDCl_3_) δ 9.61 (br s, 1H), 7.42 (d, *J* = 8.5 Hz, 2H), 7.40–7.36 (m, 2H), 4.31 (q, *J* = 7.1 Hz, 2H), 1.25 (t, *J* = 7.1 Hz, 3H). ^13^C NMR (100 MHz, CDCl_3_) δ 160.4 (d, ^4^*J*_CF_ = 2.8 Hz), 153.7 (d, ^1^*J*_CF_ = 255.4 Hz), 134.1, 131.7, 128.2, 128.0 (dq, ^1^*J*_CF_ = 312.3, ^4^*J*_CF_ = 3.3 Hz), 128.0 (d, ^3^*J*_CF_ = 2.8 Hz), 120.5 (d, ^3^*J*_CF_ = 2.8 Hz), 117.1 (d, ^2^*J*_CF_ = 11.3 Hz), 98.8 (dq, ^2^*J*_CF_ = 26.7, ^3^*J*_CF_ = 2.6 Hz), 61.9, 14.1. ^19^F NMR (376 MHz, CDCl_3_) δ −44.4 (d, ^5^*J*_FF_ = 2.2 Hz, 3F), −155.1 (s, 1F). HRMS (ESI) *m*/*z*: [M + Na]^+^ calcd. for C_14_H_10_ClF_4_NNaO_2_S^+^ 389.9949; found 389.9952.
**Ethyl 3-(4-bromophenyl)-4-fluoro-5-((trifluoromethyl)thio)-1*H*-pyrrole-2-carboxylate** (**4c**). Eluents: hexane/CH_2_Cl_2_ (10:1, 5:1, 3:1). Yield: 24.1 mg (73%) obtained from 29.4 mg (0.080 mmol) of pyrrole **2c**. Colorless solid, mp 160–165 °C. ^1^H NMR (400 MHz, CDCl_3_) δ 9.55 (br s, 1H), 7.57–7.51 (m, 2H), 7.36 (d, *J* = 7.8 Hz, 2H), 4.30 (q, *J* = 7.1 Hz, 2H), 1.25 (t, *J* = 7.1 Hz, 3H). ^13^C NMR (100 MHz, CDCl_3_) δ 160.1 (d, ^4^*J*_CF_ = 2.5 Hz), 153.7 (d, ^1^*J*_CF_ = 255.9 Hz), 132.0, 131.2, 128.4 (d, ^3^*J*_CF_ = 2.9 Hz), 128.0 (dq, ^1^*J*_CF_ = 312.0, ^4^*J*_CF_ = 3.0 Hz), 122.4, 120.5 (d, ^3^*J*_CF_ = 2.9 Hz), 117.13 (d, ^2^*J*_CF_ = 11.3 Hz), 98.7 (dq, ^2^*J*_CF_ = 26.3, ^3^*J*_CF_ = 3.0 Hz), 61.8, 14.2. ^19^F NMR (376 MHz, CDCl_3_) δ −44.42 (d, ^5^*J*_FF_ = 2.3 Hz, 3F), −155.2 (s, 1F). HRMS (ESI) *m*/*z*: [M + H]^+^ calcd. for C_14_H_11_BrFN_2_O_2_S^+^ 411.9625; found 411.9625.
**Ethyl 4-fluoro-3-(4-fluorophenyl)-5-((trifluoromethyl)thio)-1*H*-pyrrole-2-carboxylate** (**4d**). Eluents: hexane/CH_2_Cl_2_ (10:1, 5:1, 3:1). Yield: 31.6 mg (72%) obtained from 38.4 mg (0.125 mmol) of pyrrole **2d**. Colorless solid, mp 167–172 °C. ^1^H NMR (400 MHz, CDCl_3_) δ 9.41 (s, 1H), 7.45 (dd, *J* = 8.0, 5.5 Hz, 2H), 7.14–7.06 (m, 2H), 4.29 (q, *J* = 7.1 Hz, 2H), 1.24 (t, *J* = 7.1 Hz, 3H). ^13^C NMR (100 MHz, CDCl_3_) δ 162.7 (d, ^1^*J*_CF_ = 247.5 Hz), 160.1 (d, ^4^*J*_CF_ = 3.1 Hz), 153.8 (d, ^1^*J*_CF_ = 255.2 Hz), 132.2 (d, ^3^*J*_CF_ = 8.0 Hz), 128.0 (dq, ^1^*J*_CF_ = 312.5, ^4^*J*_CF_ = 3.6 Hz), 125.4 (pseudo-t, ^3^*J*_CF_ = ^4^*J*_CF_ = 2.9 Hz), 120.5 (d, ^3^*J*_CF_ = 3.2 Hz), 117.4 (d, ^2^*J*_CF_ = 11.3 Hz), 115.0 (d, ^2^*J*_CF_ = 21.6 Hz), 98.5 (dq, ^2^*J*_CF_ = 26.8, ^3^*J*_CF_ = 2.7 Hz), 61.7, 14.2. ^19^F NMR (376 MHz, CDCl_3_) δ −44.41 (d, ^5^*J*_FF_ = 2.2 Hz, 3F), −114.90 (tt, *J* = 8.7, 5.4 Hz, 1F), −155.34 (s, 1F). HRMS (ESI) *m*/*z*: [M + H]^+^ calcd. for C_14_H_11_F_5_NO_2_S^+^ 352.0425; found 352.0425.
**Ethyl 4-fluoro-3-(2-fluorophenyl)-5-((trifluoromethyl)thio)-1*H*-pyrrole-2-carboxylate** (**4e**). Eluents: hexane/CH_2_Cl_2_ (5:1, 3:1). Yield: 45.8 mg (90%) Prepared from 44.9 mg (0.146 mmol) of pyrrole **2e**. Off-white solid, mp 140–145 °C. ^1^H NMR (400 MHz, CDCl_3_) δ 9.92 (br s, 1H), 7.44–7.33 (m, 2H), 7.19 (td, *J* = 7.5, 1.1 Hz, 1H), 7.17–7.10 (m, 1H), 4.27 (q, *J* = 7.1 Hz, 2H), 1.17 (t, *J* = 7.1 Hz, 3H). ^13^C NMR (100 MHz, CDCl_3_) δ 160.4 (d, ^4^*J*_CF_ = 2.3 Hz), 160.3 (d, ^1^*J*_CF_ = 248.5 Hz), 154.0 (d, ^1^*J*_CF_ = 256.6 Hz), 132.4 (d, ^4^*J*_CF_ = 2.3 Hz), 130.2 (d, ^3^*J*_CF_ = 8.2 Hz), 128.0 (dq, ^1^*J*_CF_ = 312.2, ^4^*J*_CF_ = 3.3 Hz), 123.7 (d, ^3^*J*_CF_ = 3.5 Hz), 121.7 (d, ^3^*J*_CF_ = 3.2 Hz), 117.8 (dd, ^2^*J*_CF_ = 15.7, ^3^*J*_CF_ = 2.6 Hz), 115.5 (d, ^1^*J*_CF_ = 22.1 Hz), 111.3 (d, ^1^*J*_CF_ = 12.7 Hz), 98.6 (dq, ^2^*J*_CF_ = 26.5, ^3^*J*_CF_ = 2.7 Hz), 61.7, 13.9. ^19^F NMR (376 MHz, CDCl_3_) δ −44.54 (d, ^5^*J*_FF_ = 2.3 Hz, 3F), −114.28–−114.39 (m, 1F), −153.61 (dd, *J* = 4.3, 1.8 Hz, 1F). HRMS (ESI) *m*/*z*: [M + H]^+^ calcd. for C_14_H_11_F_5_NO_2_S^+^ 352.0425; found 352.0425.
**Ethyl 4-fluoro-3-(4-(methoxycarbonyl)phenyl)-5-((trifluoromethyl)thio)-1*H*-pyrrole-2-carboxylate** (**4f**). Eluents: hexane/CH_2_Cl_2_ (1:1, 1:3). Yield: 37.4 mg (68%) obtained from 48.8 mg (0.140 mmol) of pyrrole **2f**. Pale yellow solid, mp 155–157 °C. ^1^H NMR (400 MHz, CDCl_3_) δ 9.68 (br s, 1H), 8.08 (d, *J* = 8.4 Hz, 2H), 7.56 (d, *J* = 7.9 Hz, 2H), 4.29 (q, *J* = 7.1 Hz, 2H), 3.94 (s, 3H), 1.22 (t, *J* = 7.1 Hz, 3H). ^13^C NMR (100 MHz, CDCl_3_) δ 167.1, 160.1 (d, ^4^*J*_CF_ = 2.6 Hz), 153.7 (d, ^1^*J*_CF_ = 256.3 Hz), 134.3 (d, ^3^*J*_CF_ = 2.7 Hz), 130.5, 129.6, 129.2, 128.0 (dq, ^1^*J*_CF_ = 312.6, ^3^*J*_CF_ = 2.9 Hz), 120.7 (d, ^3^*J*_CF_ = 2.6 Hz), 117.2 (d, ^2^*J*_CF_ = 11.1 Hz), 98.9 (dq, ^2^*J*_CF_ = 26.6, ^3^*J*_CF_ = 2.6 Hz) 61.9, 52.3, 14.1. ^19^F NMR (376 MHz, CDCl_3_) δ −44.37 (d, ^5^*J*_FF_ = 2.3 Hz, 3F), −154.70 (s, 1F). HRMS (ESI-TOF) *m*/*z*: [M + Na]^+^ calcd. for C_16_H_13_F_4_NNaO_4_S^+^ 414.0394; found 414.0397.
**Ethyl 3-(4-cyanophenyl)-4-fluoro-5-((trifluoromethyl)thio)-1*H*-pyrrole-2-carboxylate** (**4g**). Eluents: hexane/CH_2_Cl_2_ (3:1, 1:1). Yield: 78.9 mg (66%) obtained from 105.9 mg (0.336 mmol) of pyrrole **2g**. Colorless solid, mp 155–160 °C. ^1^H NMR (400 MHz, CDCl_3_) δ 10.04 (br s, 1H), 7.75–7.66 (m, 2H), 7.61 (d, *J* = 8.0 Hz, 2H), 4.32 (q, *J* = 7.1 Hz, 2H), 1.25 (t, *J* = 7.1 Hz, 3H). ^13^C NMR (100 MHz, CDCl_3_) δ 160.0 (d, ^4^*J*_CF_ = 2.9 Hz), 153.6 (d, ^1^*J*_CF_ = 256.6 Hz), 134.5 (d, ^3^*J*_CF_ = 2.9 Hz), 131.7, 131.2, 128.0 (dd, ^1^*J*_CF_ = 312.3, ^3^*J*_CF_ = 3.1 Hz), 120.7 (d, ^3^*J*_CF_ = 2.6 Hz), 118.9, 116.3 (d, ^2^*J*_CF_ = 11.0 Hz), 111.7, 99.3 (dq, ^2^*J*_CF_ = 26.5, ^3^*J*_CF_ = 2.6 Hz), 62.0, 14.1. ^19^F NMR (376 MHz, CDCl_3_) δ −44.25 (d, ^5^*J*_FF_ = 2.4 Hz, 3F), −154.66 (s, 1F). HRMS (ESI) *m*/*z*: [M + Na]^+^ calcd. for C_15_H_10_F_4_N_2_NaO_2_S^+^ 381.0291; found 381.0294.
**Ethyl 4-fluoro-3-(4-nitrophenyl)-5-((trifluoromethyl)thio)-1*H*-pyrrole-2-carboxylate** (**4h**). Eluents: hexane/CH_2_Cl_2_ (5:1, 3:1, 1:1). Yield: 246.4 mg (87%) obtained from 250.8 mg (0.748 mmol) of pyrrole **2h**. Pale orange solid, mp 163–165 °C. ^1^H NMR (400 MHz, CDCl_3_) δ 10.28 (br s, 1H), 8.27 (d, *J* = 8.8 Hz, 2H), 7.67 (d, *J* = 8.3 Hz, 2H), 4.35 (q, *J* = 7.1 Hz, 2H), 1.25 (t, *J* = 7.1 Hz, 3H). ^13^C NMR (100 MHz, CDCl_3_) δ 160.2 (d, ^4^*J*_CF_ = 2.7 Hz), 153.6 (d, ^1^*J*_CF_ = 256.9 Hz), 147.4, 136.5 (d, ^3^*J*_CF_ = 2.8 Hz), 131.4, 127.9 (dq, ^1^*J*_CF_ = 312.2, ^3^*J*_CF_ = 3.2 Hz), 123.2, 120.7 (d, ^3^*J*_CF_ = 2.6 Hz), 115.9 (d, ^2^*J*_CF_ = 11.0 Hz), 99.5 (dq, ^2^*J*_CF_ = 26.4, ^3^*J*_CF_ = 2.8 Hz), 62.2, 14.1. ^19^F NMR (376 MHz, CDCl_3_) δ −44.25–−44.39 (m, 3F), −154.49–−154.89 (m, 1F). HRMS (ESI) *m*/*z*: [M + H]^+^ calcd. for C_14_H_11_F_4_N_2_O_4_S^+^ 379.0370; found 379.0370.
**Ethyl 4-fluoro-3-(2-nitrophenyl)-5-((trifluoromethyl)thio)-1*H*-pyrrole-2-carboxylate** (**4i**). Eluents: hexane/CH_2_Cl_2_ (5:1, 3:1, 1:1). Yield: 83.3 mg (76%) obtained from 96.9 mg (0.289 mmol) of pyrrole **2i**. Yellowish solid, mp 120–123 °C. ^1^H NMR (400 MHz, CDCl_3_) δ 10.22 (br s, 1H), 8.11 (dd, *J* = 8.2, 1.0 Hz, 1H), 7.66 (td, *J* = 7.5, 1.2 Hz, 1H), 7.56 (td, *J* = 8.1, 1.4 Hz, 1H), 7.48 (d, *J* = 7.6 Hz, 1H), 4.18 (dq, *J* = 7.1, 2.5 Hz, 2H), 1.08 (t, *J* = 7.1 Hz, 3H). ^13^C NMR (100 MHz, CDCl_3_) δ 160.1 (d, ^4^*J*_CF_ = 2.8 Hz), 153.4 (d, ^1^*J*_CF_ = 255.5 Hz), 149.3, 133.5, 132.8, 129.4, 128.0 (dq, ^1^*J*_CF_ = 312.4, ^3^*J*_CF_ = 3.1 Hz), 125.3 (d, ^3^*J*_CF_ = 2.3 Hz), 124.7, 120.7 (d, ^3^*J*_CF_ = 2.6 Hz), 113.5 (d, ^2^*J*_CF_ = 12.4 Hz), 99.2 (dq, ^2^*J*_CF_ = 26.3, ^3^*J*_CF_ = 2.6 Hz), 62.0, 13.7. ^19^F NMR (376 MHz, CDCl_3_) δ −44.37–−44.54 (m, 3F), −153.81–−154.09 (m, 1F). HRMS (ESI) *m*/*z*: [M + H]^+^ calcd. for C_14_H_11_F_4_N_2_O_4_S^+^ 379.0370; found 379.0370.
**Ethyl 3-(4-(tert-butyl)phenyl)-4-fluoro-5-((trifluoromethyl)thio)-1*H*-pyrrole-2-carboxylate** (**4j**). Eluent: hexane/CH_2_Cl_2_ (3:1). Yield: 36.2 mg (68%) obtained from 47.1 mg (0.138 mmol) of pyrrole **2j**. White solid, mp 135–137 °C. ^1^H NMR (400 MHz, CDCl_3_) δ 9.43 (br s, 1H), 7.44 (s, 4H), 4.30 (q, *J* = 7.1 Hz, 2H), 1.36 (s, 9H), 1.25 (t, *J* = 7.1 Hz, 3H). ^13^C NMR (100 MHz, CDCl_3_) δ 160.2 (d, ^4^*J*_CF_ = 3.0 Hz), 153.9 (d, ^1^*J*_CF_ = 255.4 Hz), 151.1, 130.01 (s), 128.0 (dq, ^1^*J*_CF_ = 312.2, ^3^*J*_CF_ = 3.4 Hz), 126.4 (d, ^3^*J*_CF_ = 2.9 Hz), 124.9, 120.5 (d, ^3^*J*_CF_ = 3.3 Hz), 118.5 (d, ^2^*J*_CF_ = 11.3 Hz), 98.3 (dq, ^2^*J*_CF_ = 26.9, ^3^*J*_CF_ = 2.8 Hz), 61.6, 34.8, 31.5, 14.1. ^19^F NMR (376 MHz, CDCl_3_) δ −44.45 (d, ^5^*J*_FF_ = 2.3 Hz, 3F), −154.94 (s, 1F). HRMS (ESI) *m*/*z*: [M + Na]^+^ calcd. for C_18_H_19_F_4_NO_2_SNa^+^ 412.0965; found 412.0964.
**Ethyl 4-fluoro-5-((trifluoromethyl)thio)-3-(2,4,5-trimethylphenyl)-1*H*-pyrrole-2-carboxylate** (**4k**). Eluents: hexane/CH_2_Cl_2_ (10:1, 5:1, 3:1). Yield: 45.2 mg (89%) obtained from 45.0 mg (0.135 mmol) of pyrrole **2k**. White solid, mp 97–103 °C. ^1^H NMR (400 MHz, CDCl_3_) δ 9.66 (br s, 1H), 7.04 (s, 1H), 7.00 (s, 1H), 4.32–4.13 (m, 2H), 2.27 (s, 3H), 2.24 (s, 3H), 2.13 (s, 3H), 1.14 (t, *J* = 7.1 Hz, 3H). ^13^C NMR (100 MHz, CDCl_3_) δ 160.4 (d, ^4^*J*_CF_ = 2.7 Hz), 154.0 (d, ^1^*J*_CF_ = 254.4 Hz), 136.7, 134.5, 133.2, 132.2, 131.2, 131.2 (dq, ^1^*J*_CF_ = 312.4, ^3^*J*_CF_ = 3.1 Hz), 126.5 (d, ^3^*J*_CF_ = 2.2 Hz), 121.2 (d, ^3^*J*_CF_ = 3.8 Hz), 117.8 (d, ^2^*J*_CF_ = 13.7 Hz), 98.1 (dq, ^2^*J*_CF_ = 27.3, ^3^*J*_CF_ = 2.6 Hz), 61.3, 19.7, 19.4 (d, ^5^*J*_CF_ = 1.0 Hz), 19.3, 14.0. ^19^F NMR (376 MHz, CDCl_3_) δ −44.69 (d, ^5^*J*_FF_ = 2.3 Hz, 3F), −152.99 (s, 1F). HRMS (ESI) *m*/*z*: [M + H]^+^ calcd. for C_17_H_18_F_4_NO_2_S^+^ 376.0989; found 376.0990.
**Ethyl 4-fluoro-3-(4-methoxyphenyl)-5-((trifluoromethyl)thio)-1*H*-pyrrole-2-carboxylate** (**4l**). Eluents: hexane/CH_2_Cl_2_ (5:1, 3:1, 1:1). Yield: 93.0 mg (82%) obtained from 100.0 mg (0.312 mmol) of pyrrole **2l**. Off-white solid, mp 142–147 °C. ^1^H NMR (400 MHz, CDCl_3_) δ 9.44 (br s, 1H), 7.43 (d, *J* = 8.0 Hz, 2H), 6.99–6.91 (m, 2H), 4.30 (q, *J* = 7.1 Hz, 2H), 3.85 (s, 3H), 1.26 (t, *J* = 7.1 Hz, 3H). ^13^C NMR (100 MHz, CDCl_3_) δ 160.3 (d, ^4^*J*_CF_ = 3.0 Hz), 159.5, 153.9 (d, ^1^*J*_CF_ = 254.7 Hz), 131.6, 128.0 (dq, ^1^*J*_CF_ = 312.4, ^3^*J*_CF_ = 3.5 Hz), 121.6 (d, ^3^*J*_CF_ = 2.7 Hz), 120.4 (d, ^3^*J*_CF_ = 2.9 Hz), 118.2 (d, ^2^*J*_CF_ = 11.2 Hz), 113.5, 98.3 (dq, ^2^*J*_CF_ = 26.9, ^3^*J*_CF_ = 2.5 Hz), 61.6, 55.4, 14.2. ^19^F NMR (376 MHz, CDCl_3_) δ −44.48 (d, ^5^*J*_FF_ = 2.3 Hz, 3F), −155.46 (s, 1F). HRMS (ESI) *m*/*z*: [M + H]^+^ calcd. for C_15_H_14_F_4_NO_3_S^+^ 364.0625; found 364.0624.
**N-butyl-4-fluoro-3-phenyl-5-thiocyanato-1*H*-pyrrole-2-carboxamide** (**4m**). Eluents: hexane/CH_2_Cl_2_ (1:1), DCM. Yield 51.6 mg (48%) obtained from 95.3 mg (0.3 mmol) of pyrrole **3m**. Pale yellow solid, mp 105–110 °C. ^1^H NMR (400 MHz, CDCl_3_) δ 11.18 (br s, 1H), 7.55–7.41 (m, 5H), 5.77 (t, *J* = 5.2 Hz, 1H), 3.31 (dd, *J* = 12.7, 6.8 Hz, 2H), 1.39–1.28 (m, 2H), 1.21–1.08 (m, 2H), 0.84 (t, *J* = 7.3 Hz, 3H). ^13^C NMR (100 MHz, CDCl_3_) δ 160.0 (d, ^4^*J*_CF_ = 2.4 Hz), 153.6 (d, ^1^*J*_CF_ = 254.8 Hz), 130.5, 129.9 (d, ^3^*J*_CF_ = 1.9 Hz), 129.5, 129.0, 128.2 (dq, ^1^*J*_CF_ = 311.9, ^3^*J*_CF_ = 3.0 Hz), 123.9, 113.2 (d, ^2^*J*_CF_ = 12.8 Hz), 96.6 (dd, ^2^*J*_CF_ = 26.0, ^3^*J*_CF_ = 2.8 Hz), 39.3, 31.1, 20.0, 13.7. ^19^F NMR (376 MHz, CDCl_3_) δ −44.95 (d, ^5^*J*_FF_ = 1.8 Hz), −156.47 (d, ^5^*J*_FF_ = 1.8 Hz). HRMS (ESI) *m*/*z*: [M + H]^+^ calcd. for C_16_H_17_F_4_N_2_OS^+^ 361.0992, found 361.0994.
**Ethyl 4-methyl-3-phenyl-5-((trifluoromethyl)thio)-1*H*-pyrrole-2-carboxylate** (**4n**). Eluents: hexane/CH_2_Cl_2_ (10:1, 5:1, 3:1). Yield: 23.7 mg (55%) obtained from 37.8 mg of pyrrole **2n**. Off-white solid, mp 125–130 °C. ^1^H NMR (400 MHz, CDCl_3_) δ 9.40 (br s, 1H), 7.42–7.27 (m, 5H), 4.19 (q, *J* = 7.1 Hz, 2H), 2.08 (s, 3H), 1.14 (t, *J* = 7.1 Hz, 3H). ^13^C NMR (100 MHz, CDCl_3_) δ 160.5, 134.1, 131.5, 130.7, 130.3, 128.7 (q, ^1^*J*_CF_ = 311.7 Hz), 127.8, 127.4, 123.4, 110.9 (d, ^3^*J*_CF_ = 2.5 Hz), 60.9, 14.1, 10.4. ^19^F NMR (376 MHz, CDCl_3_) δ −44.30 (s). HRMS (ESI) *m*/*z*: [M + Na]^+^ calcd. for C_15_H_14_F_3_NNaO_2_S^+^ 352.0590; found 352.0607.
**Ethyl 3-(4-methoxyphenyl)-4-methyl-5-((trifluoromethyl)thio)-1*H*-pyrrole-2-carboxylate** (**4o**). Eluents: hexane/CH_2_Cl_2_ (5:1, 3:1, 1:1). Yield: 30.6 mg (64%) obtained from 42.0 mg of pyrrole **2o**. Off-white solid, mp 161–166 °C. ^1^H NMR (400 MHz, CDCl_3_) δ 9.39 (br s, 1H), 7.26–7.21 (m, 2H), 6.96–6.90 (m, 2H), 4.21 (q, *J* = 7.1 Hz, 2H), 3.85 (s, 3H), 2.08 (s, 3H), 1.18 (t, *J* = 7.1 Hz, 3H). ^13^C NMR (100 MHz, CDCl_3_) δ 160.5, 158.9, 131.5, 131.3, 130.8, 128.6 (q, ^1^*J*_CF_ = 311.7 Hz), 126.2, 123.3, 113.2, 110.8 (d, ^3^*J*_CF_ = 2.4 Hz), 60.9, 55.4, 14.2, 10.5. ^19^F NMR (376 MHz, CDCl_3_) δ −44.37 (s). HRMS (ESI) *m*/*z*: [M + H]^+^ calcd. for C_16_H_17_F_3_NO_3_S 360.0876; found 360.0876.
**2-((Trimethylsilyl)oxy)ethyl 4-fluoro-3-phenyl-5-((trifluoromethyl)thio)-1*H*-pyrrole-2-carboxylate** (**4p**). Eluents: hexane/CH_2_Cl_2_ (1:1, 1:3). Yield: 18.8 mg (53%) obtained from 26 mg (0.085 mmol) of pyrrole **2p**. White solid, mp 115–120 °C. ^1^H NMR (400 MHz, CDCl_3_) δ 9.44 (br s, 1H), 7.54–7.48 (m, 2H), 7.44–7.33 (m, 3H), 4.33–4.28 (m, 2H), 3.79–3.73 (m, 2H), 0.09 (s, 9H). ^13^C NMR (100 MHz, CDCl_3_) δ 160.0 (d, ^4^*J*_CF_ = 3.0 Hz), 155.1, 152.6, 130.4, 129.3 (d, *J* = 2.8 Hz), 128.1, 128.0, 128.0 (dq, ^1^*J*_CF_ = 312.2, ^3^*J*_CF_ = 3.3 Hz), 120.1 (d, ^3^*J*_CF_ = 3.2 Hz), 118.7 (d, ^2^*J*_CF_ = 11.2 Hz), 98.6 (dq, ^2^*J*_CF_ = 27.1, ^3^*J*_CF_ = 2.3 Hz), 66.5, 60.4, −0.51. ^19^F NMR (376 MHz, CDCl_3_) δ −44.30–−44.49 (m, 3F), −154.78–−155.42 (m, 1F). HRMS (ESI) *m*/*z*: [M + Na]^+^ calcd. for C_17_H_19_F_4_NNaO_3_SSi^+^ 444.0683; found 444.0686.
**Ethyl 5-((diethoxyphosphoryl)thio)-4-fluoro-3-phenyl-1*H*-pyrrole-2-carboxylate** (**5**). A vial was charged with pyrrole **2a** (43.3 mg, 0.15 mmol, 1.0 mol equiv) and MeCN (1 mL). To the resulting solution, a mixture of DBU (22.8 mg, 0.15 mmol, 1.0 mol equiv) and diethyl phosphite (31.1 mg, 0.23 mmol, 1.5 mol equiv) was added. The reaction mixture was stirred at room temperature overnight until the starting material was fully consumed (monitored by TLC). The mixture was then concentrated under reduced pressure. The residue was purified by column chromatography on silica gel (eluents: CH_2_Cl_2_, CH_2_Cl_2_/EtOAc (20:1)) to afford product **5** (17.3 mg, 29% yield) as a colorless oil. ^1^H NMR (400 MHz, CDCl_3_) δ 9.24 (br s, 1H), 7.51–7.45 (m, 2H), 7.43–7.31 (m, 3H), 4.33–4.20 (m, 6H), 1.38 (t, *J* = 7.1 Hz, 3H), 1.37 (t, *J* = 7.1 Hz, 3H), 1.23 (t, *J* = 7.1 Hz, 3H). ^13^C NMR (100 MHz, CDCl_3_) δ 160.1 (pseudo-t, ^4^*J*_CF_ = ^6^*J*_CP_ = 2.4 Hz), 152.1 (dd, ^1^*J*_CF_ = 249.8, ^3^*J*_CP_ = 6.8 Hz), 130.4, 130.3, 129.9 (d, ^3^*J*_CF_ = 2.5 Hz), 127.9, 118.6 (pseudo-t, ^3^*J*_CF_ = ^5^*J*_CP_ = 3.6 Hz), 118.3 (dd, ^2^*J*_CF_ = 11.5, ^4^*J*_CP_ = 3.5 Hz), 100.00 (dd, ^2^*J*_CF_ = 26.8, ^2^*J*_CP_ = 8.2 Hz), 65.1, 65.0, 61.1, 16.2, 16.1, 14.1. ^31^P{^1^H} NMR (162 MHz, CDCl_3_) δ 19.02 (d, ^4^*J*_PF_ = 6.8 Hz). ^19^F NMR (376 MHz, DMSO) δ −158.81–−158.87 (m). HRMS (ESI) *m*/*z*: [M + H]^+^ calcd. for C_17_H_22_FNO_5_PS^+^ 402.0935, found 402.0938.
**Ethyl 5-((1H-tetrazol-5-yl)thio)-4-fluoro-3-phenyl-1*H*-pyrrole-2-carboxylate** (**6**). To a solution of pyrrole **2a** (57 mg, 0.196 mmol, 1.0 mol equiv) in DMSO (1 mL) was added NaN_3_ (18.2 mg, 0.281 mmol, 1.5 equiv). The reaction mixture was stirred at 80 °C for 8 h until the starting material was consumed (monitored by TLC). The mixture was then quenched with water (20 mL) and extracted with EtOAc (3 × 20 mL). The combined organic phases were dried over anhydrous Na_2_SO_4_, filtered, and concentrated under reduced pressure. The residue was dried in vacuo to afford pure sulfenyl tetrazole **6** (65.4 mg, quant.) as a pale orange solid, mp 195–198 °C. ^1^H NMR (400 MHz, DMSO-d6) δ 12.82 (br s, 1H), 7.47–7.31 (m, 1H), 4.16 (q, *J* = 7.1 Hz, 1H), 1.14 (t, *J* = 7.1 Hz, 1H). The NH proton of the tetrazole ring was not detected. ^13^C NMR (100 MHz, DMSO-d6) δ 159.5 (d, ^4^*J*_CF_ = 3.2 Hz), 153.9, 151.4 (d, ^1^*J*_CF_ = 248.4 Hz), 130.1, 130.0 (d, ^4^*J*_CF_ = 2.6 Hz), 127.8, 127.5, 119.1 (d, ^4^*J*_CF_ = 4.0 Hz), 116.7 (d, ^2^*J*_CF_ = 11.3 Hz), 100.6 (d, ^2^*J*_CF_ = 26.0 Hz), 60.4, 13.9. ^19^F NMR (376 MHz, DMSO-d6) δ −160.24 (s). HRMS (ESI) *m*/*z*: [M + H]^+^ calcd. for C_14_H_13_FN_5_O_2_S^+^ 334.0768, found 334.0771.
**Ethyl 4-fluoro-3-phenyl-5-((trifluoromethyl)sulfonyl)-1*H*-pyrrole-2-carboxylate** (**7**). To a solution of pyrrole **4a** (51.8 mg, 0.155 mmol, 1.0 mol equiv) in TFA (0.6 mL) was added dropwise (30% aq., 0.6 mL, 5.82 mmol, 39 mol equiv). The mixture was stirred at room temperature overnight until the reaction was complete (TLC monitoring). The reaction mixture was then concentrated under reduced pressure. The residue was purified by column chromatography on silica gel (eluents: Hex/DCM (1:1), DCM) to afford sulfone **7** (44.9 mg, 79%) as an off-white solid. Mp: 139–143 °C. ^1^H NMR (400 MHz, CDCl_3_) δ 10.08 (br s, 1H), 7.52–7.35 (m, 5H), 4.34 (q, *J* = 7.1 Hz, 2H), 1.23 (t, *J* = 7.1 Hz, 3H). ^13^C NMR (100 MHz, CDCl_3_) δ 159.8 (d, ^4^*J*_CF_ = 1.9 Hz), 153.0 (d, ^1^*J*_CF_ = 269.2 Hz), 130.4, 128.8, 128.2, 127.7 (d, ^3^*J*_CF_ = 1.9 Hz), 124.3 (d, ^3^*J*_CF_ = 1.7 Hz), 119.8 (q, ^1^*J*_CF_ = 324.1 Hz), 119.1 (d, ^2^*J*_CF_ = 10.7 Hz), 106.8 (d, ^2^*J*_CF_ = 20.8 Hz), 62.8, 13.9. ^19^F NMR (376 MHz, DMSO-d6) δ −80.03 (d, ^5^*J*_FF_ = 2.9 Hz, 3F), −145.78 (q, ^5^*J*_FF_ = 2.9 Hz, 1F). HRMS (ESI) *m*/*z*: [M + H]^+^ calcd. for C_14_H_12_F_4_NO_4_S^+^ 366.0410, found 366.0421.
**4-Fluoro-3-phenyl-5-((trifluoromethyl)thio)-1H-pyrrole-2-carboxylic acid** (**8**). To a solution of pyrrole **4a** (149.1 mg, 0.447 mmol, 1.0 mol equiv) in MeOH (4.5 mL) was added LiOH·H_2_O (372.6 mg, 8.880 mmol, ~20 mol equiv). The reaction mixture was stirred in a sealed vial at 90 °C for 11 h until the reaction was complete (TLC monitoring). Upon completion, the mixture was acidified with 5% HCl (20 mL) and extracted with EtOAc (3 × 20 mL). The combined organic phases were dried over anhydrous Na_2_SO_4_, filtered, and concentrated under reduced pressure. The residue was purified by column chromatography on silica gel (gradient elution: Hex/DCM (1:1), DCM, DCM/EtOAc (10:1, 1:1)) to afford carboxylic acid **8** (112.3 mg, 82%) as a dark brown solid, mp 93–96 °C. ^1^H NMR (400 MHz, CDCl_3_) δ 9.83 (br s, 1H), 9.37 (s, 1H), 7.50 (d, *J* = 7.7 Hz, 2H), 7.46–7.35 (m, 3H). ^13^C NMR (100 MHz, CDCl_3_) δ 164.8 (d, ^4^*J*_CF_ = 2.6 Hz), 153.8 (d, ^1^*J*_CF_ = 256.1 Hz), 130.3, 128.8 (d, ^3^*J*_CF_ = 2.6 Hz), 128.4, 128.3, 127.9 (dq, ^1^*J*_CF_ = 312.2, ^3^*J*_CF_ = 3.0 Hz), 120.07 (d, ^2^*J*_CF_ = 11.4 Hz), 119.05 (d, ^3^*J*_CF_ = 2.8 Hz), 100.41 (dq, ^2^*J*_CF_ *=* 25.9, ^3^*J*_CF_ = 2.6 Hz). ^19^F NMR (376 MHz, CDCl_3_) δ −44.27 (d, ^5^*J*_FF_ = 2.2 Hz, 3F), −155.01 (s, 1F). HRMS (ESI) *m*/*z*: [M + Na]^+^ calcd. for C_12_H_7_F_4_NNaO_2_S^+^ 328.0026, found 328.0027.

## Figures and Tables

**Table 1:** Screening of S-nucleophiles in reaction with 2,5-dichloro-2*H*-pyrroles **A** ^a^.

Entry	Pyrrole	S-Nucleophile (mol equiv)	Base (mol equiv)	Yield 2, %	Yield 3, %
1	**1b**	PhCH_2_SH (3.0)	-	0	49 ^b^
2	**1b**	PhCH_2_SH (3.0)	DBU (3.0)	0	50 ^b^
3	**1b**	PhSH (3.0)	DBU(3.0)	0	90
4	**1b**	Pyridine-2-thiol (3.0)	DBU (3.0)	0	91
5	**1b**	Benzo[d]thiazole-2-thiol (1.2)	DBU (1.2)	0	94
6	**1a**	CH_3_C(O)SH (1.2)	Et_3_N (1.2)	0	86
7	**1a**	CH_3_C(O)SH (1.2)	K_2_CO_3_ (1.2)	0	72
8	**1a**	CH_3_C(O)SH (1.2)	DBU (1.2)	0	62
9	**1b**	HSCH_2_CO_2_Et (2)	K_2_CO_3_ (5)	0	73 ^b^
10	**1b**	HSCH_2_CO_2_Et (2)	K_2_CO_3_ (10)	0	73 ^b^
11	**1b**	HSCH_2_CO_2_Et (2)	DIPEA (5)	0	71 ^b^
12	**1a**	EtOCS_2_K (3.0)	-	0	94
13	**1a**	NH_4_SCN (1.0)	-	31	10 ^b^
14	**1a**	NH_4_SCN (2.0)	-	77	6
15	**1a**	NH_4_SCN (3.0)	-	79	2
16	**1b**	KSeCN (3.0)	-	0	98

^a^—reaction conditions: **1a** (0.2 mmol), TCCA (0.67 mmol) in MeCN (2 mL) at rt, overnight (16–18 h). ^b^—unidentified products were also observed on ^19^F NMR spectra.

## Data Availability

The original contributions presented in this study are included in the article and Supplementary Materials. Further inquiries can be directed to the corresponding authors.

## Supplementary Materials

The following supporting information can be downloaded at: https://www.mdpi.com/article/10.3390/ijms27125442/s1.
