# Supplementary material for: Efficient One-Pot Functionalization of Pyrroles via Dearomative Chlorination–Thiocyanation Strategy
Source: Int J Mol Sci. 2026 Jun 16;27(12):5442. doi: 10.3390/ijms27125442 (PMC13299718; doi:10.3390/ijms27125442)
Supplement: Supplementary file 1 [file ijms-27-05442-s001.zip › ijms-4351481-supplementary.pdf]

Supplementary Materials

Table of content

NMR spectra of compound **1p**	**S2**
NMR spectra of compound **2a**	**S5**
NMR spectra of compound **2b**	**S8**
NMR spectra of compound **2c**	**S11**
NMR spectra of compound **2d**	**S14**
NMR spectra of compound **2e**	**S17**
NMR spectra of compound **2f**	**S20**
NMR spectra of compound **2g**	**S23**
NMR spectra of compound **2h**	**S26**
NMR spectra of compound **2i**	**S29**
NMR spectra of compound **2j**	**S32**
NMR spectra of compound **2k**	**S35**
NMR spectra of compound **2l**	**S38**
NMR spectra of compound **2m**	**S41**
NMR spectra of compound **2n**	**S44**
NMR spectra of compound **2o**	**S46**
NMR spectra of compound **2p**	**S48**
NMR spectra of compound **2q**	**S51**
NMR spectra of compound **4a**	**S54**
NMR spectra of compound **4b**	**S57**
NMR spectra of compound **4c**	**S60**
NMR spectra of compound **4d**	**S63**
NMR spectra of compound **4e**	**S66**
NMR spectra of compound **4f**	**S69**
NMR spectra of compound **4g**	**S72**
NMR spectra of compound **4h**	**S75**
NMR spectra of compound **4i**	**S78**
NMR spectra of compound **4j**	**S81**
NMR spectra of compound **4k**	**S84**
NMR spectra of compound **4l**	**S87**
NMR spectra of compound **4m**	**S90**
NMR spectra of compound **4n**	**S93**
NMR spectra of compound **4o**	**S96**
NMR spectra of compound **4p**	**S99**
NMR spectra of compound **5**	**S102**
NMR spectra of compound **6**	**S106**
NMR spectra of compound **7**	**S109**
NMR spectra of compound **8**	**S112**

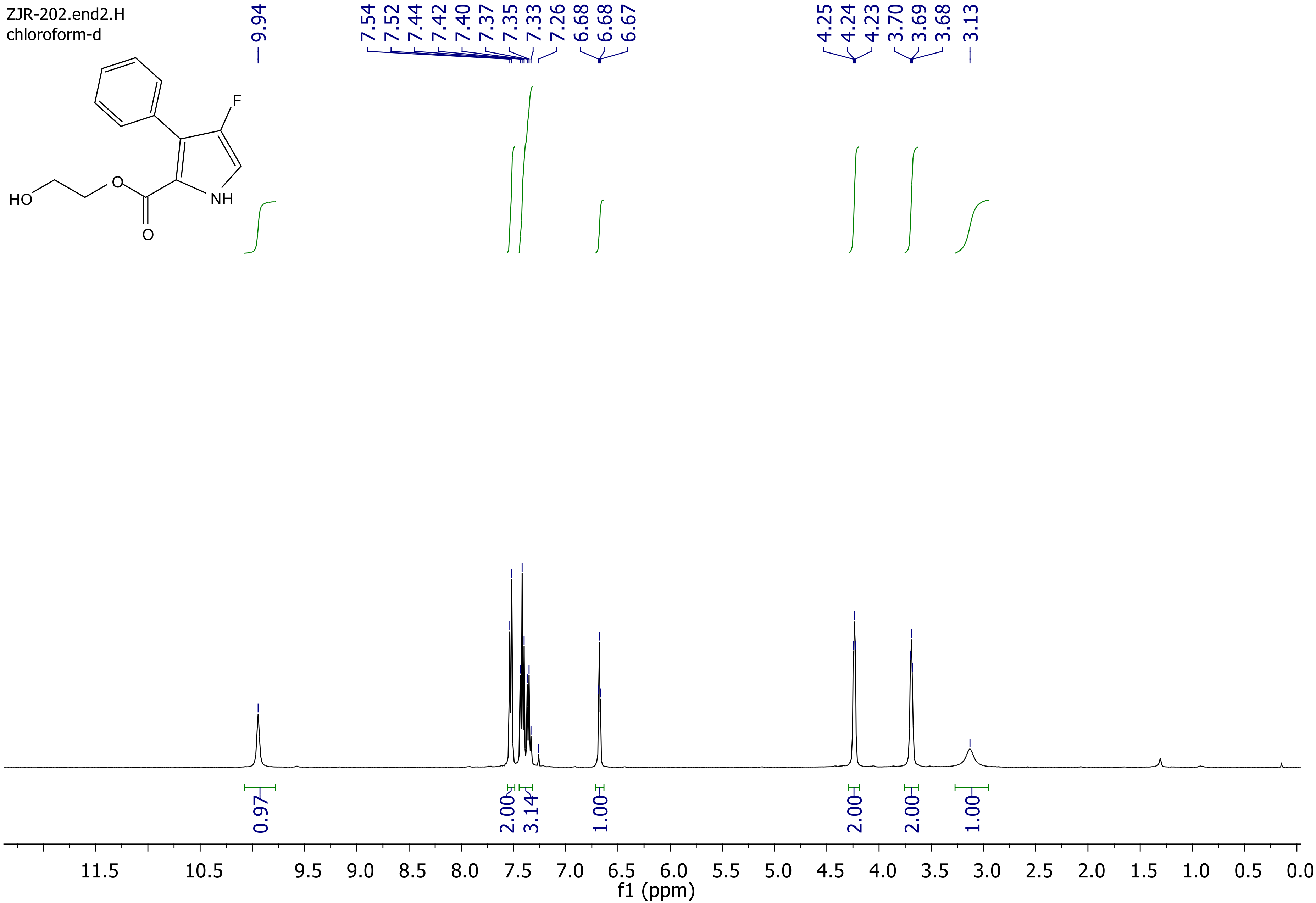

^{1}H NMR spectrum of 2-hydroxyethyl 4-fluoro-3-phenyl-1H-pyrrole-2-carboxylate (**1p**) in $CDCl_3$ at 400 MHz

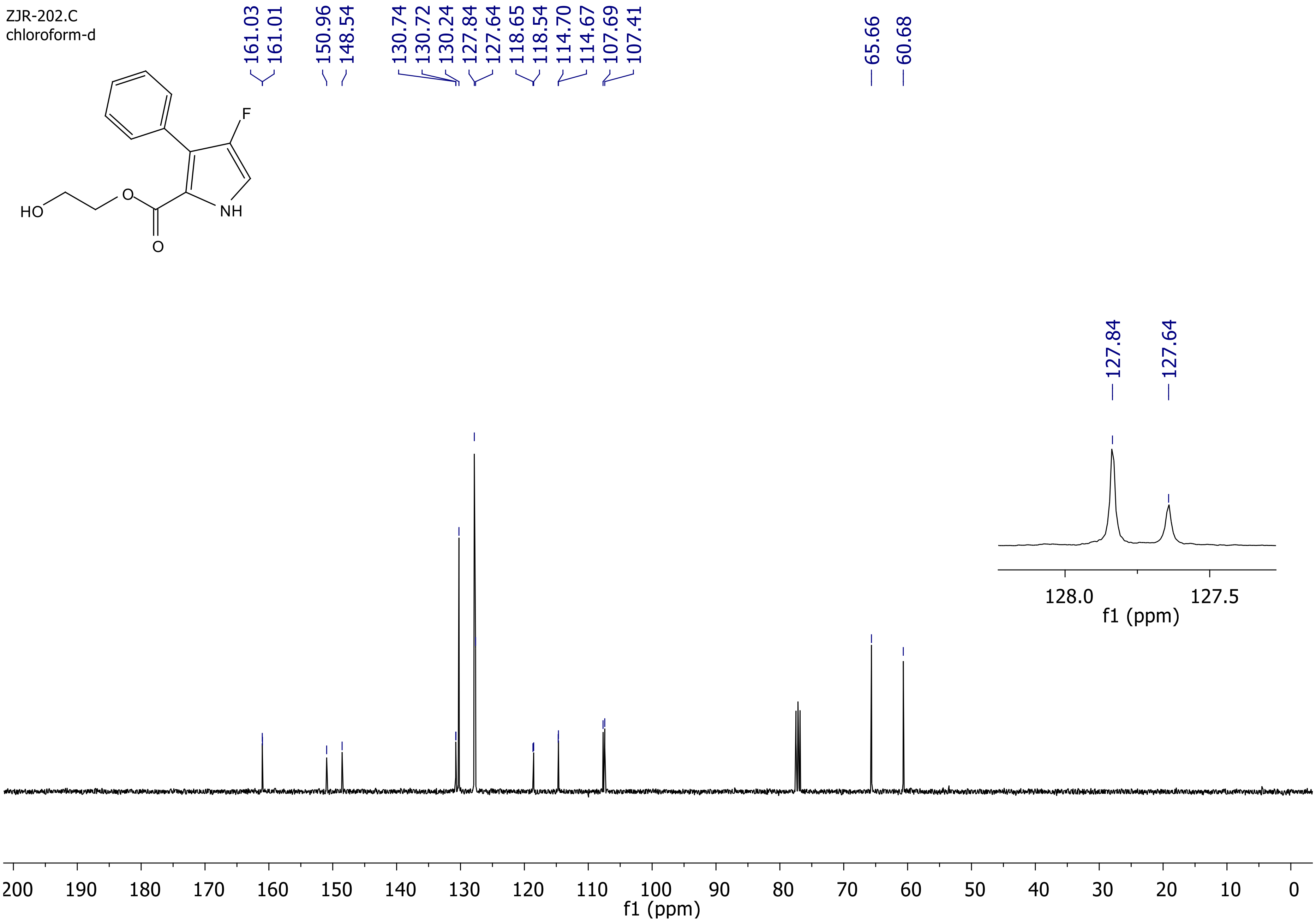

^{13}C NMR spectrum of 2-hydroxyethyl 4-fluoro-3-phenyl-1H-pyrrole-2-carboxylate (**1p**) in $CDCl_3$ at 100 MHz

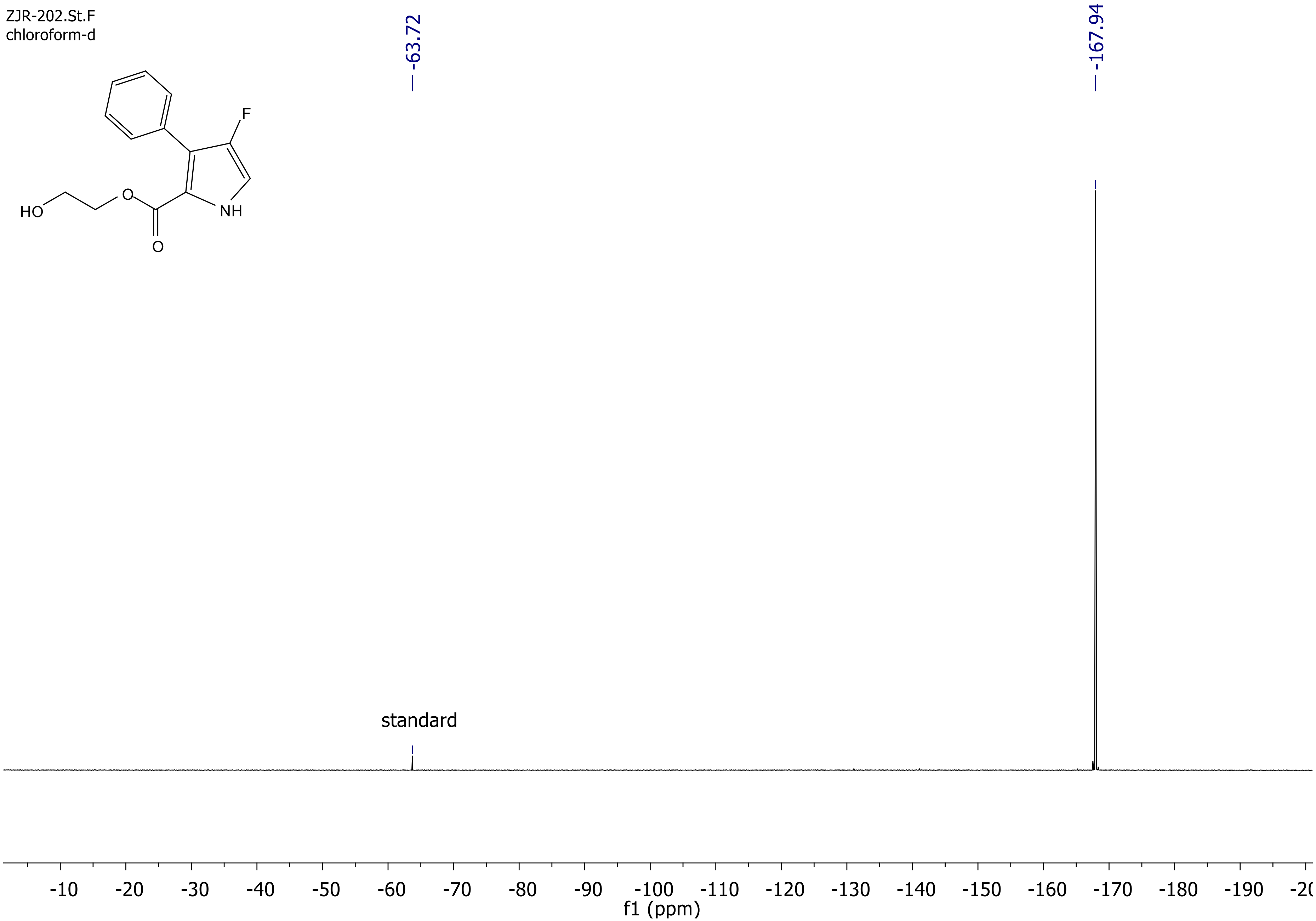

^{19}F NMR spectrum of 2-hydroxyethyl 4-fluoro-3-phenyl-1H-pyrrole-2-carboxylate (**1p**) in $CDCl_3$ at 376 MHz

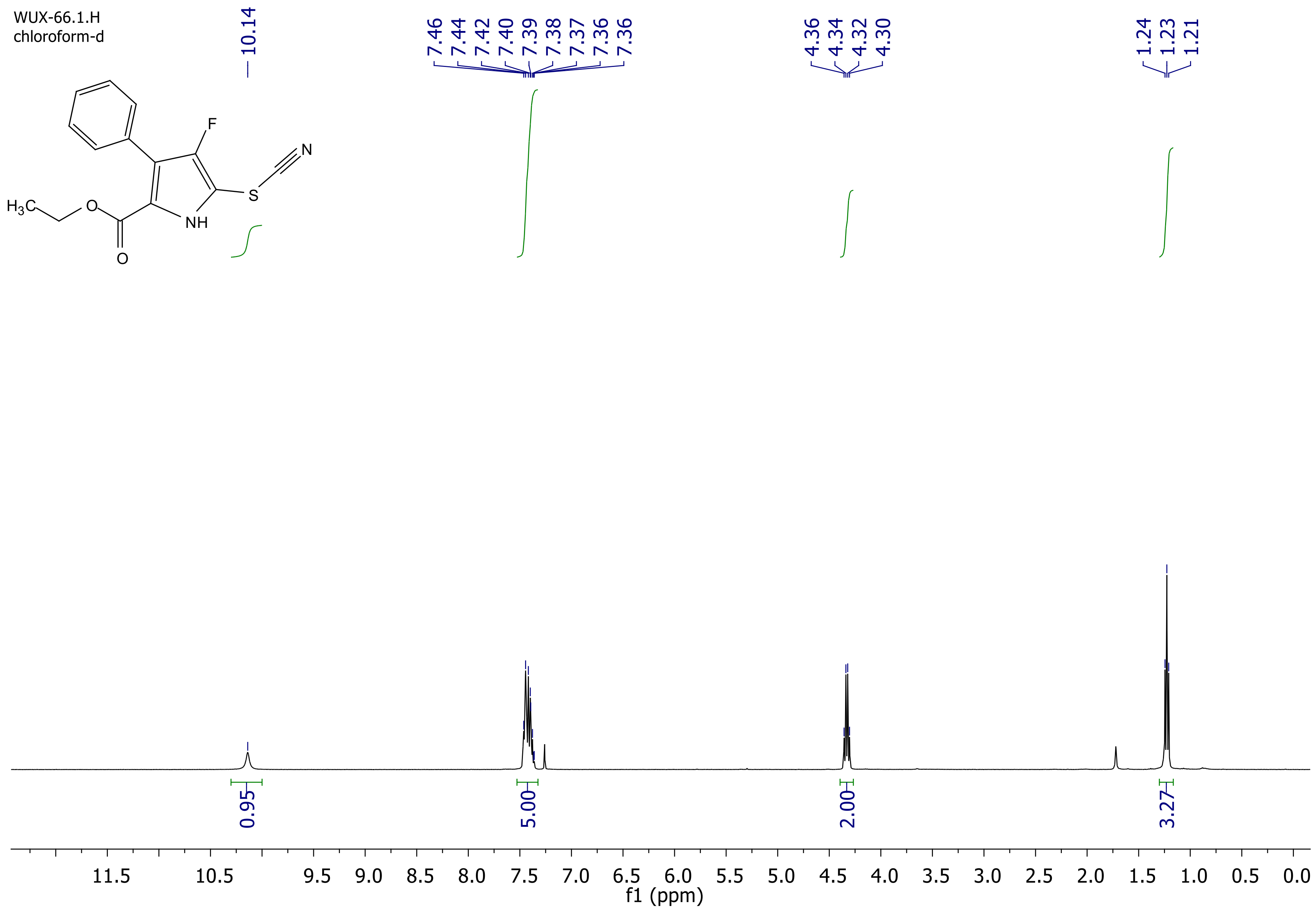

^{1}H NMR spectrum of ethyl 4-fluoro-3-phenyl-5-thiocyanato-1H-pyrrole-2-carboxylate (**2a**) in $CDCl_3$ at 400 MHz

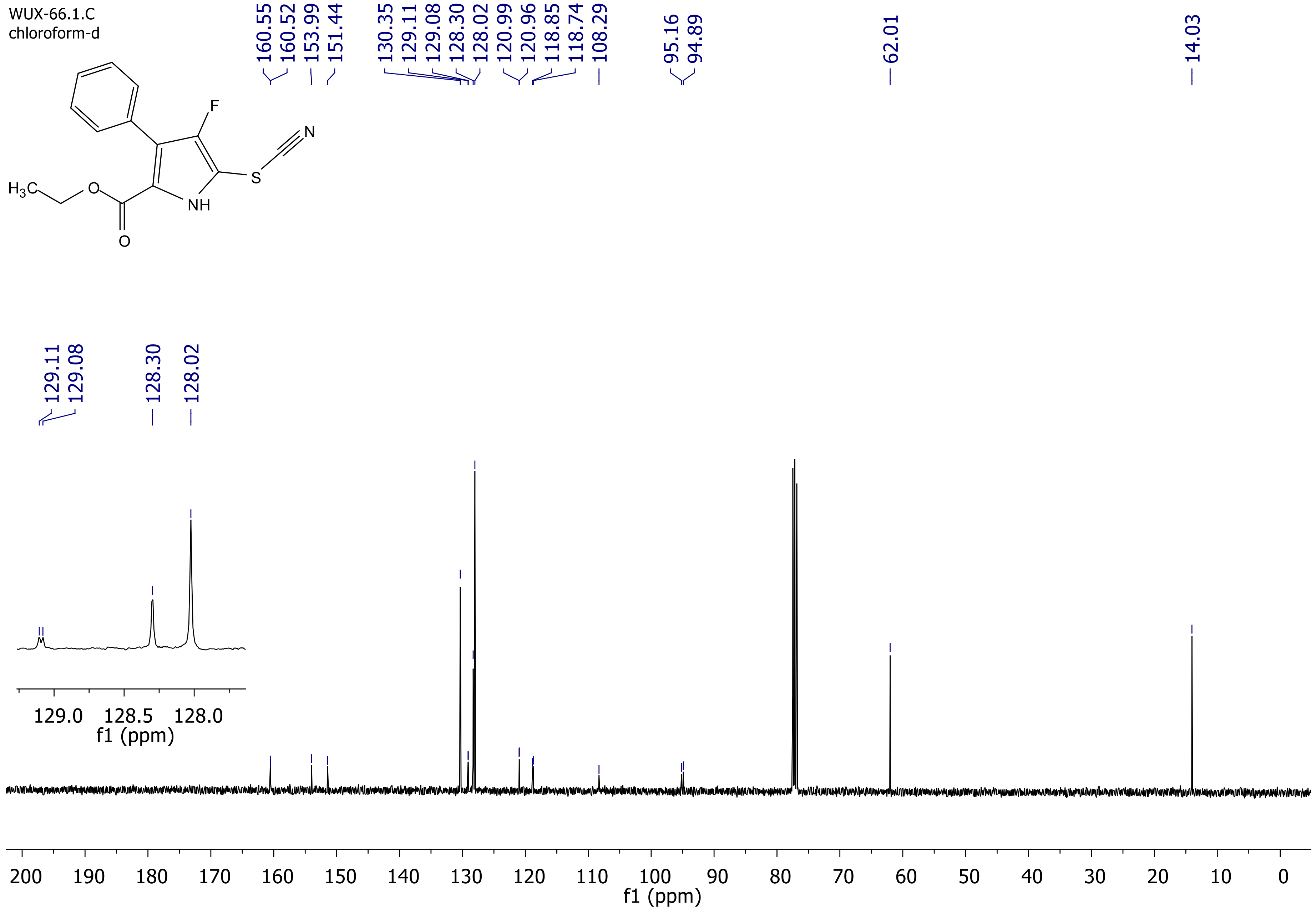

^{13}C NMR spectrum of ethyl 4-fluoro-3-phenyl-5-thiocyanato-1H-pyrrole-2-carboxylate (**2a**) in $CDCl_3$ at 100 MHz

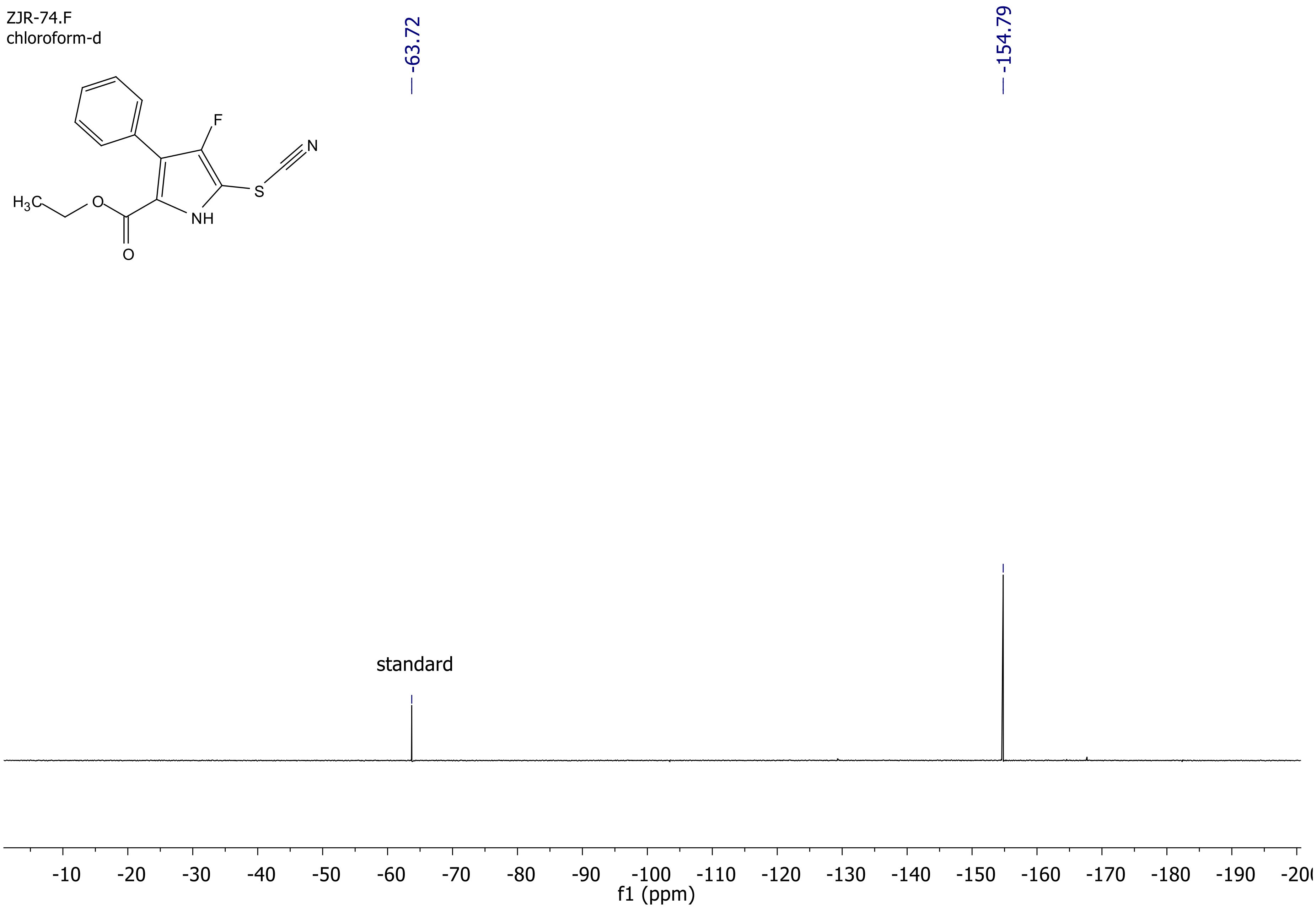

^{19}F NMR spectrum of ethyl 4-fluoro-3-phenyl-5-thiocyanato-1H-pyrrole-2-carboxylate (**2a**) in $CDCl_3$ at 376 MHz

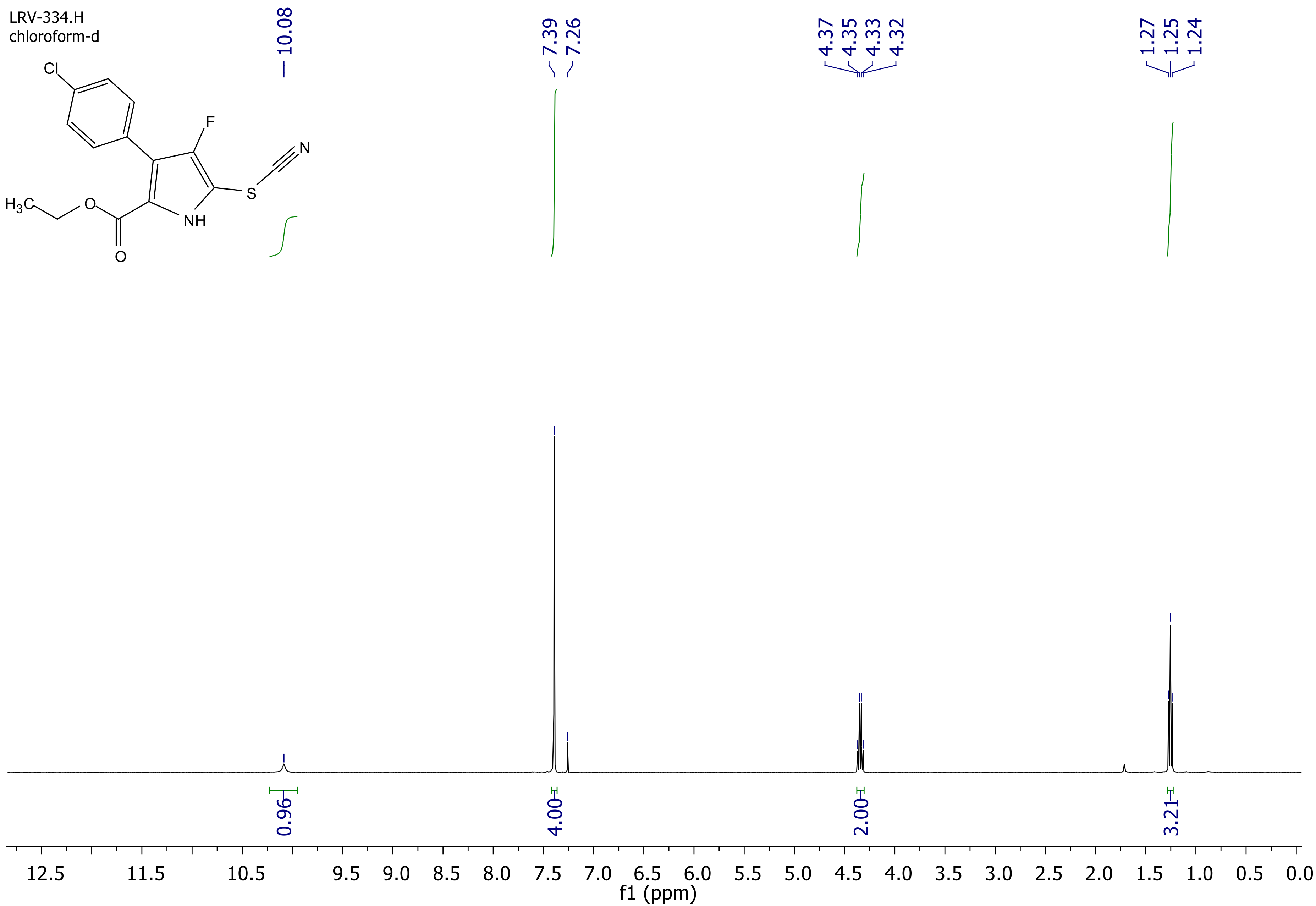

^{1}H NMR spectrum of ethyl 3-(4-chlorophenyl)-4-fluoro-5-thiocyanato-1H-pyrrole-2-carboxylate (2**b**) in $CDCl_3$ at 400 MHz

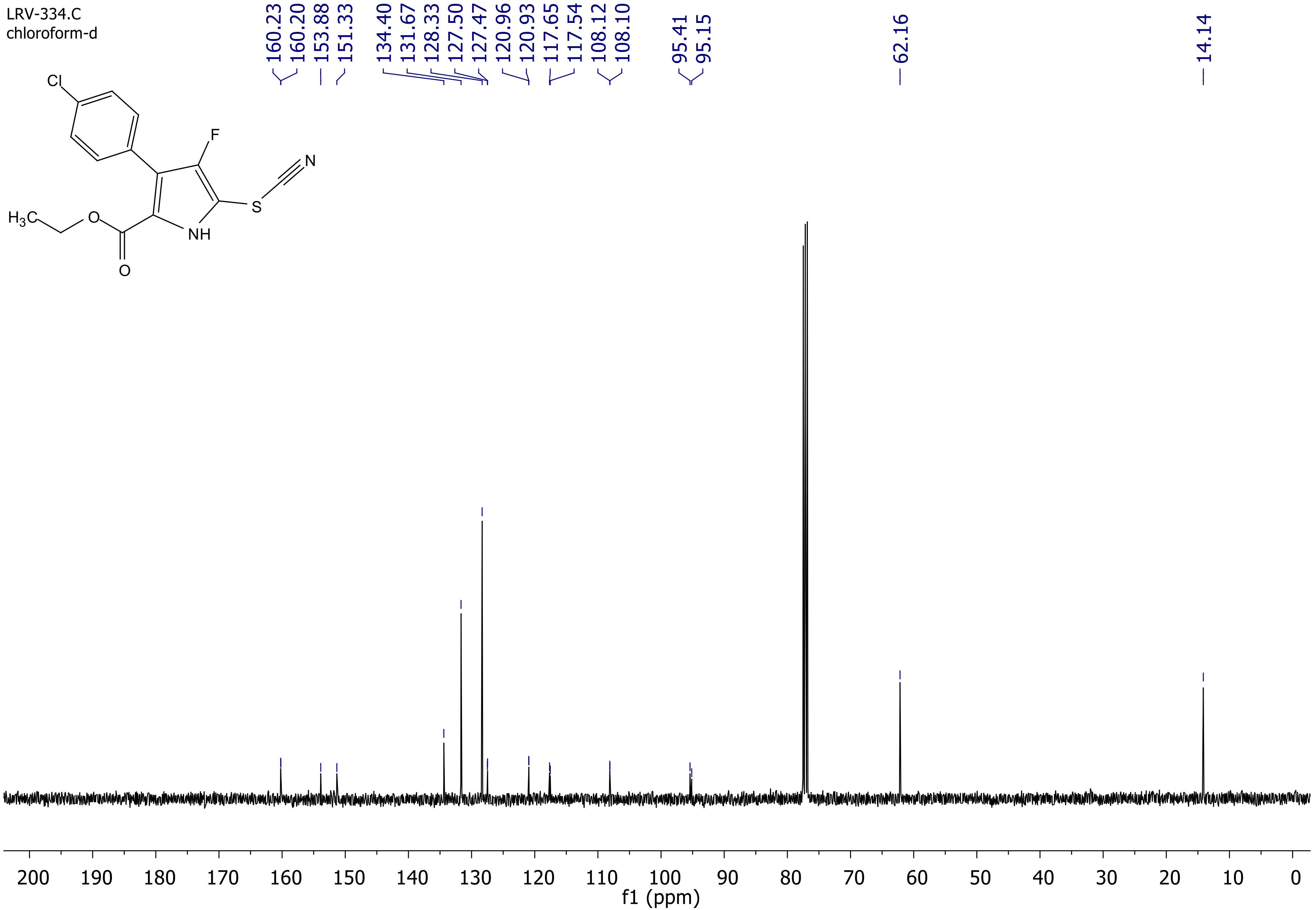

^{13}C NMR spectrum of ethyl 3-(4-chlorophenyl)-4-fluoro-5-thiocyanato-1H-pyrrole-2-carboxylate (2**b**) in $CDCl_3$ at 100 MHz

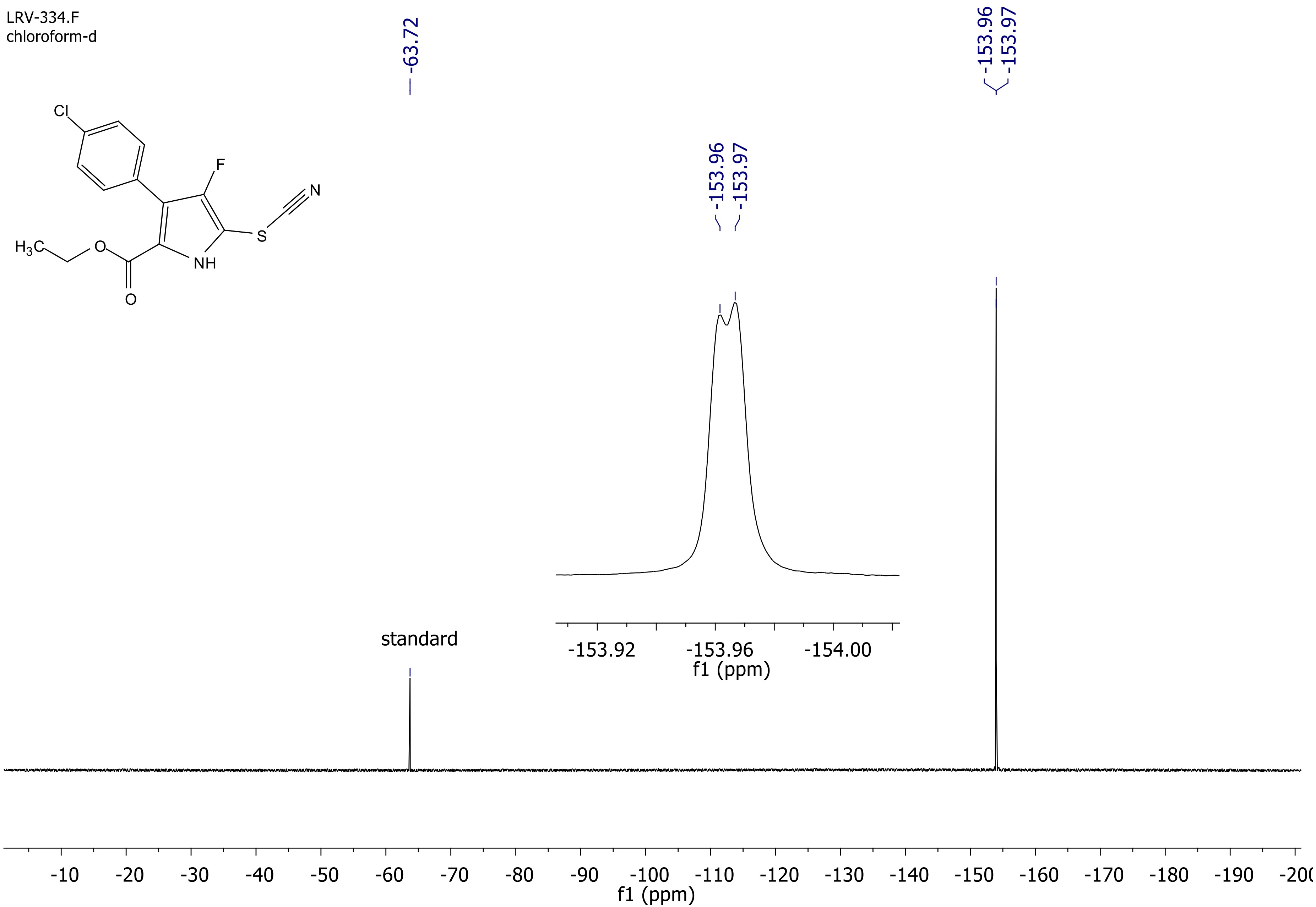

^{19}F NMR spectrum of ethyl 3-(4-chlorophenyl)-4-fluoro-5-thiocyanato-1H-pyrrole-2-carboxylate (2**b**) in $CDCl_3$ at 376 MHz

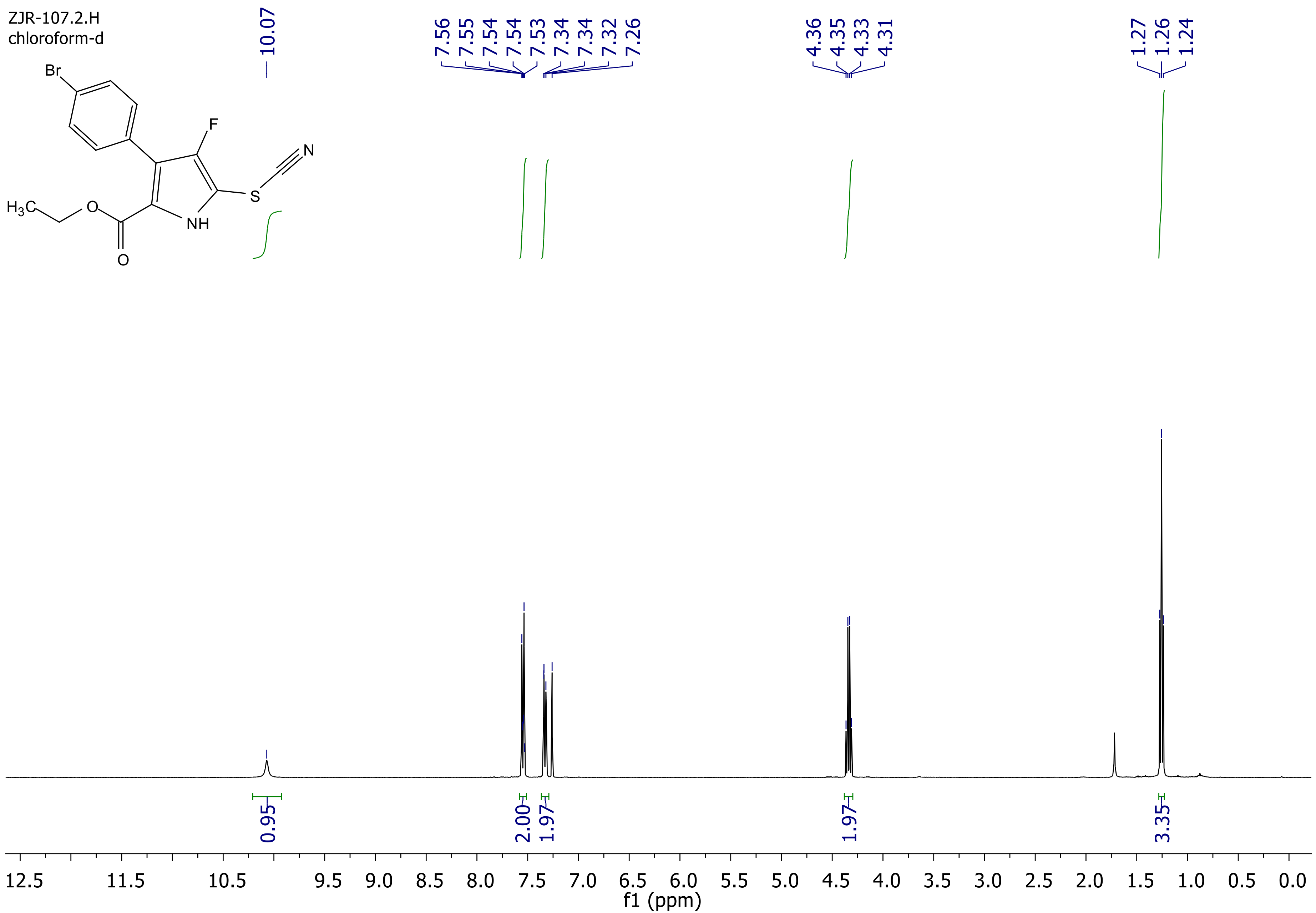

^{1}H NMR spectrum of ethyl 3-(4-bromophenyl)-4-fluoro-5-thiocyanato-1H-pyrrole-2-carboxylate (2**c**) in $CDCl_3$ at 400 MHz

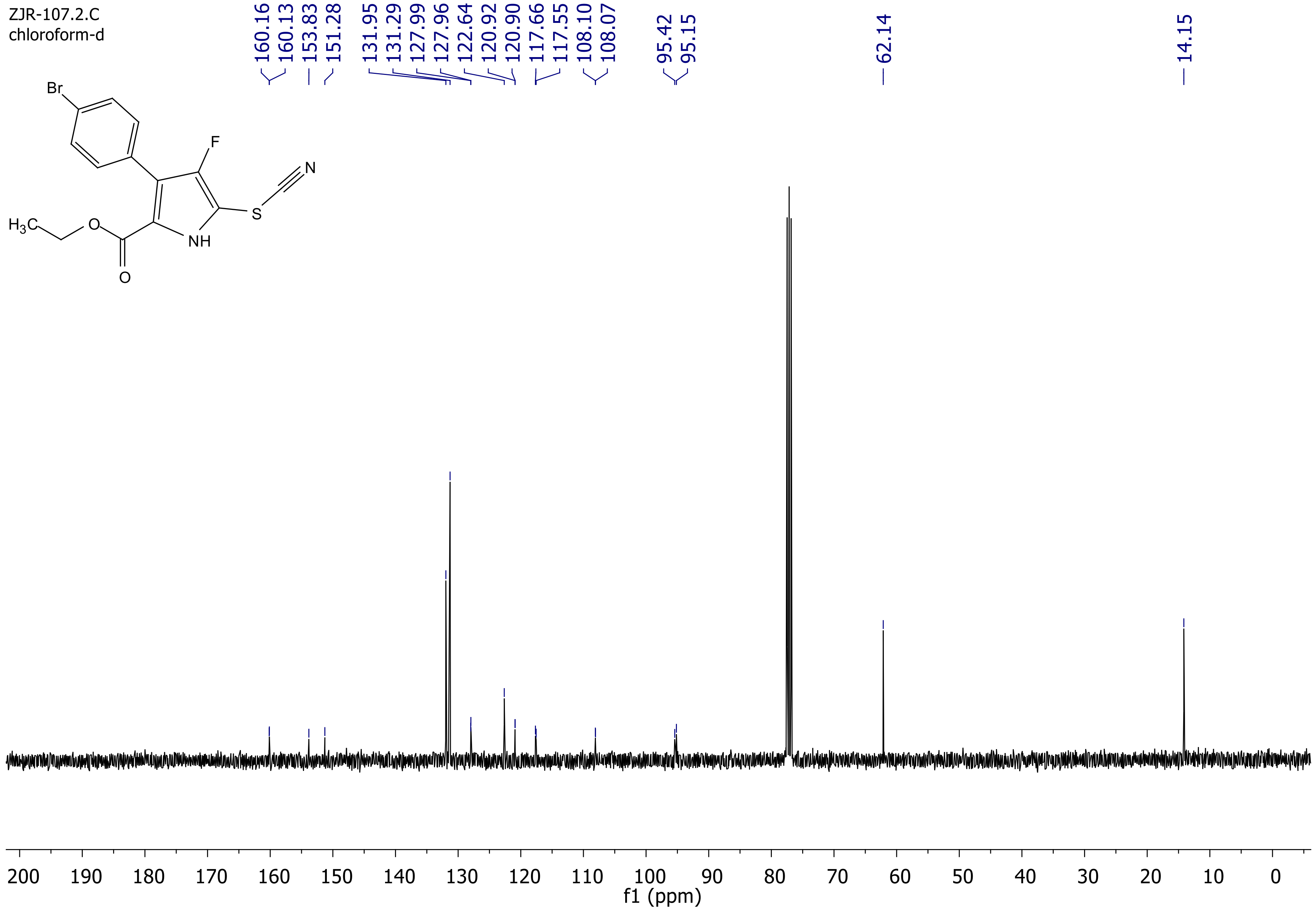

^{13}C NMR spectrum of ethyl 3-(4-bromophenyl)-4-fluoro-5-thiocyanato-1H-pyrrole-2-carboxylate (2**c**) in $CDCl_3$ at 100 MHz

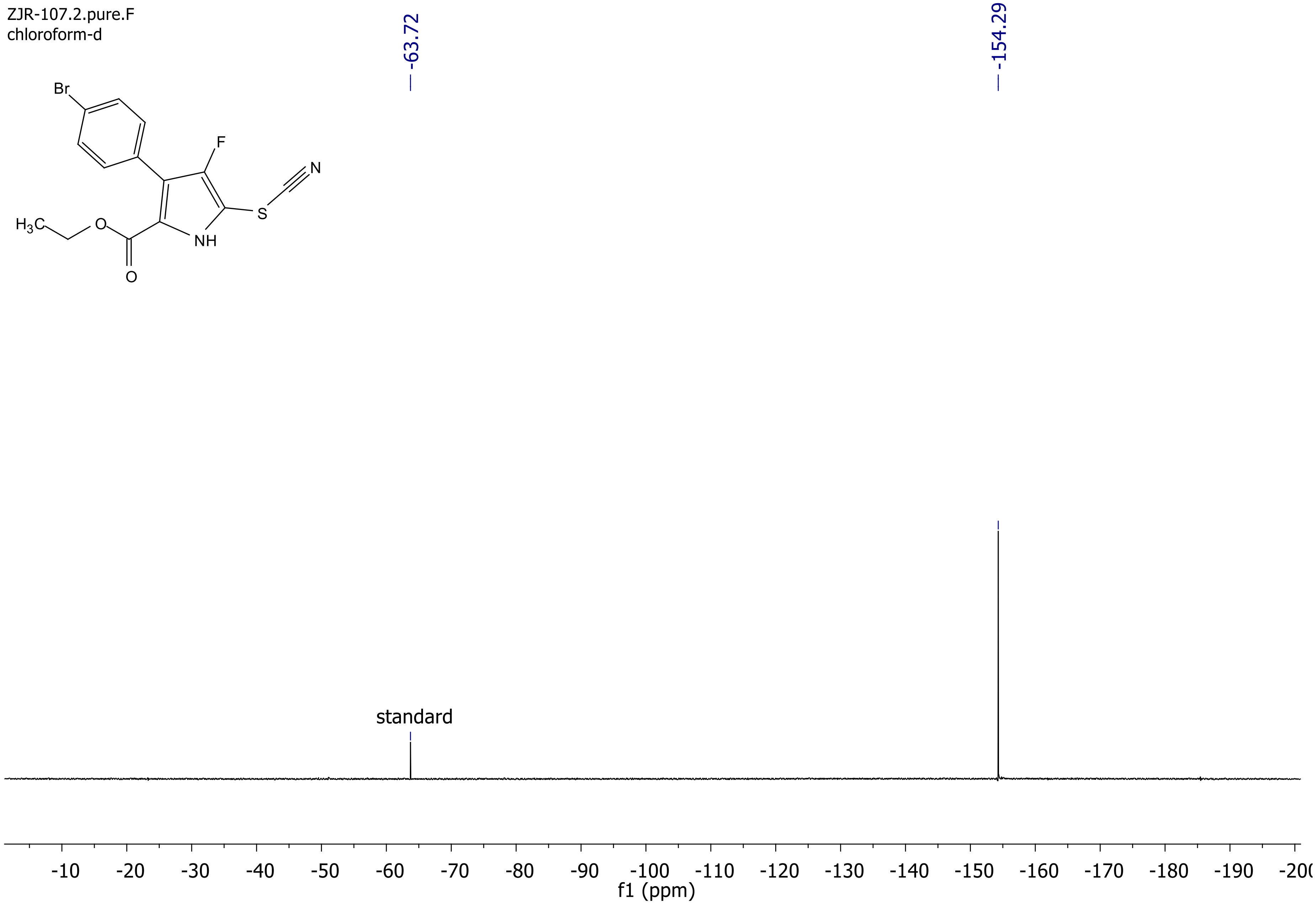

^{19}F NMR spectrum of ethyl 3-(4-bromophenyl)-4-fluoro-5-thiocyanato-1H-pyrrole-2-carboxylate (2**c**) in $CDCl_3$ at 376 MHz

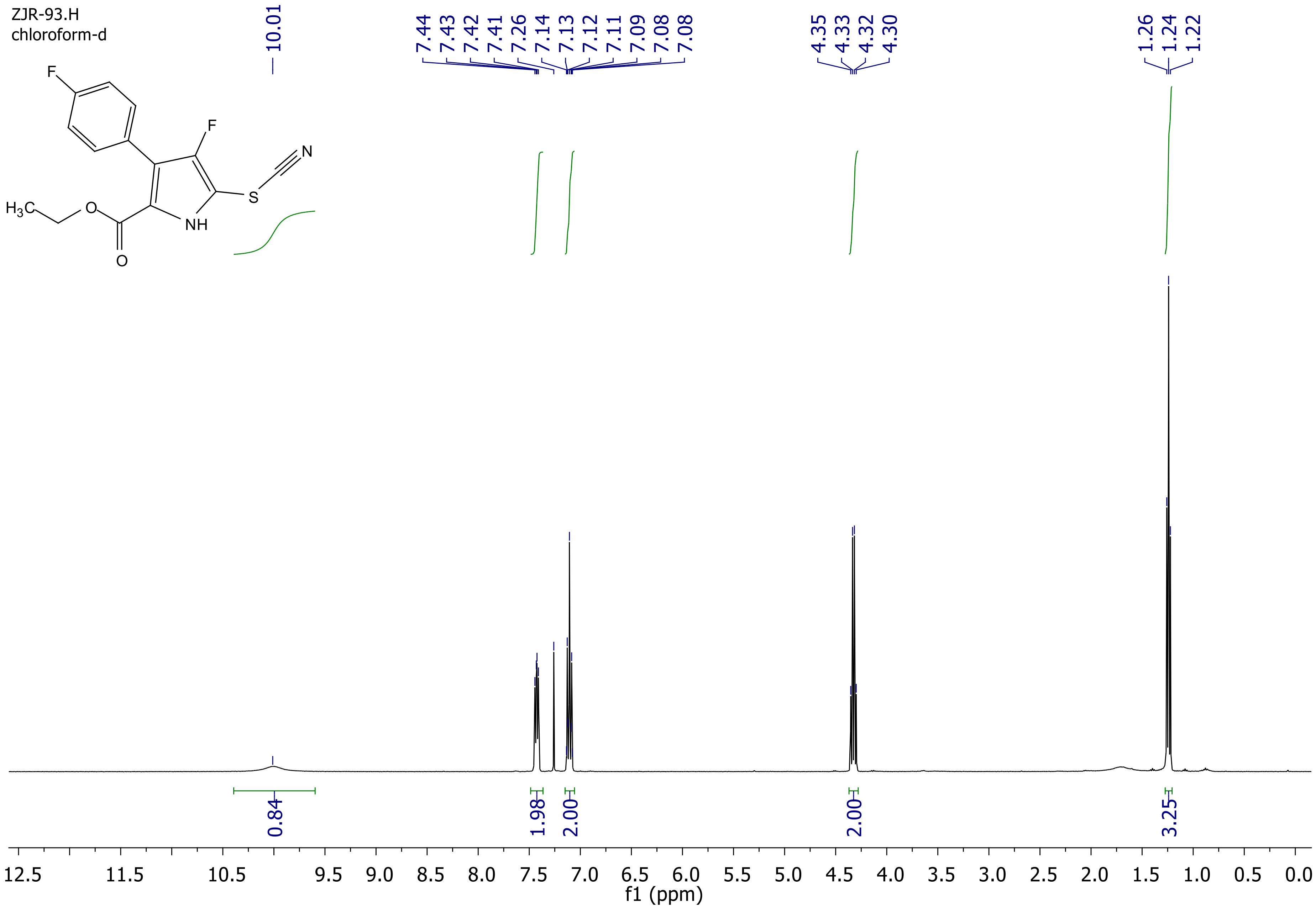

^{1}H NMR spectrum of ethyl 4-fluoro-3-(4-fluorophenyl)-5-thiocyanato-1H-pyrrole-2-carboxylate (**2d**) in $CDCl_3$ at 400 MHz

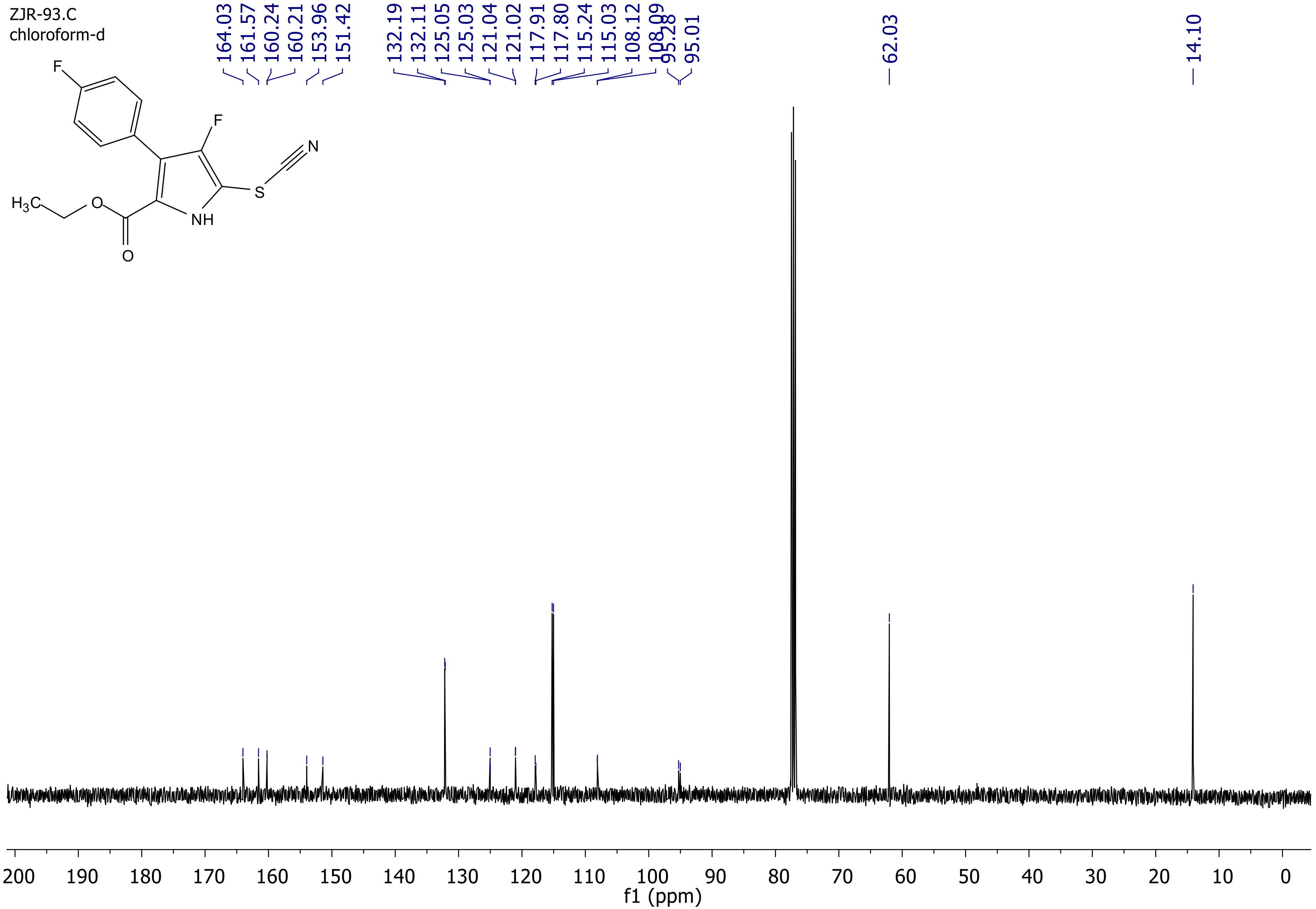

^{13}C NMR spectrum of ethyl 4-fluoro-3-(4-fluorophenyl)-5-thiocyanato-1H-pyrrole-2-carboxylate (**2d**) in $CDCl_3$ at 100 MHz

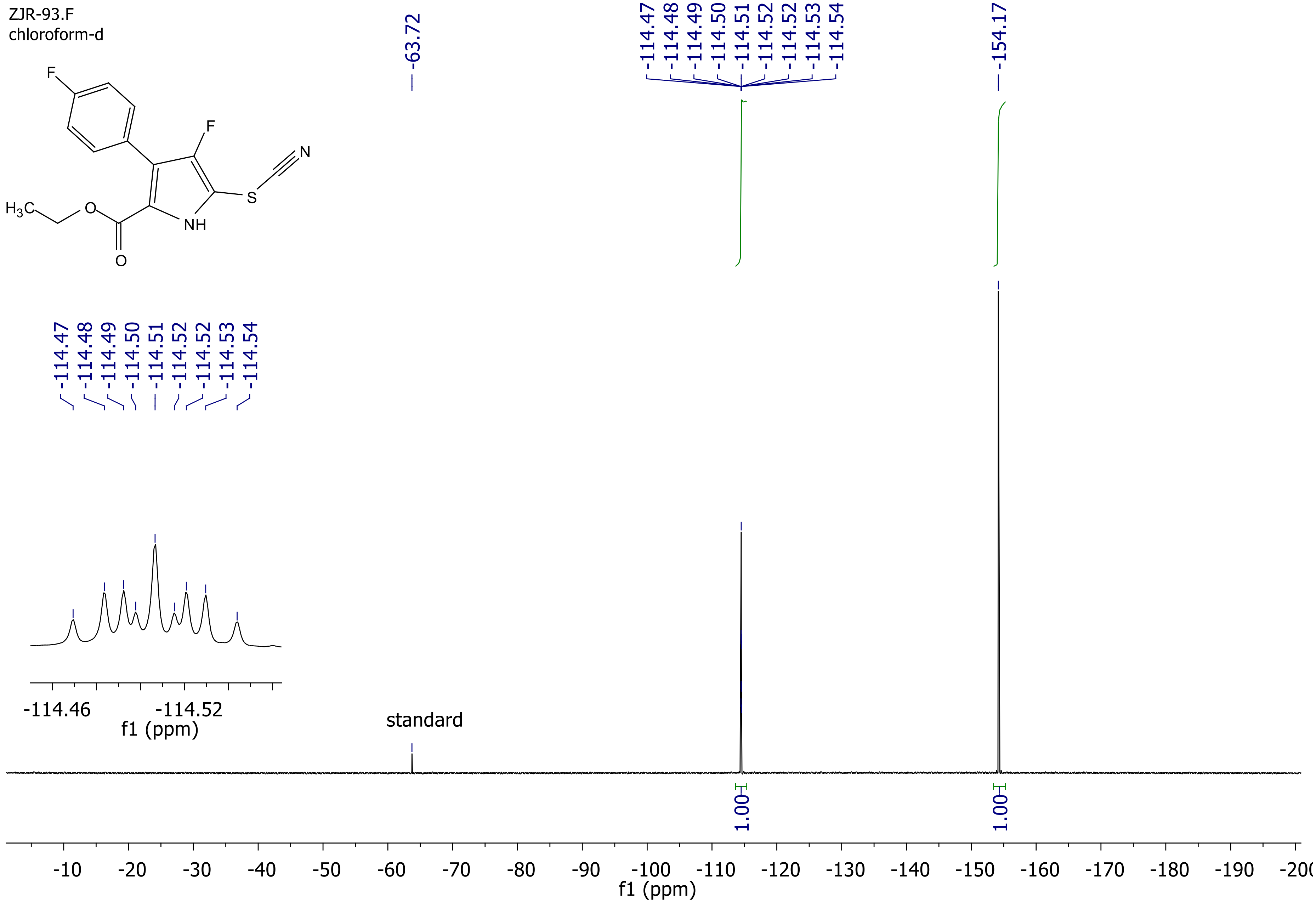

^{19}F NMR spectrum of ethyl 4-fluoro-3-(4-fluorophenyl)-5-thiocyanato-1H-pyrrole-2-carboxylate (**2d**) in $CDCl_3$ at 376 MHz

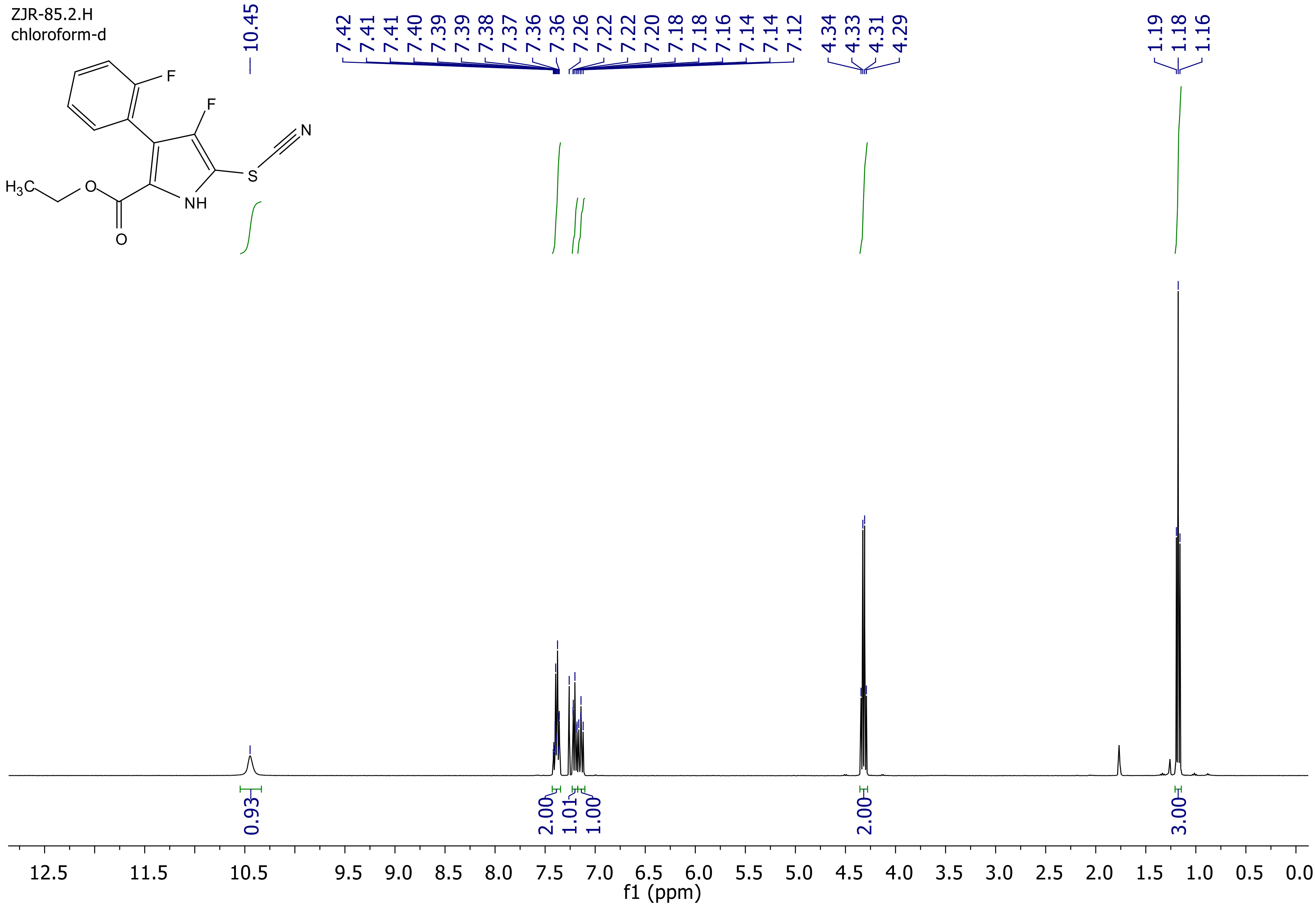

^{1}H NMR spectrum of ethyl 4-fluoro-3-(2-fluorophenyl)-5-thiocyanato-1H-pyrrole-2-carboxylate (**2e**) in $CDCl_3$ at 400 MHz

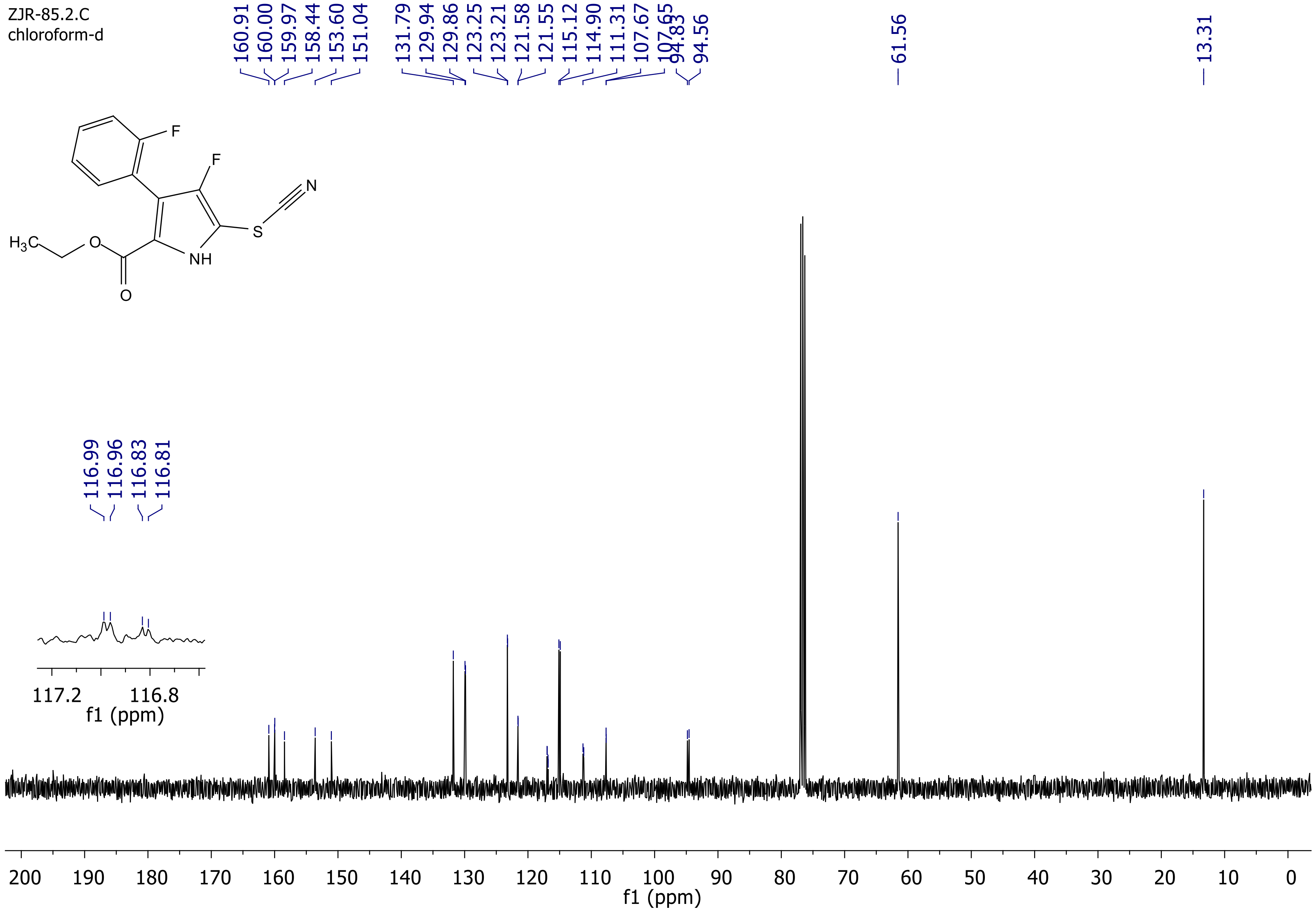

^{13}C NMR spectrum of ethyl 4-fluoro-3-(2-fluorophenyl)-5-thiocyanato-1H-pyrrole-2-carboxylate (**2e**) in $CDCl_3$ at 100 MHz

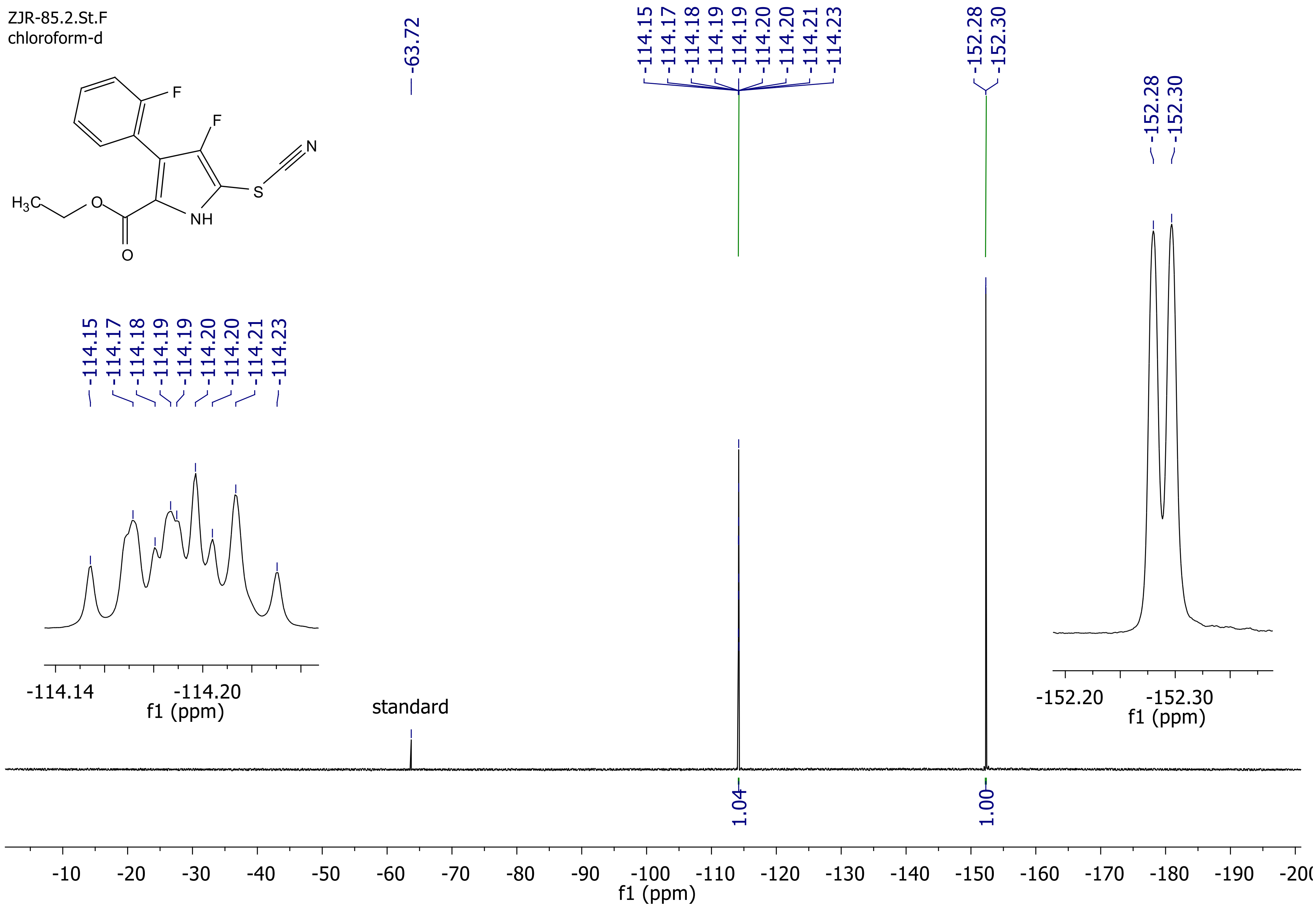

^{19}F NMR spectrum of ethyl 4-fluoro-3-(2-fluorophenyl)-5-thiocyanato-1H-pyrrole-2-carboxylate (**2e**) in $CDCl_3$ at 376 MHz

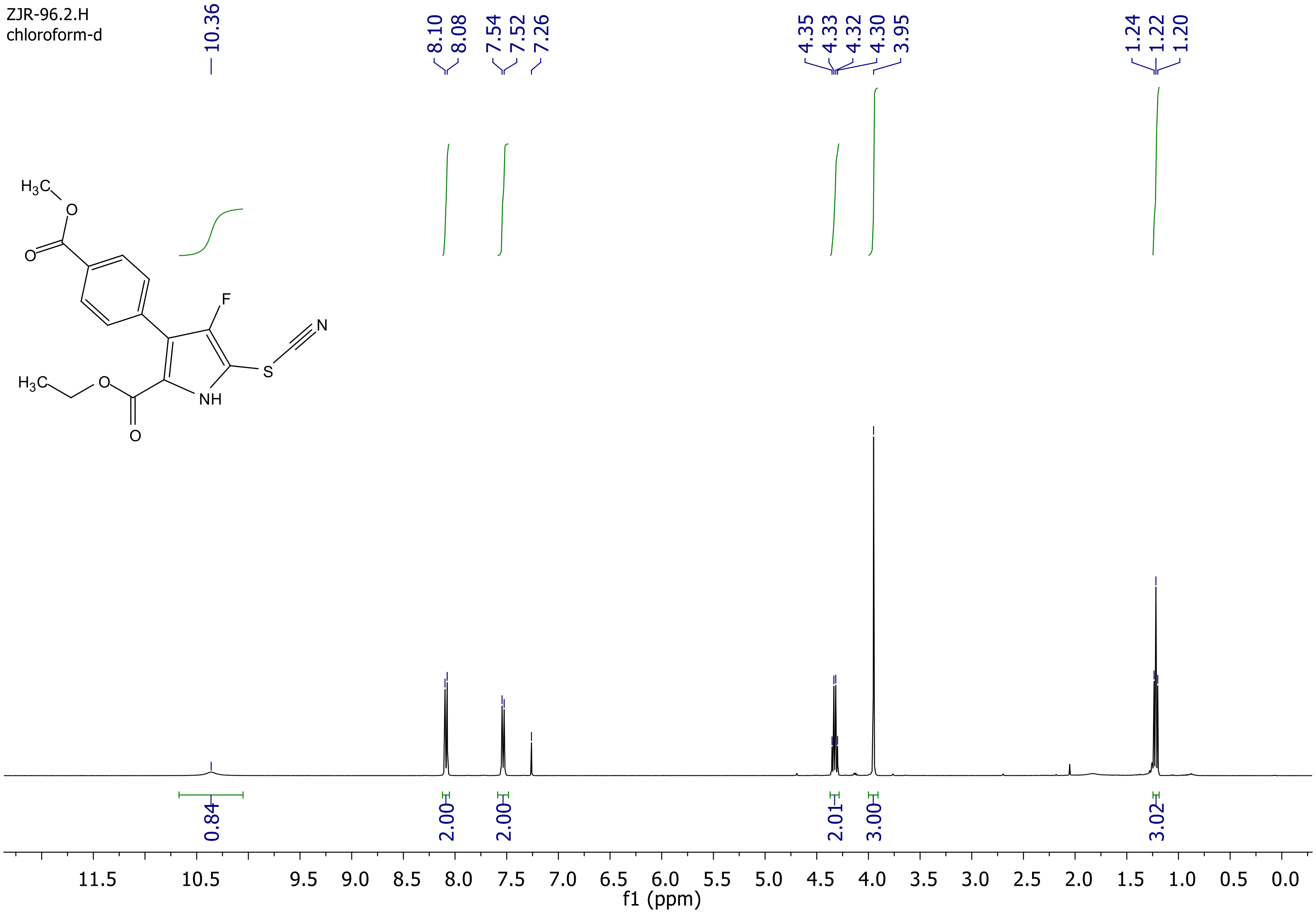

^{1}H NMR spectrum of ethyl 4-fluoro-3-(4-(methoxycarbonyl)phenyl)-5-thiocyanato-1H-pyrrole-2-carboxylate (**2f**) in $CDCl_3$ at 400 MHz

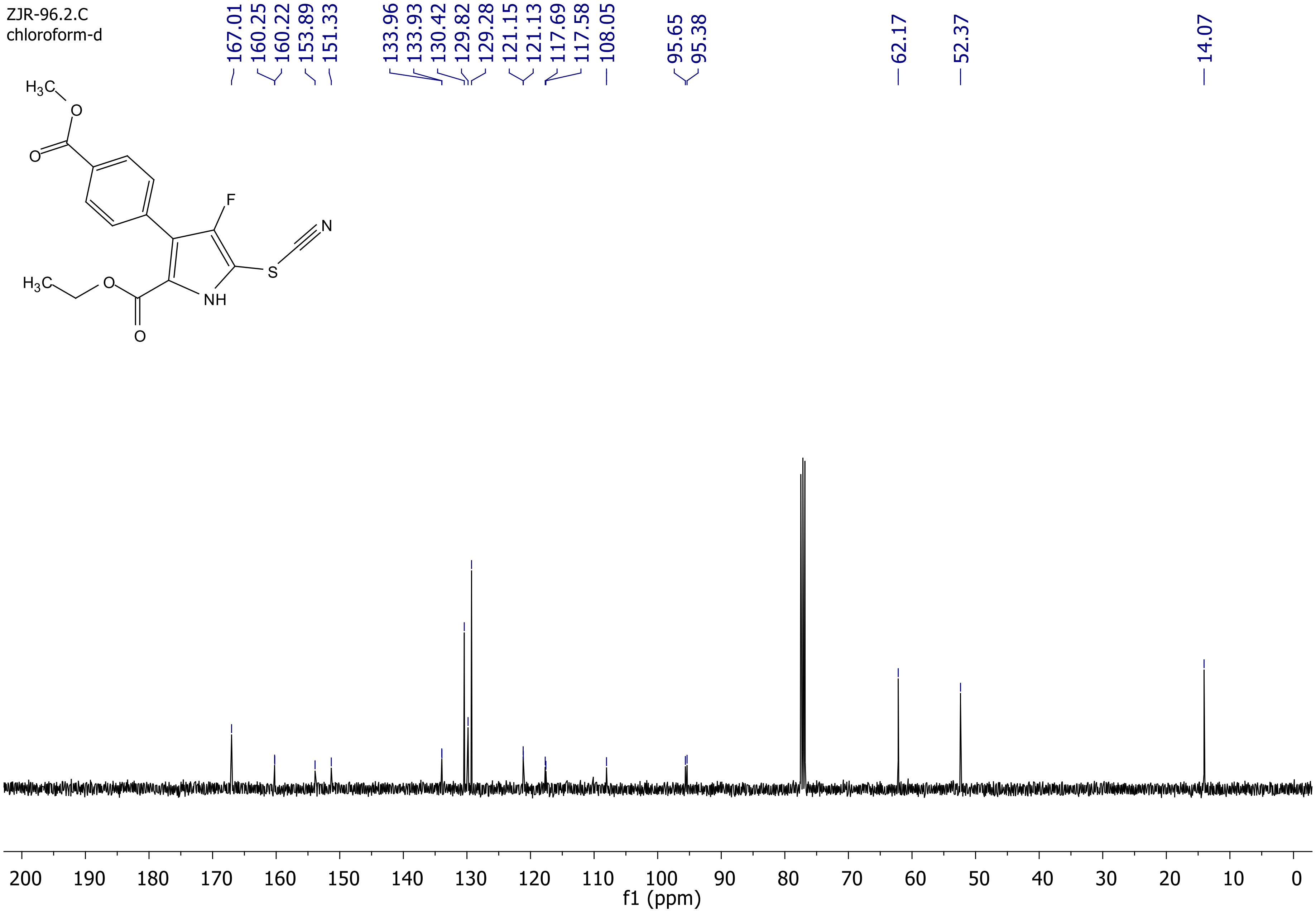

^{13}C NMR spectrum of ethyl 4-fluoro-3-(4-(methoxycarbonyl)phenyl)-5-thiocyanato-1H-pyrrole-2-carboxylate (**2f**) in $CDCl_3$ at 100 MHz

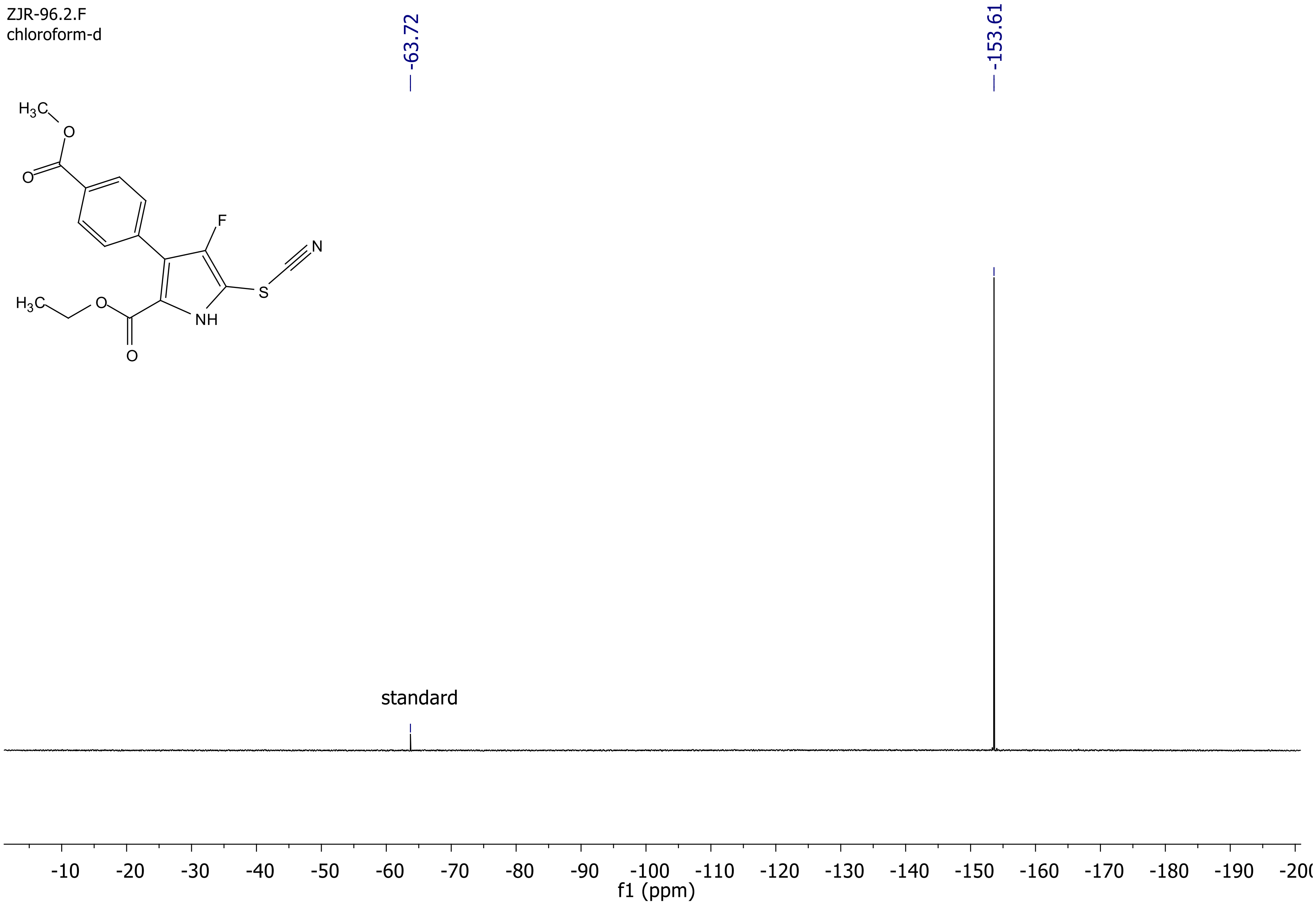

^{19}F NMR spectrum of ethyl 4-fluoro-3-(4-(methoxycarbonyl)phenyl)-5-thiocyanato-1H-pyrrole-2-carboxylate (**2f**) in $CDCl_3$ at 376 MHz

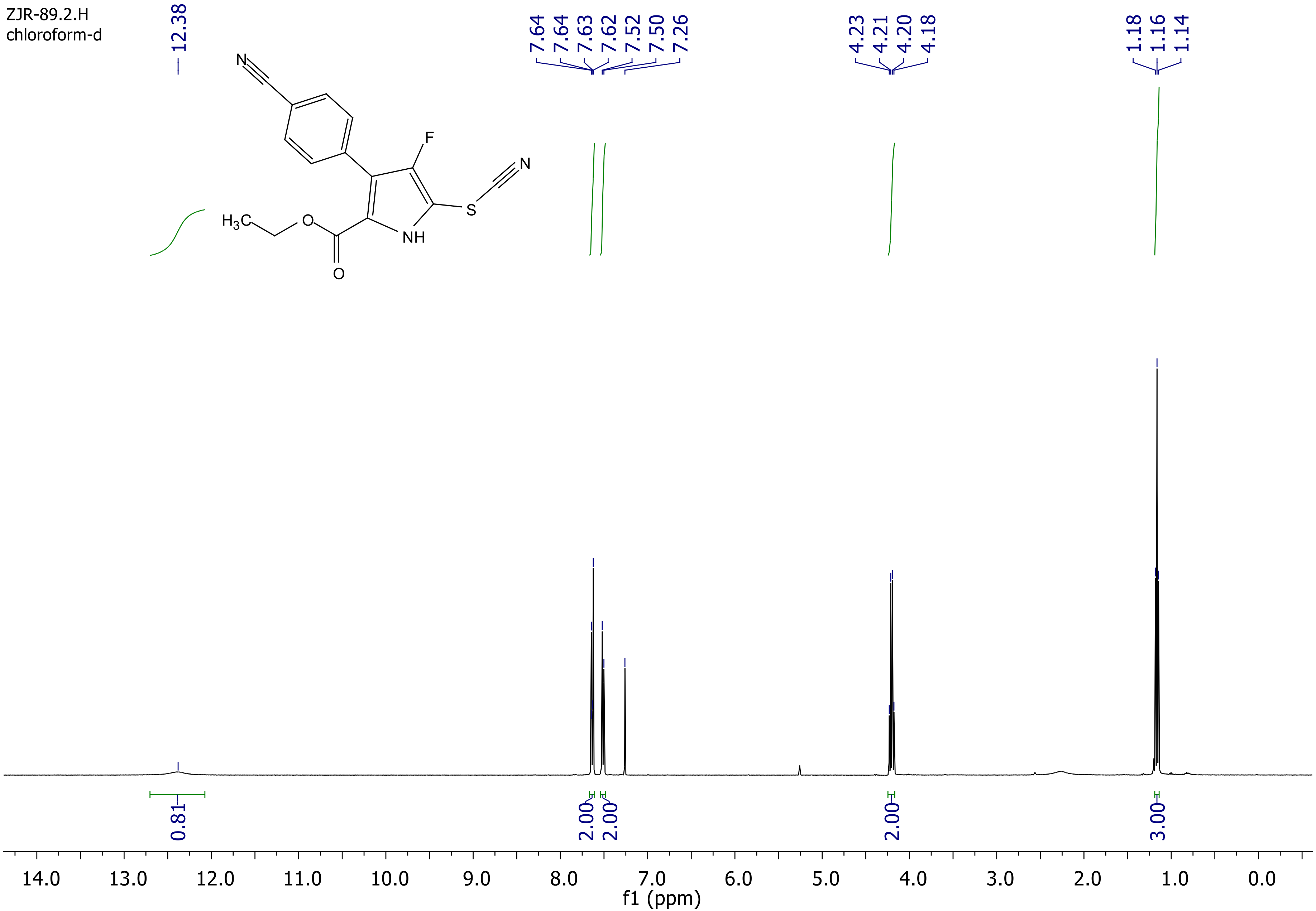

^{1}H NMR spectrum of ethyl 3-(4-cyanophenyl)-4-fluoro-5-thiocyanato-1H-pyrrole-2-carboxylate (**2g**) in $CDCl_3$ at 400 MHz

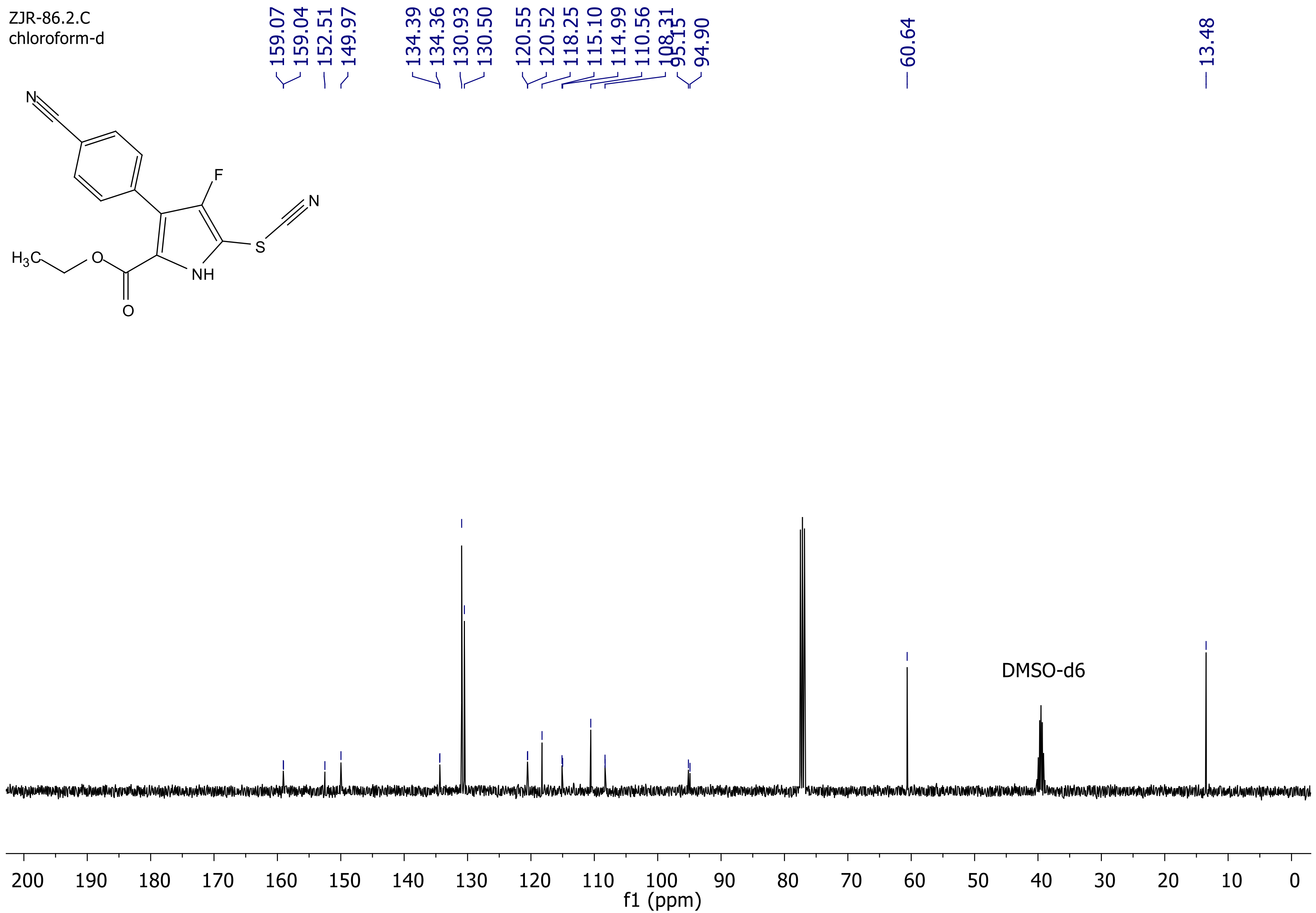

^{13}C NMR spectrum of ethyl 3-(4-cyanophenyl)-4-fluoro-5-thiocyanato-1H-pyrrole-2-carboxylate (**2g**) in $CDCl_3$ at 100 MHz

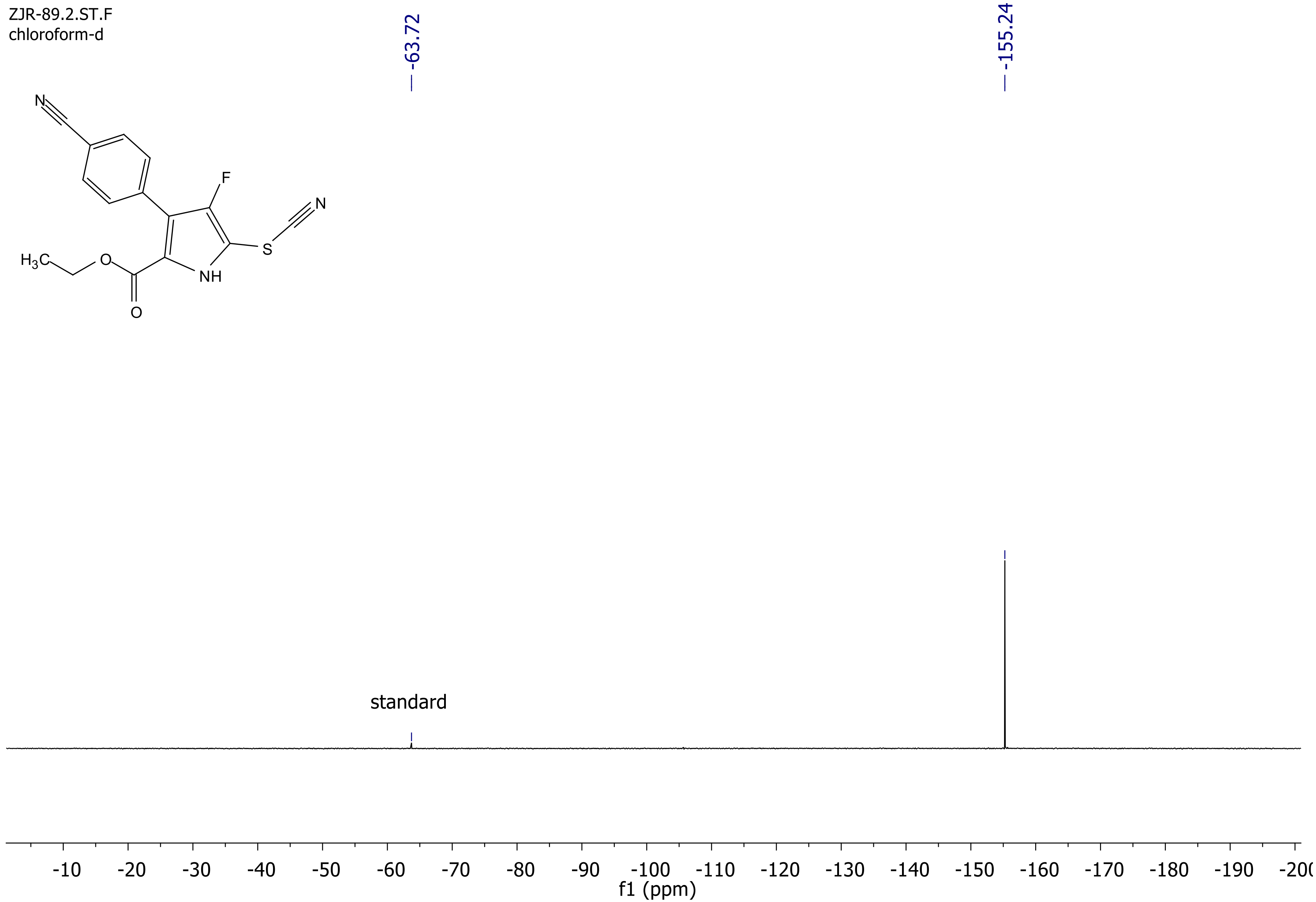

^{19}F NMR spectrum of ethyl 3-(4-cyanophenyl)-4-fluoro-5-thiocyanato-1H-pyrrole-2-carboxylate (**2g**) in $CDCl_3$ at 376 MHz

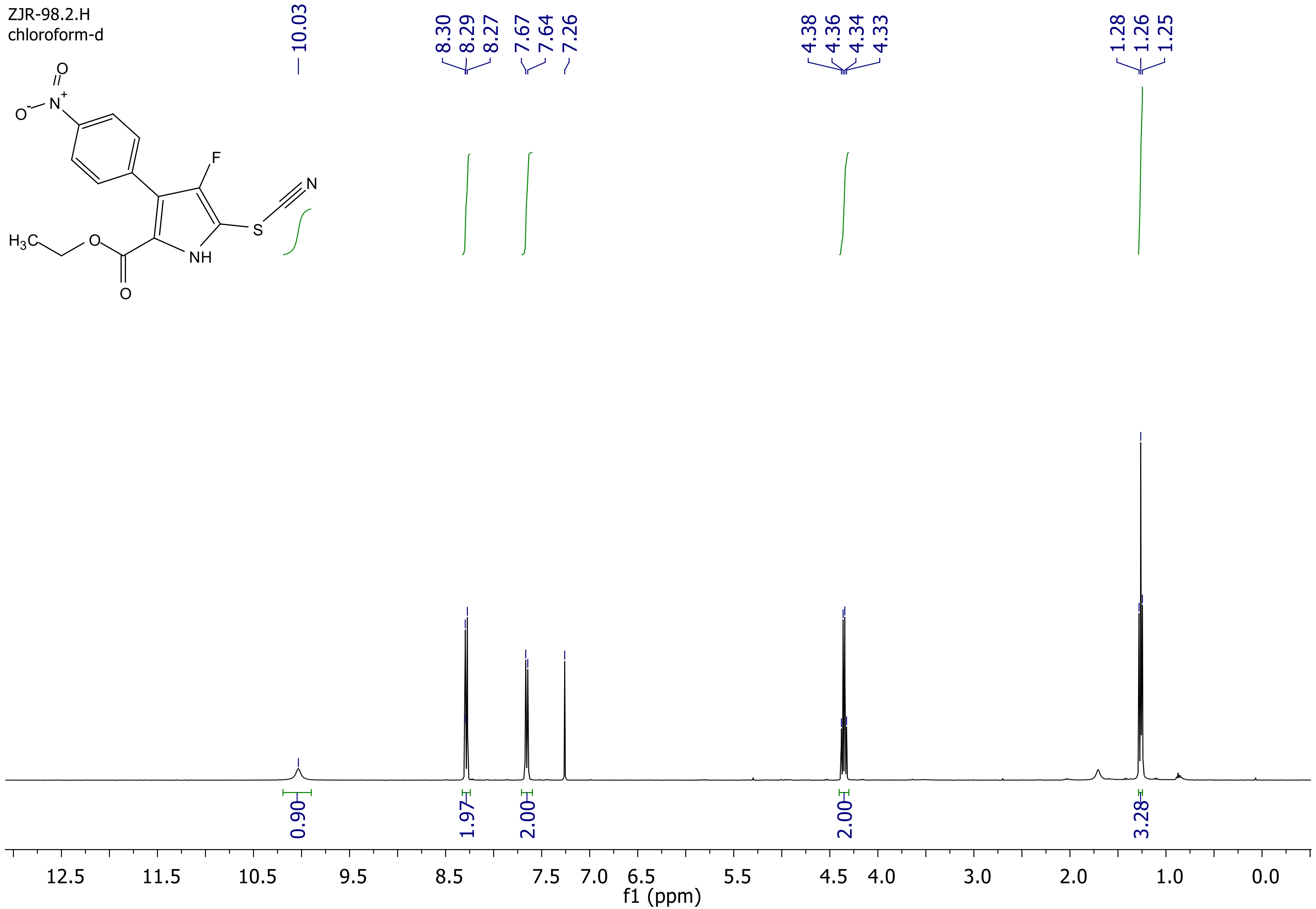

^{1}H NMR spectrum of ethyl 4-fluoro-3-(4-nitrophenyl)-5-thiocyanato-1H-pyrrole-2-carboxylate (**2h**) in $CDCl_3$ at 400 MHz

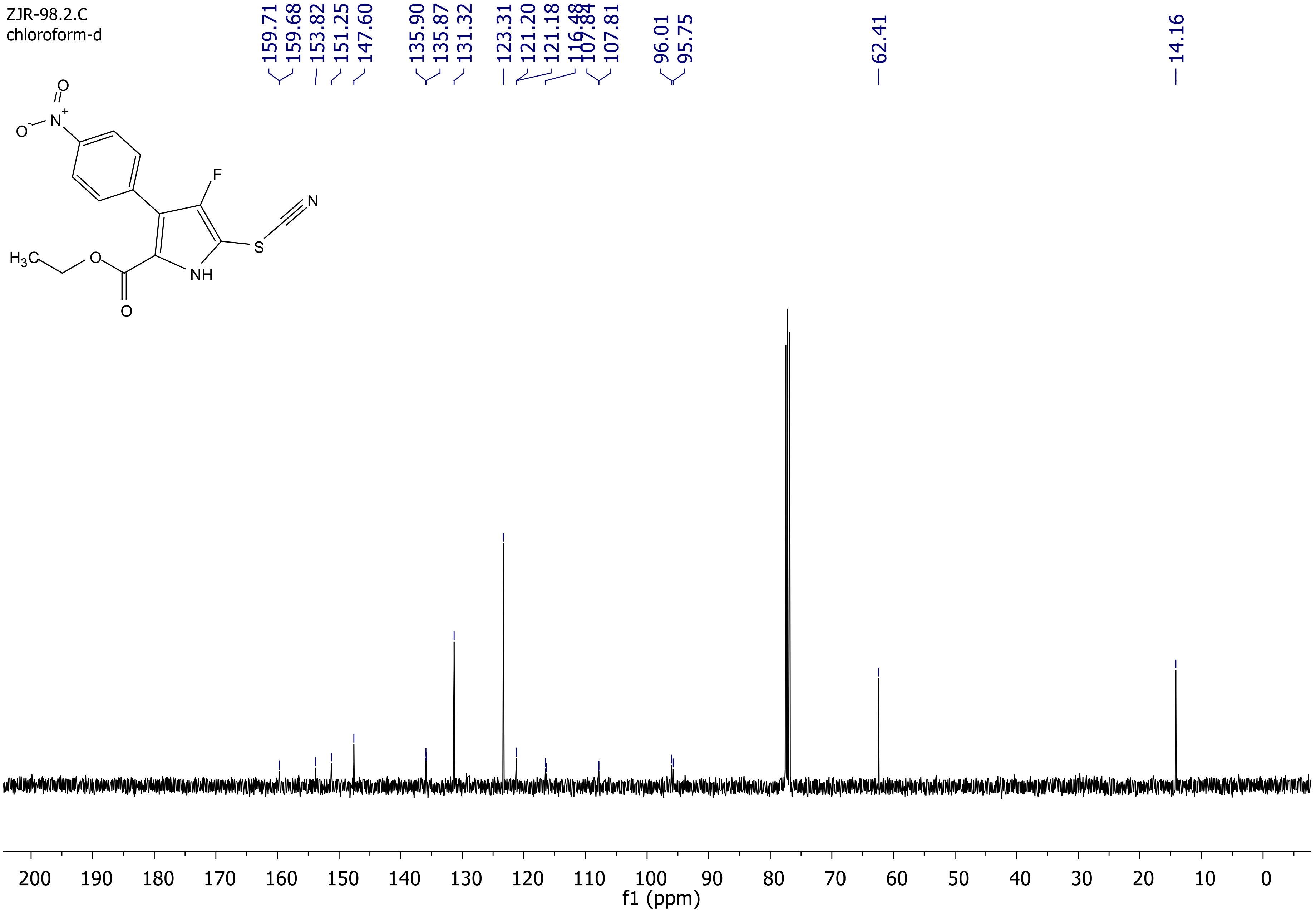

^{13}C NMR spectrum of ethyl 4-fluoro-3-(4-nitrophenyl)-5-thiocyanato-1H-pyrrole-2-carboxylate (**2h**) in $CDCl_3$ at 100 MHz

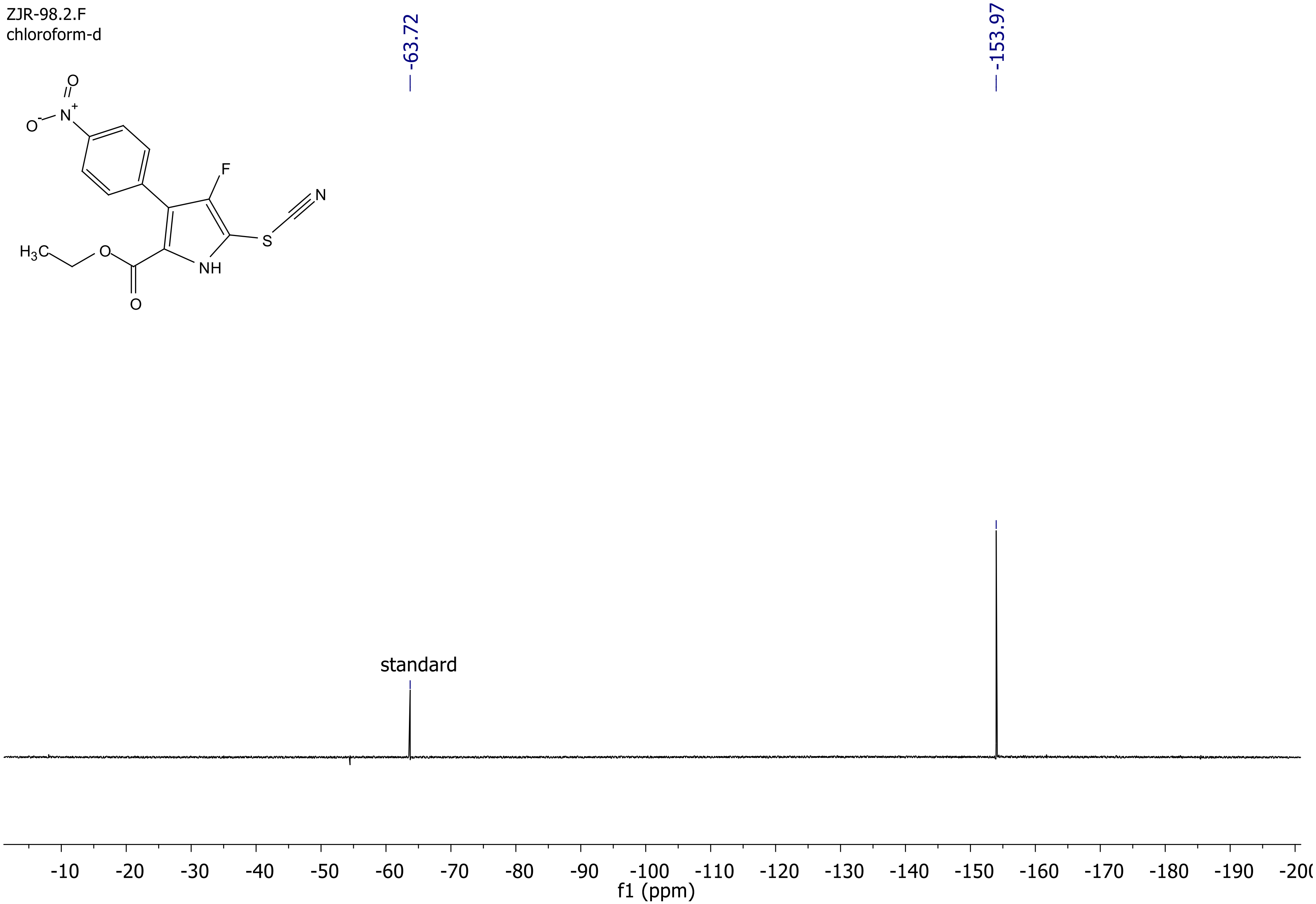

^{19}F NMR spectrum of ethyl 4-fluoro-3-(4-nitrophenyl)-5-thiocyanato-1H-pyrrole-2-carboxylate (**2h**) in $CDCl_3$ at 376 MHz

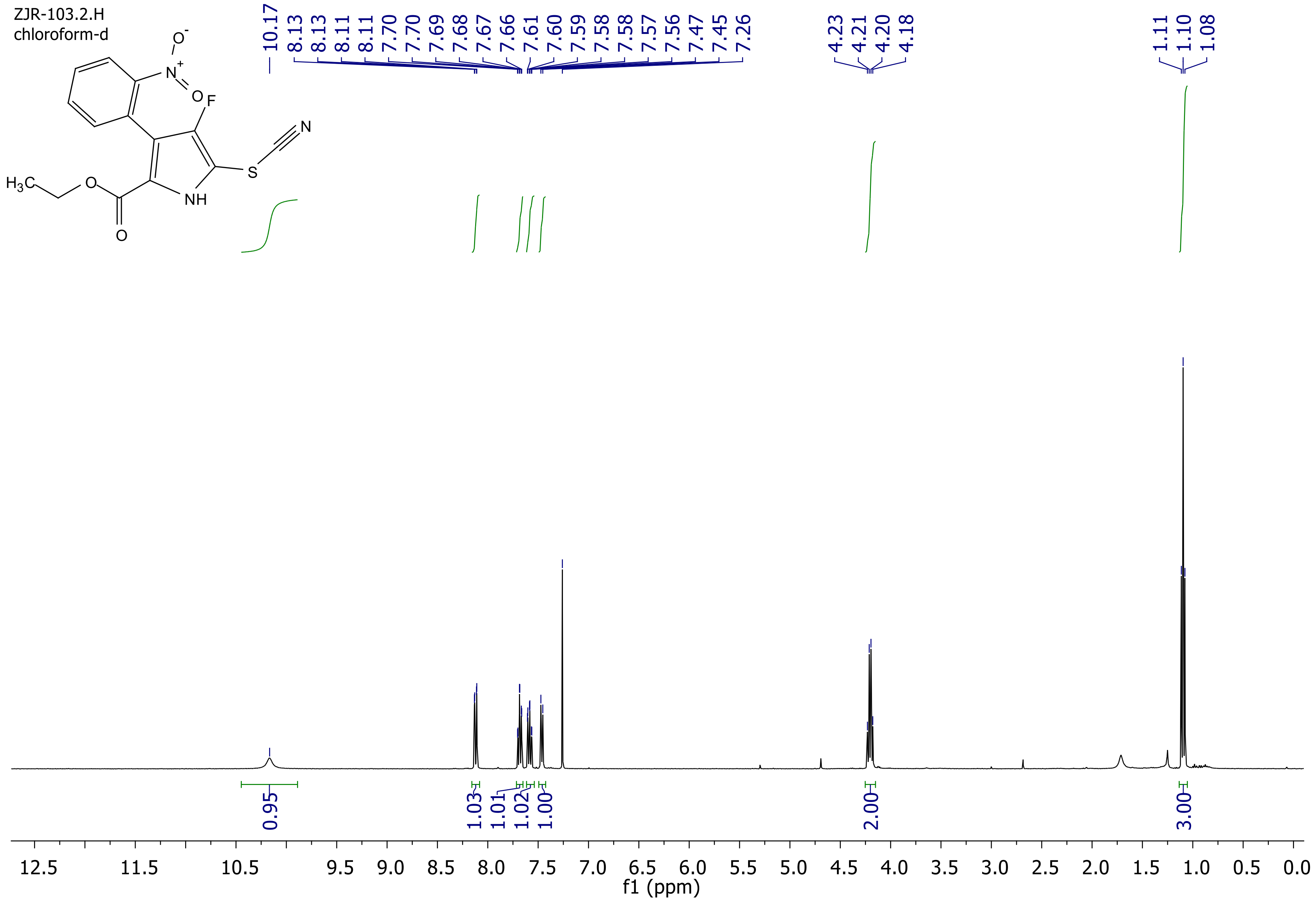

^1H NMR spectrum of ethyl 4-fluoro-3-(2-nitrophenyl)-5-thiocyanato-1H-pyrrole-2-carboxylate (**2i**) in $CDCl_3$ at 400 MHz

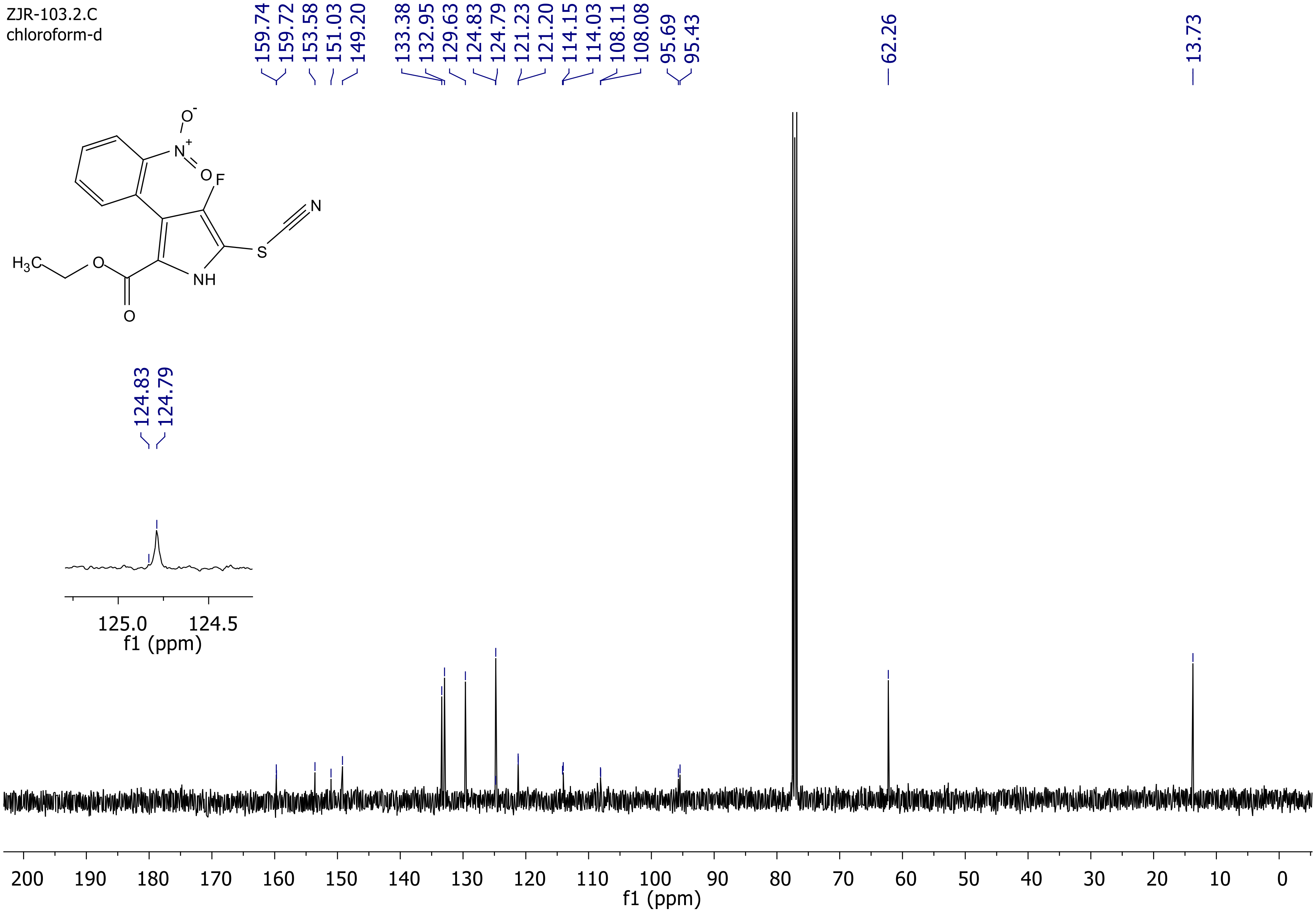

^{13}C NMR spectrum of ethyl 4-fluoro-3-(2-nitrophenyl)-5-thiocyanato-1H-pyrrole-2-carboxylate (**2i**) in $CDCl_3$ at 100 MHz

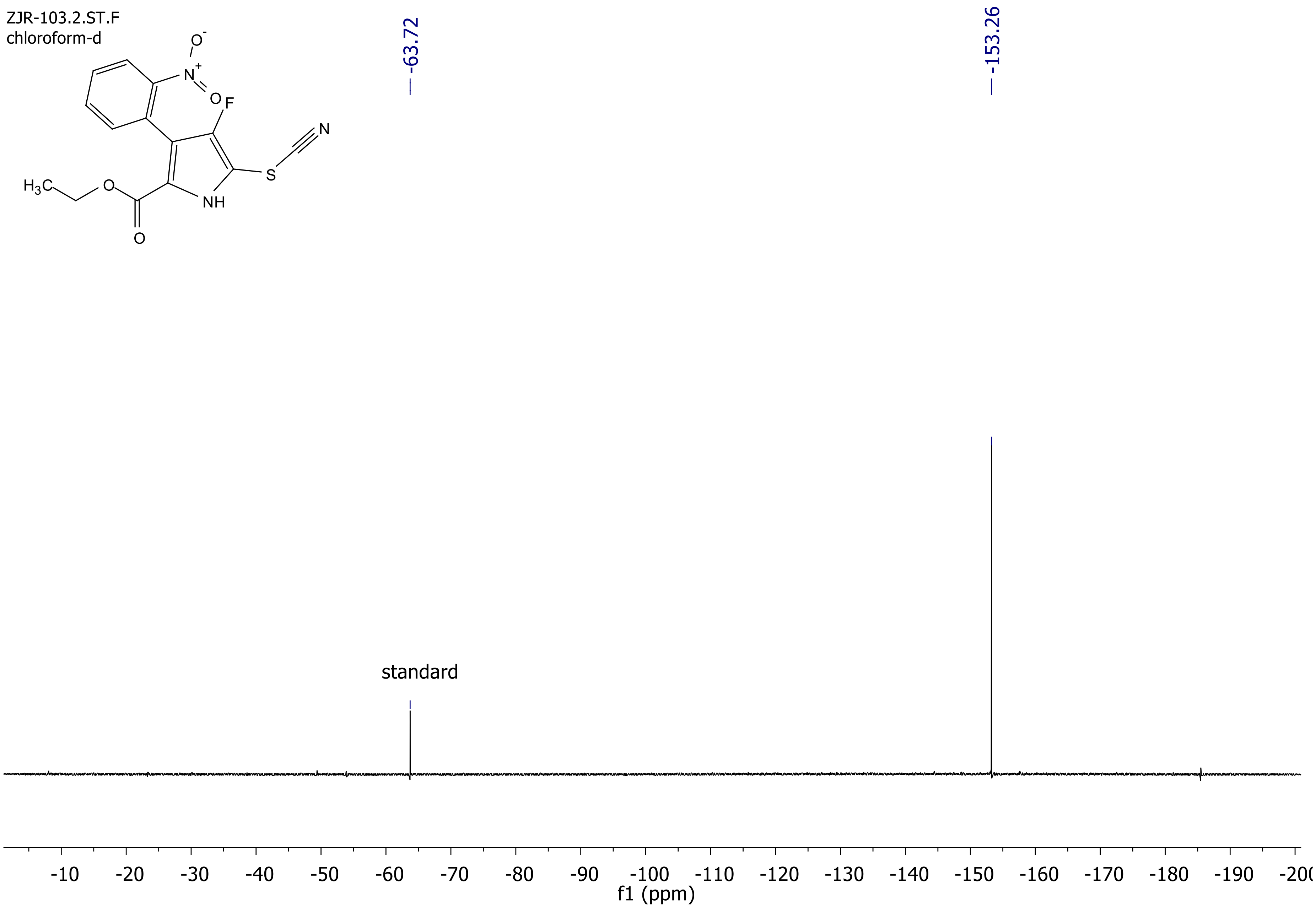

^{19}F NMR spectrum of ethyl 4-fluoro-3-(2-nitrophenyl)-5-thiocyanato-1H-pyrrole-2-carboxylate (**2i**) in $CDCl_3$ at 376 MHz

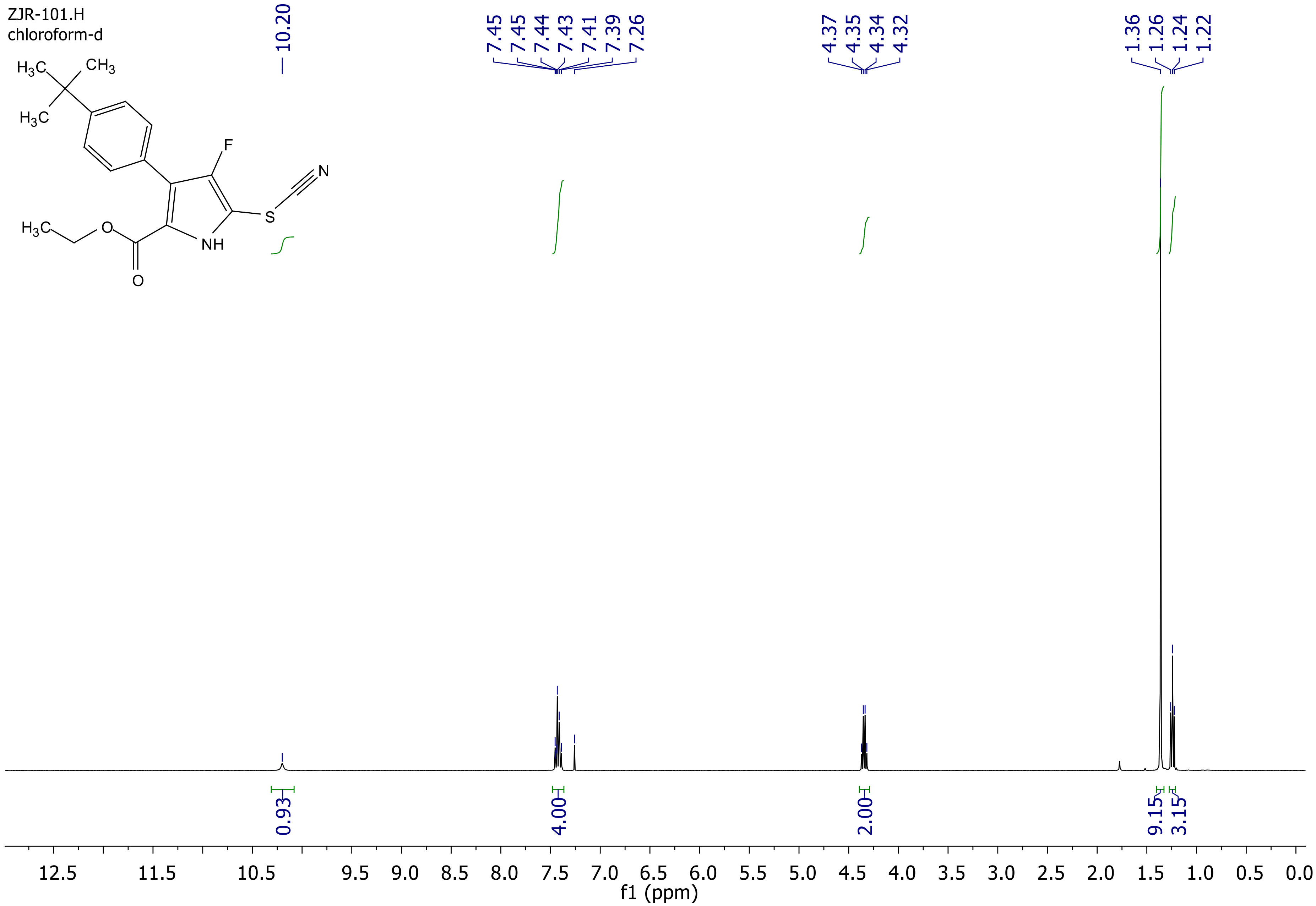

^{1}H NMR spectrum of ethyl 3-(4-(tert-butyl)phenyl)-4-fluoro-5-thiocyanato-1H-pyrrole-2-carboxylate (**2j**) in $CDCl_3$ at 400 MHz

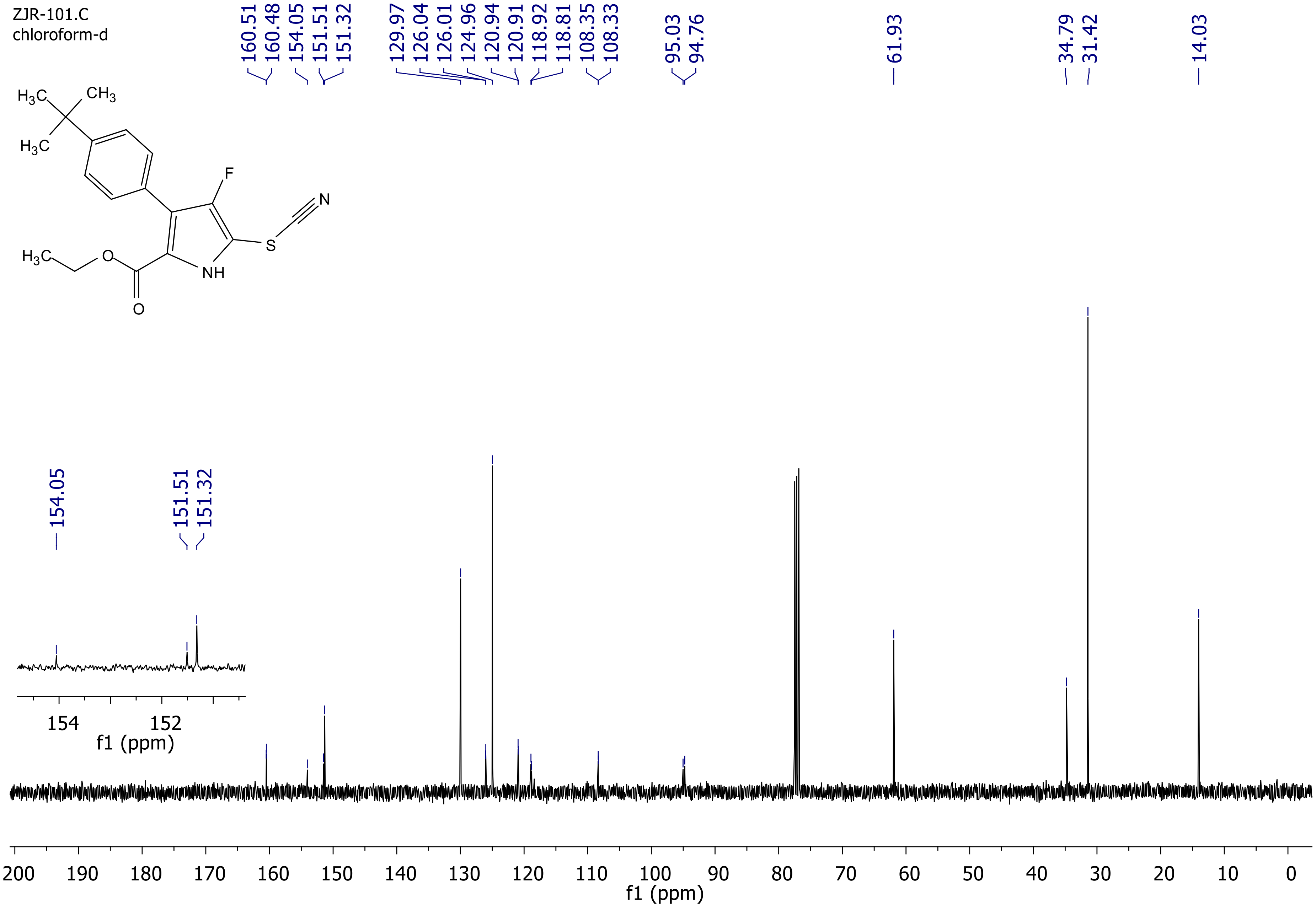

^{13}C NMR spectrum of ethyl 3-(4-(tert-butyl)phenyl)-4-fluoro-5-thiocyanato-1H-pyrrole-2-carboxylate (**2j**) in $CDCl_3$ at 100 MHz

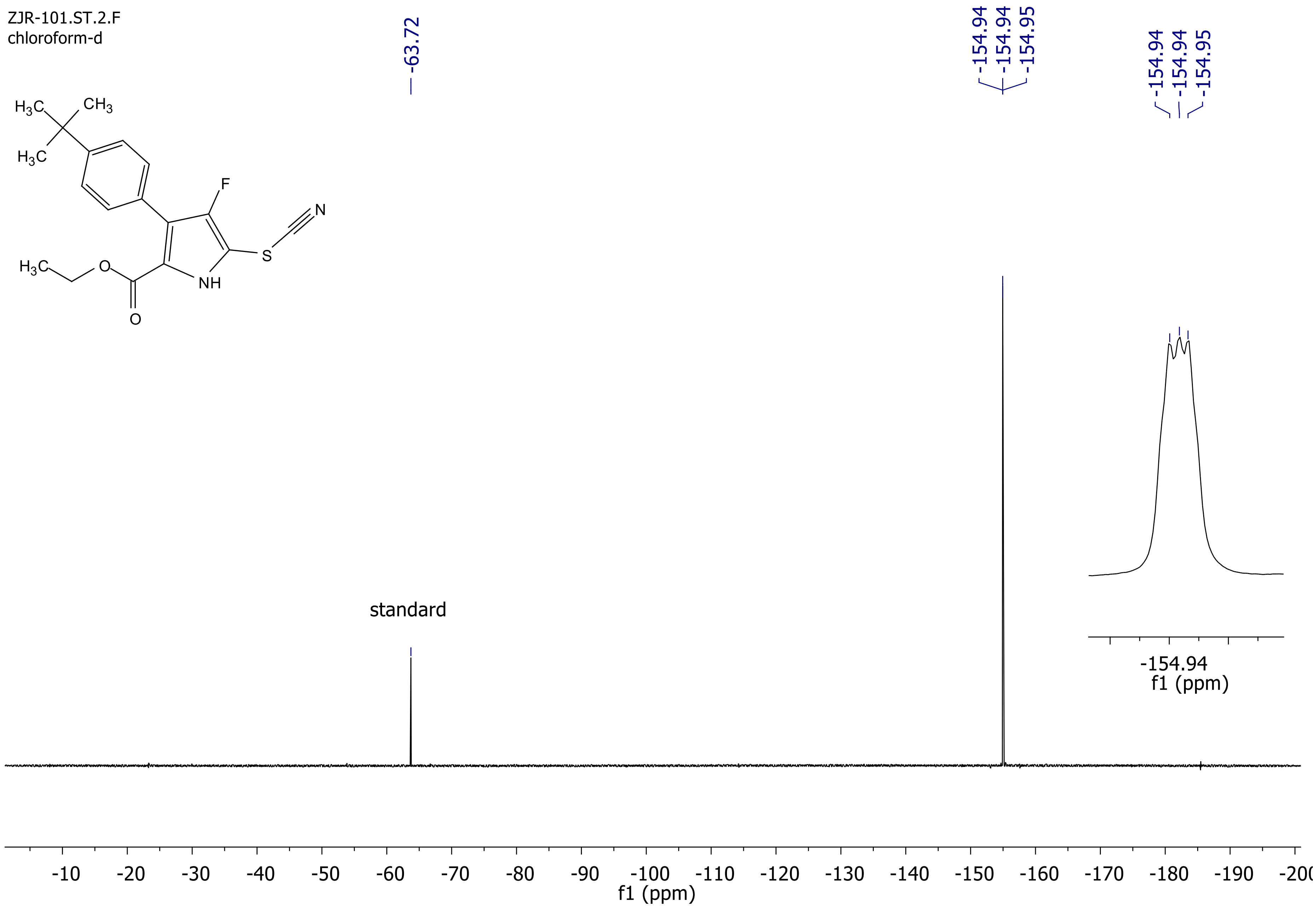

^{19}F NMR spectrum of ethyl 3-(4-(tert-butyl)phenyl)-4-fluoro-5-thiocyanato-1H-pyrrole-2-carboxylate (**2j**) in $CDCl_3$ at 376 MHz

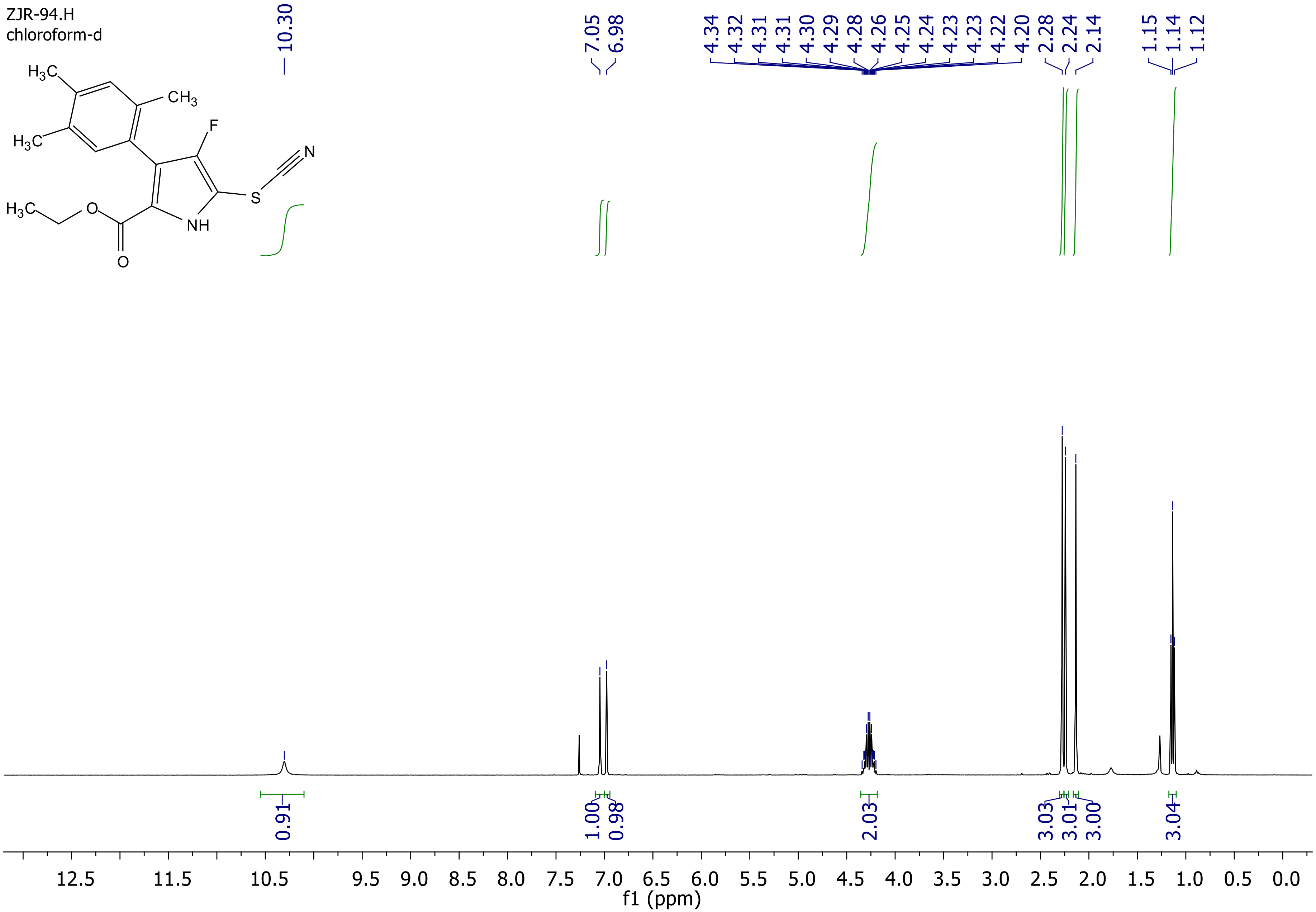

^{1}H NMR spectrum of ethyl 4-fluoro-5-thiocyanato-3-(2,4,5-trimethylphenyl)-1H-pyrrole-2-carboxylate (**2k**) in $CDCl_3$ at 400 MHz

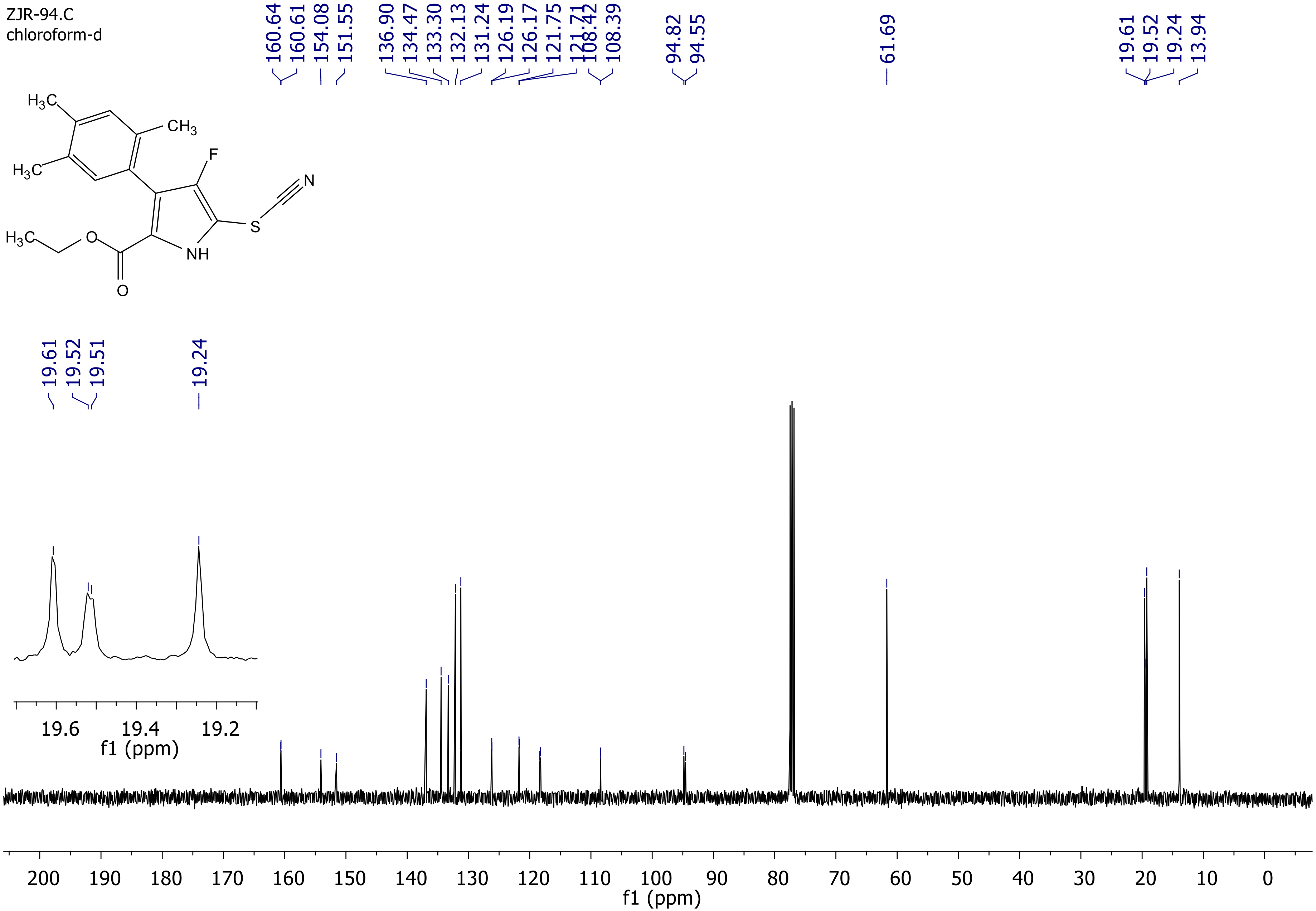

^{13}C NMR spectrum of ethyl 4-fluoro-5-thiocyanato-3-(2,4,5-trimethylphenyl)-1H-pyrrole-2-carboxylate (**2k**) in $CDCl_3$ at 100 MHz

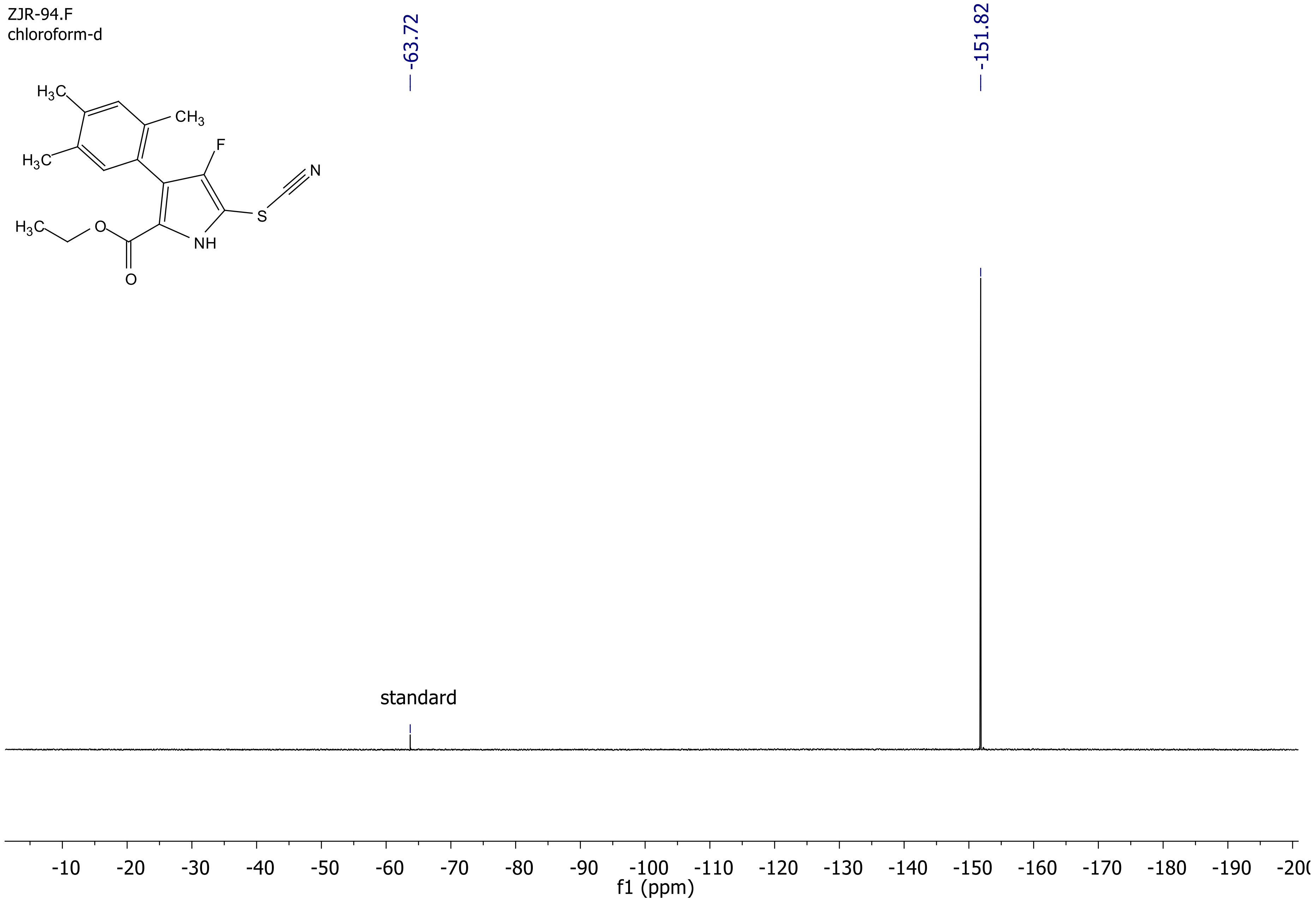

^{19}F NMR spectrum of ethyl 4-fluoro-5-thiocyanato-3-(2,4,5-trimethylphenyl)-1H-pyrrole-2-carboxylate (**2k**) in $CDCl_3$ at 376 MHz

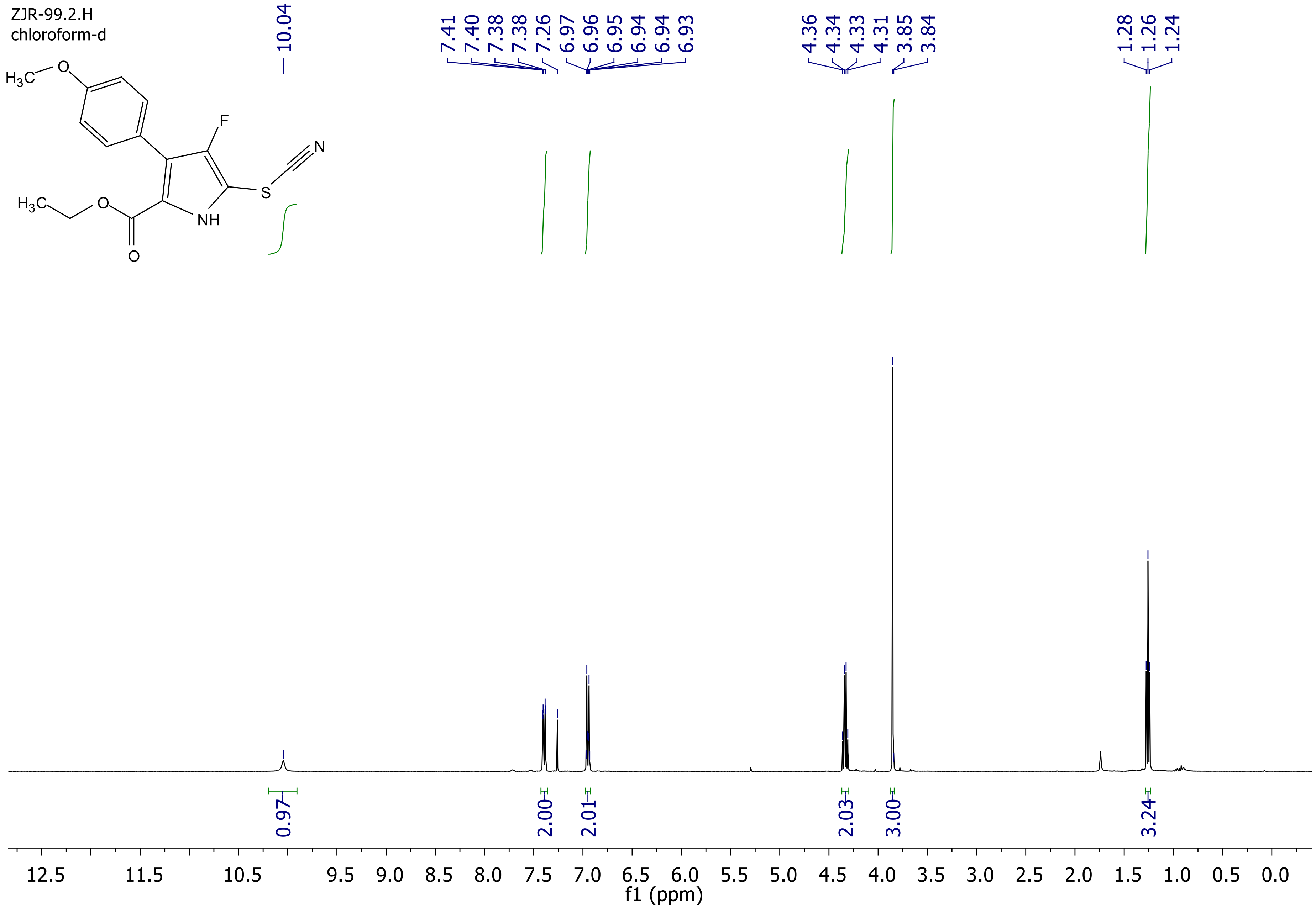

^{1}H NMR spectrum of ethyl 4-fluoro-3-(4-methoxyphenyl)-5-thiocyanato-1H-pyrrole-2-carboxylate (**2l**) in $CDCl_3$ at 400 MHz

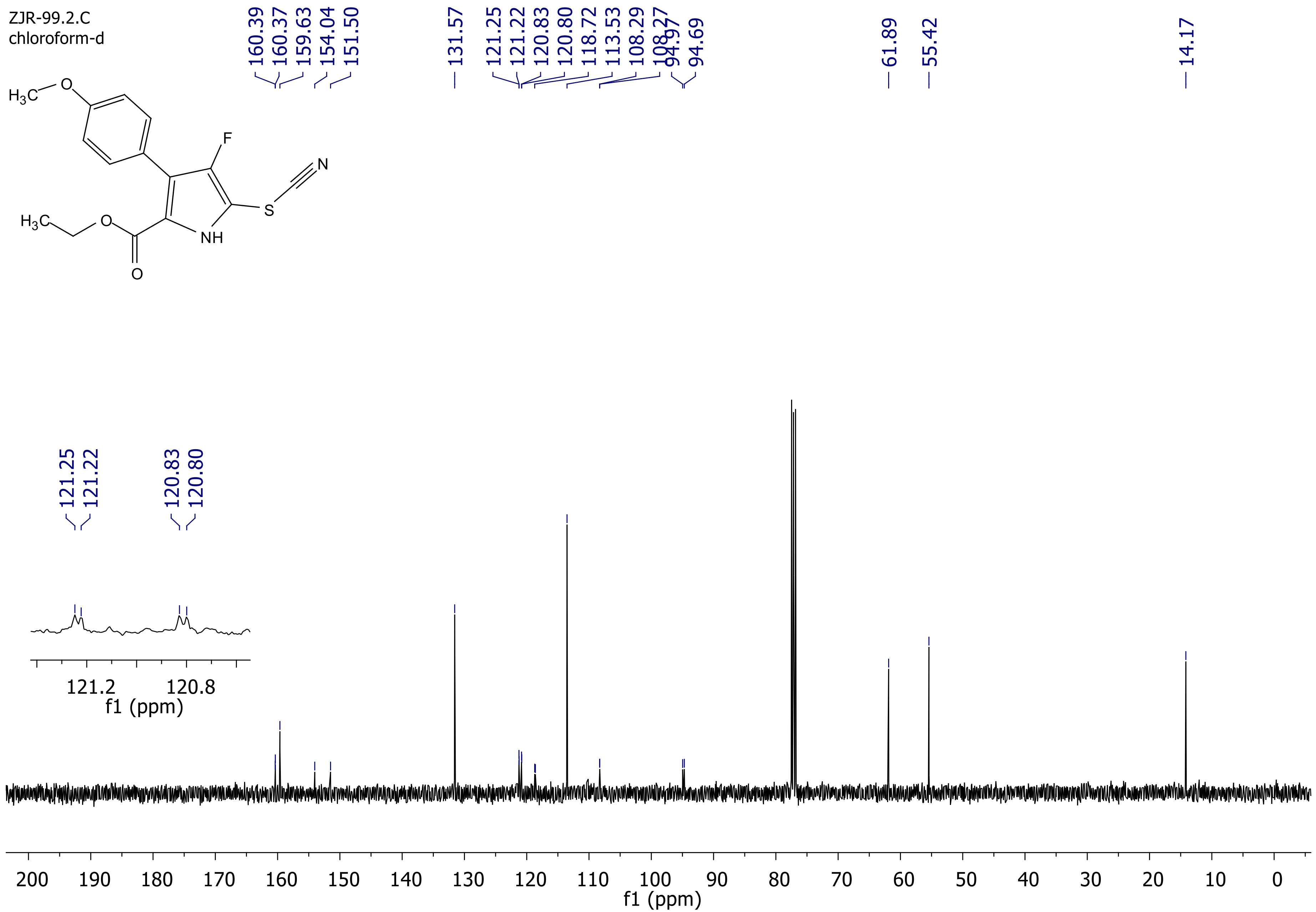

^{13}C NMR spectrum of ethyl 4-fluoro-3-(4-methoxyphenyl)-5-thiocyanato-1H-pyrrole-2-carboxylate (**2l**) in $CDCl_3$ at 100 MHz

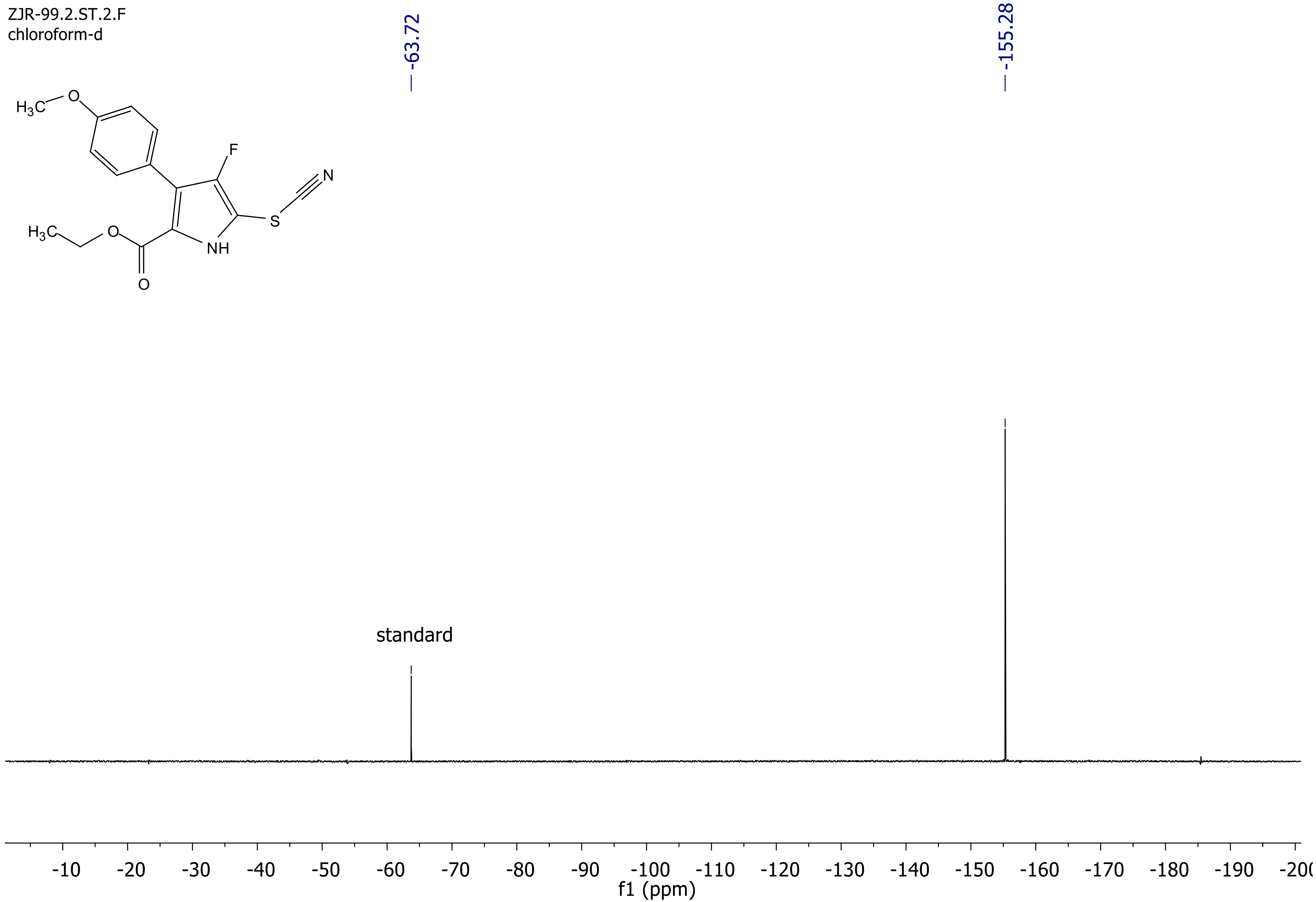

^{19}F NMR spectrum of ethyl 4-fluoro-3-(4-methoxyphenyl)-5-thiocyanato-1H-pyrrole-2-carboxylate (**2l**) in $CDCl_3$ at 376 MHz

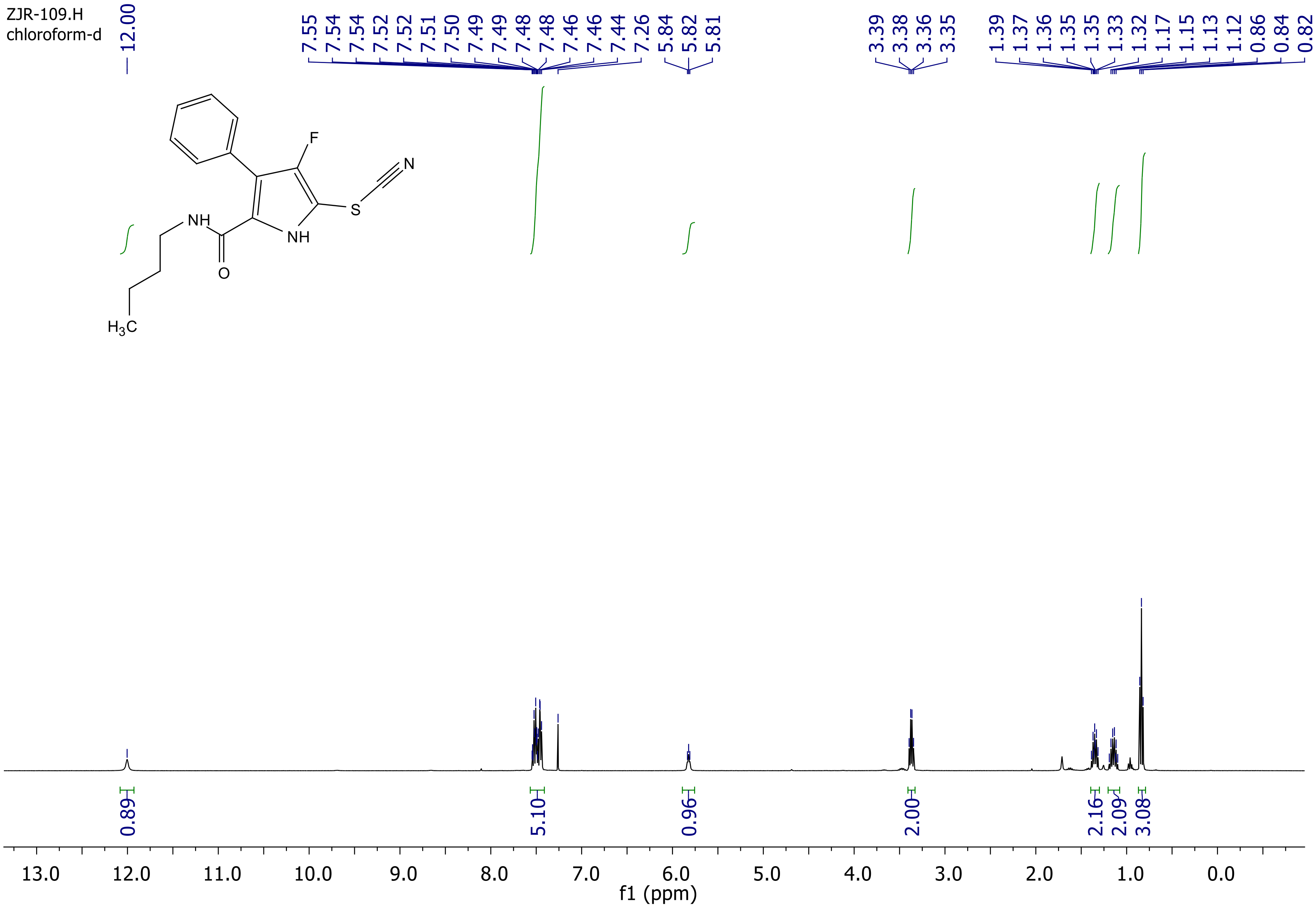

^{1}H NMR spectrum of N-butyl-4-fluoro-3-phenyl-5-thiocyanato-1H-pyrrole-2-carboxamide (**2m**) in $CDCl_3$ at 400 MHz

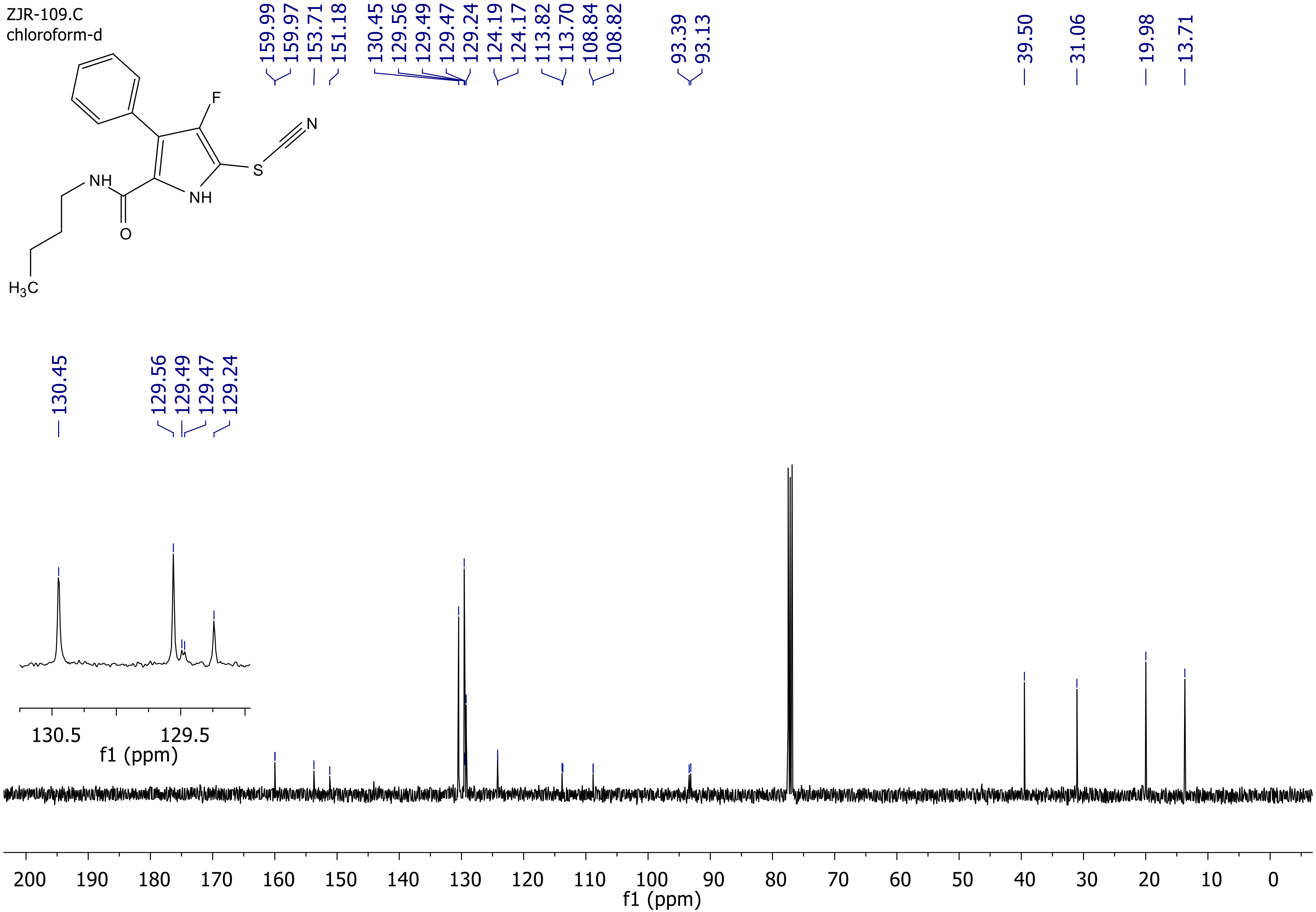

^{13}C NMR spectrum of N-butyl-4-fluoro-3-phenyl-5-thiocyanato-1H-pyrrole-2-carboxamide (**2m**) in $CDCl_3$ at 100 MHz

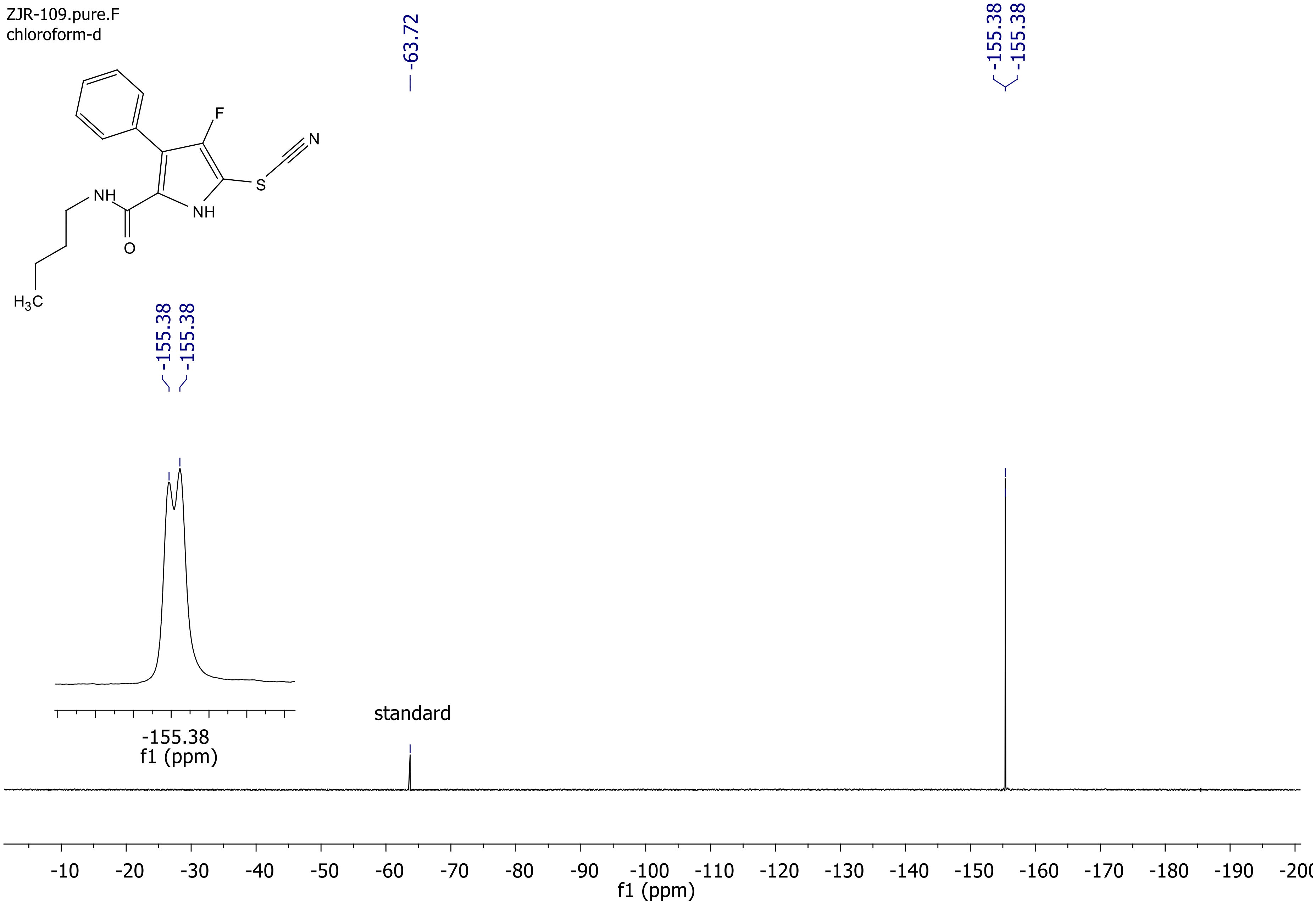

^{19}F NMR spectrum of N-butyl-4-fluoro-3-phenyl-5-thiocyanato-1H-pyrrole-2-carboxamide (**2m**) in $CDCl_3$ at 376 MHz

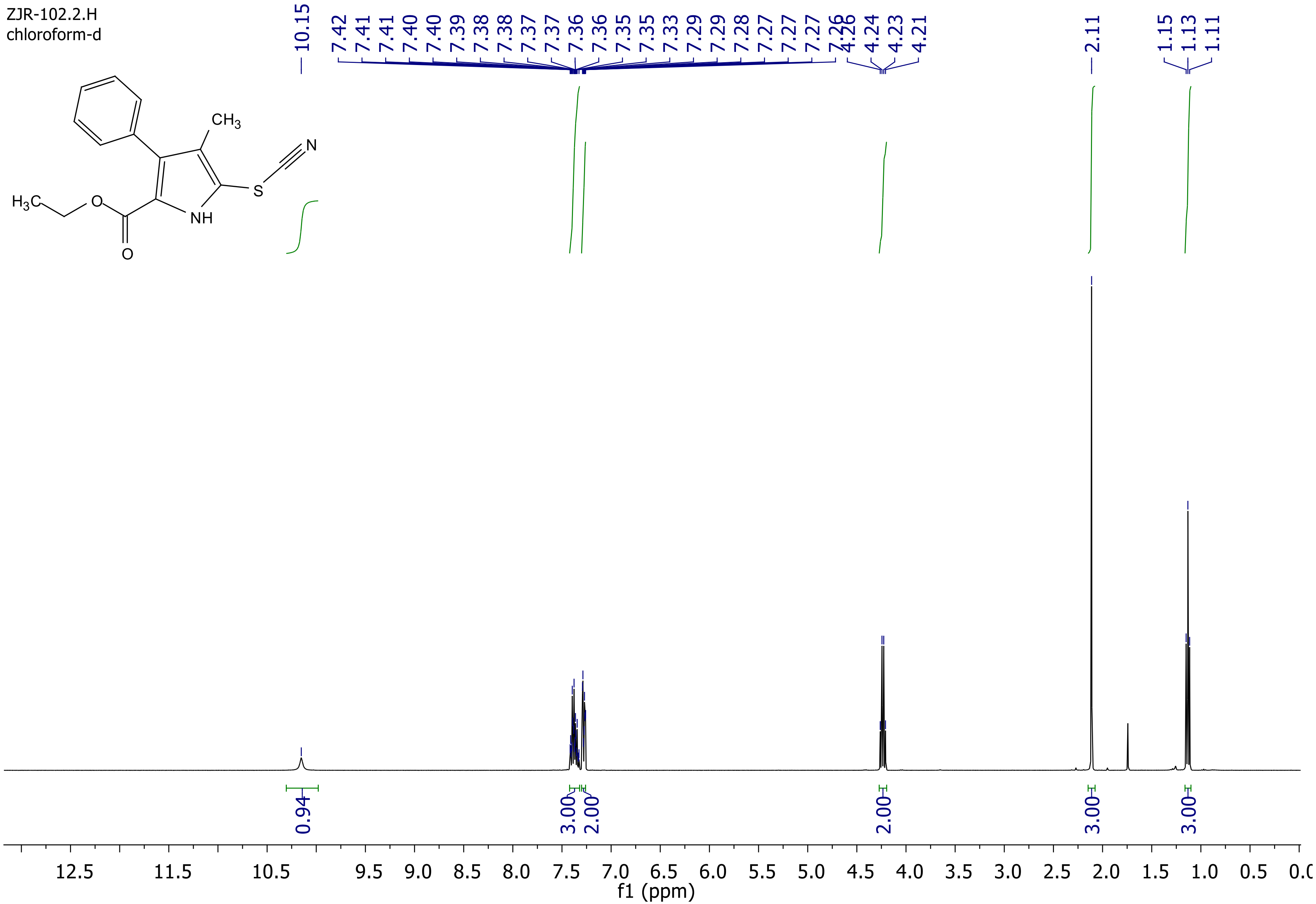

^1H NMR spectrum of ethyl 4-methyl-3-phenyl-5-thiocyanato-1H-pyrrole-2-carboxylate (**2n**) in $CDCl_3$ at 400 MHz

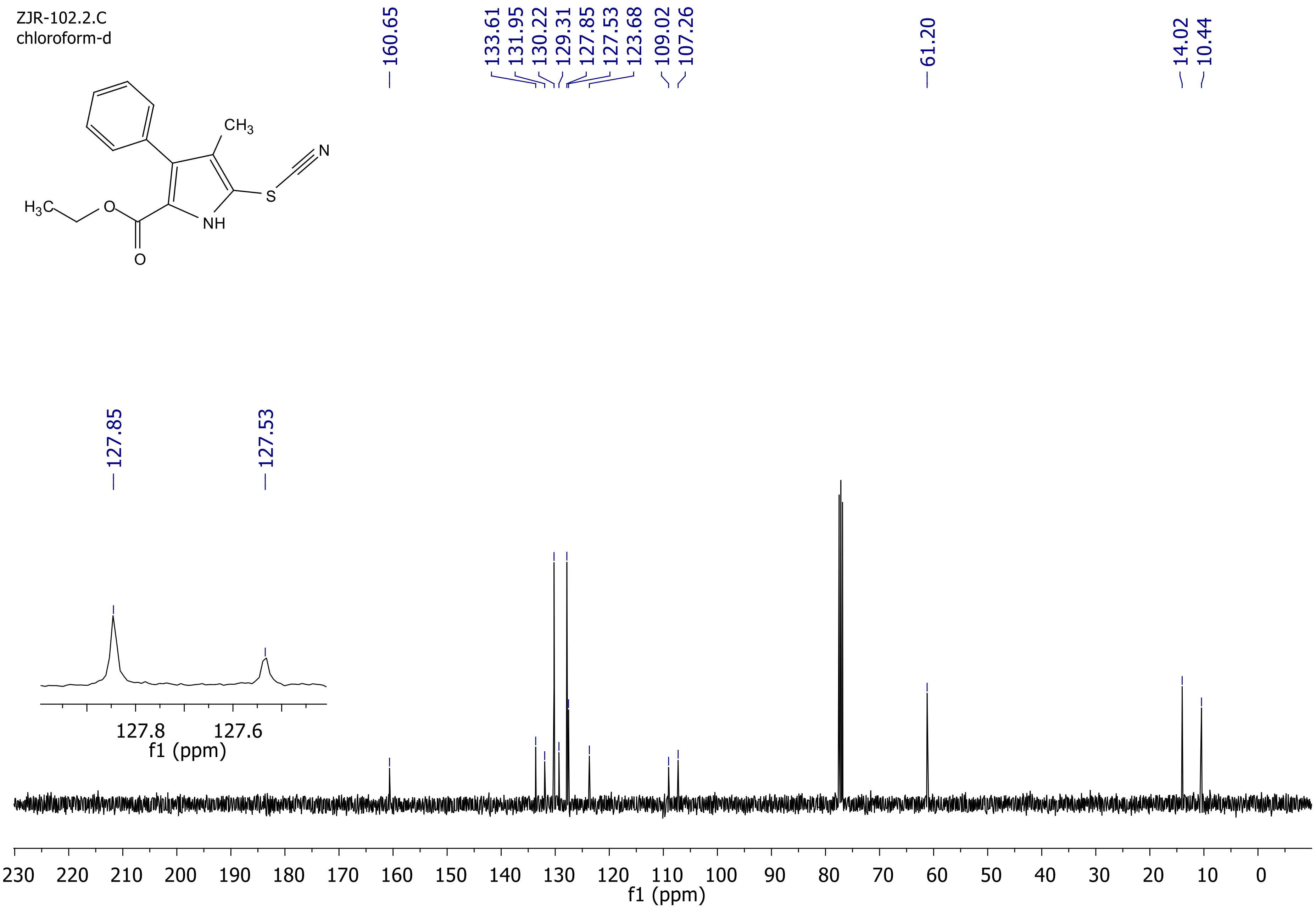

^{13}C NMR spectrum of ethyl 4-methyl-3-phenyl-5-thiocyanato-1H-pyrrole-2-carboxylate (**2n**) in $CDCl_3$ at 100 MHz

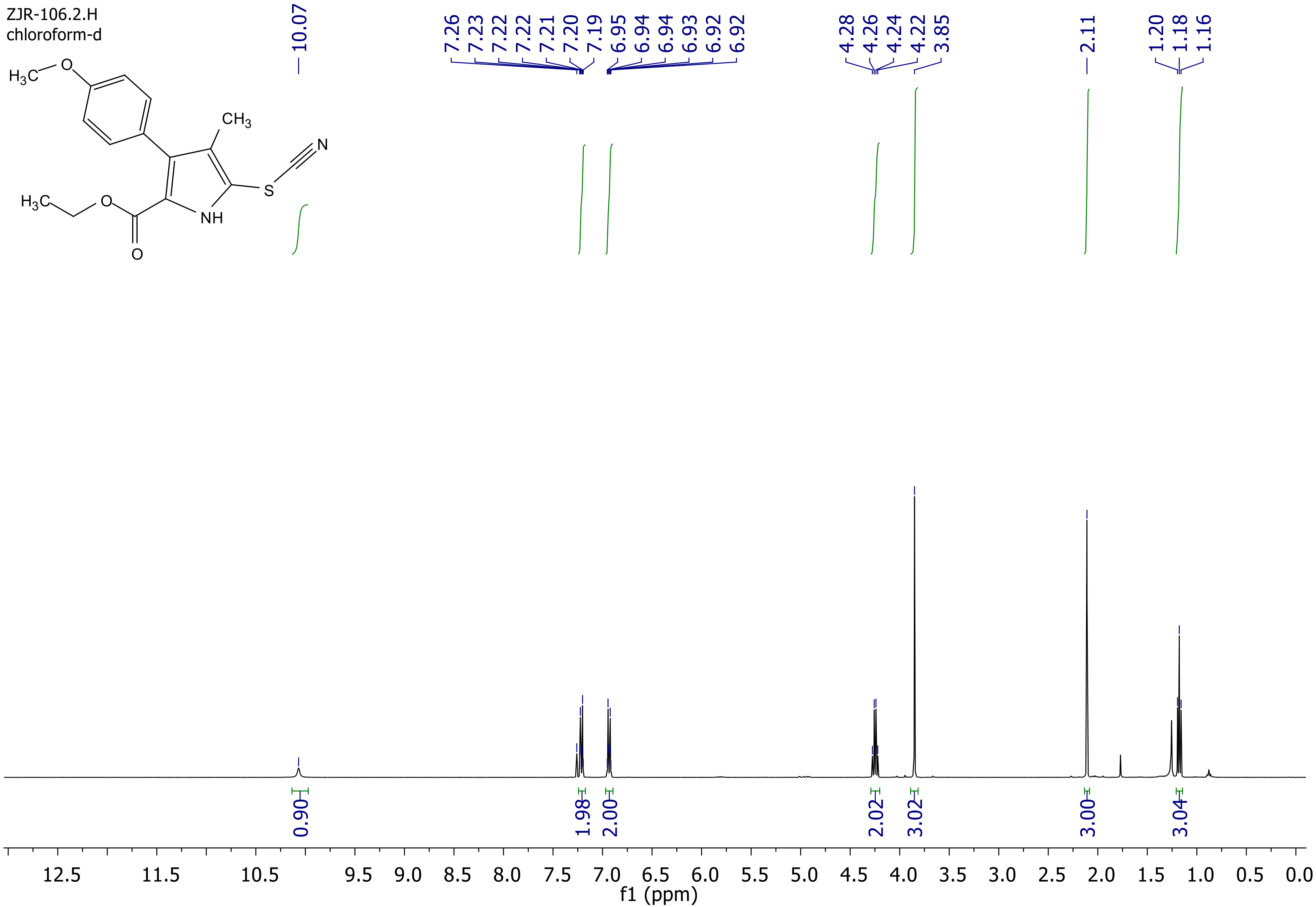

^{1}H NMR spectrum of ethyl 3-(4-methoxyphenyl)-4-methyl-5-thiocyanato-1H-pyrrole-2-carboxylate (**2o**) in $CDCl_3$ at 400 MHz

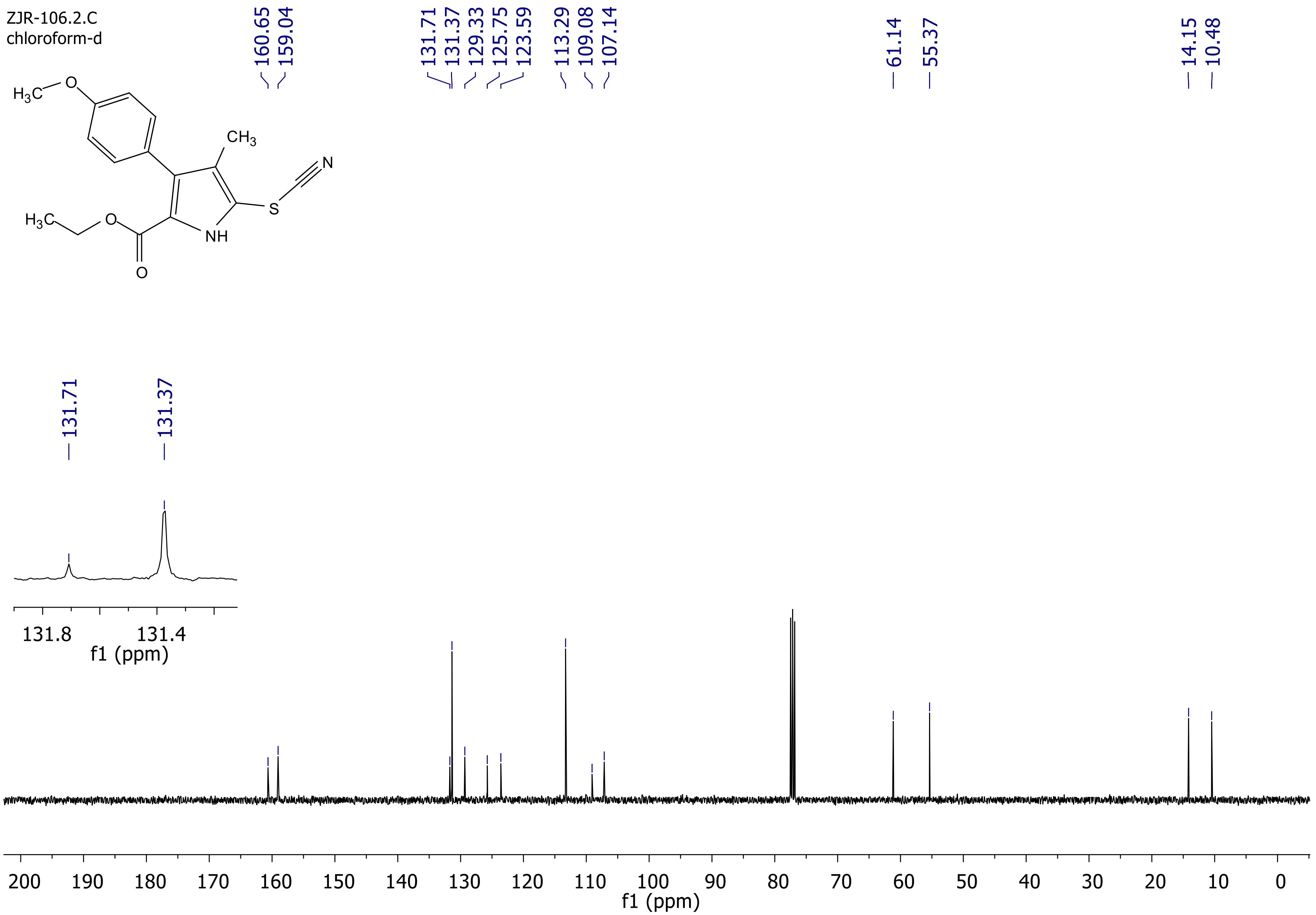

^{13}C NMR spectrum of ethyl 3-(4-methoxyphenyl)-4-methyl-5-thiocyanato-1H-pyrrole-2-carboxylate (**2o**) in $CDCl_3$ at 100 MHz

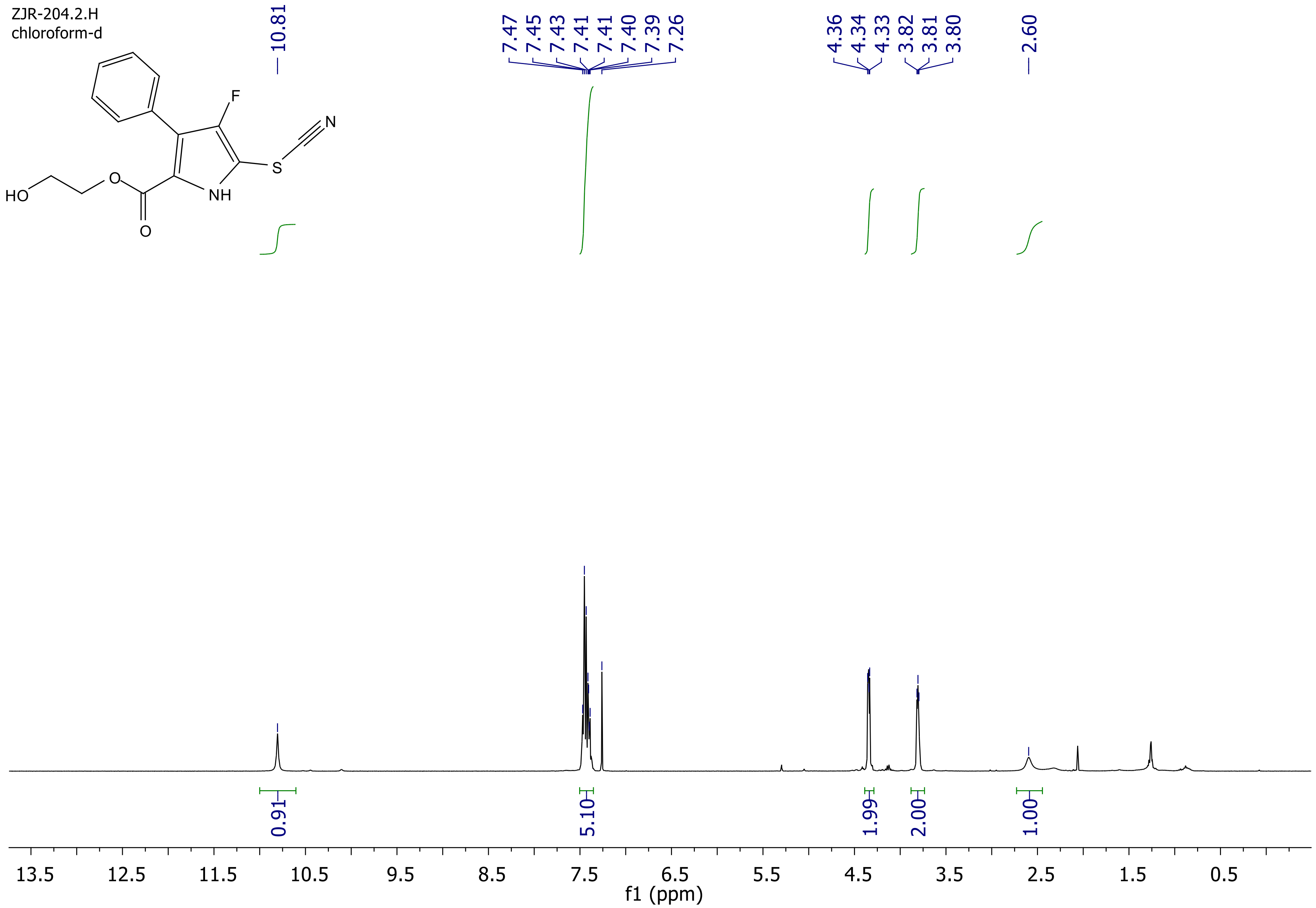

^{1}H NMR spectrum of 2-hydroxyethyl 4-fluoro-3-phenyl-5-thiocyanato-1H-pyrrole-2-carboxylate (**2p**) in $CDCl_3$ at 400 MHz

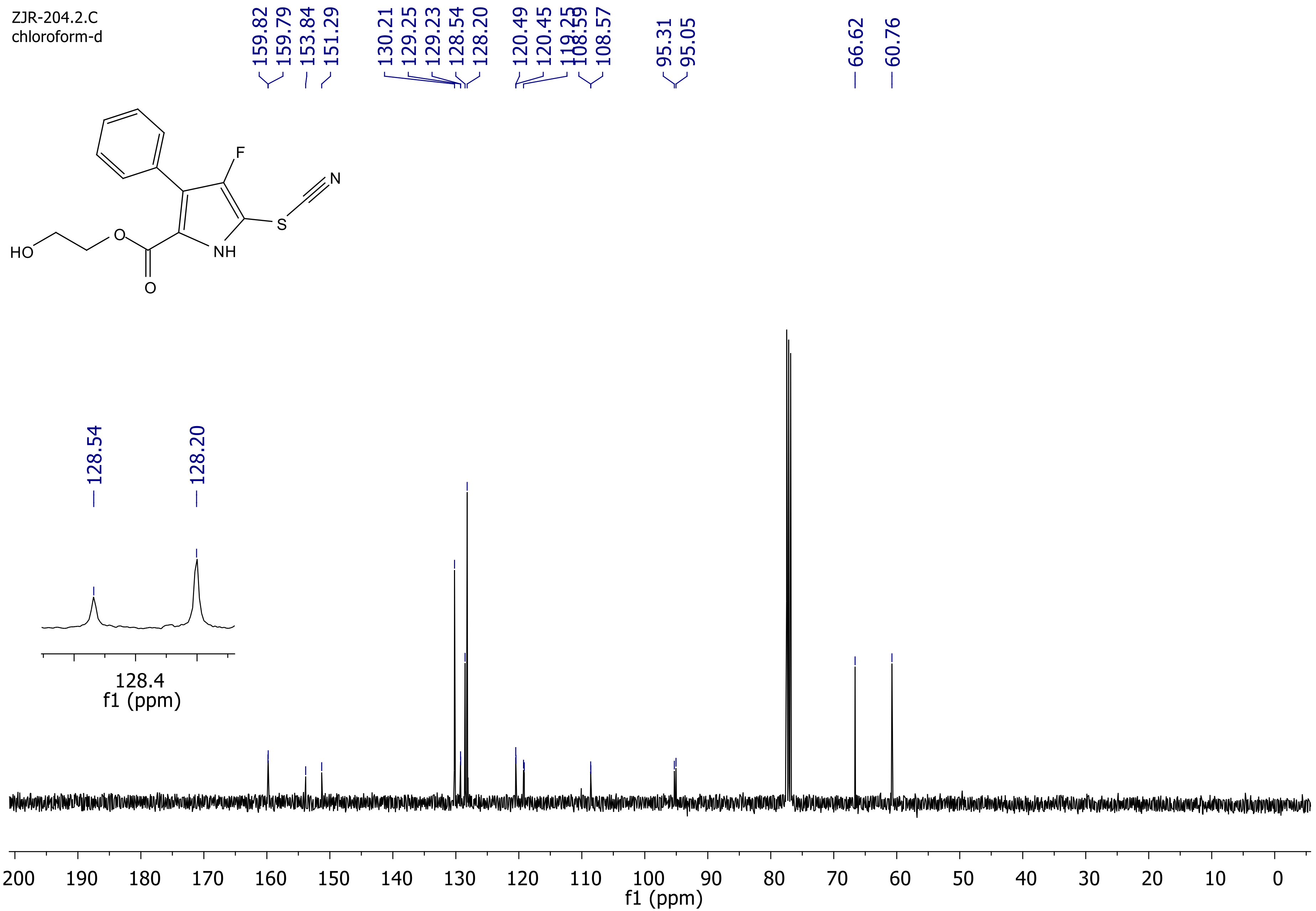

^{13}C NMR spectrum of 2-hydroxyethyl 4-fluoro-3-phenyl-5-thiocyanato-1H-pyrrole-2-carboxylate (**2p**) in $CDCl_3$ at 100 MHz

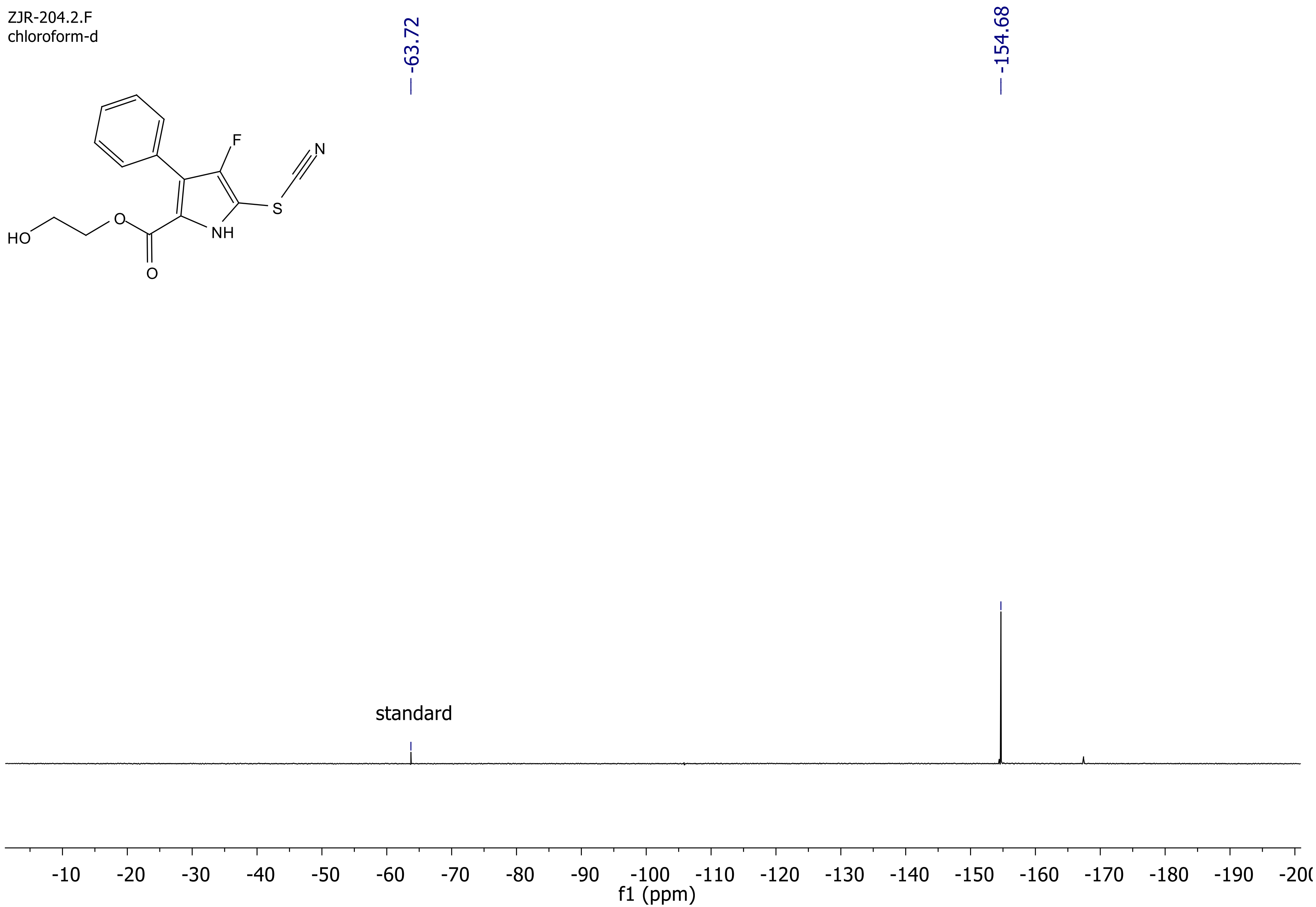

^{19}F NMR spectrum of 2-hydroxyethyl 4-fluoro-3-phenyl-5-thiocyanato-1H-pyrrole-2-carboxylate (**2p**) in $CDCl_3$ at 376 MHz

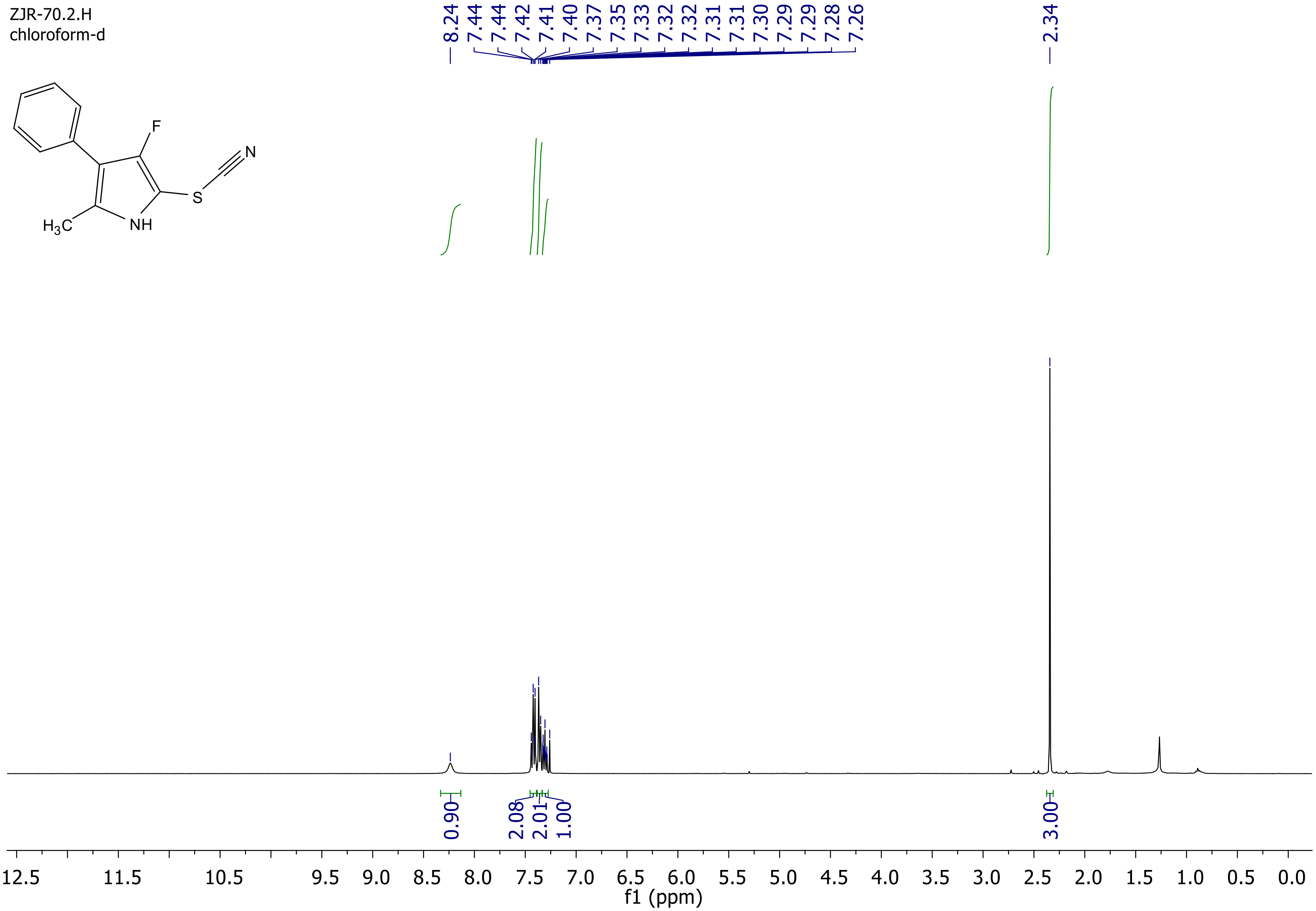

^{1}H NMR spectrum of 3-fluoro-5-methyl-4-phenyl-2-thiocyanato-1H-pyrrole (**2q**) in $CDCl_3$ at 400 MHz

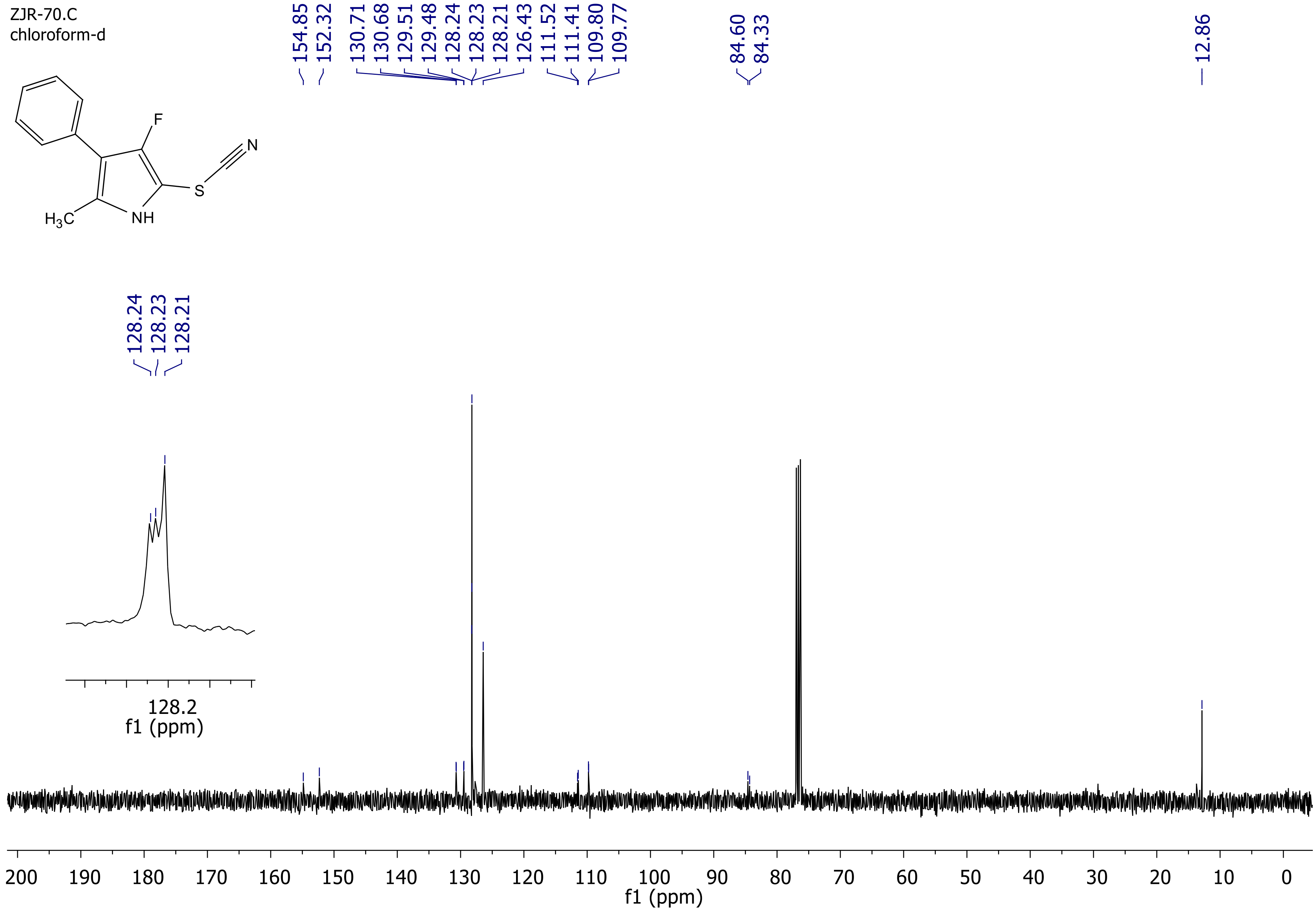

^{13}C NMR spectrum of 3-fluoro-5-methyl-4-phenyl-2-thiocyanato-1H-pyrrole (**2q**) in $CDCl_3$ at 100 MHz

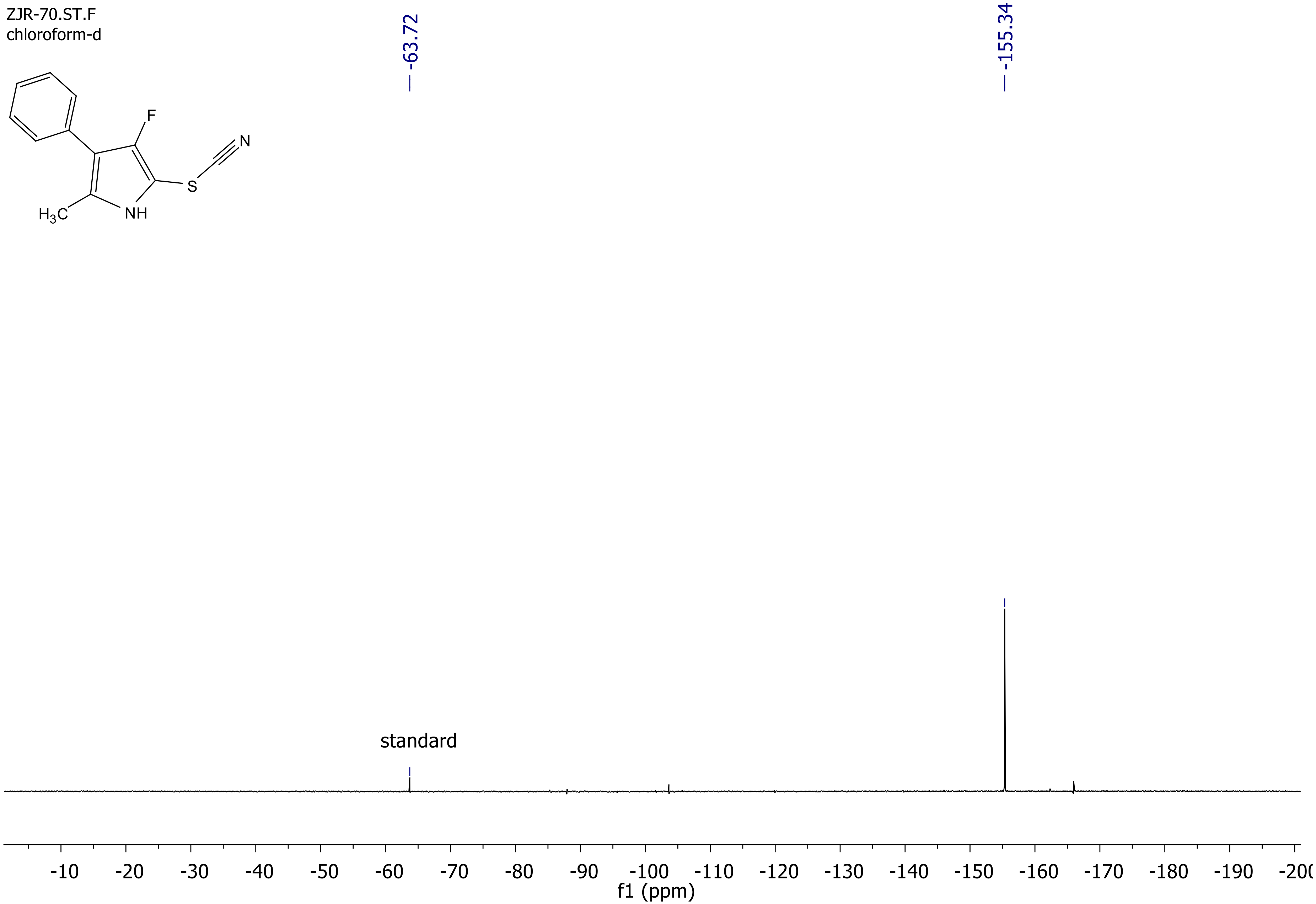

^{19}F NMR spectrum of 3-fluoro-5-methyl-4-phenyl-2-thiocyanato-1H-pyrrole (**2q**) in $CDCl_3$ at 376 MHz

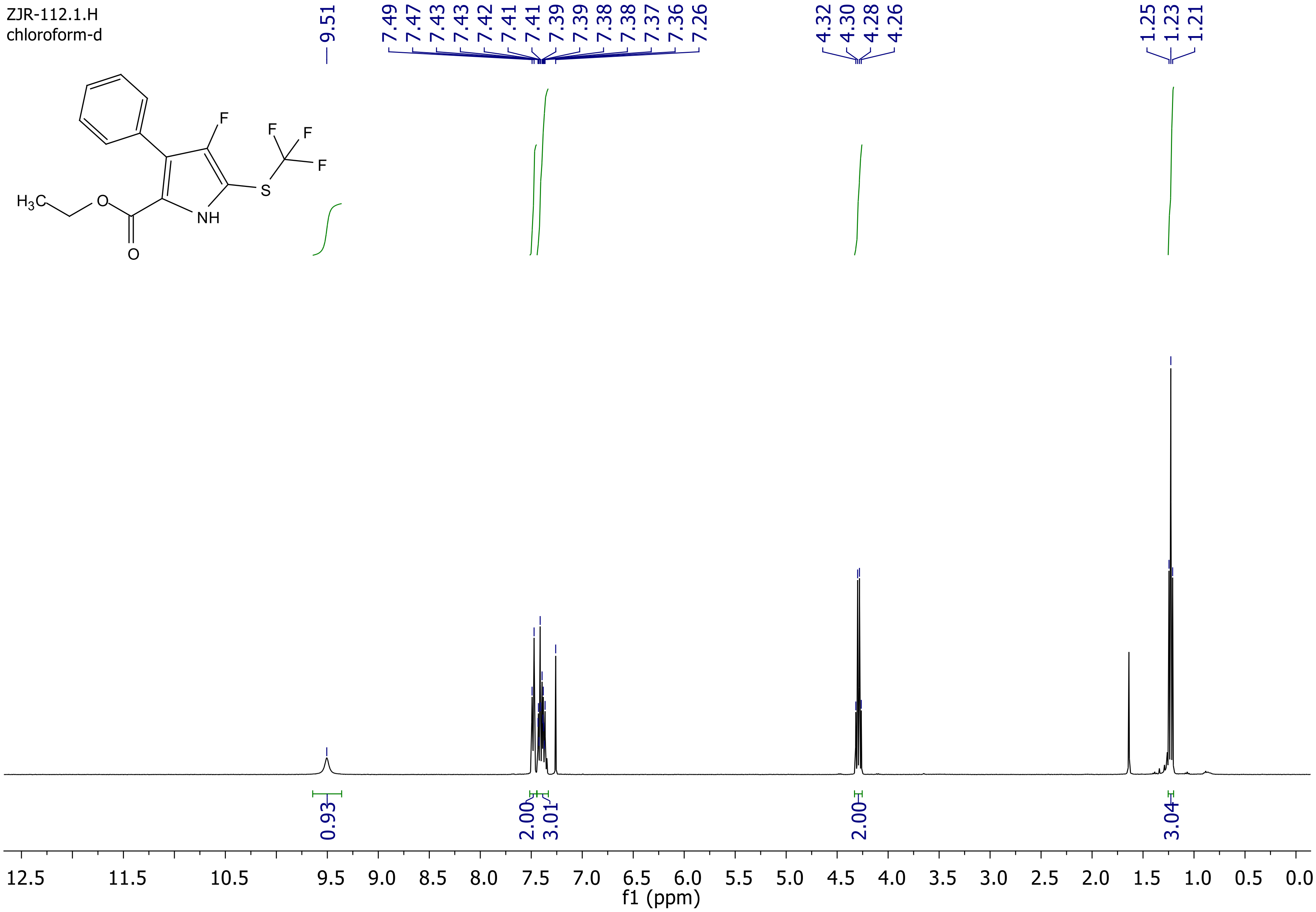

^{1}H NMR spectrum of ethyl 4-fluoro-3-phenyl-5-((trifluoromethyl)thio)-1H-pyrrole-2-carboxylate (**4a**) in $CDCl_3$ at 400 MHz

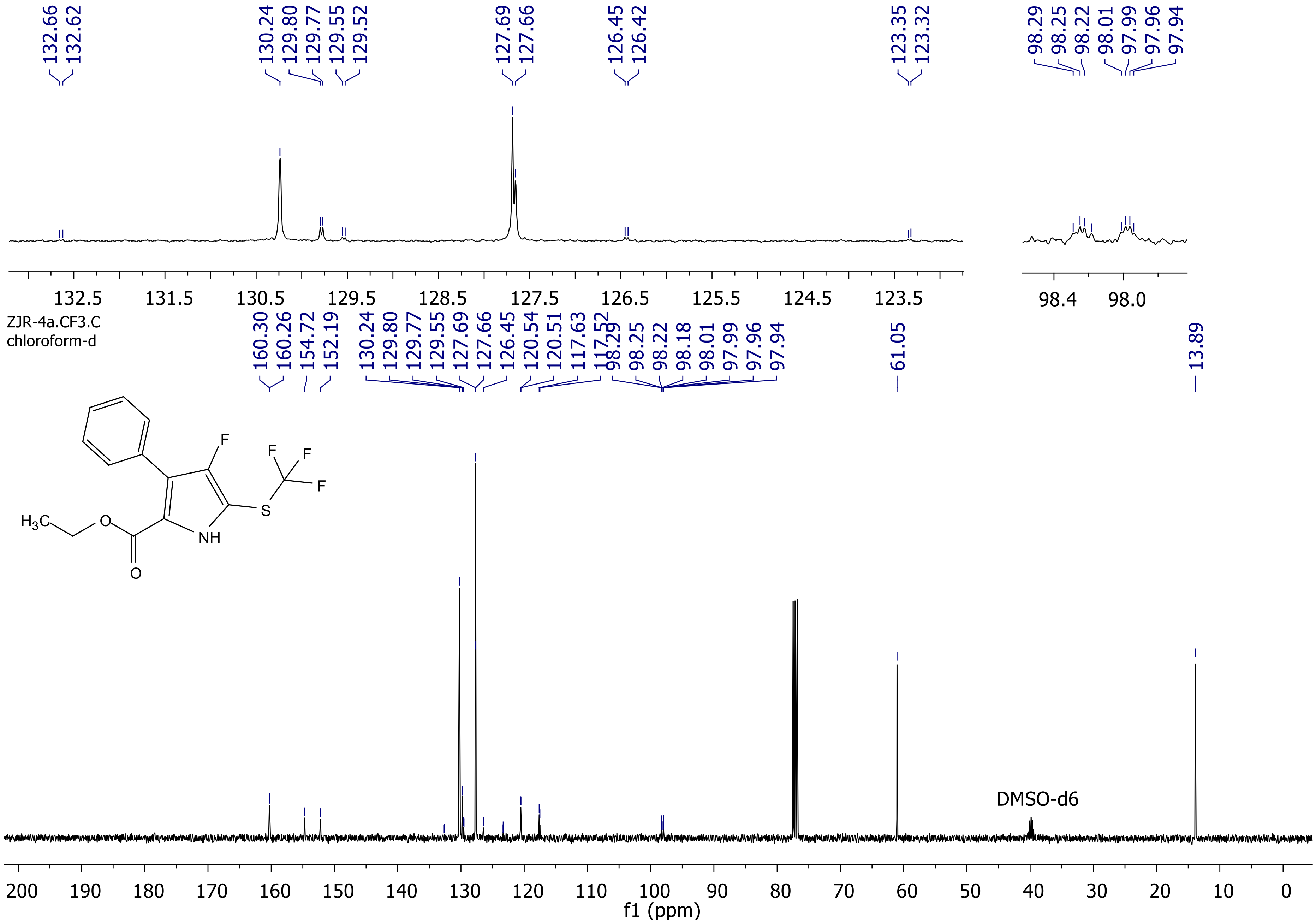

^{13}C NMR spectrum of ethyl 4-fluoro-3-phenyl-5-((trifluoromethyl)thio)-1H-pyrrole-2-carboxylate (**4a**) in $CDCl_3$ at 100 MHz

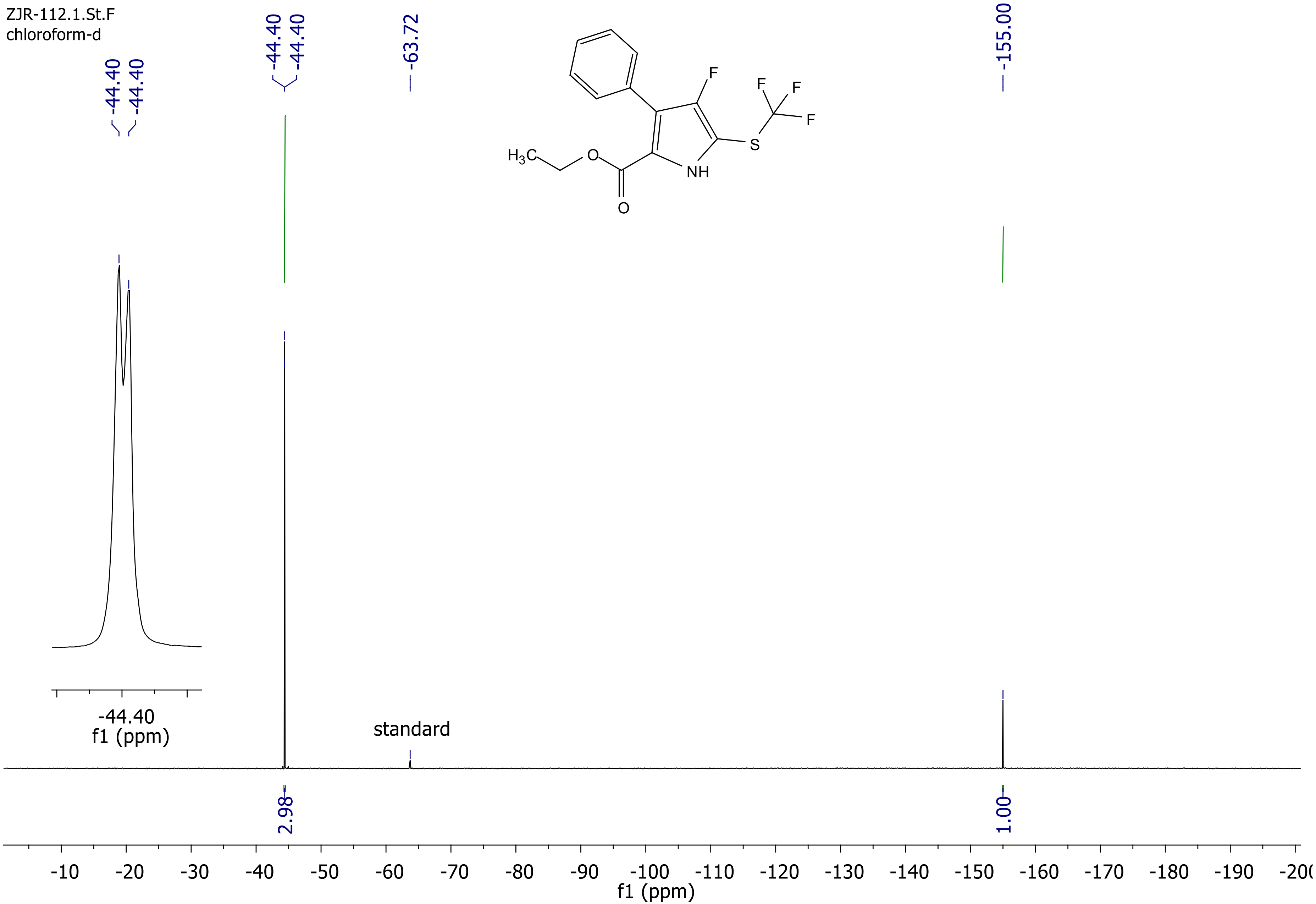

^{19}F NMR spectrum of ethyl 4-fluoro-3-phenyl-5-((trifluoromethyl)thio)-1H-pyrrole-2-carboxylate (**4a**) in $CDCl_3$ at 376 MHz

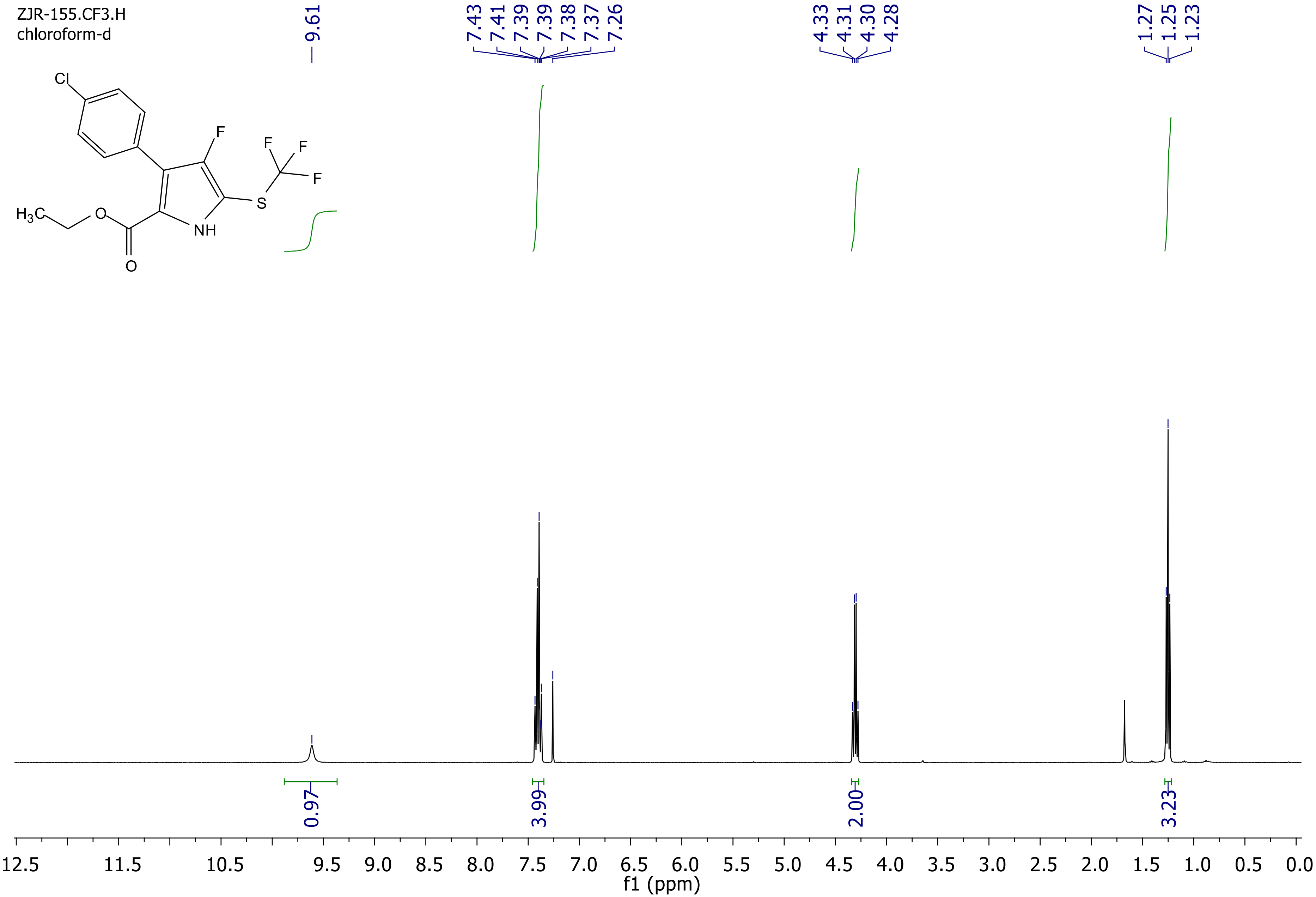

^{1}H NMR spectrum of ethyl 3-(4-chlorophenyl)-4-fluoro-5-((trifluoromethyl)thio)-1H-pyrrole-2-carboxylate (**4b**) in $CDCl_3$ at 400 MHz

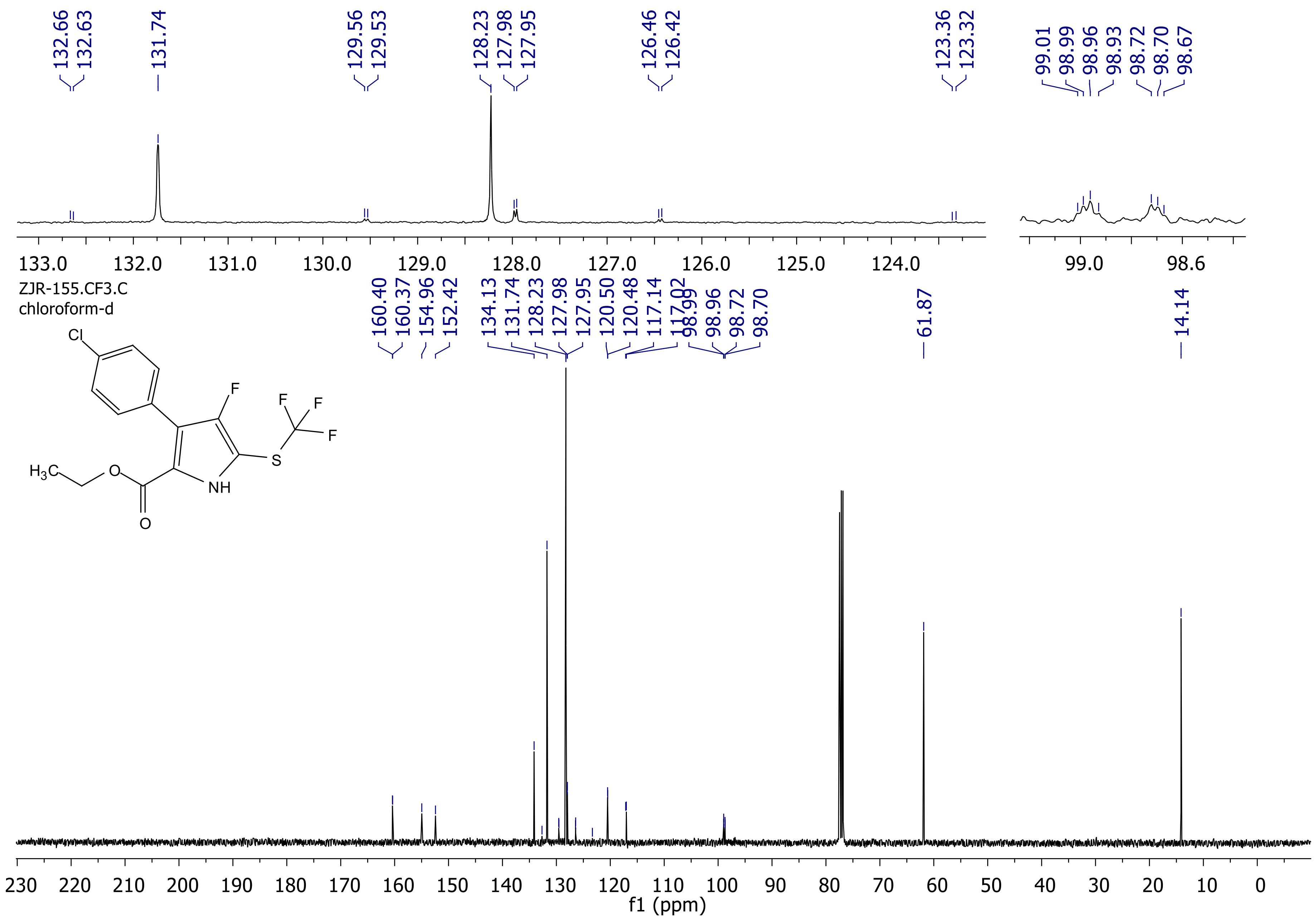

^{13}C NMR spectrum of ethyl 3-(4-chlorophenyl)-4-fluoro-5-((trifluoromethyl)thio)-1H-pyrrole-2-carboxylate (**4b**) in $CDCl_3$ at 100 MHz

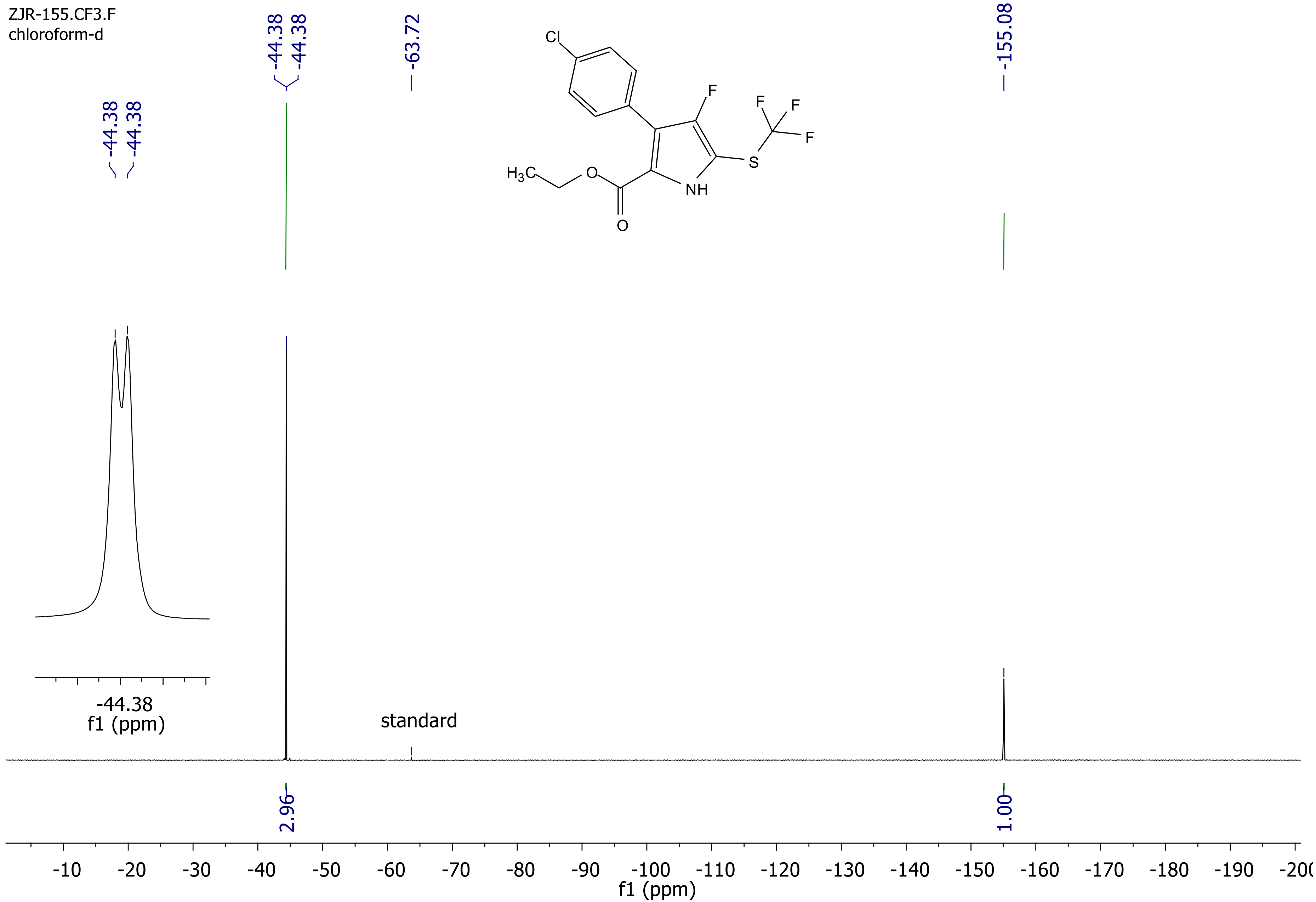

^{19}F NMR spectrum of ethyl 3-(4-chlorophenyl)-4-fluoro-5-((trifluoromethyl)thio)-1H-pyrrole-2-carboxylate (**4b**) in $CDCl_3$ at 376 MHz

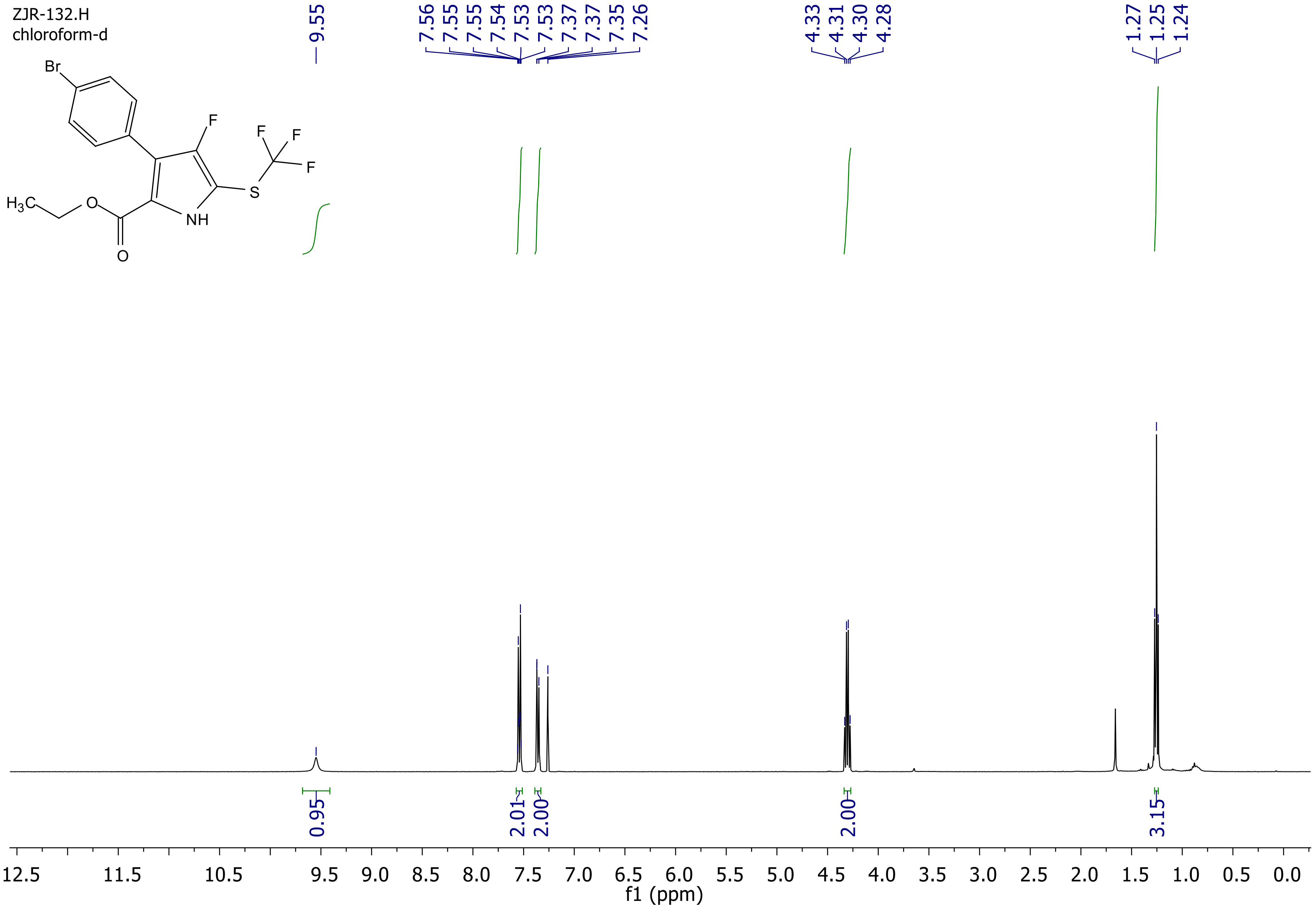

^{1}H NMR spectrum of ethyl 3-(4-bromophenyl)-4-fluoro-5-((trifluoromethyl)thio)-1H-pyrrole-2-carboxylate (**4c**) in $CDCl_3$ at 400 MHz

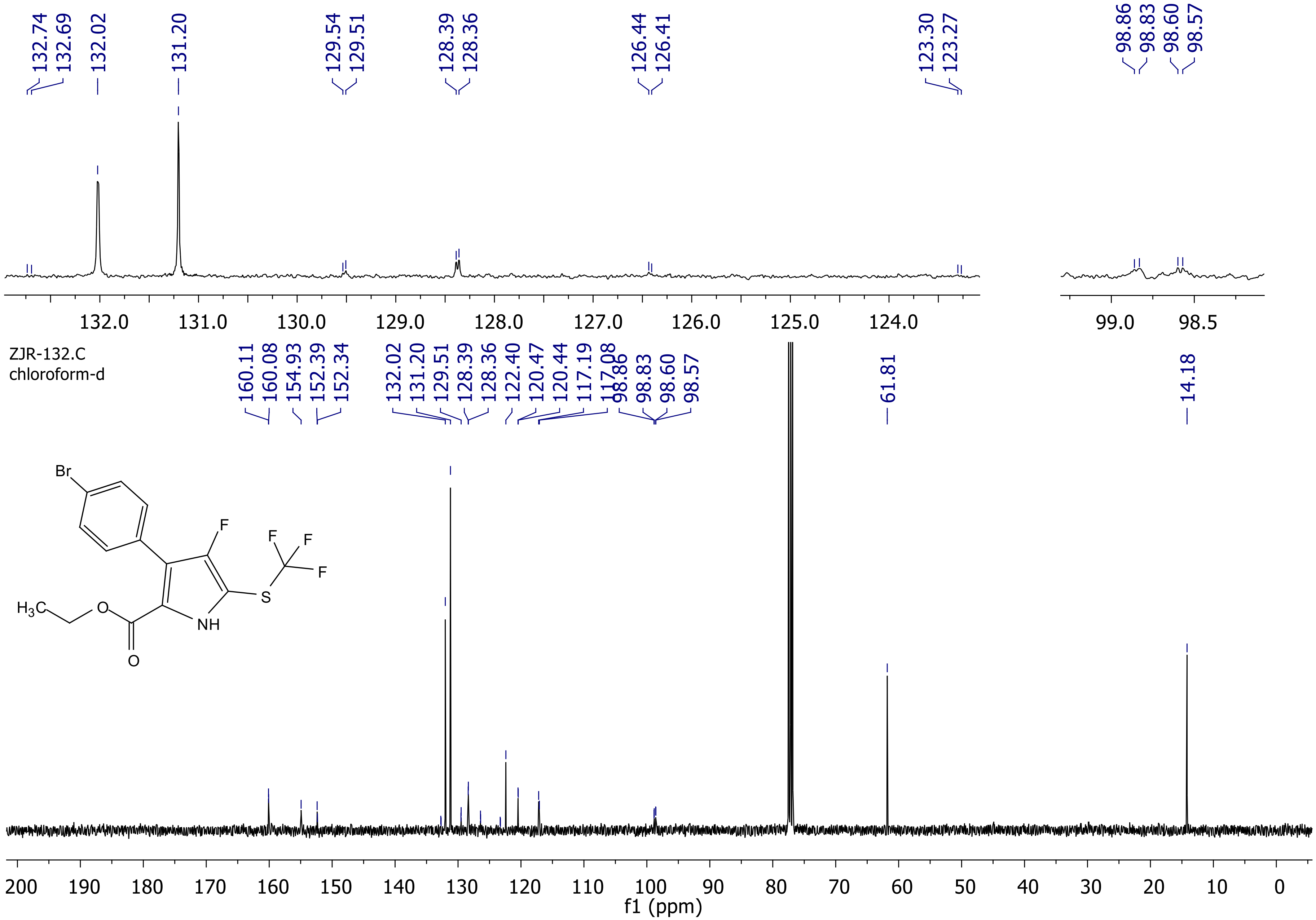

^{13}C NMR spectrum of ethyl 3-(4-bromophenyl)-4-fluoro-5-((trifluoromethyl)thio)-1H-pyrrole-2-carboxylate (**4c**) in $CDCl_3$ at 100 MHz

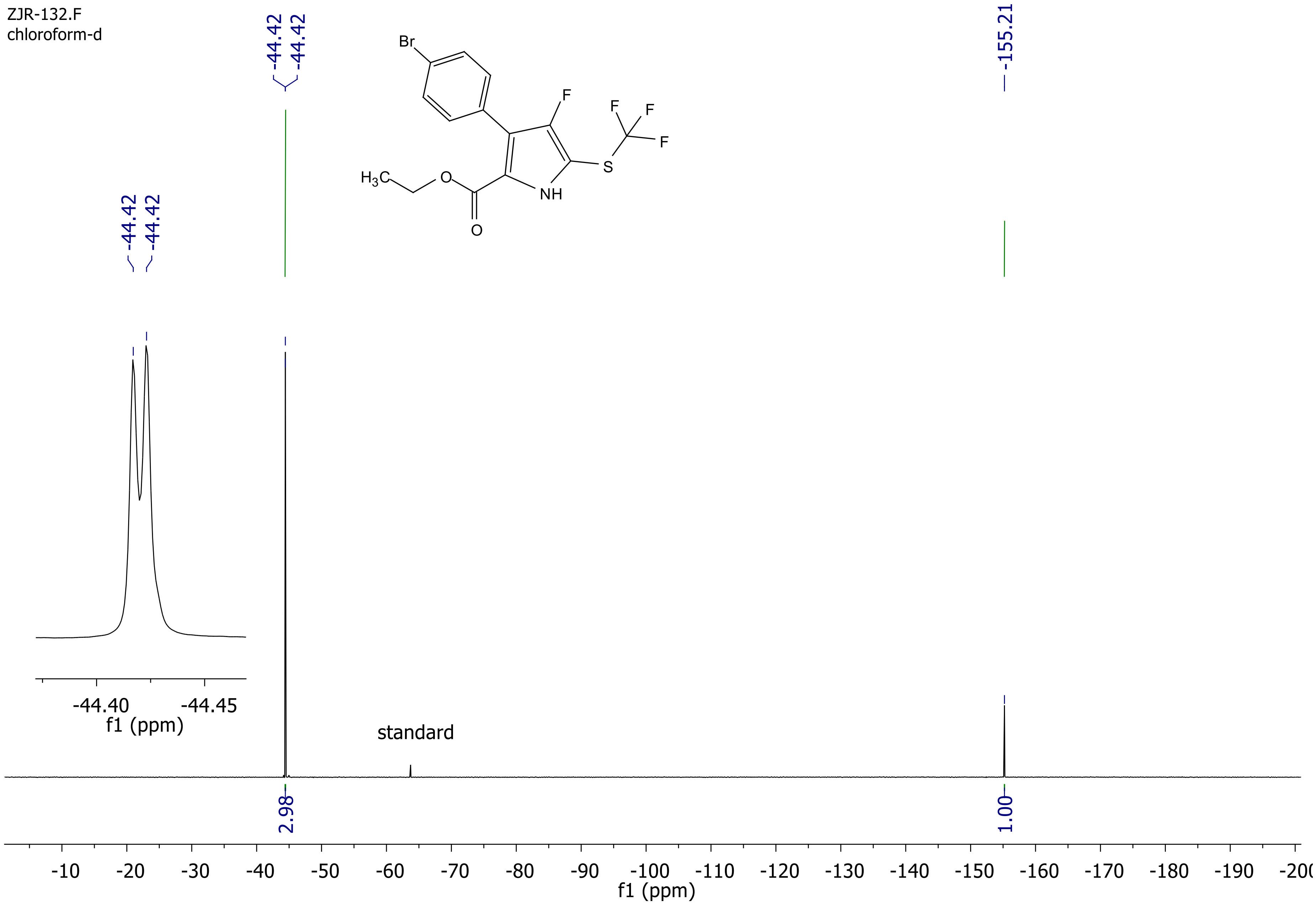

^{19}F NMR spectrum of ethyl 3-(4-bromophenyl)-4-fluoro-5-((trifluoromethyl)thio)-1H-pyrrole-2-carboxylate (**4c**) in $CDCl_3$ at 376 MHz

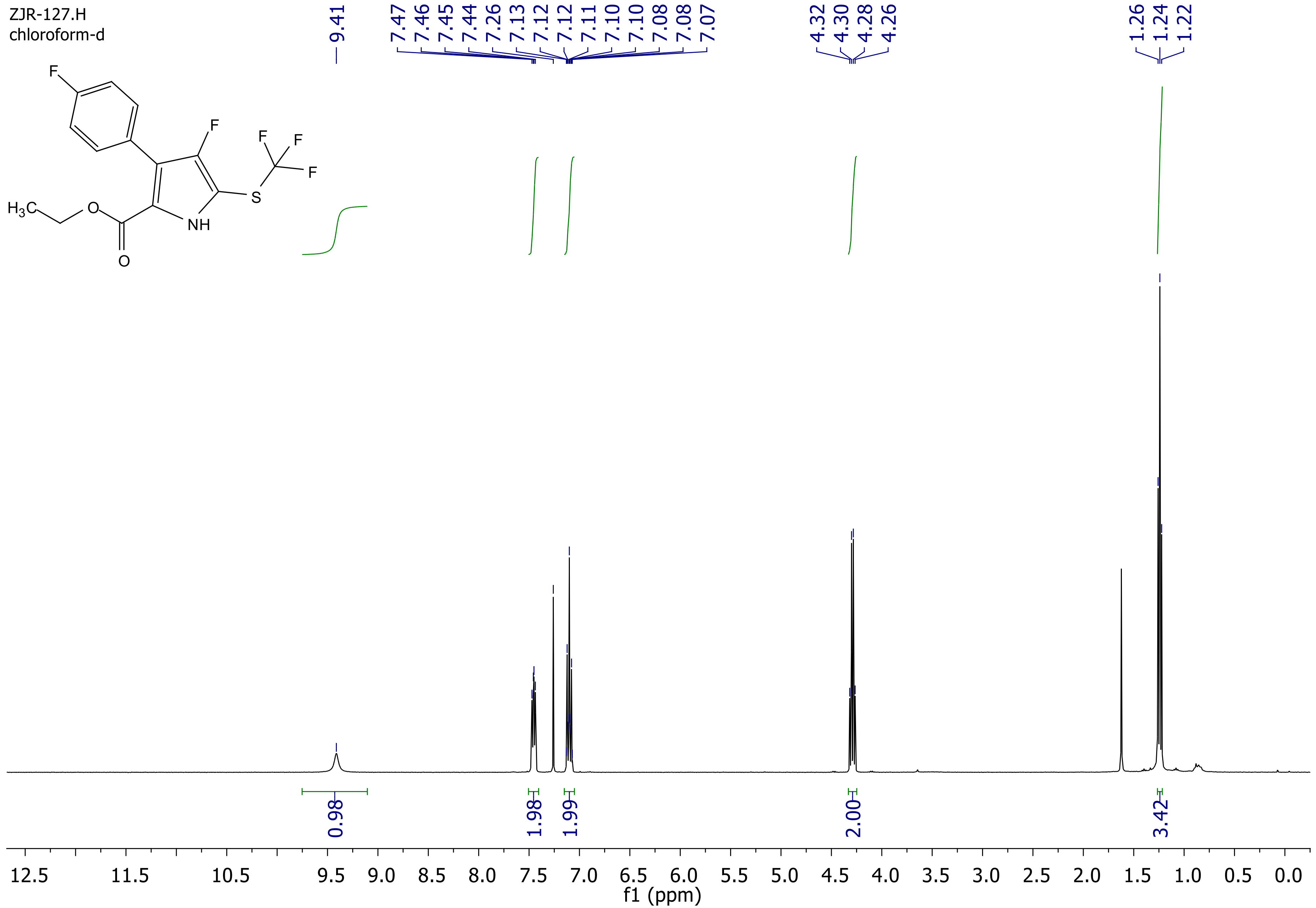

^{1}H NMR spectrum of ethyl 4-fluoro-3-(4-fluorophenyl)-5-((trifluoromethyl)thio)-1H-pyrrole-2-carboxylate (**4d**) in $CDCl_3$ at 400 MHz

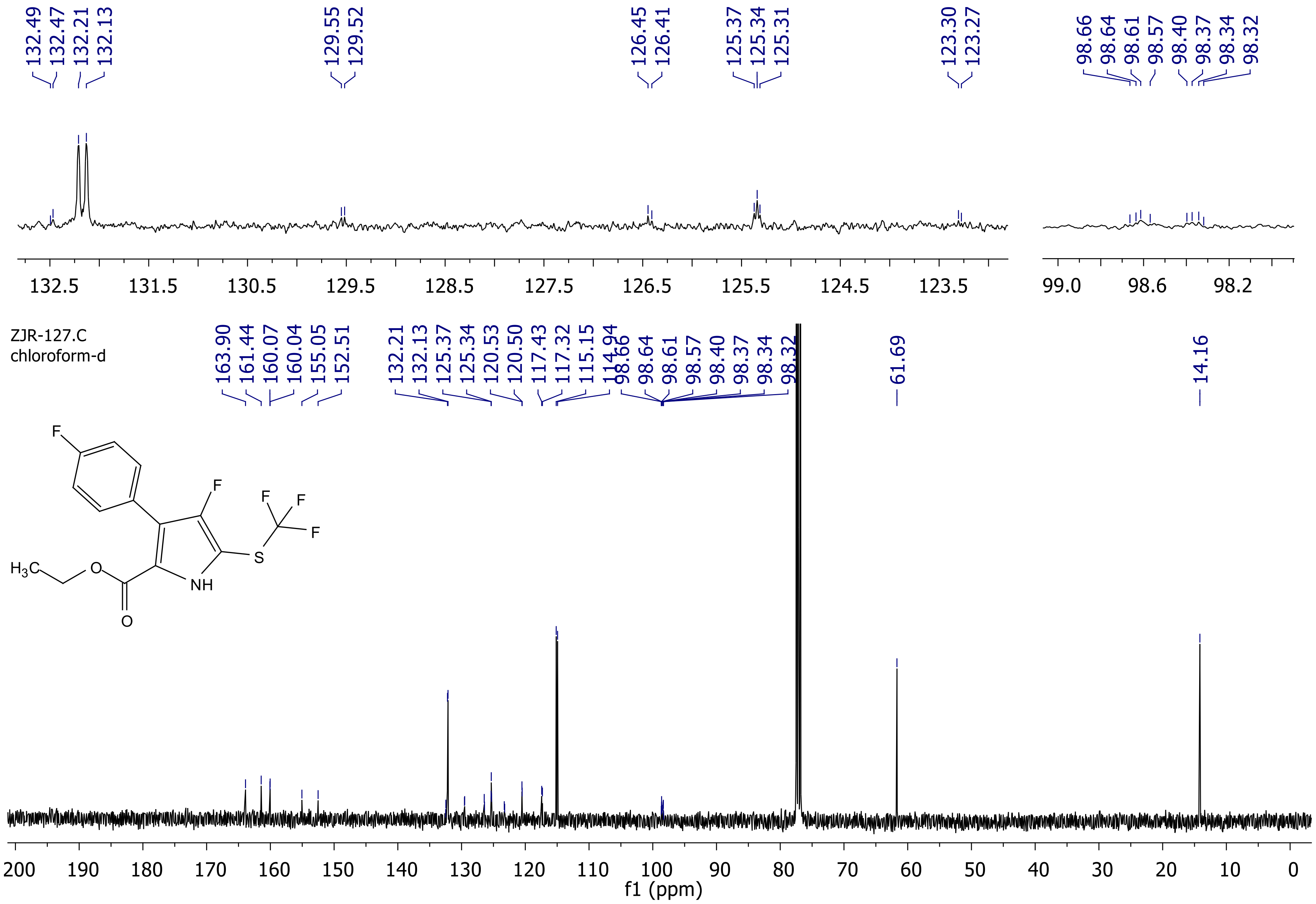

^{13}C NMR spectrum of ethyl 4-fluoro-3-(4-fluorophenyl)-5-((trifluoromethyl)thio)-1H-pyrrole-2-carboxylate (**4d**) in $CDCl_3$ at 100 MHz

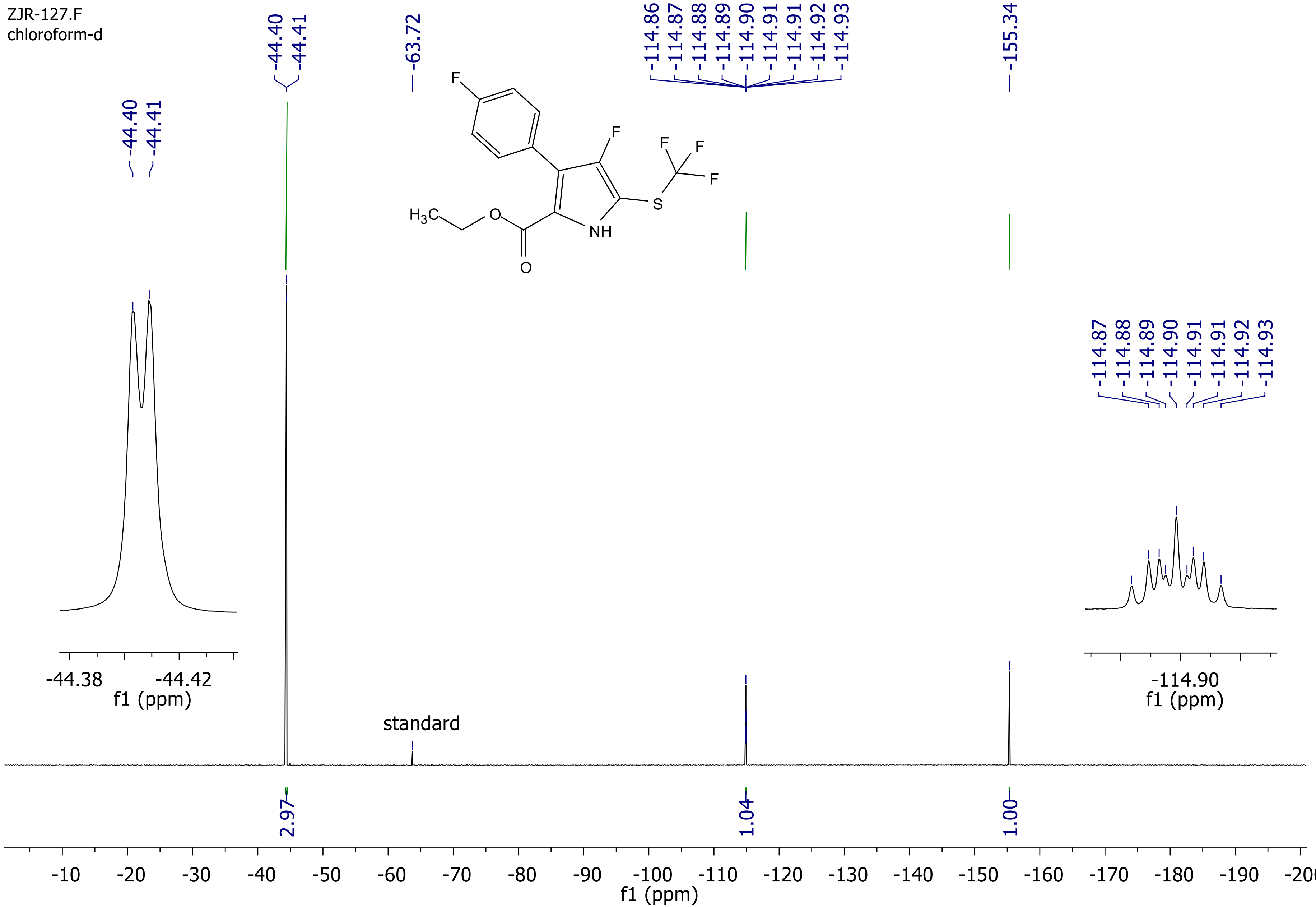

^{19}F NMR spectrum of ethyl 4-fluoro-3-(4-fluorophenyl)-5-((trifluoromethyl)thio)-1H-pyrrole-2-carboxylate (**4d**) in $CDCl_3$ at 376 MHz

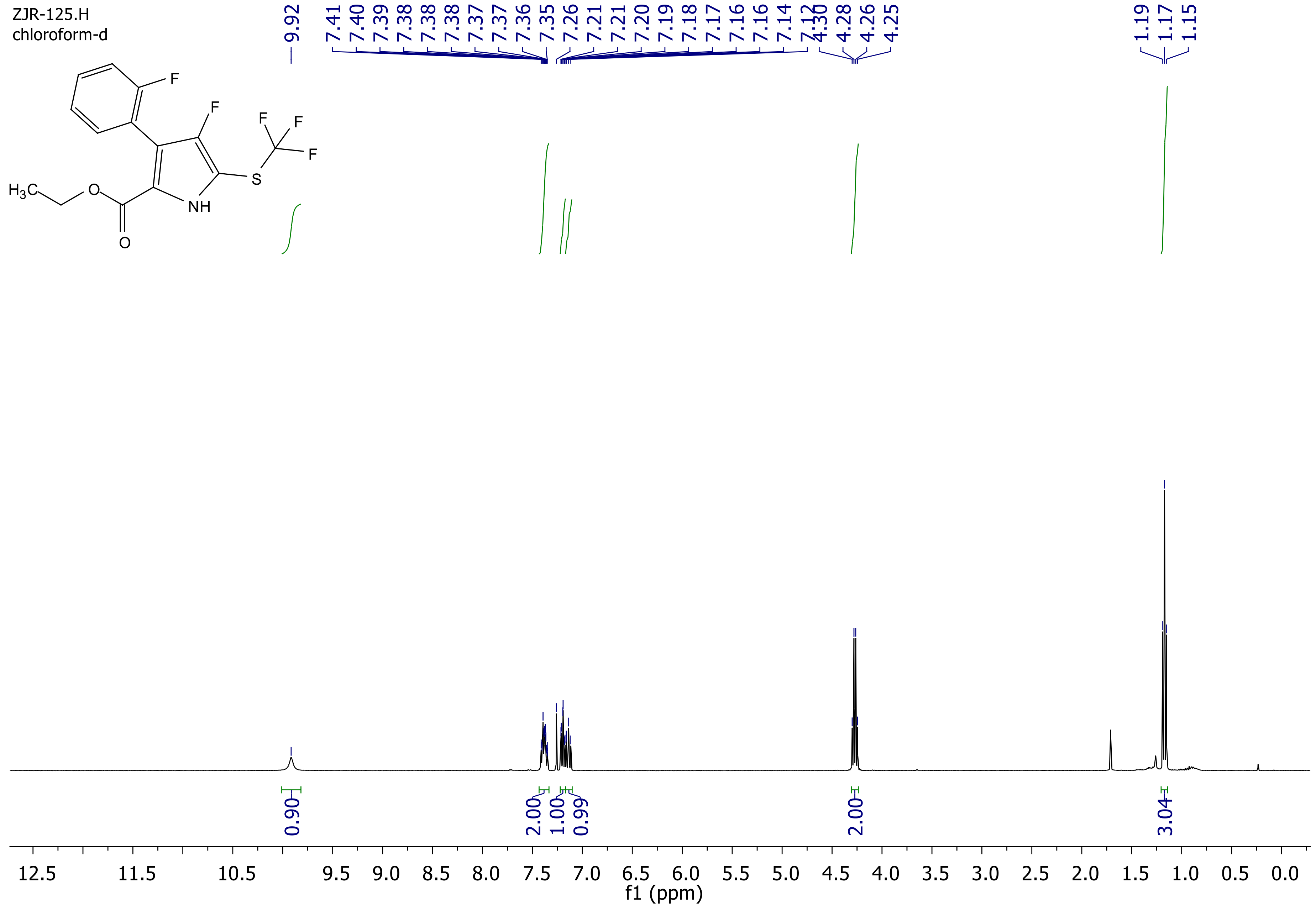

^{1}H NMR spectrum of ethyl 4-fluoro-3-(2-fluorophenyl)-5-((trifluoromethyl)thio)-1H-pyrrole-2-carboxylate (**4e**) in $CDCl_3$ at 400 MHz

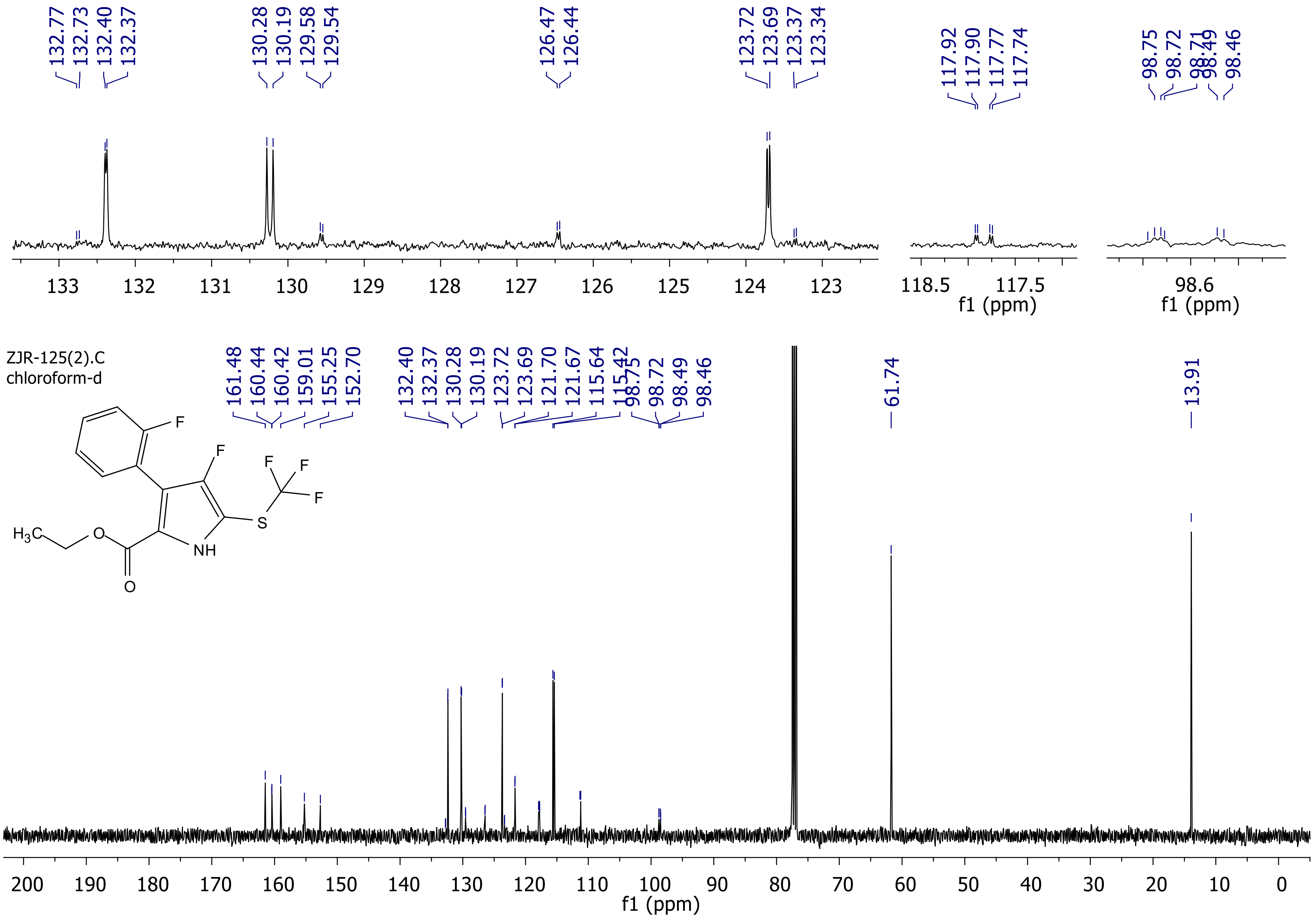

^{13}C NMR spectrum of ethyl 4-fluoro-3-(2-fluorophenyl)-5-((trifluoromethyl)thio)-1H-pyrrole-2-carboxylate (**4e**) in $CDCl_3$ at 100 MHz

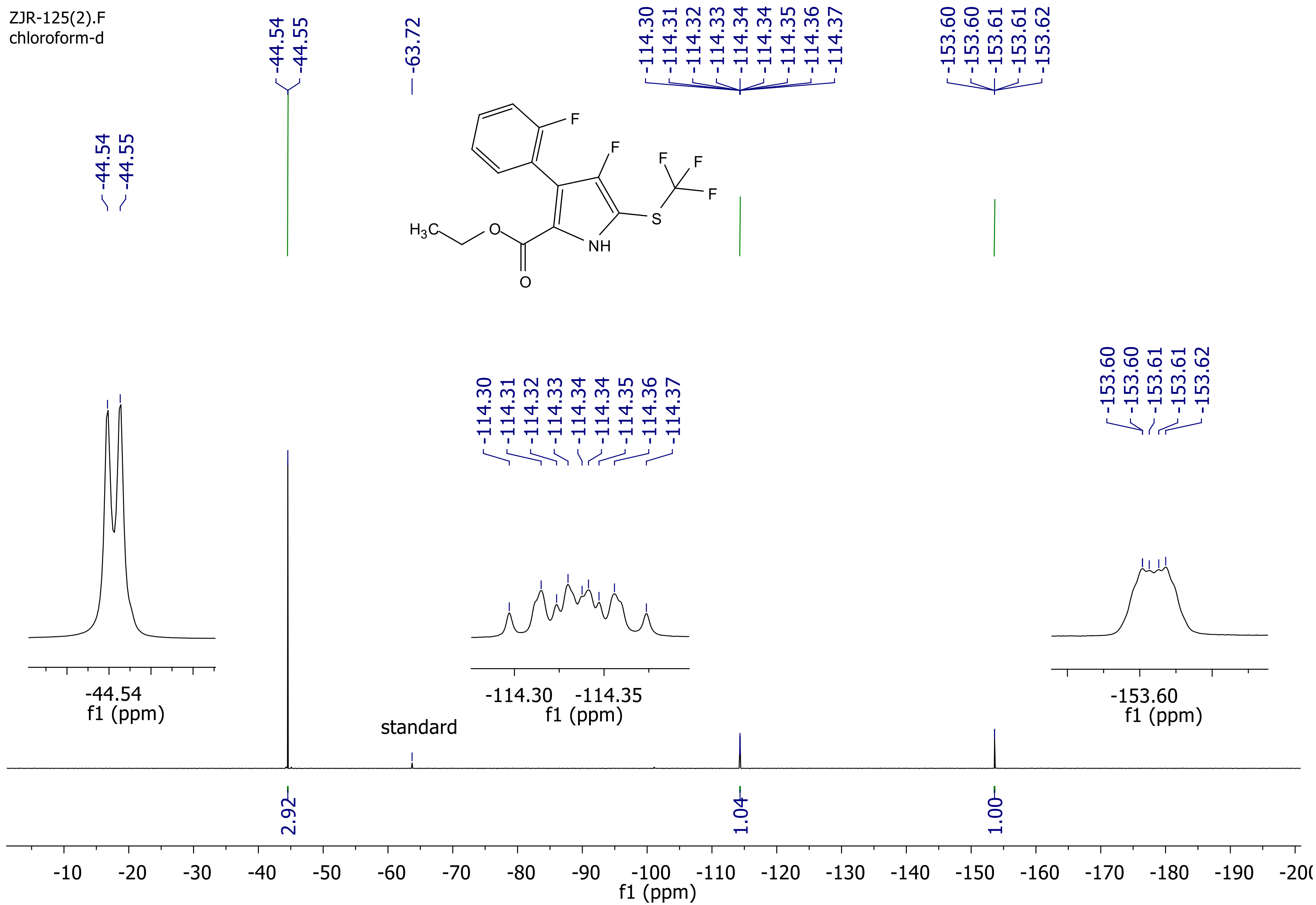

^{19}F NMR spectrum of ethyl 4-fluoro-3-(2-fluorophenyl)-5-((trifluoromethyl)thio)-1H-pyrrole-2-carboxylate (**4e**) in $CDCl_3$ at 376 MHz

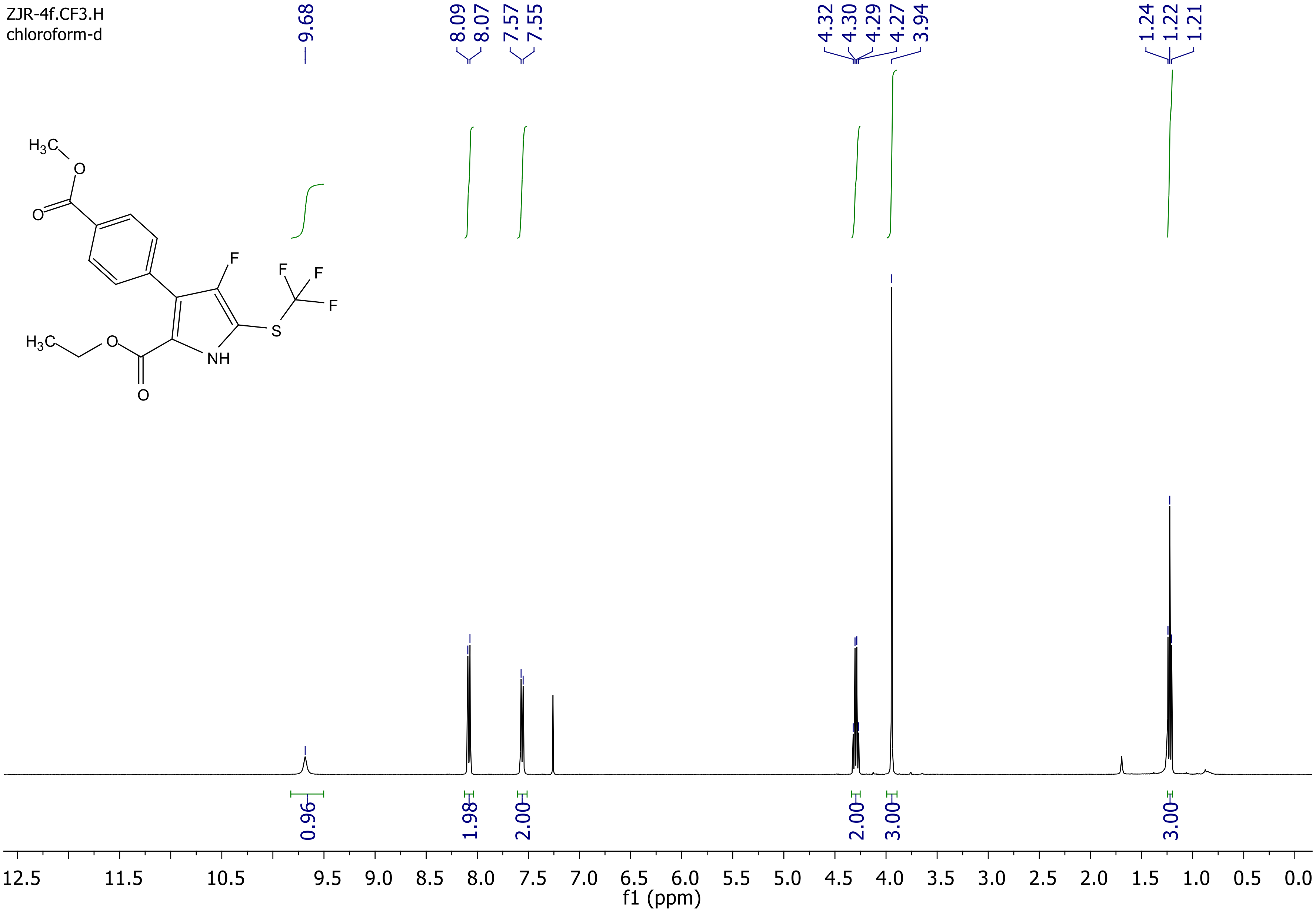

^{1}H NMR spectrum of ethyl 4-fluoro-3-(4-(methoxycarbonyl)phenyl)-5-((trifluoromethyl)thio)-1H-pyrrole-2-carboxylate (**4f**) in $CDCl_3$ at 400 MHz

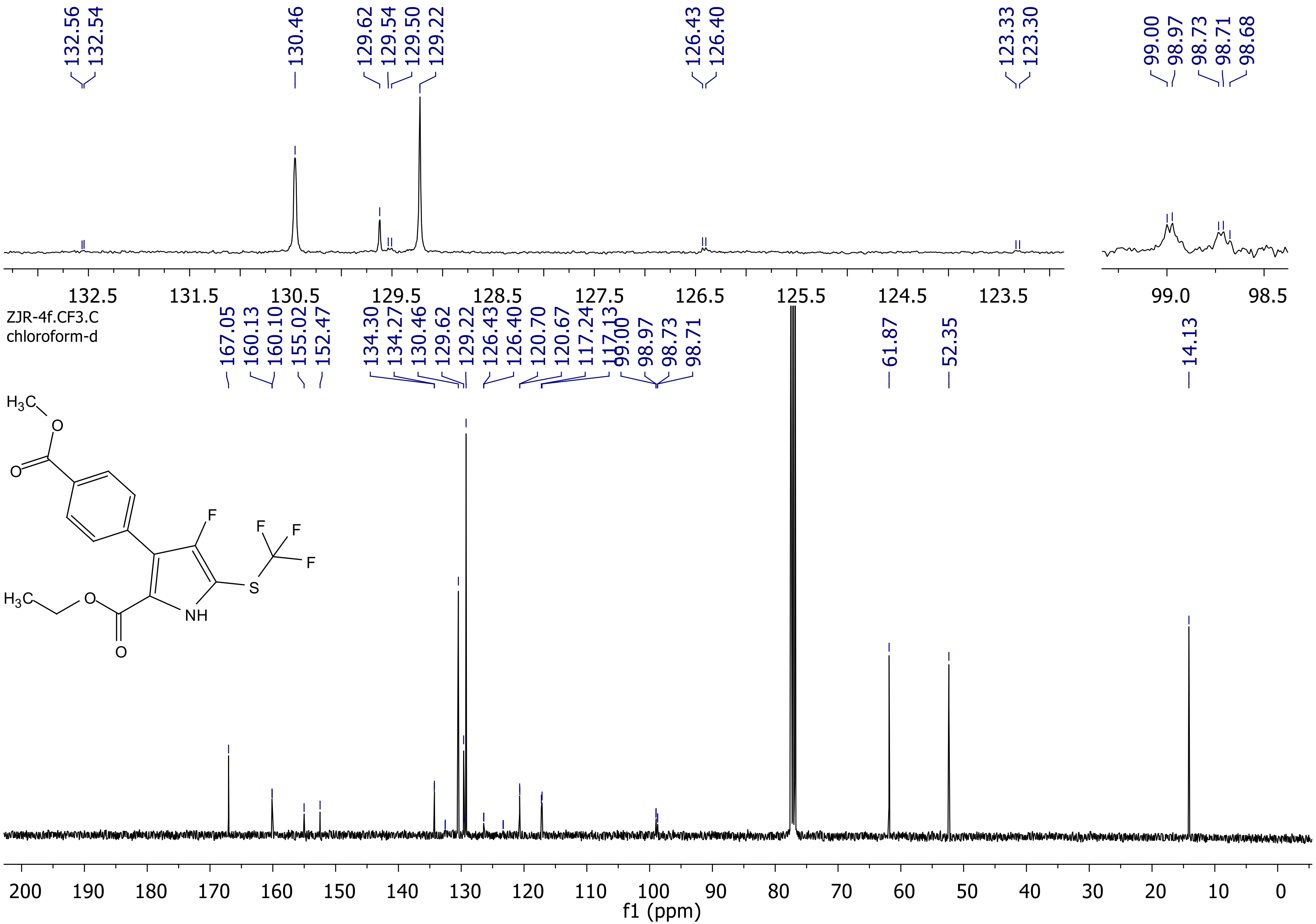

^{13}C NMR spectrum of ethyl 4-fluoro-3-(4-(methoxycarbonyl)phenyl)-5-((trifluoromethyl)thio)-1H-pyrrole-2-carboxylate (**4f**) in $CDCl_3$ at 100 MHz

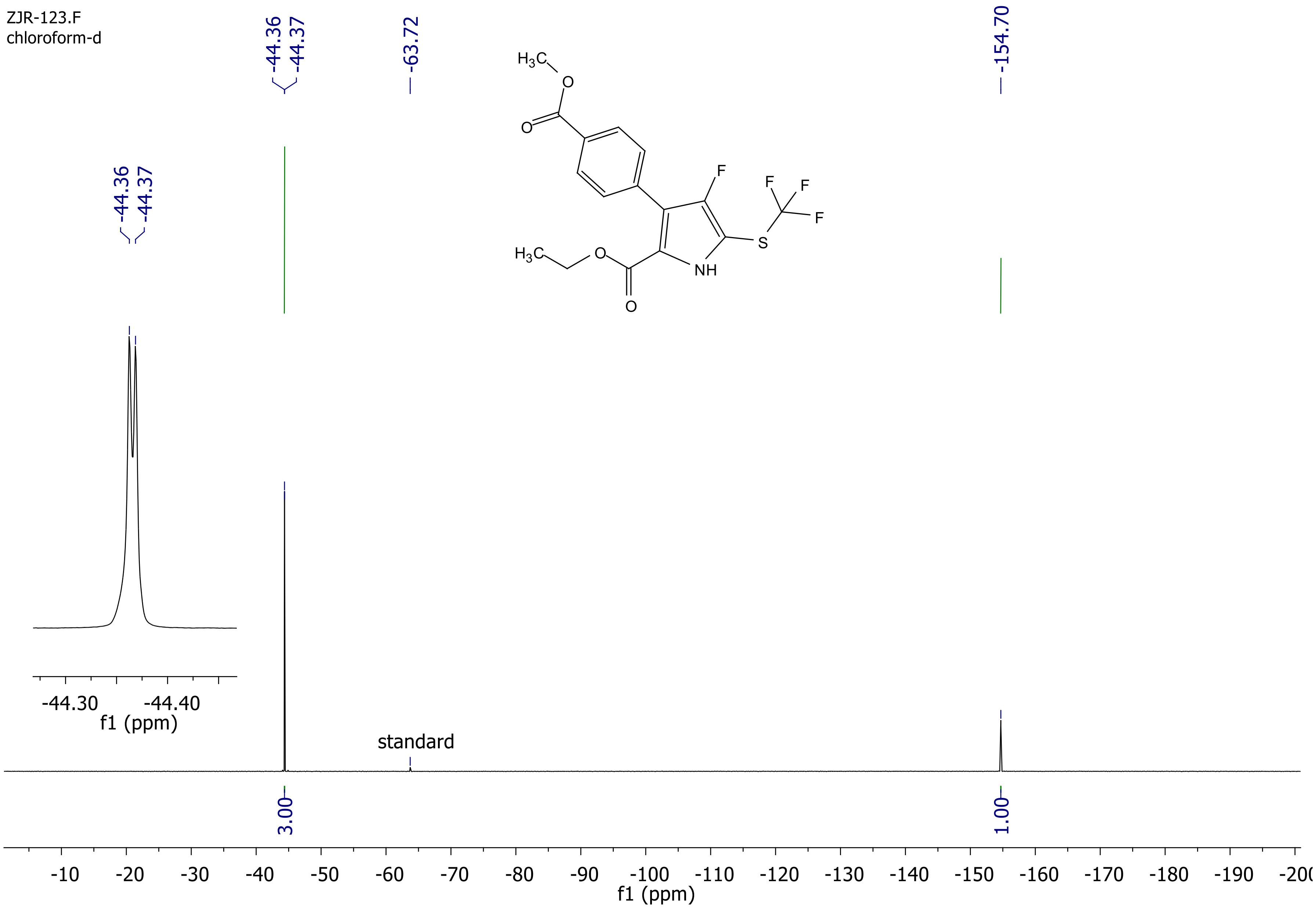

^{19}F NMR spectrum of ethyl 4-fluoro-3-(4-(methoxycarbonyl)phenyl)-5-((trifluoromethyl)thio)-1H-pyrrole-2-carboxylate (**4f**) in $CDCl_3$ at 376 MHz

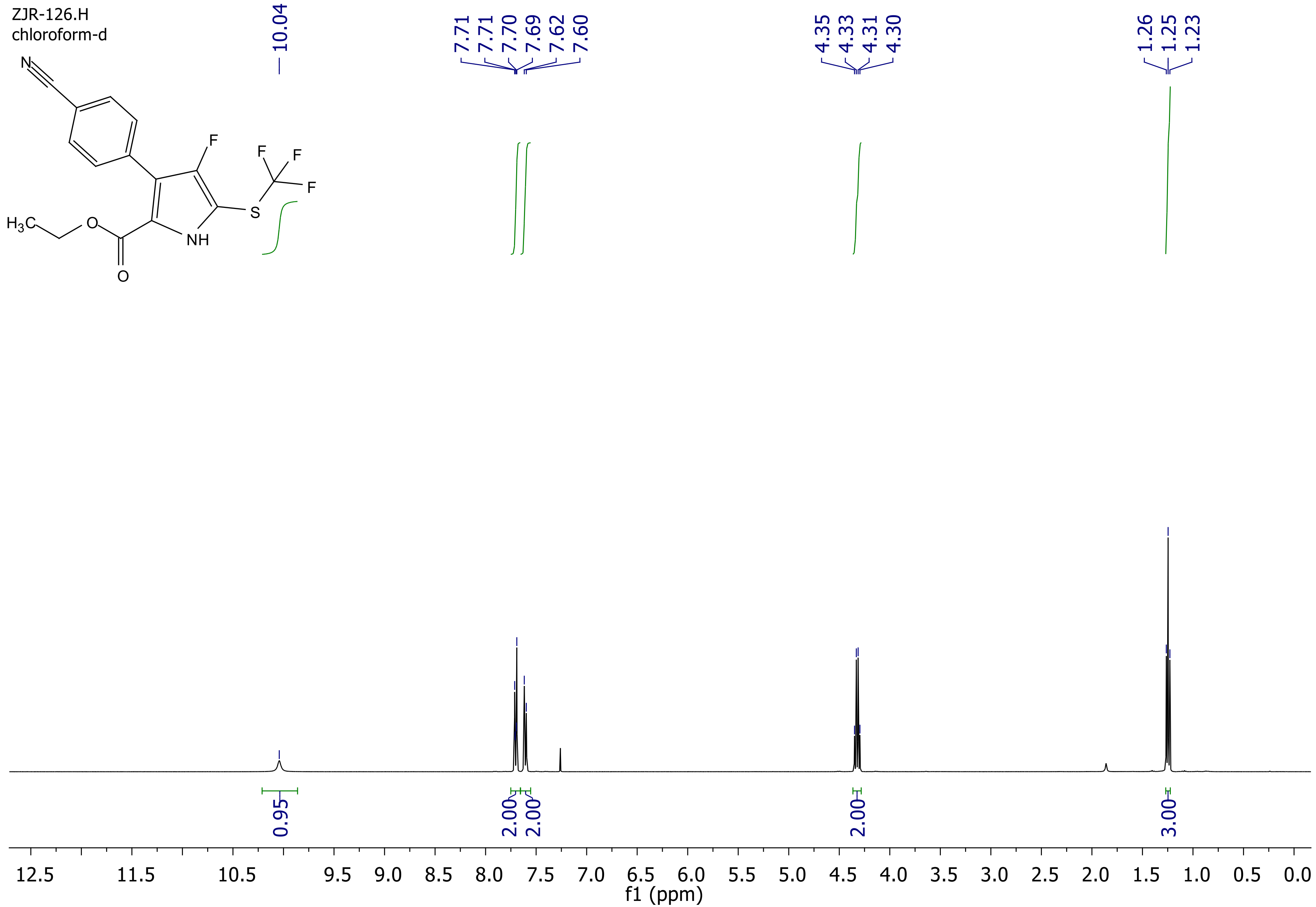

^{1}H NMR spectrum of ethyl 3-(4-cyanophenyl)-4-fluoro-5-((trifluoromethyl)thio)-1H-pyrrole-2-carboxylate (**4g**) in $CDCl_3$ at 400 MHz

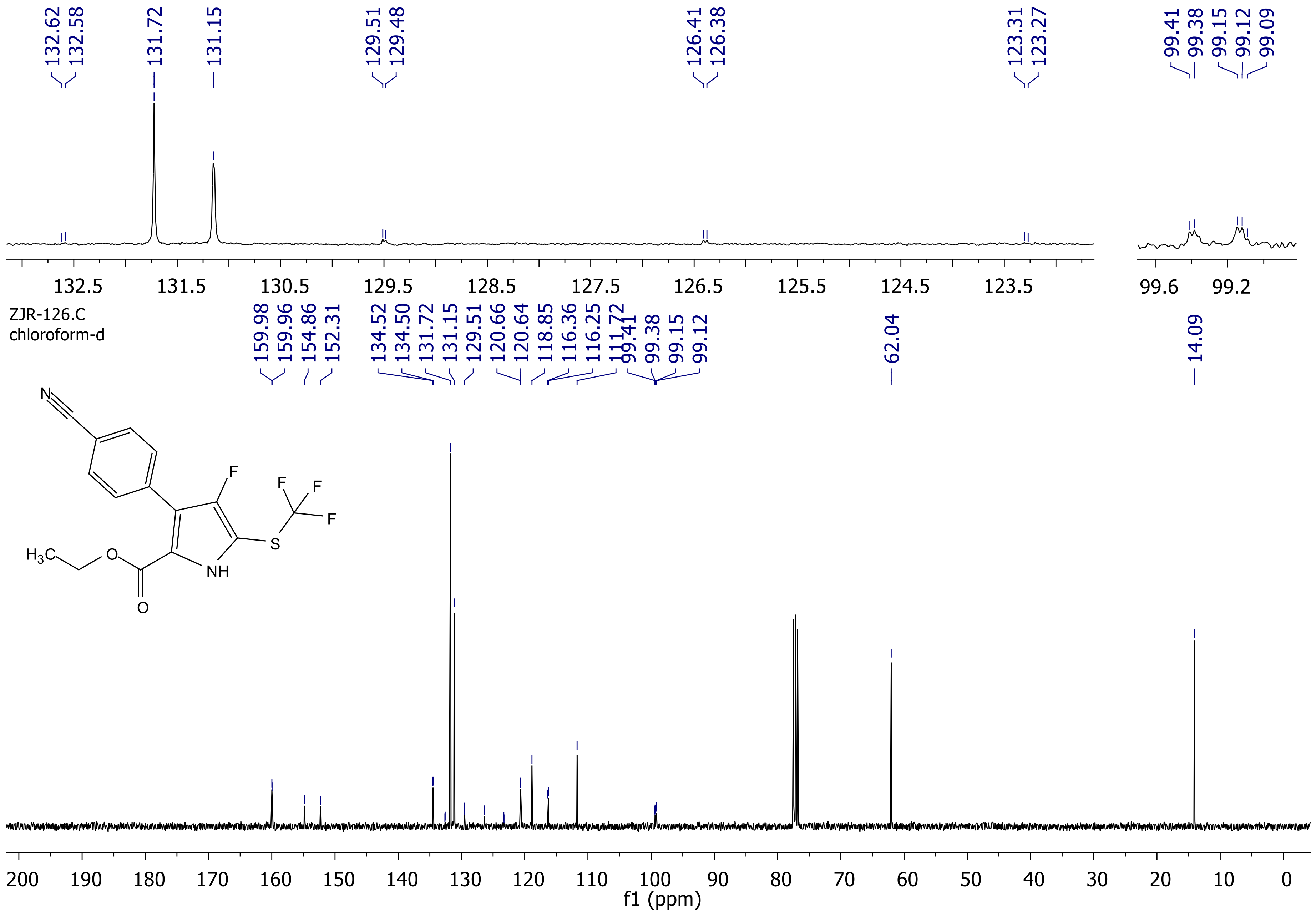

^{13}C NMR spectrum of ethyl 3-(4-cyanophenyl)-4-fluoro-5-((trifluoromethyl)thio)-1H-pyrrole-2-carboxylate (**4g**) in $CDCl_3$ at 100 MHz

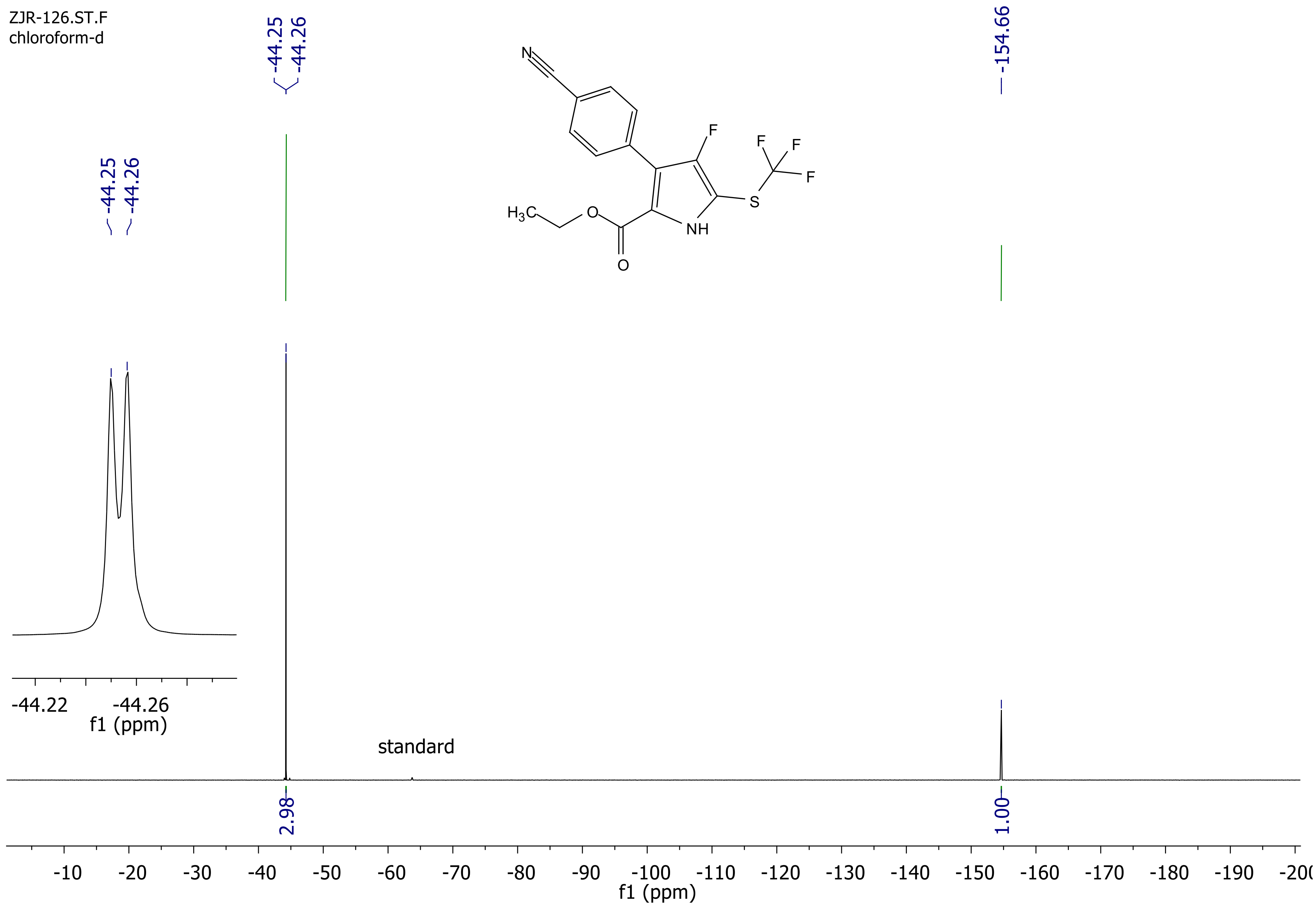

^{19}F NMR spectrum of ethyl 3-(4-cyanophenyl)-4-fluoro-5-((trifluoromethyl)thio)-1H-pyrrole-2-carboxylate (**4g**) in $CDCl_3$ at 376 MHz

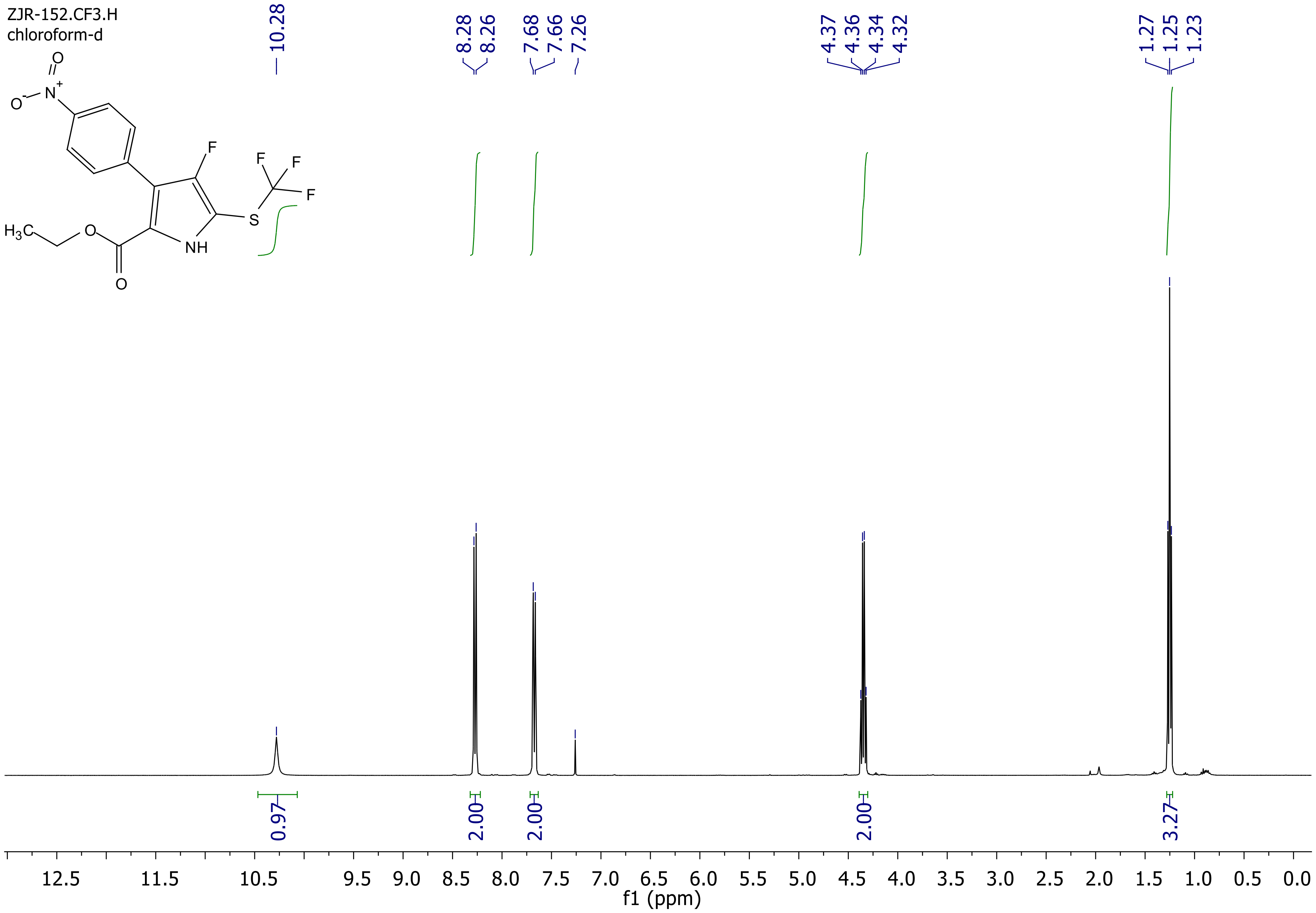

^{1}H NMR spectrum of ethyl 4-fluoro-3-(4-nitrophenyl)-5-((trifluoromethyl)thio)-1H-pyrrole-2-carboxylate (**4h**) in $CDCl_3$ at 400 MHz

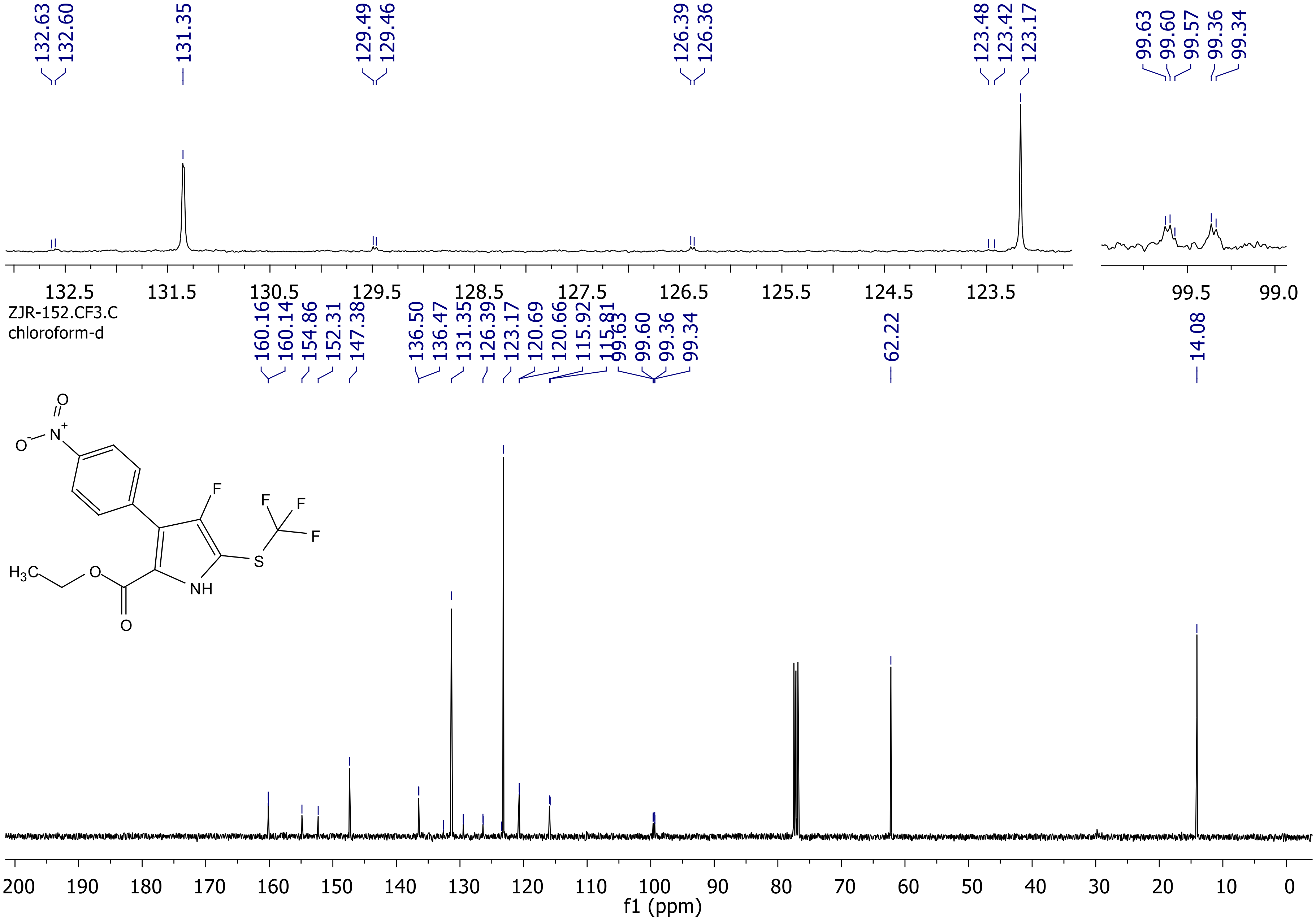

^{13}C NMR spectrum of ethyl 4-fluoro-3-(4-nitrophenyl)-5-((trifluoromethyl)thio)-1H-pyrrole-2-carboxylate (**4h**) in $CDCl_3$ at 100 MHz

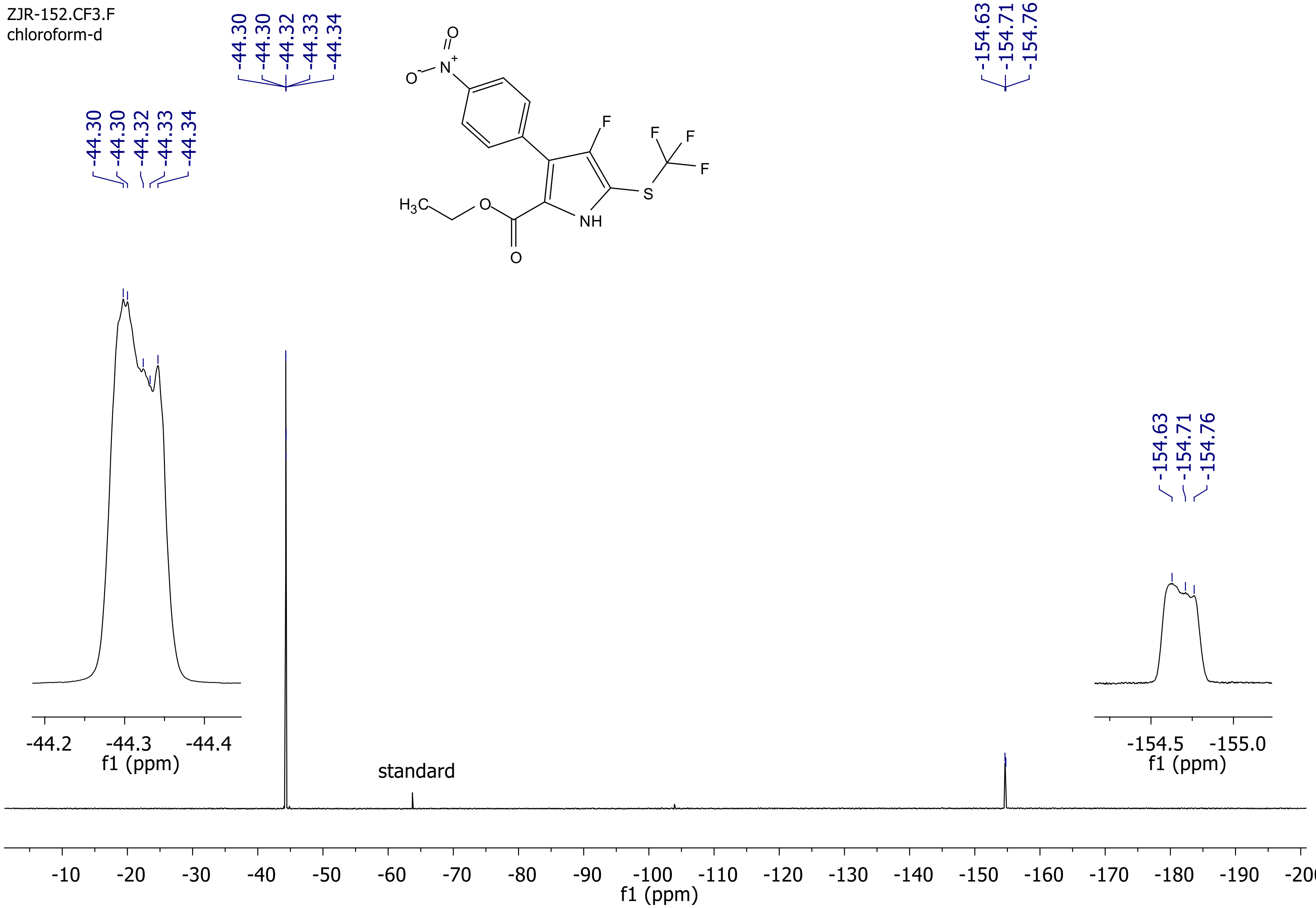

^{19}F NMR spectrum of ethyl 4-fluoro-3-(4-nitrophenyl)-5-((trifluoromethyl)thio)-1H-pyrrole-2-carboxylate (**4h**) in $CDCl_3$ at 376 MHz

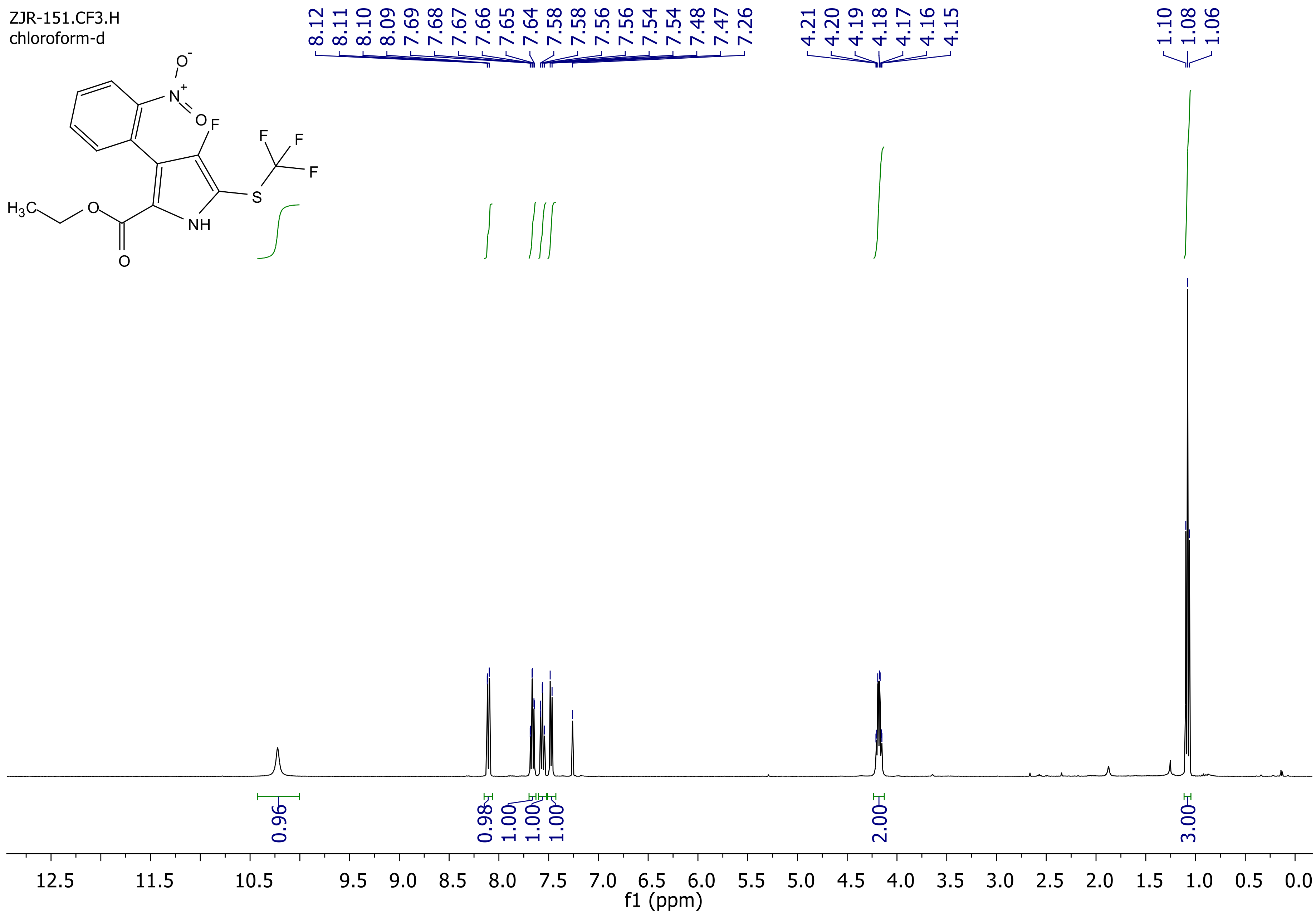

^{1}H NMR spectrum of ethyl 4-fluoro-3-(2-nitrophenyl)-5-((trifluoromethyl)thio)-1H-pyrrole-2-carboxylate (**4i**) in $CDCl_3$ at 400 MHz

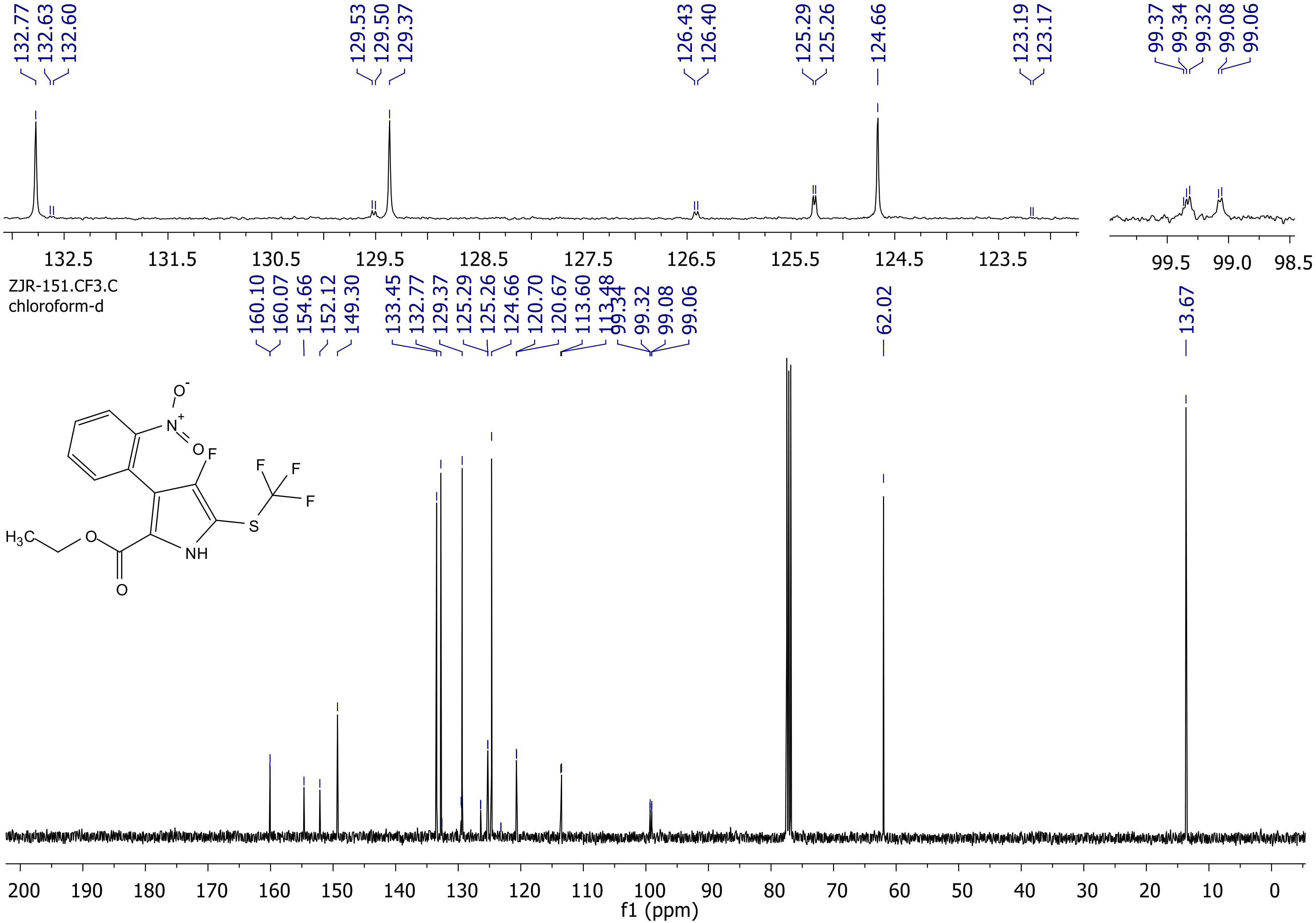

^{13}C NMR spectrum of ethyl 4-fluoro-3-(2-nitrophenyl)-5-((trifluoromethyl)thio)-1H-pyrrole-2-carboxylate (**4i**) in $CDCl_3$ at 100 MHz

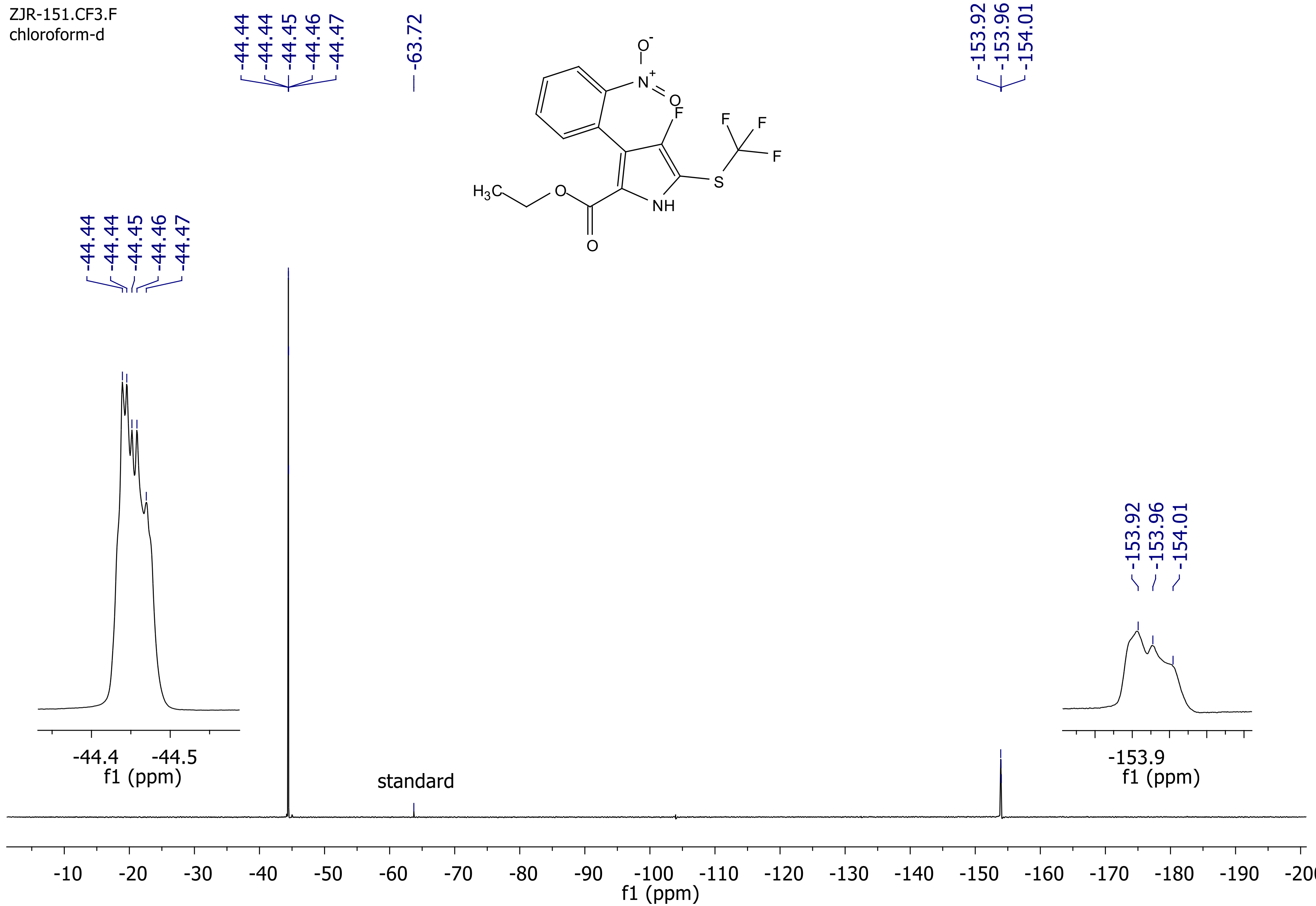

^{19}F NMR spectrum of ethyl 4-fluoro-3-(2-nitrophenyl)-5-((trifluoromethyl)thio)-1H-pyrrole-2-carboxylate (**4i**) in $CDCl_3$ at 376 MHz

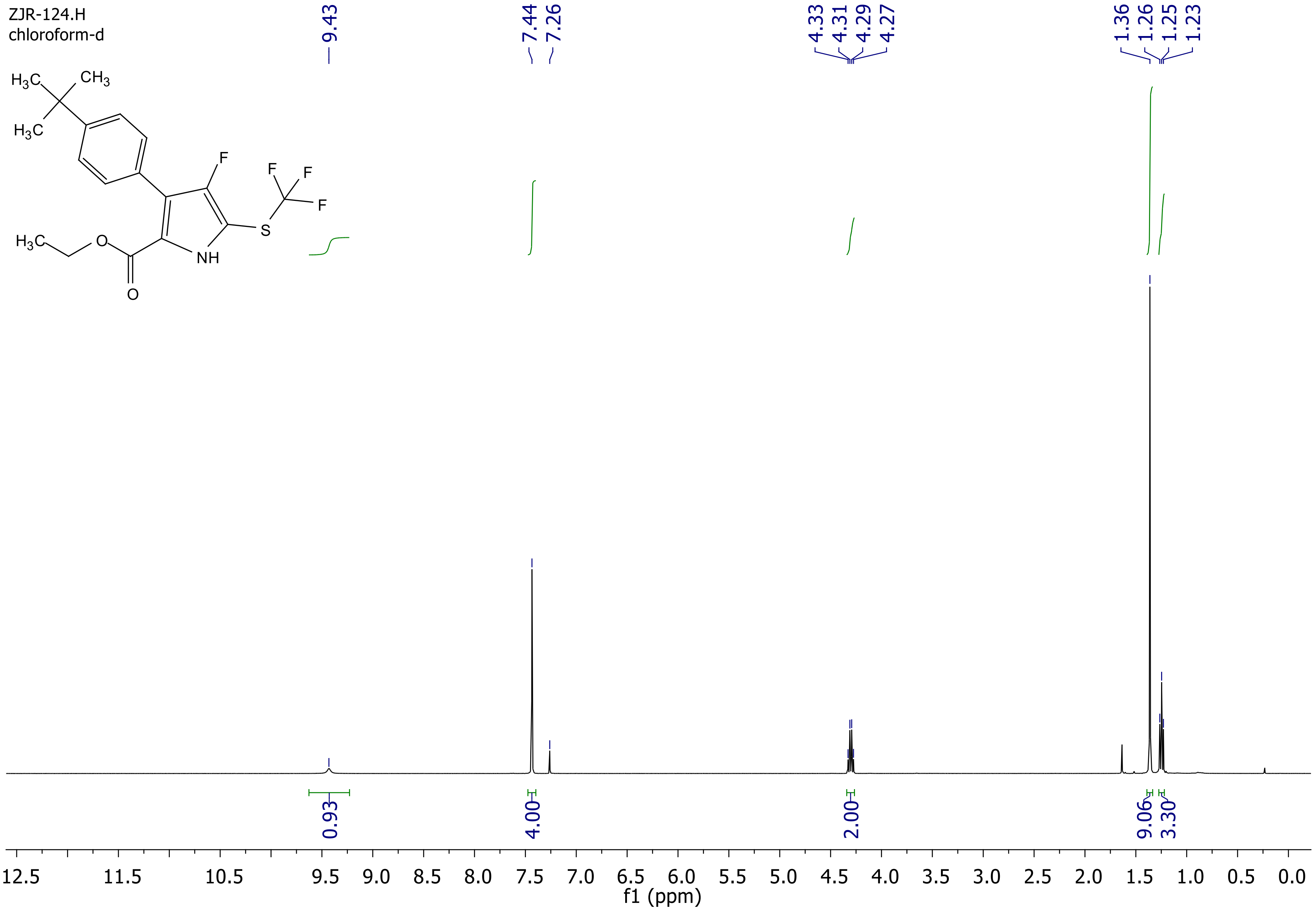

^{1}H NMR spectrum of ethyl 3-(4-(tert-butyl)phenyl)-4-fluoro-5-((trifluoromethyl)thio)-1H-pyrrole-2-carboxylate (**4j**) in $CDCl_3$ at 400 MHz

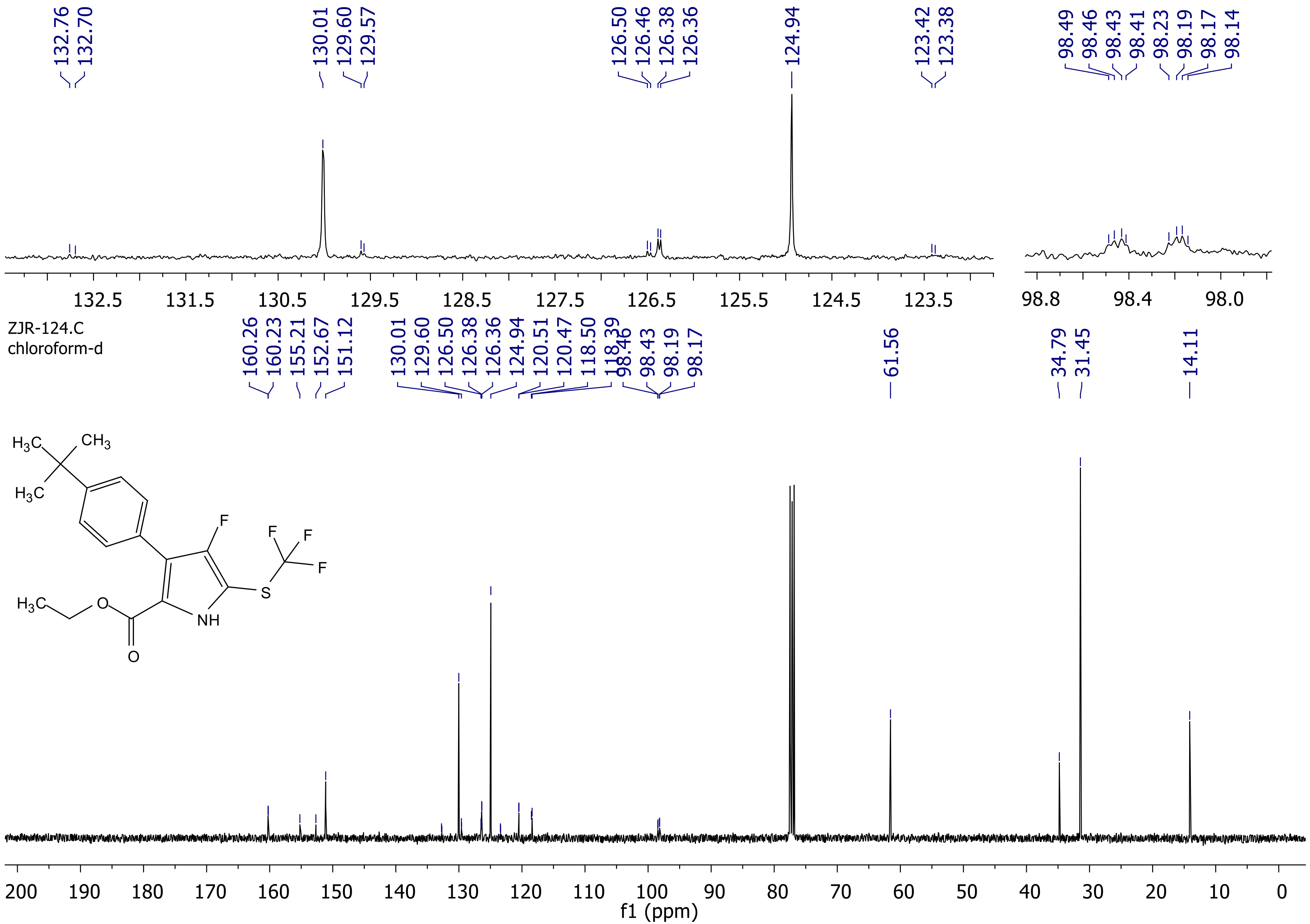

^{13}C NMR spectrum of ethyl 3-(4-(tert-butyl)phenyl)-4-fluoro-5-((trifluoromethyl)thio)-1H-pyrrole-2-carboxylate (**4j**) in $CDCl_3$ at 100 MHz

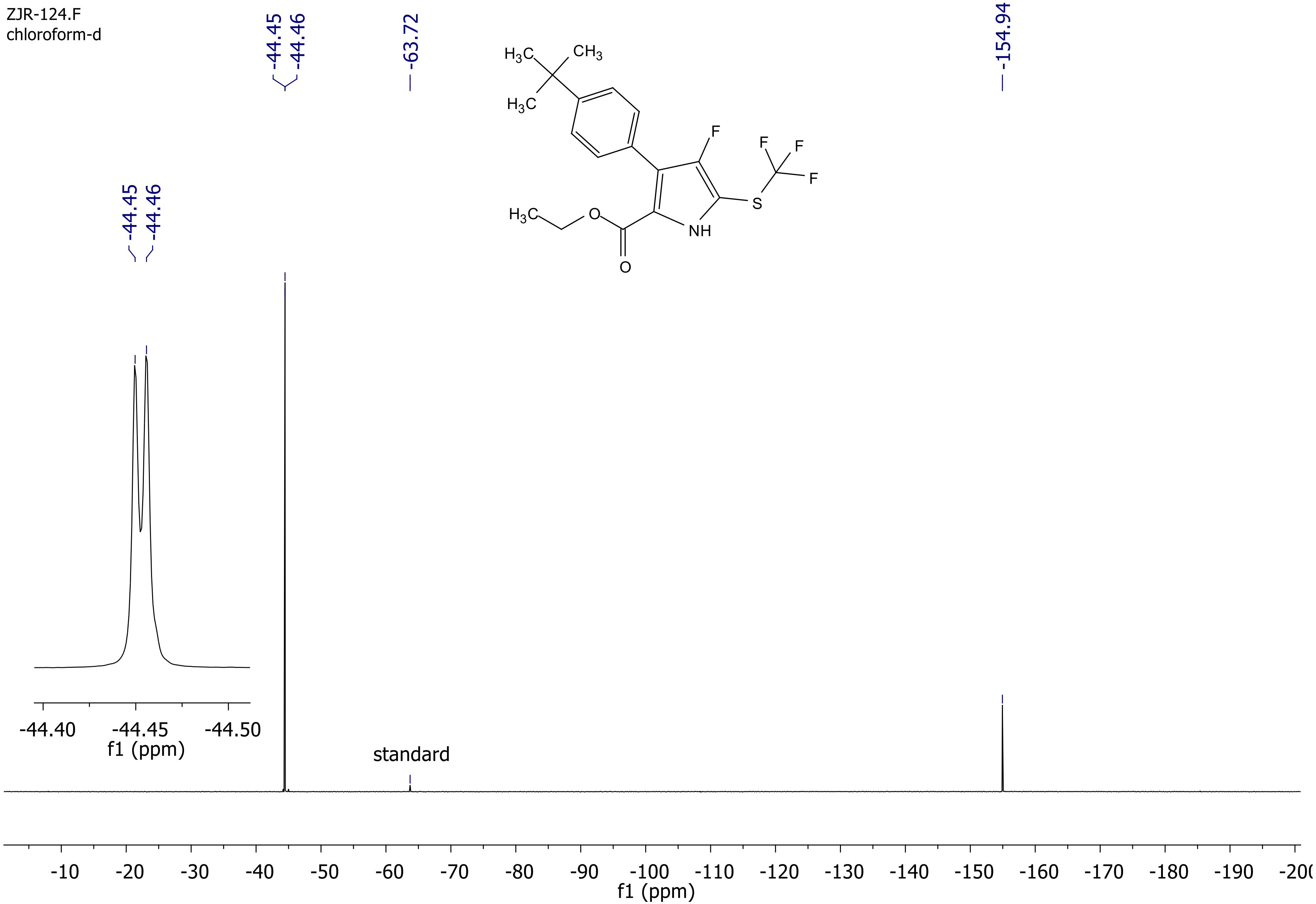

^{19}F NMR spectrum of ethyl 3-(4-(tert-butyl)phenyl)-4-fluoro-5-((trifluoromethyl)thio)-1H-pyrrole-2-carboxylate (**4j**) in $CDCl_3$ at 376 MHz

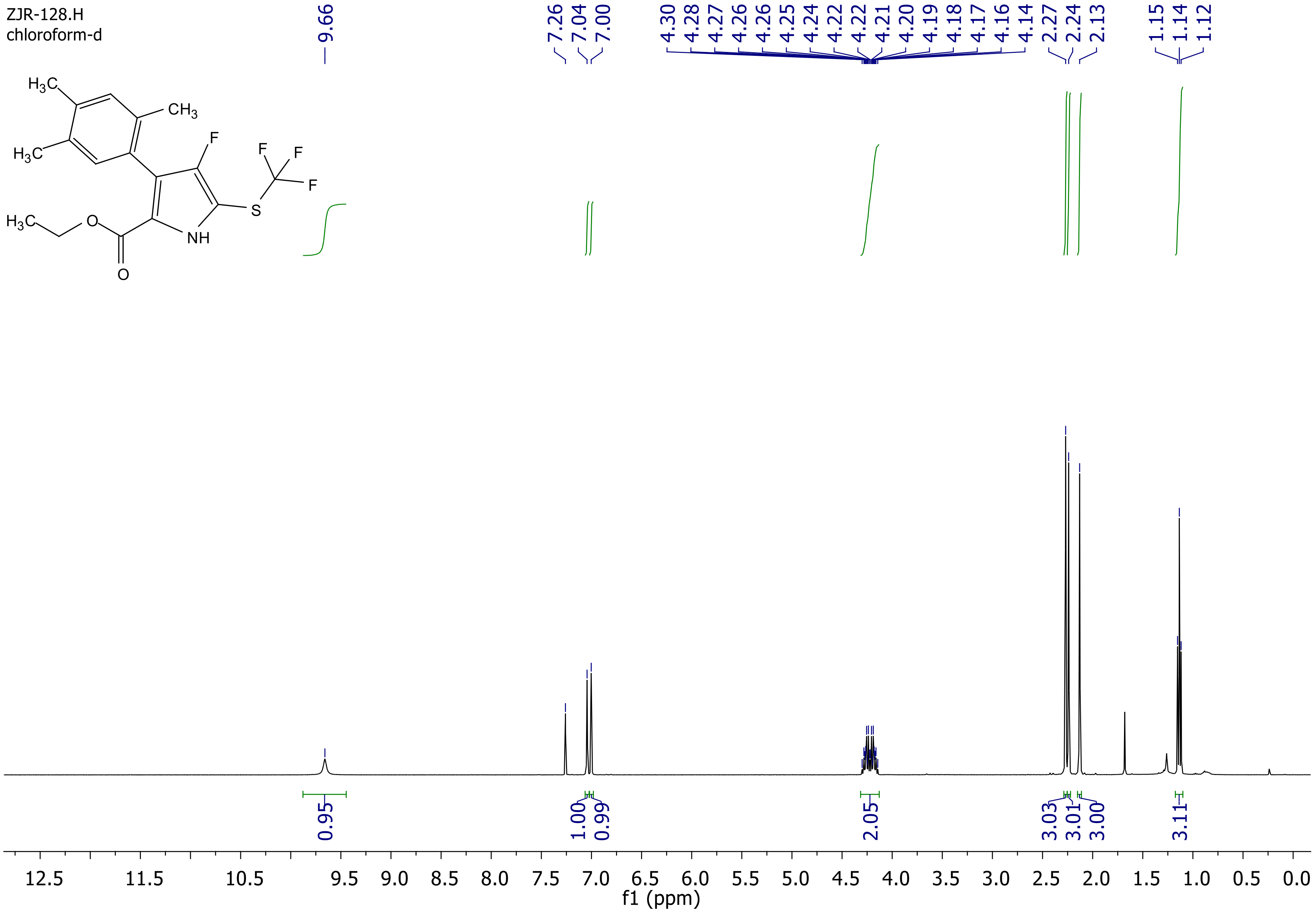

^{1}H NMR spectrum of ethyl 4-fluoro-5-((trifluoromethyl)thio)-3-(2,4,5-trimethylphenyl)-1H-pyrrole-2-carboxylate (**4k**) in $CDCl_3$ at 400 MHz

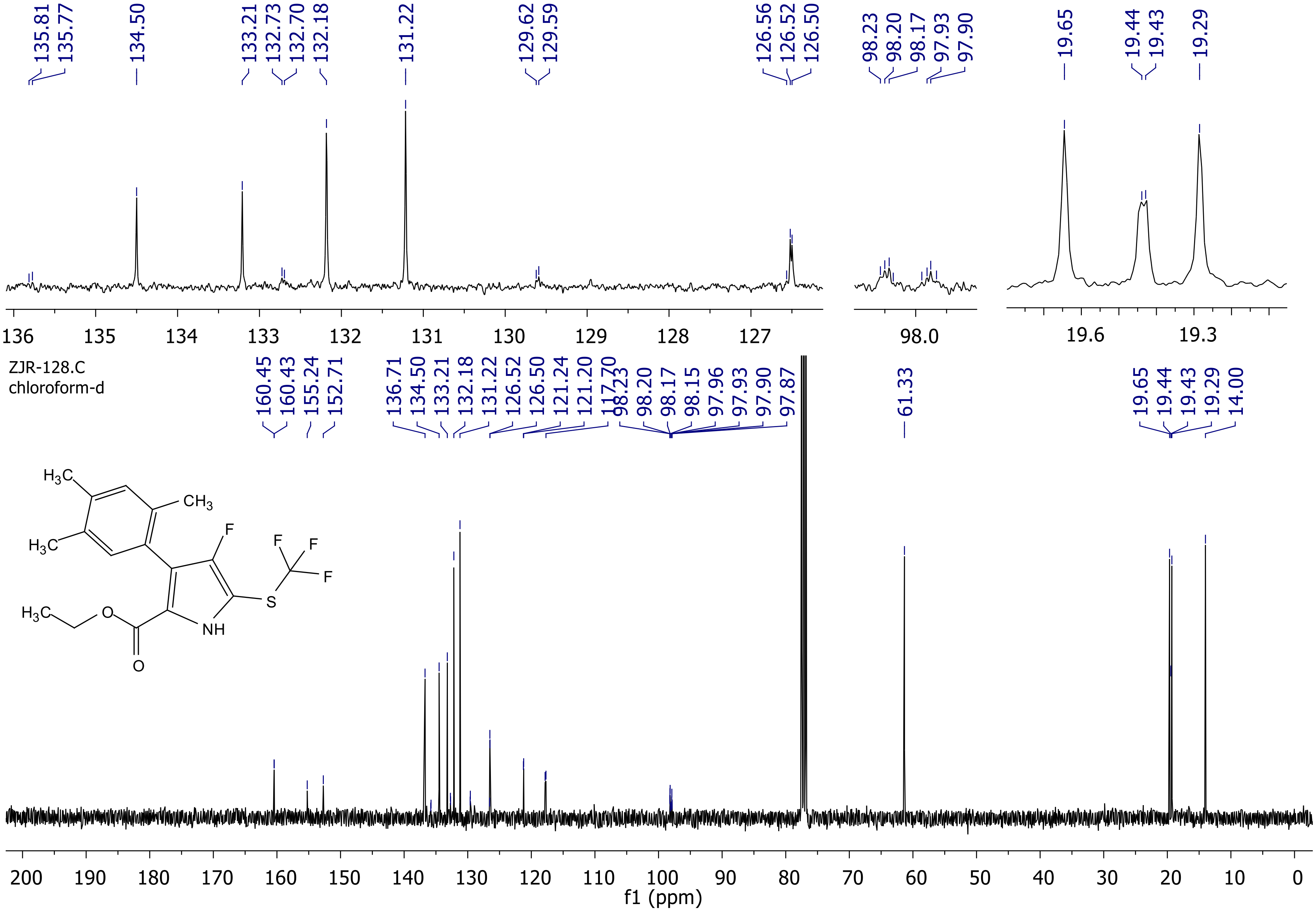

^{13}C NMR spectrum of ethyl 4-fluoro-5-((trifluoromethyl)thio)-3-(2,4,5-trimethylphenyl)-1H-pyrrole-2-carboxylate (**4k**) in $CDCl_3$ at 100 MHz

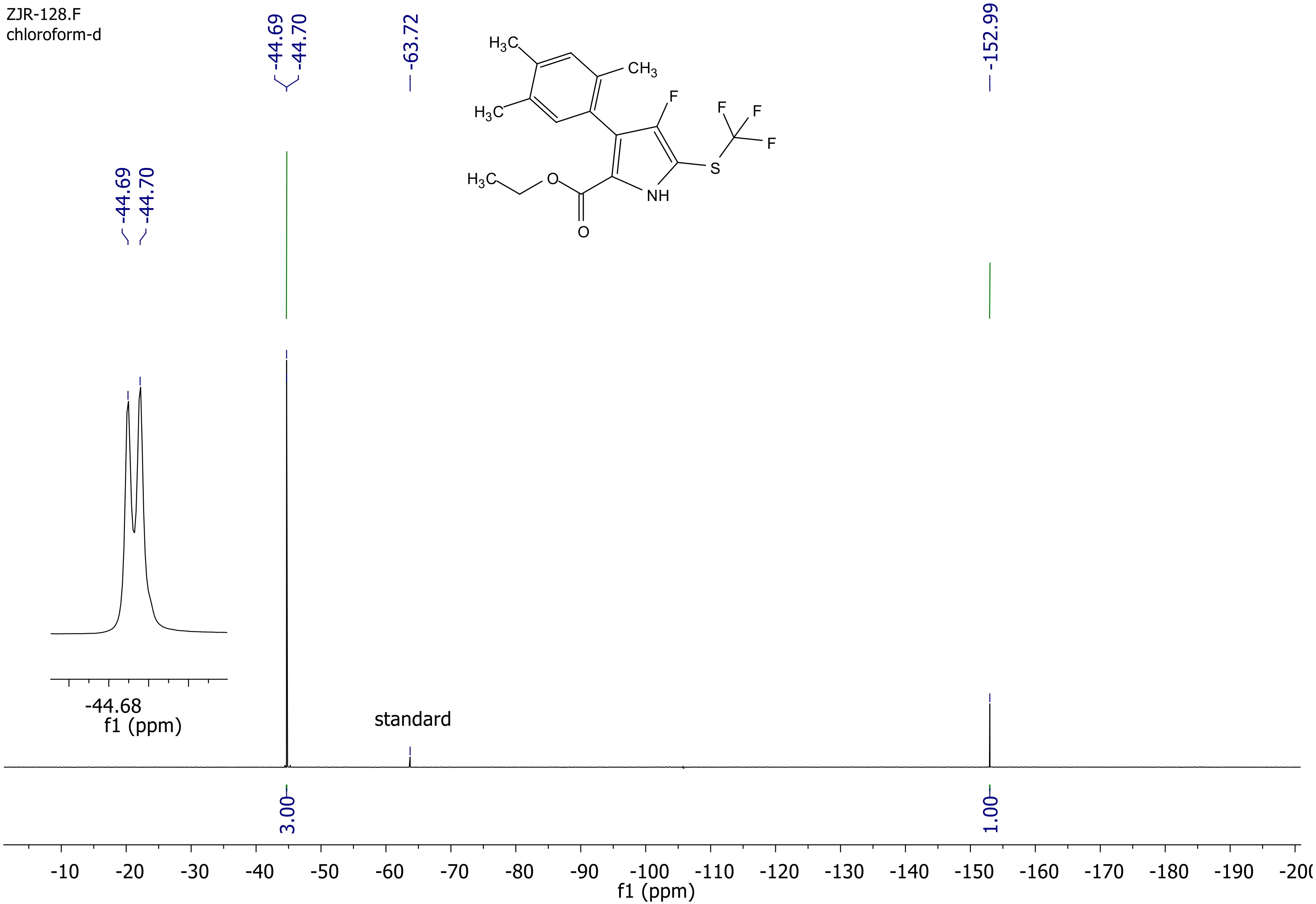

^{19}F NMR spectrum of ethyl 4-fluoro-5-((trifluoromethyl)thio)-3-(2,4,5-trimethylphenyl)-1H-pyrrole-2-carboxylate (**4k**) in $CDCl_3$ at 376 MHz

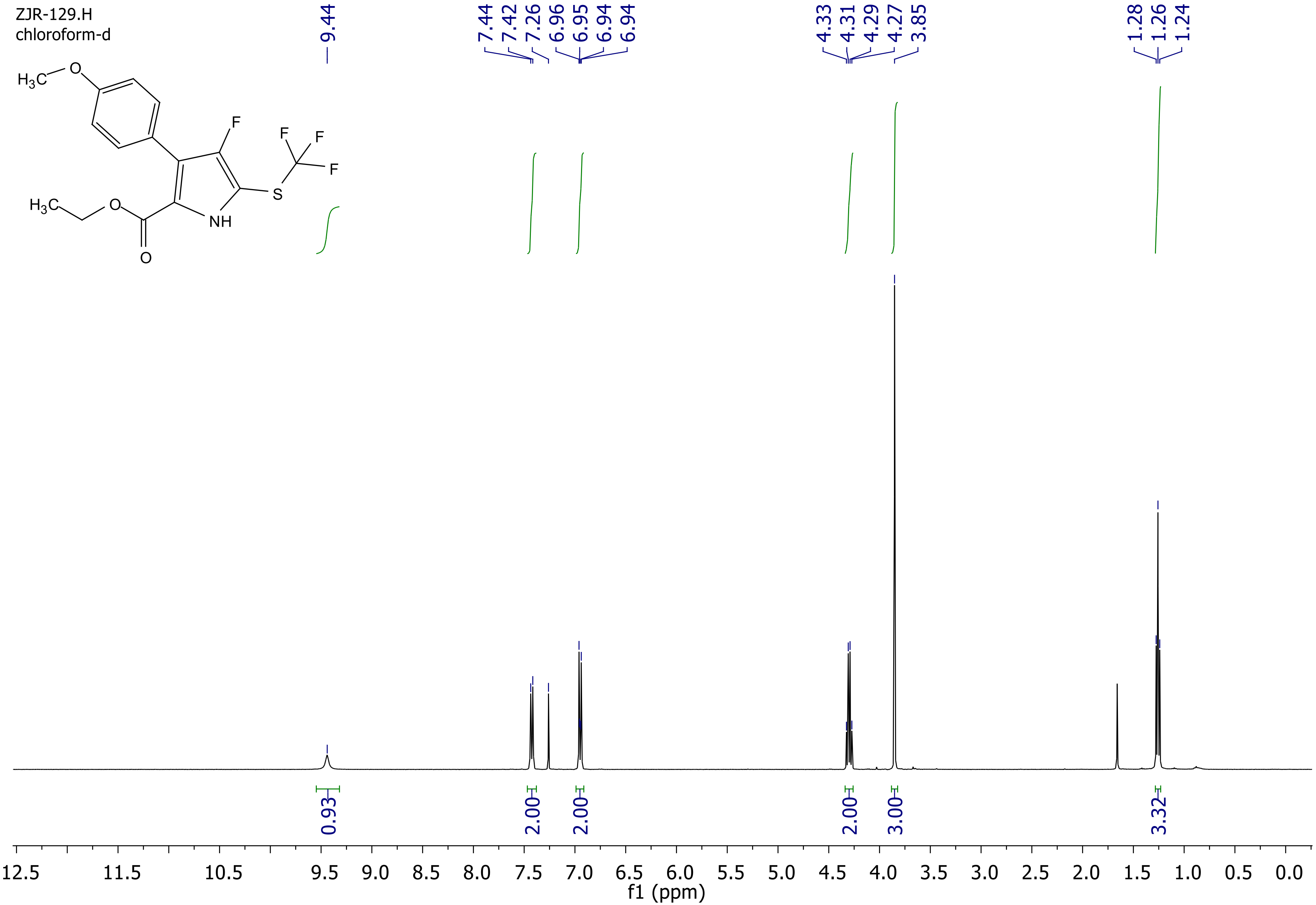

^1H NMR spectrum of ethyl 4-fluoro-3-(4-methoxyphenyl)-5-((trifluoromethyl)thio)-1H-pyrrole-2-carboxylate (**4l**) in $CDCl_3$ at 400 MHz

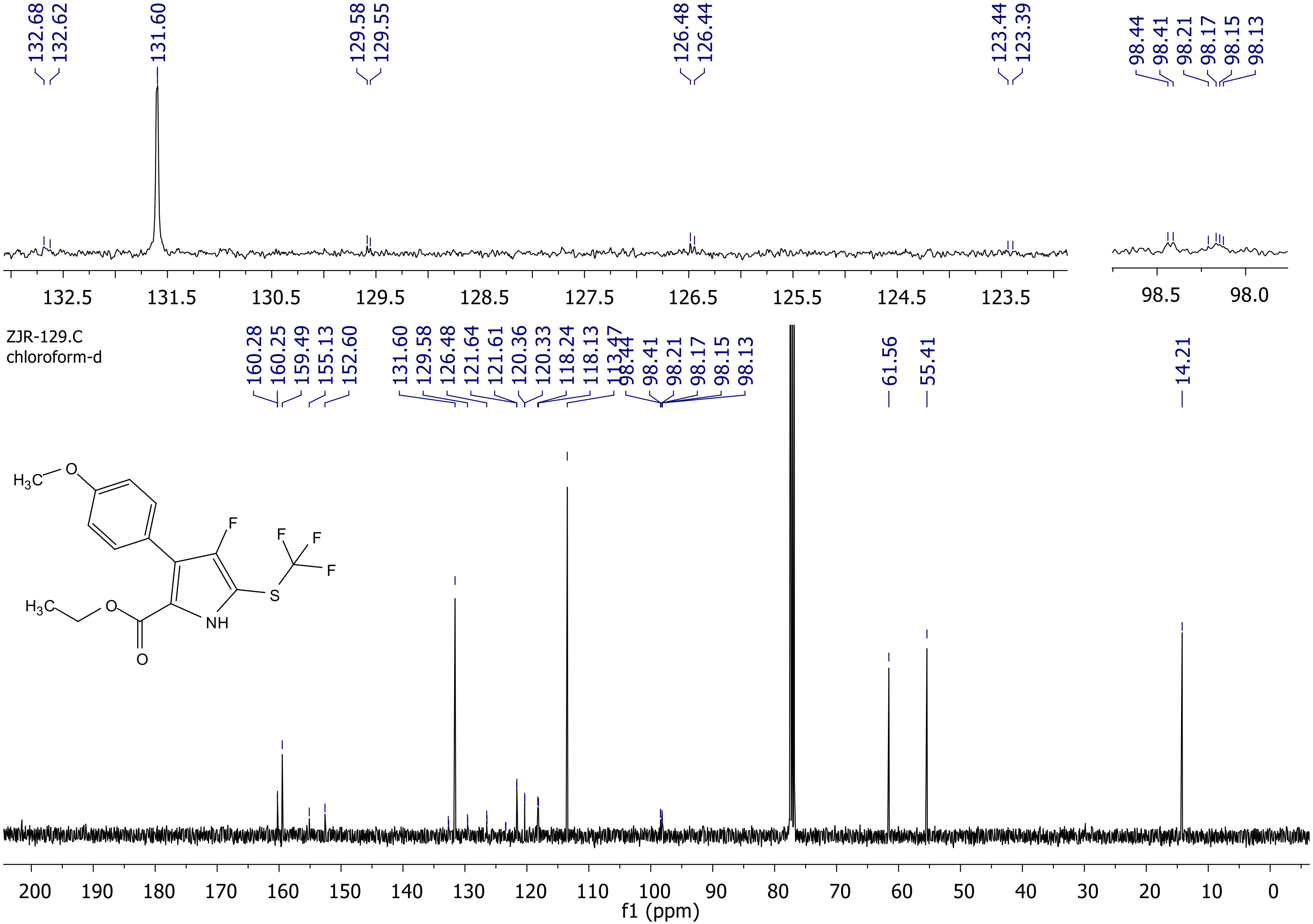

^{13}C NMR spectrum of ethyl 4-fluoro-3-(4-methoxyphenyl)-5-((trifluoromethyl)thio)-1H-pyrrole-2-carboxylate (**4l**) in $CDCl_3$ at 100 MHz

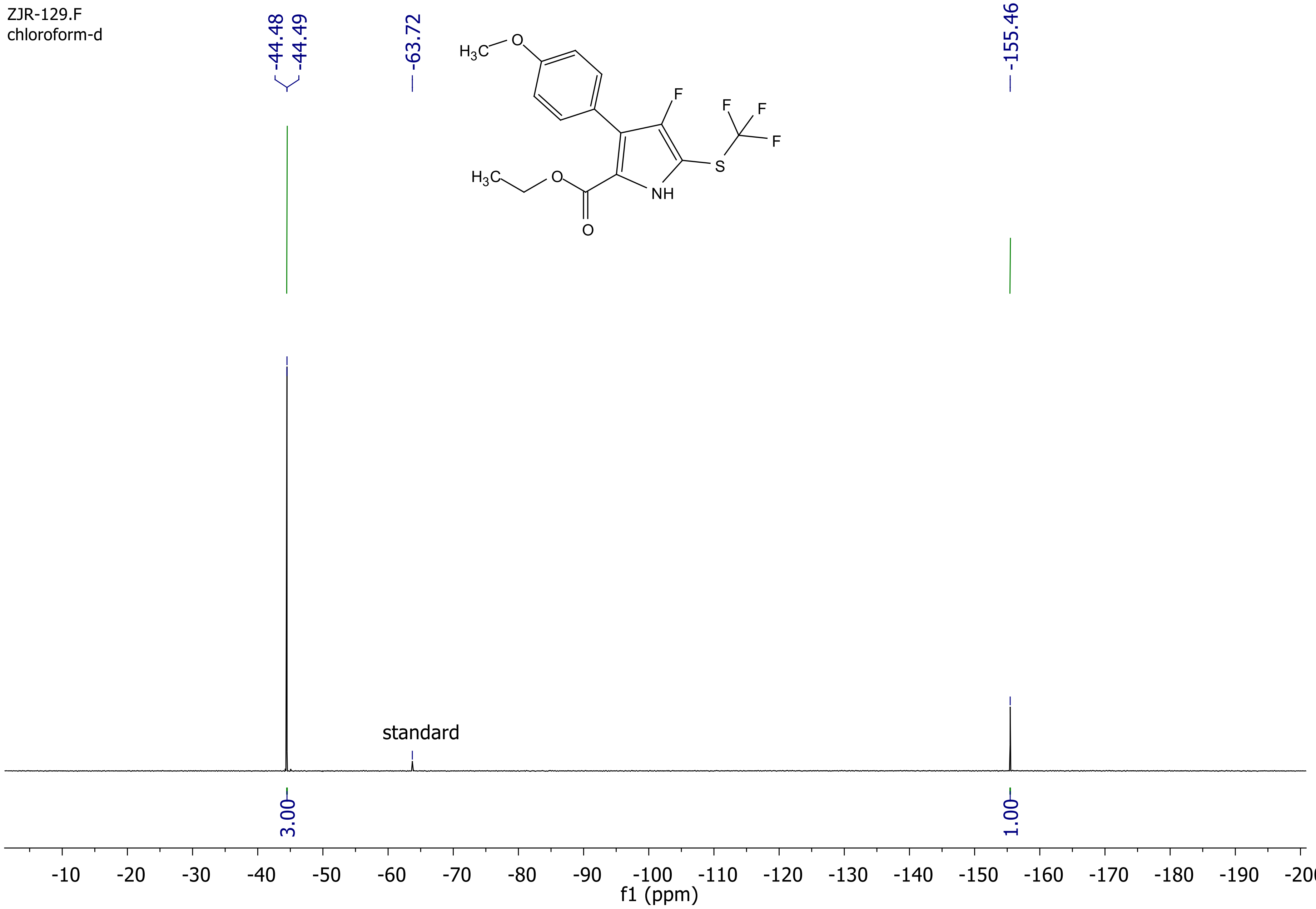

^{19}F NMR spectrum of ethyl 4-fluoro-3-(4-methoxyphenyl)-5-((trifluoromethyl)thio)-1H-pyrrole-2-carboxylate (**4l**) in $CDCl_3$ at 376 MHz

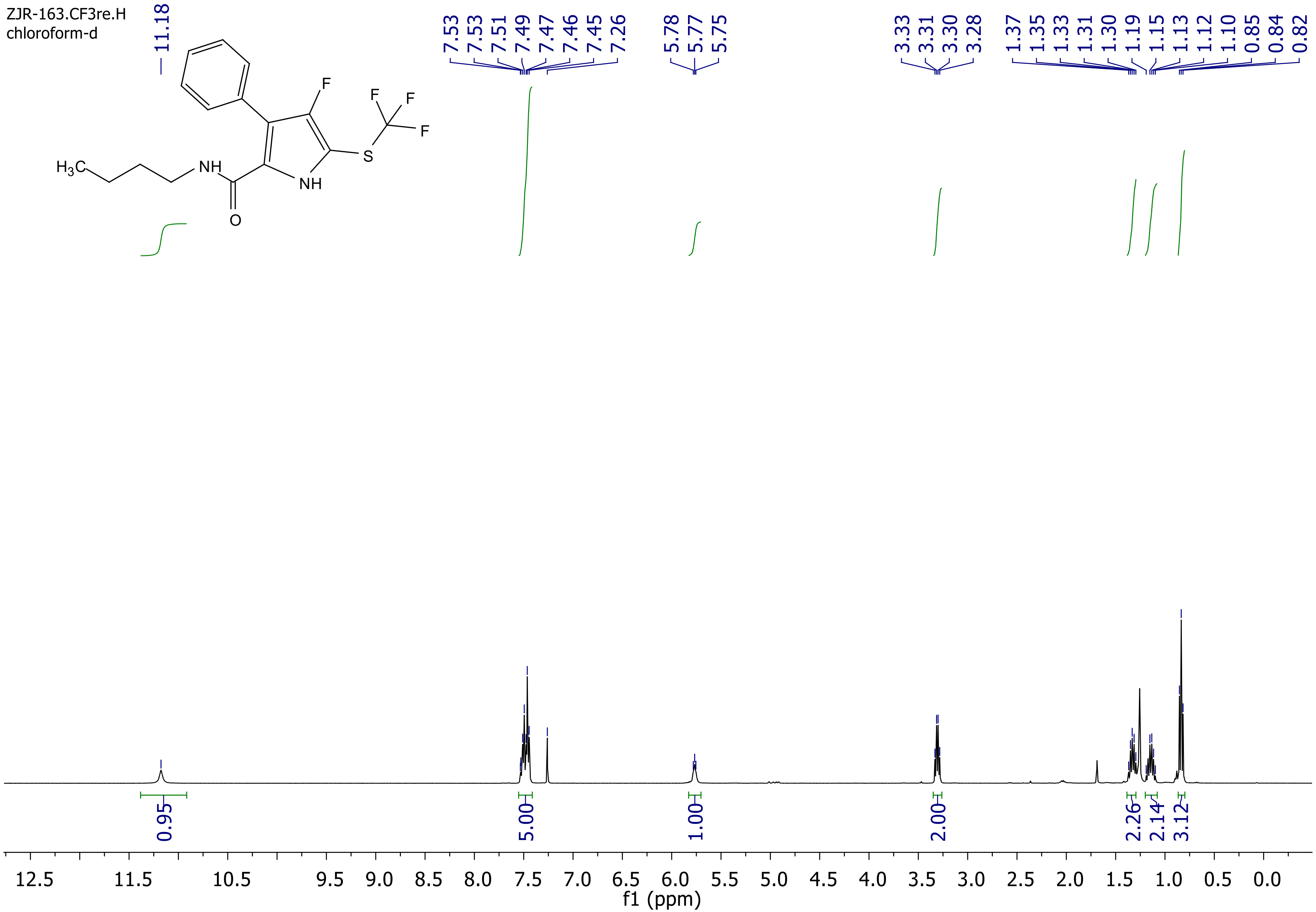

^{1}H NMR spectrum of N-butyl-4-fluoro-3-phenyl-5-((trifluoromethyl)thio)-1H-pyrrole-2-carboxamide (**4m**) in $CDCl_3$ at 400 MHz

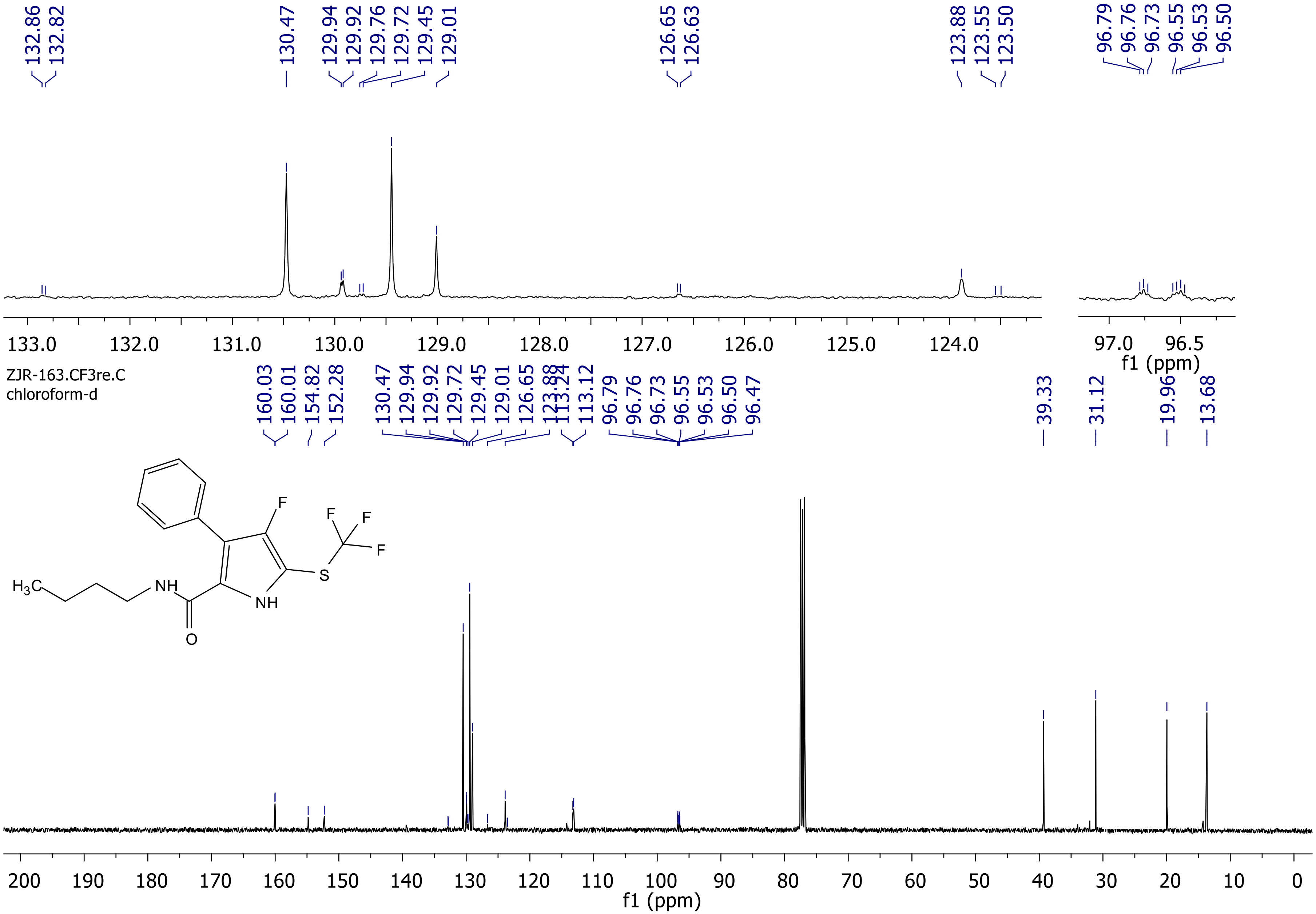

^{13}C NMR spectrum of N-butyl-4-fluoro-3-phenyl-5-((trifluoromethyl)thio)-1H-pyrrole-2-carboxamide (**4m**) in $CDCl_3$ at 100 MHz

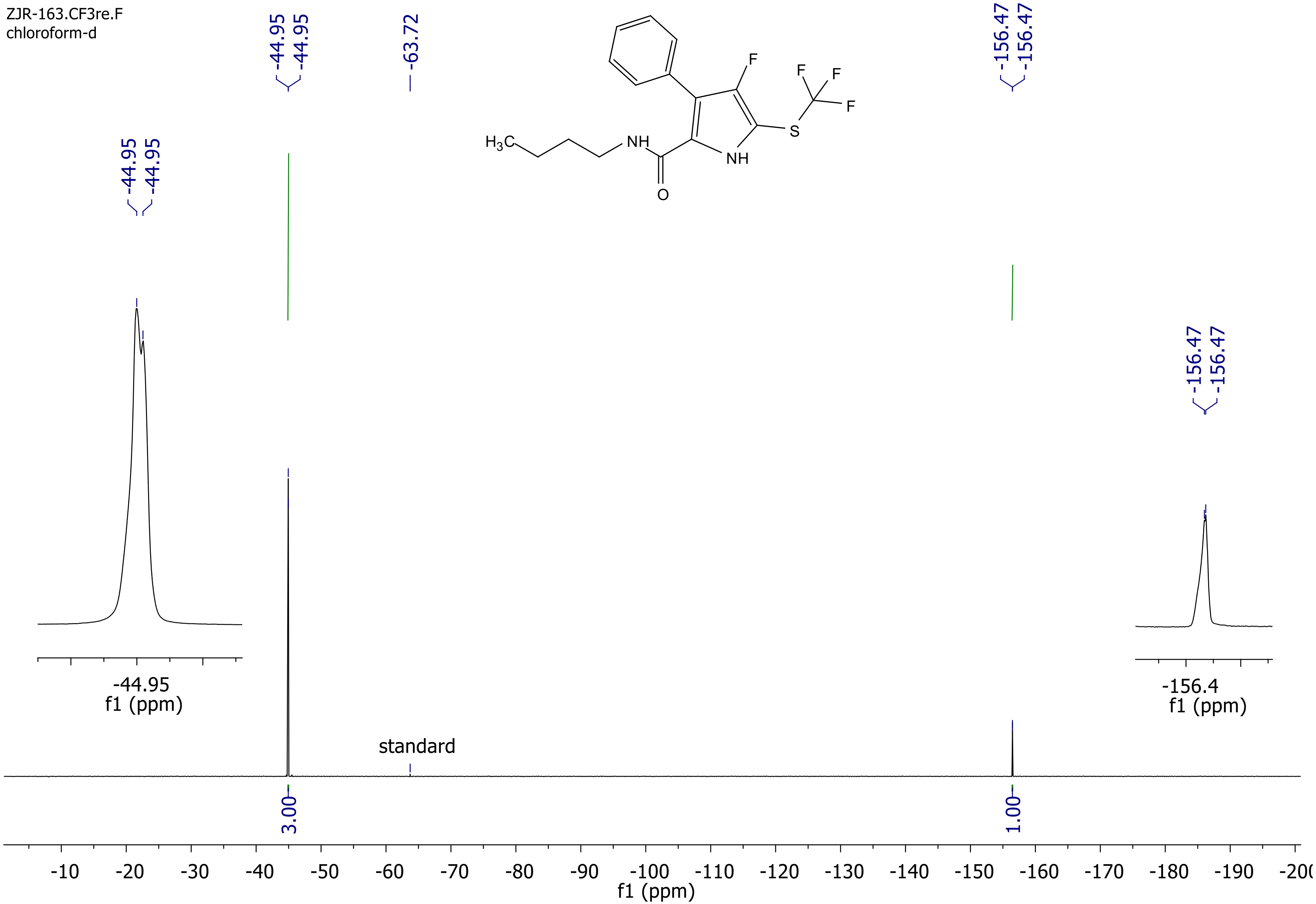

^{19}F NMR spectrum of N-butyl-4-fluoro-3-phenyl-5-((trifluoromethyl)thio)-1H-pyrrole-2-carboxamide (**4m**) in $CDCl_3$ at 376 MHz

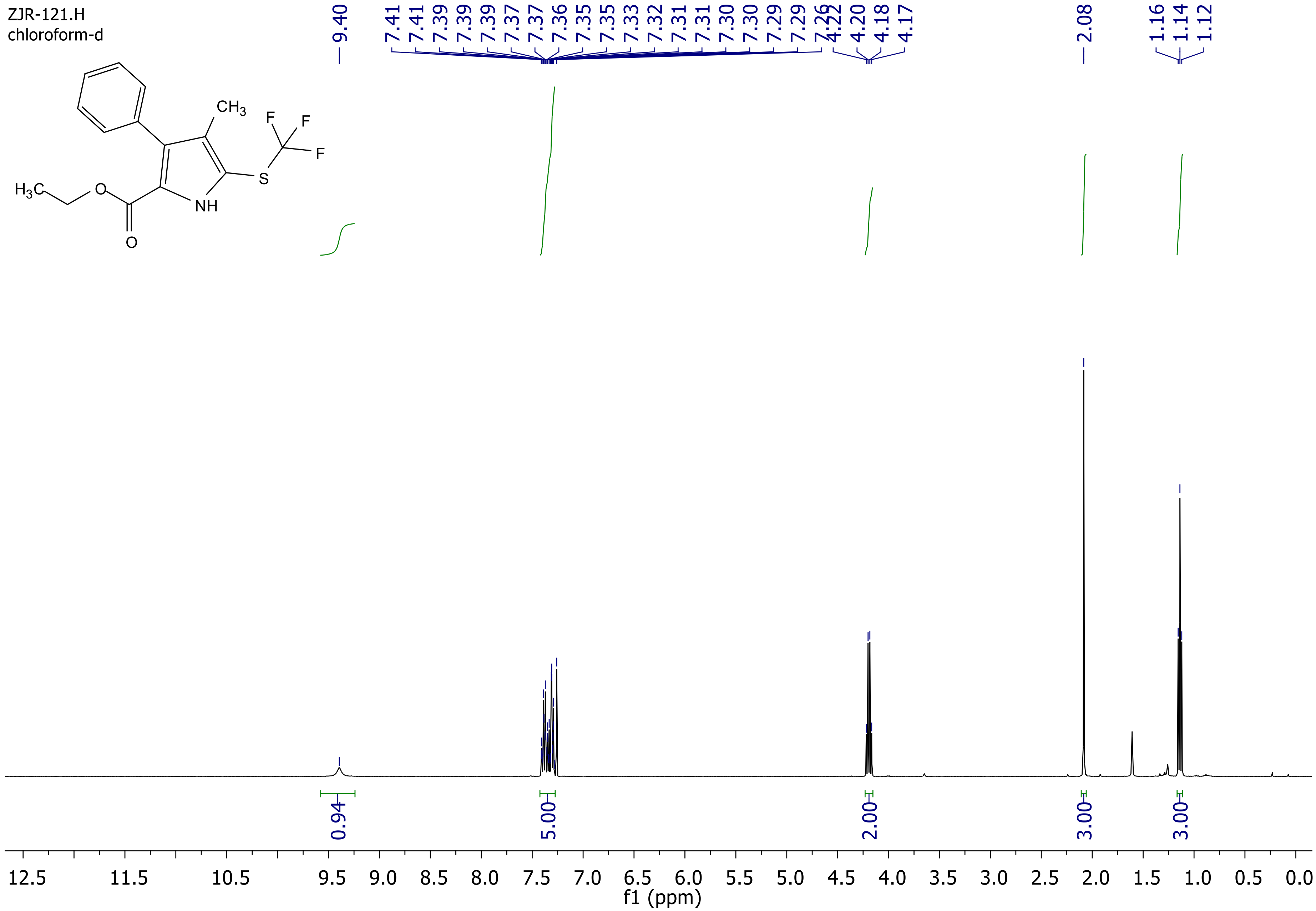

^{1}H NMR spectrum of ethyl 4-methyl-3-phenyl-5-((trifluoromethyl)thio)-1H-pyrrole-2-carboxylate (**4n**) in $CDCl_3$ at 400 MHz

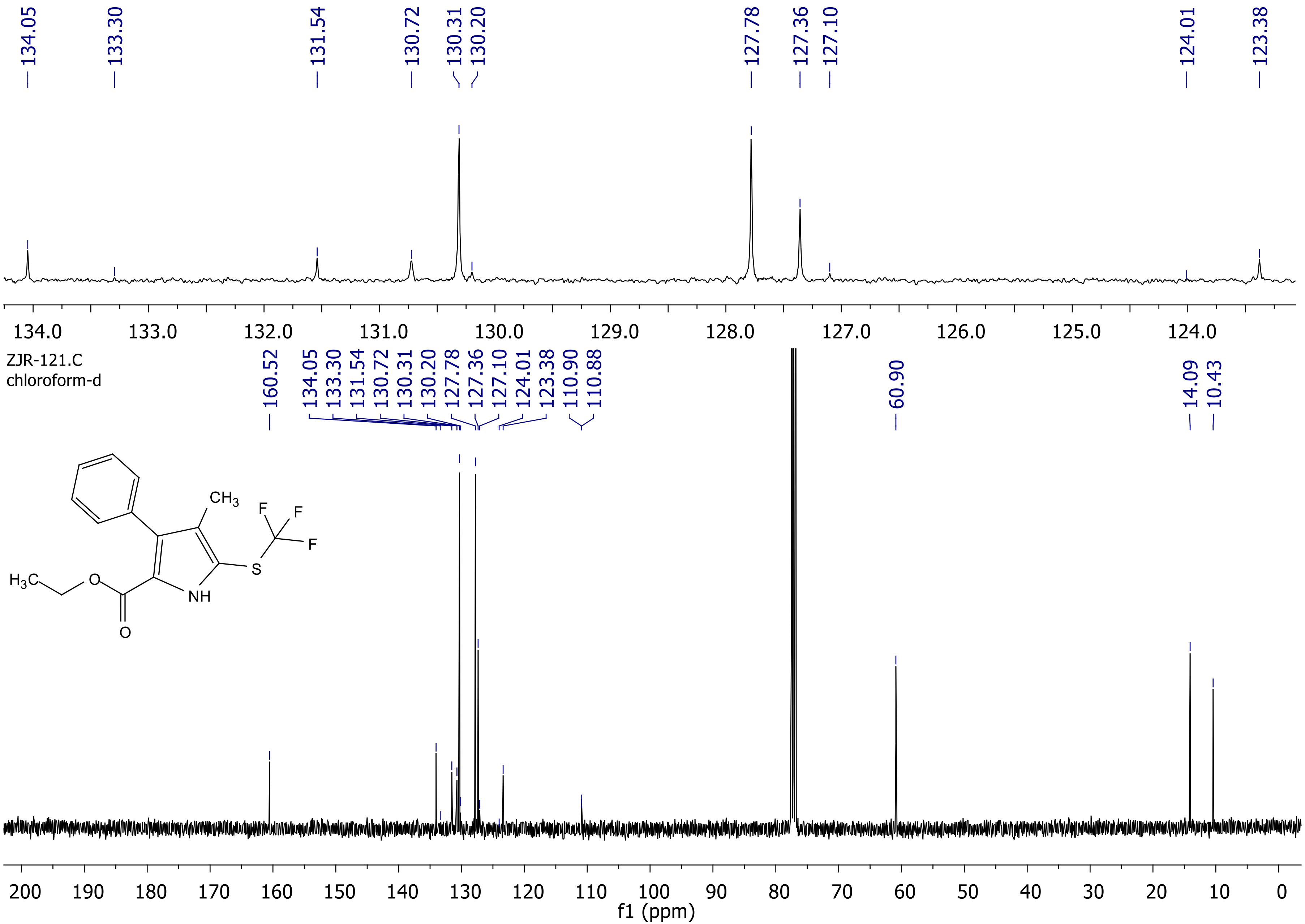

^{19}F NMR spectrum of ethyl 4-methyl-3-phenyl-5-((trifluoromethyl)thio)-1H-pyrrole-2-carboxylate (**4n**) in $CDCl_3$ at 100 MHz

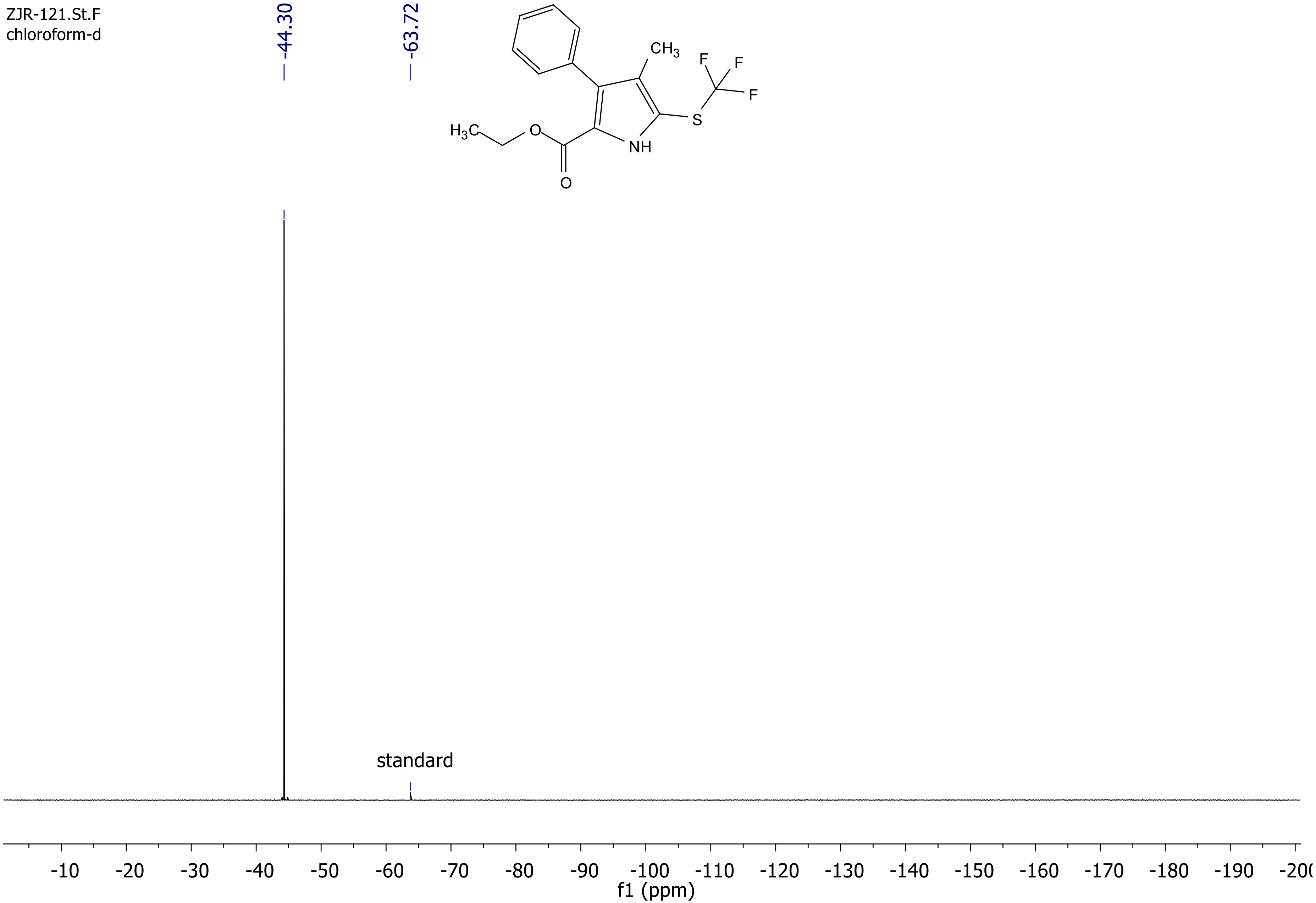

^{19}F NMR spectrum of ethyl 4-methyl-3-phenyl-5-((trifluoromethyl)thio)-1H-pyrrole-2-carboxylate (**4n**) in $CDCl_3$ at 376 MHz

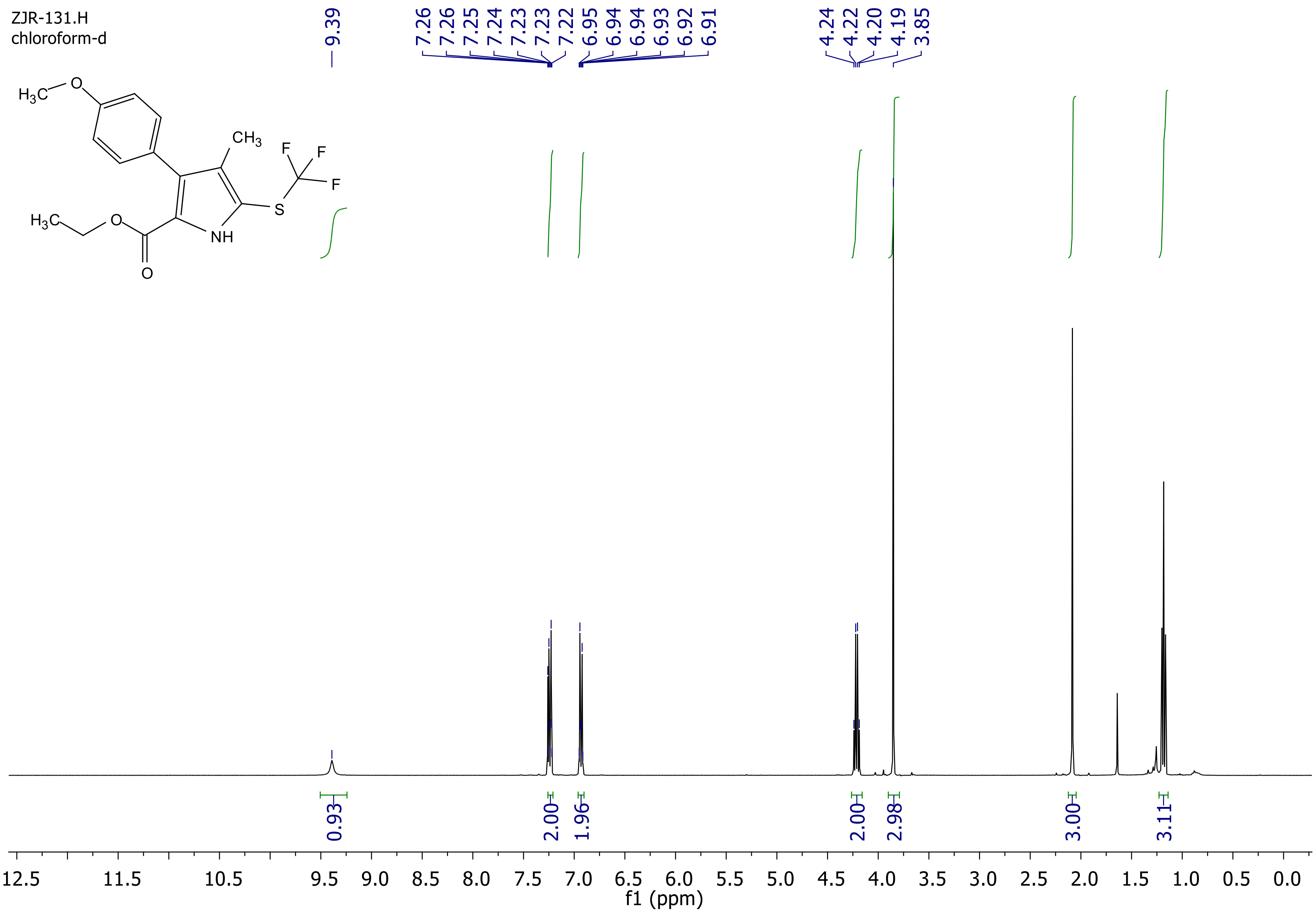

^{1}H NMR spectrum of ethyl 3-(4-methoxyphenyl)-4-methyl-5-((trifluoromethyl)thio)-1H-pyrrole-2-carboxylate (**4o**) in $CDCl_3$ at 400 MHz

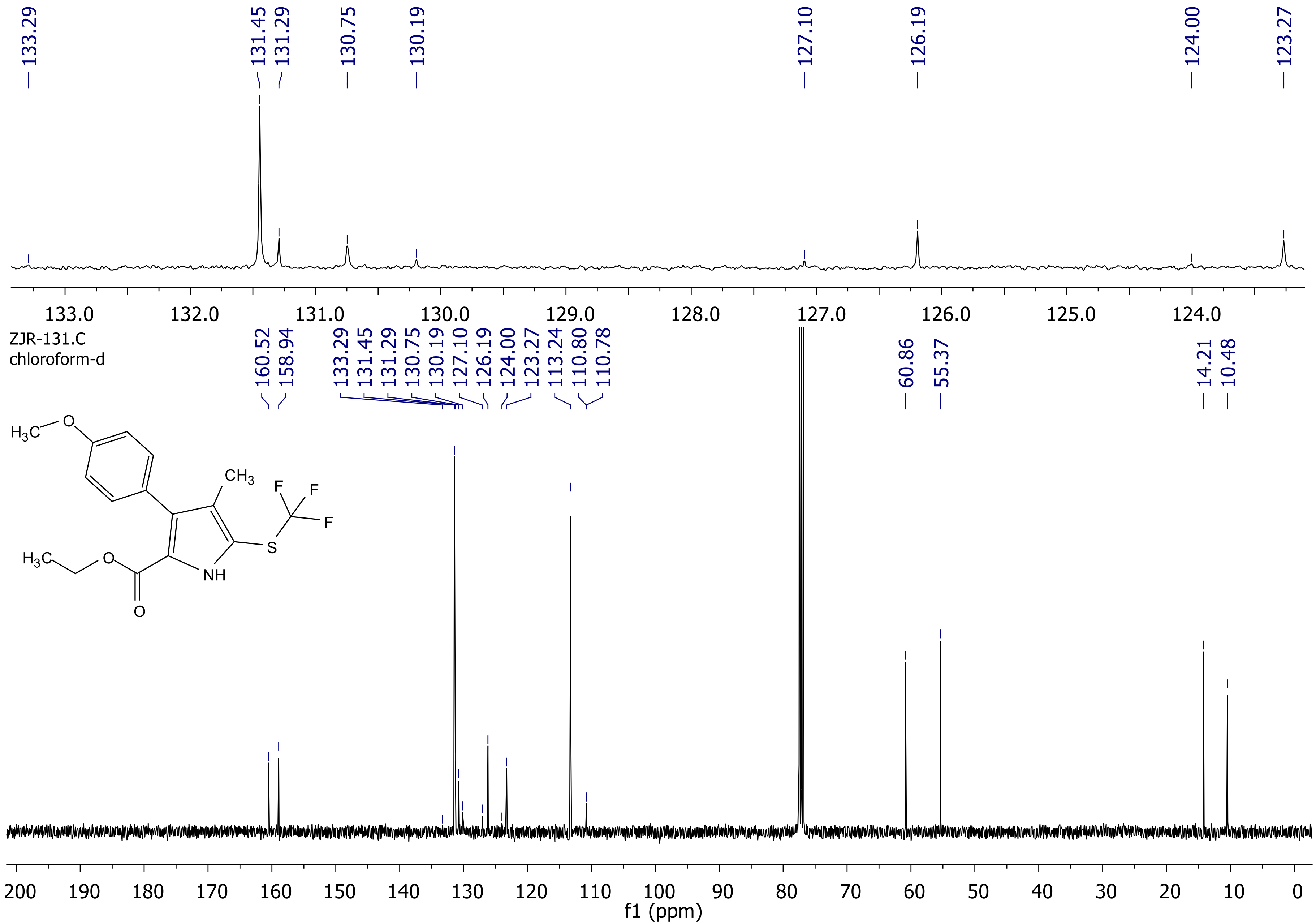

^{19}F NMR spectrum of ethyl 3-(4-methoxyphenyl)-4-methyl-5-((trifluoromethyl)thio)-1H-pyrrole-2-carboxylate (**4o**) in $CDCl_3$ at 100 MHz

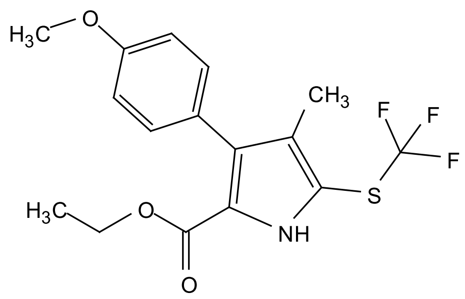

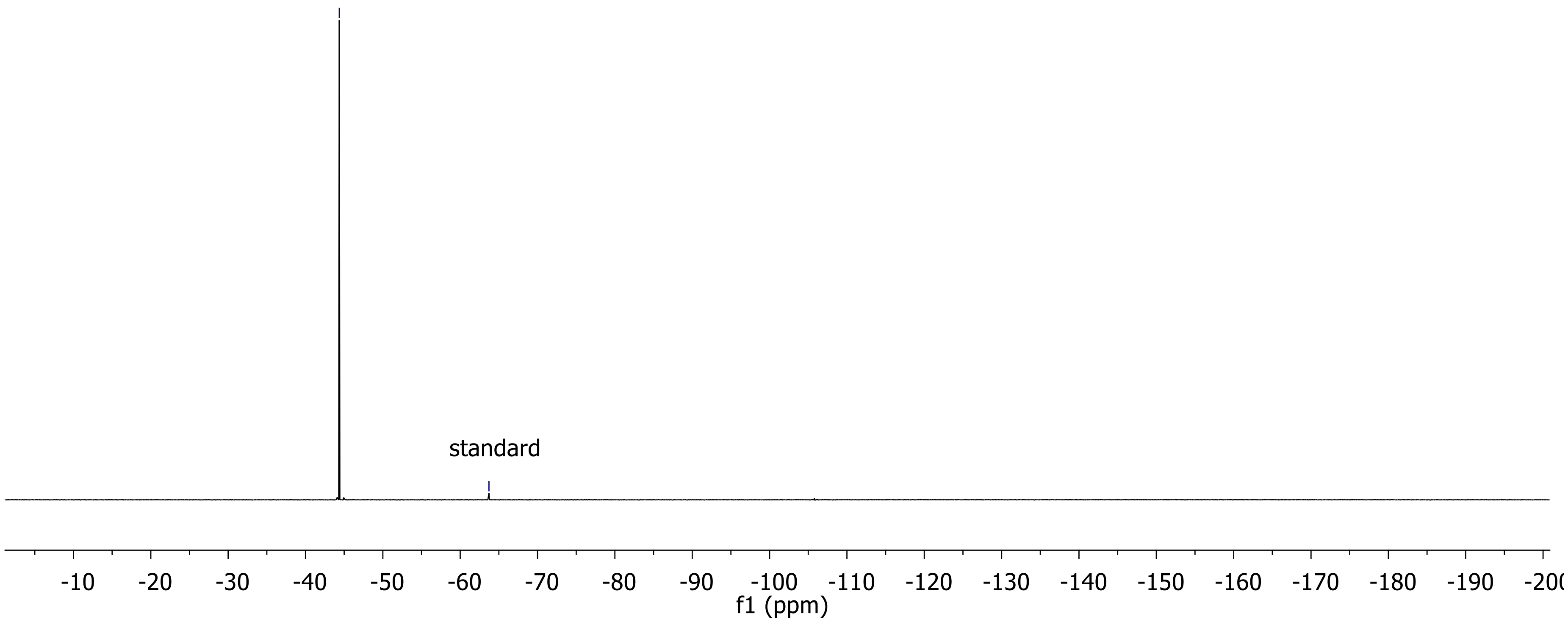

^{19}F NMR spectrum of ethyl 3-(4-methoxyphenyl)-4-methyl-5-((trifluoromethyl)thio)-1H-pyrrole-2-carboxylate (**4o**) in $CDCl_3$ at 376 MHz

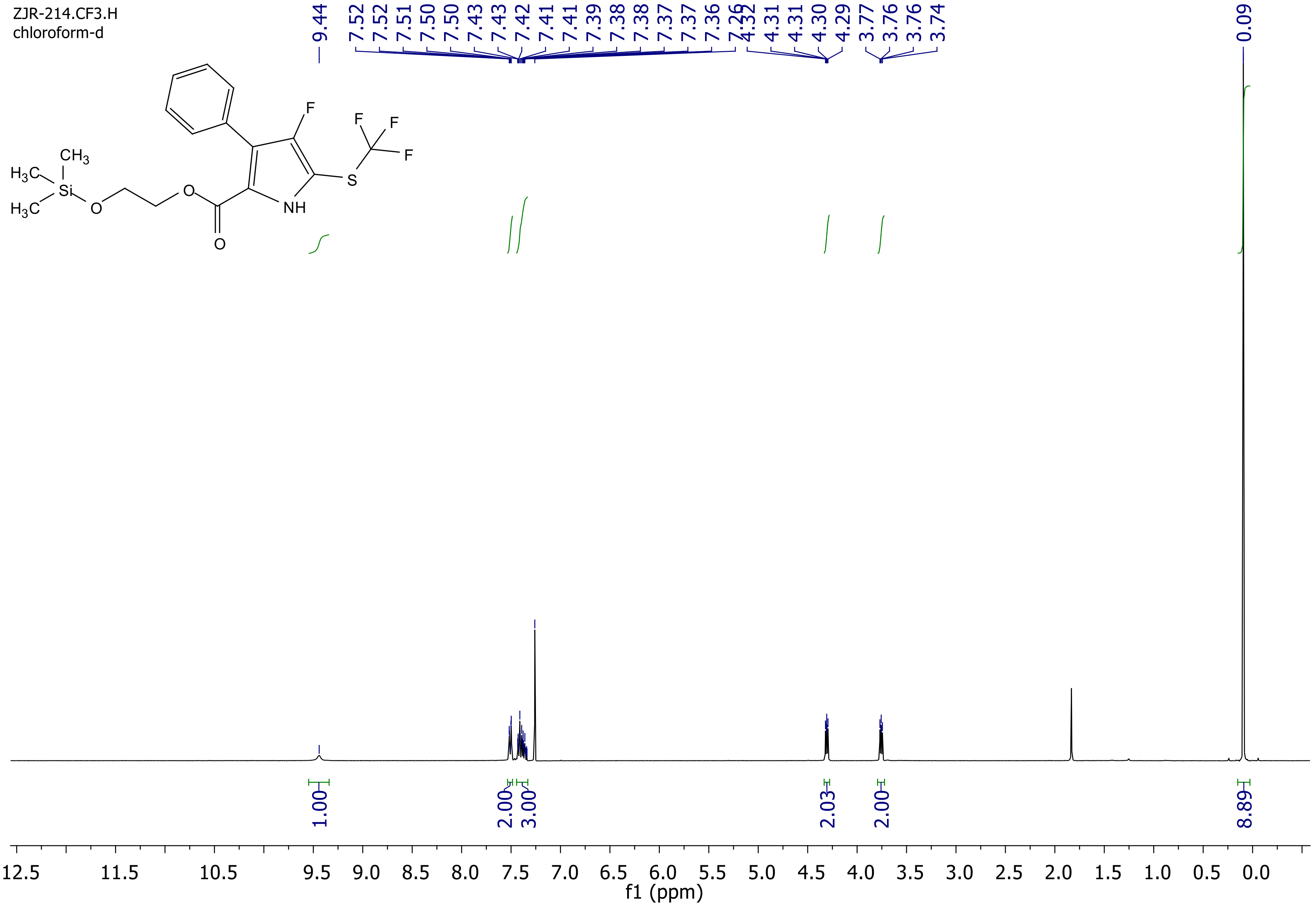

^{1}H NMR spectrum of 2-((trimethylsilyl)oxy)ethyl 4-fluoro-3-phenyl-5-((trifluoromethyl)thio)-1H-pyrrole-2-carboxylate (**4p**) in $CDCl_3$ at 400 MHz

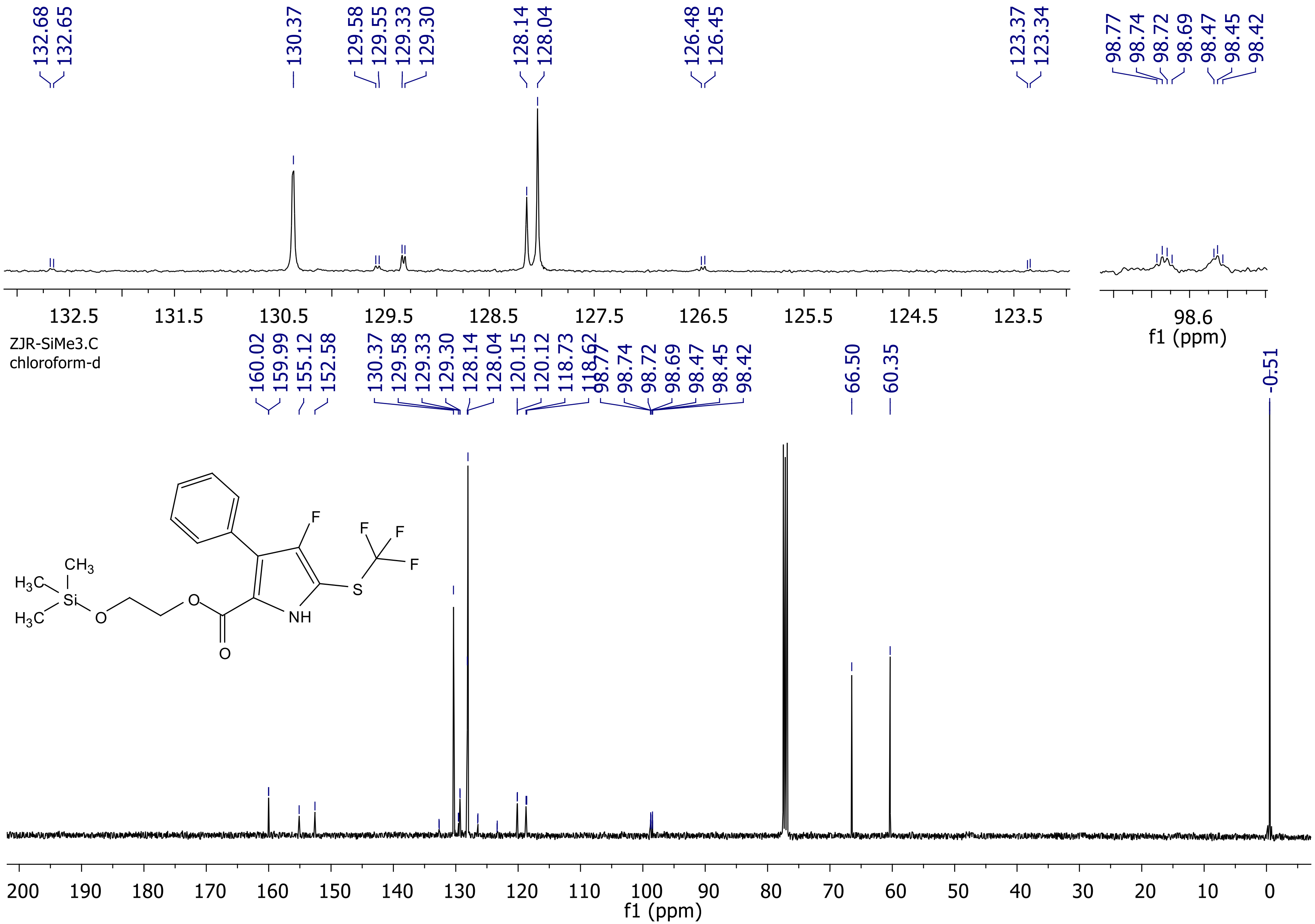

^{13}C NMR spectrum of 2-((trimethylsilyl)oxy)ethyl 4-fluoro-3-phenyl-5-((trifluoromethyl)thio)-1H-pyrrole-2-carboxylate (**4p**) in $CDCl_3$ at 100 MHz

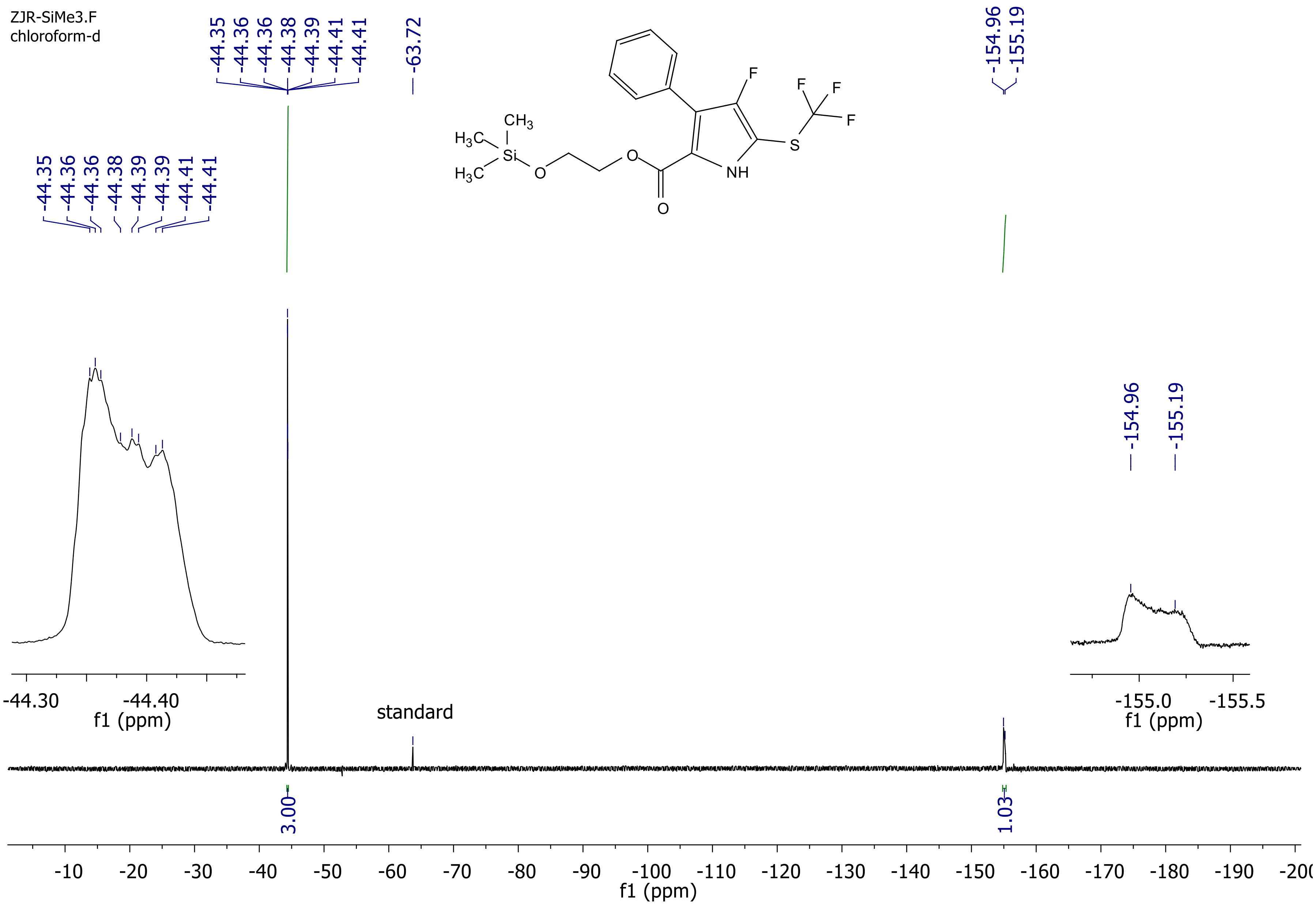

^{19}F NMR spectrum of 2-((trimethylsilyl)oxy)ethyl 4-fluoro-3-phenyl-5-((trifluoromethyl)thio)-1H-pyrrole-2-carboxylate (**4p**) in $CDCl_3$ at 376 MHz

ZJR-190.2.H
chloroform-d

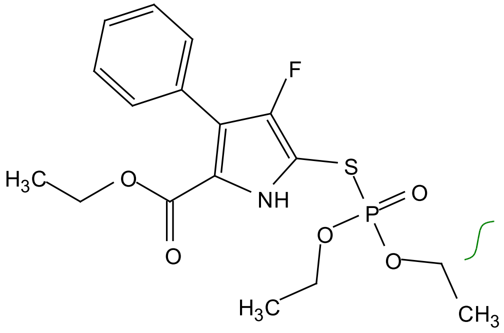

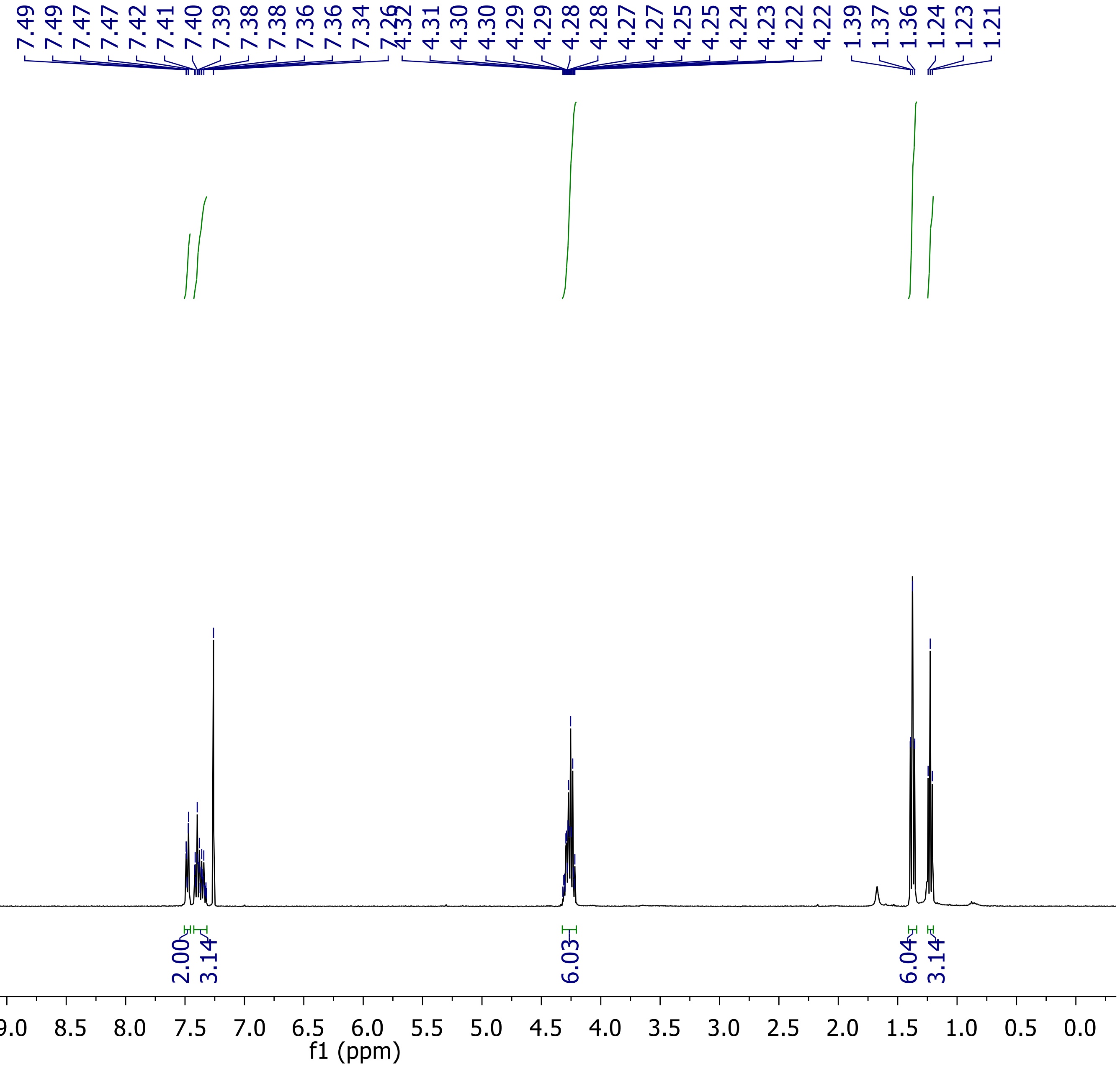

^{1}H NMR spectrum of ethyl 5-((diethoxyphosphoryl)thio)-4-fluoro-3-phenyl-1H-pyrrole-2-carboxylate (**5**) in $CDCl_3$ at 400 MHz

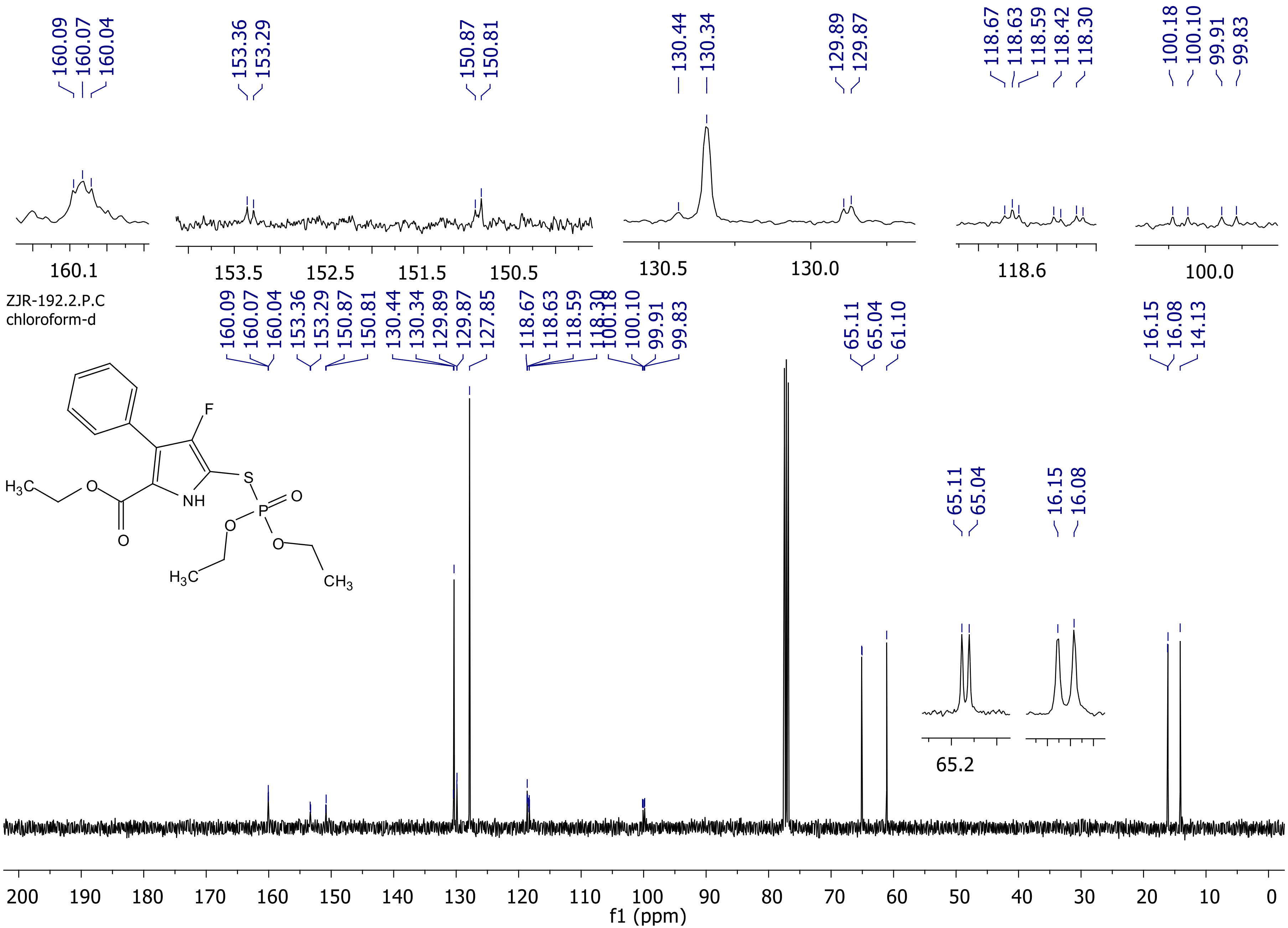

^{13}C NMR spectrum of ethyl 5-((diethoxyphosphoryl)thio)-4-fluoro-3-phenyl-1H-pyrrole-2-carboxylate (**5**) in $CDCl_3$ at 100 MHz

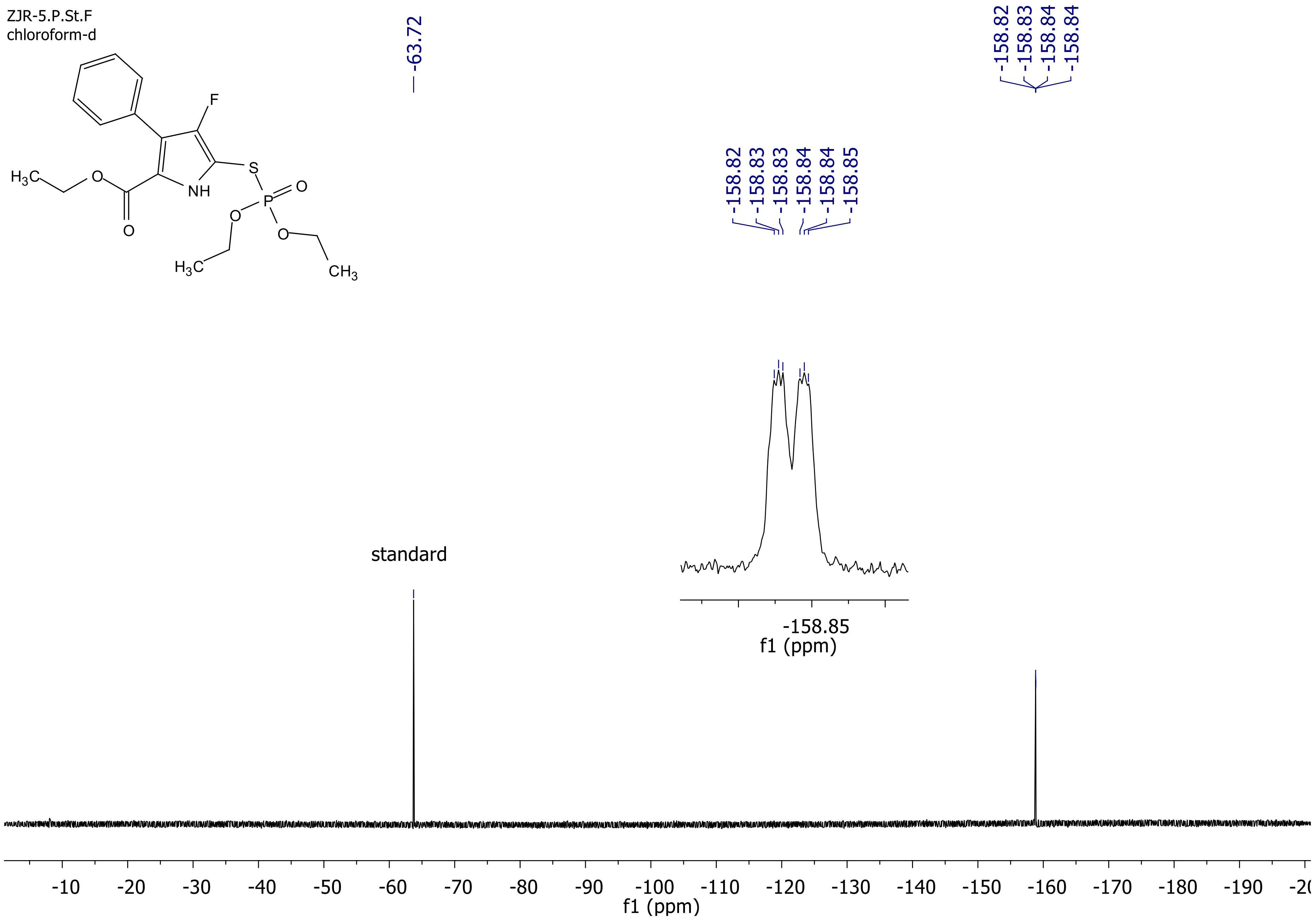

^{19}F NMR spectrum of ethyl 5-((diethoxyphosphoryl)thio)-4-fluoro-3-phenyl-1H-pyrrole-2-carboxylate (**5**) in $CDCl_3$ at 376 MHz

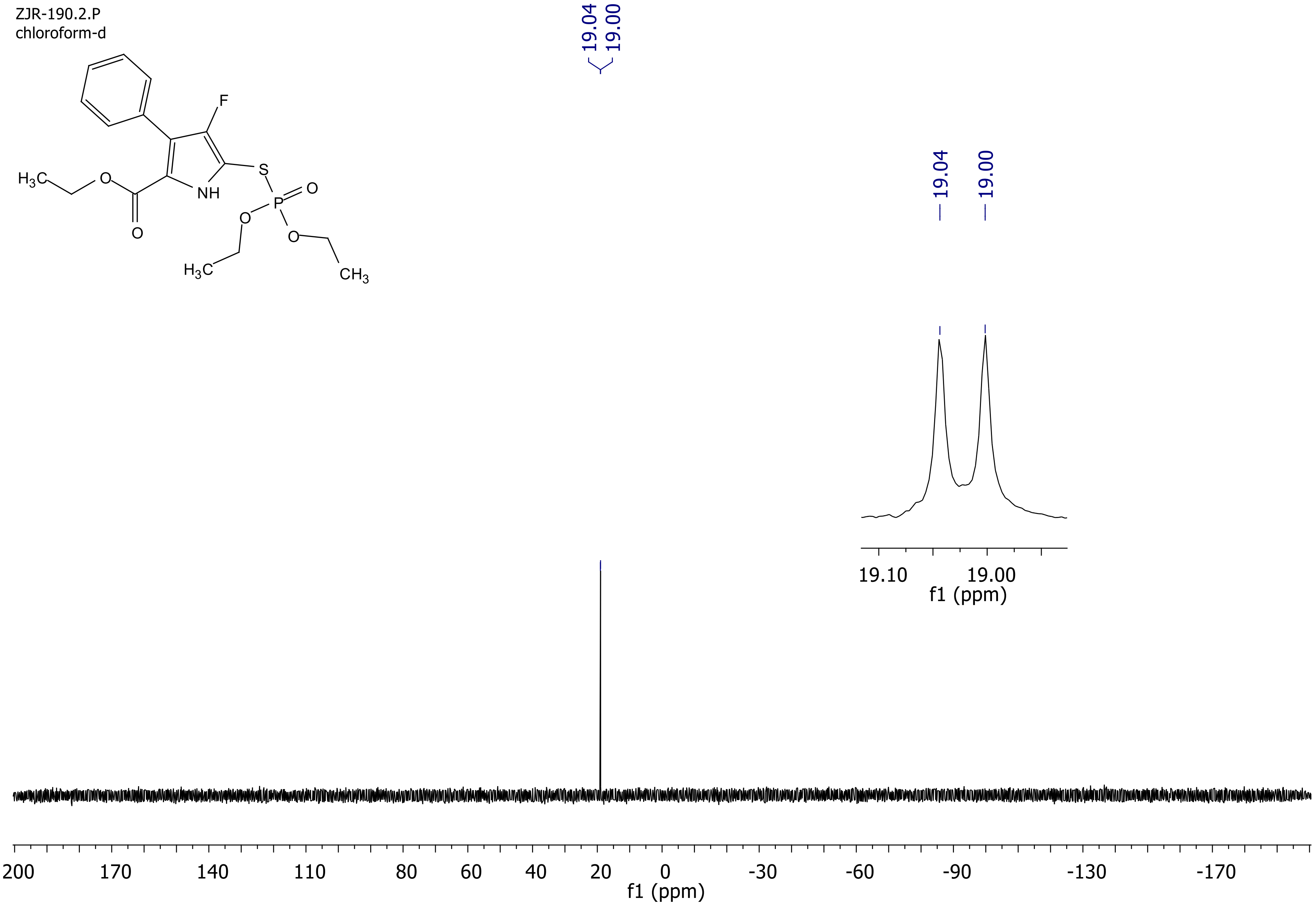

^{31}P NMR spectrum of ethyl 5-((diethoxyphosphoryl)thio)-4-fluoro-3-phenyl-1H-pyrrole-2-carboxylate (**5**) in $CDCl_3$ at 162 MHz

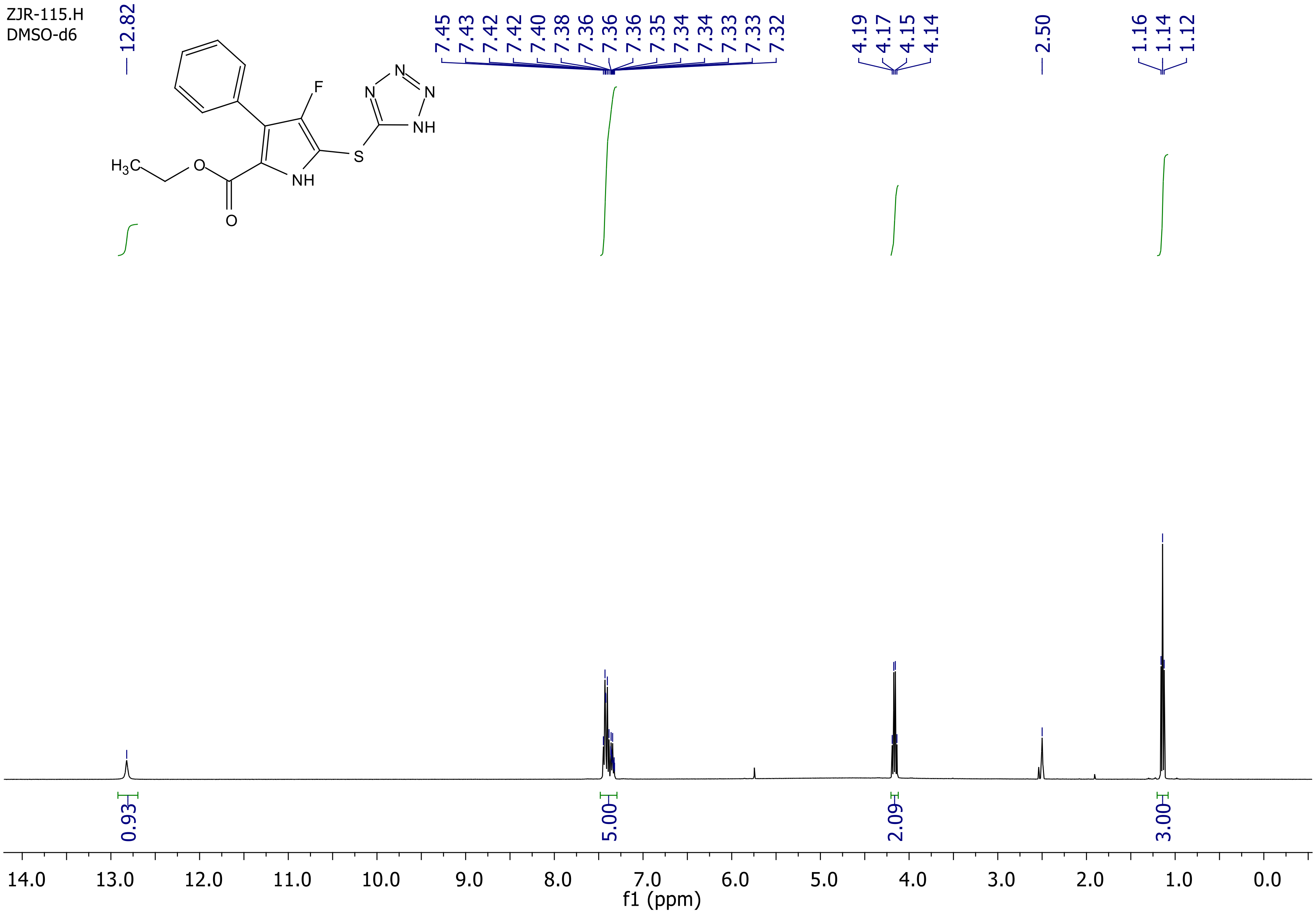

^{1}H NMR spectrum of ethyl 5-((1H-tetrazol-5-yl)thio)-4-fluoro-3-phenyl-1H-pyrrole-2-carboxylate (**6**) in DMSO-d6 at 400 MHz

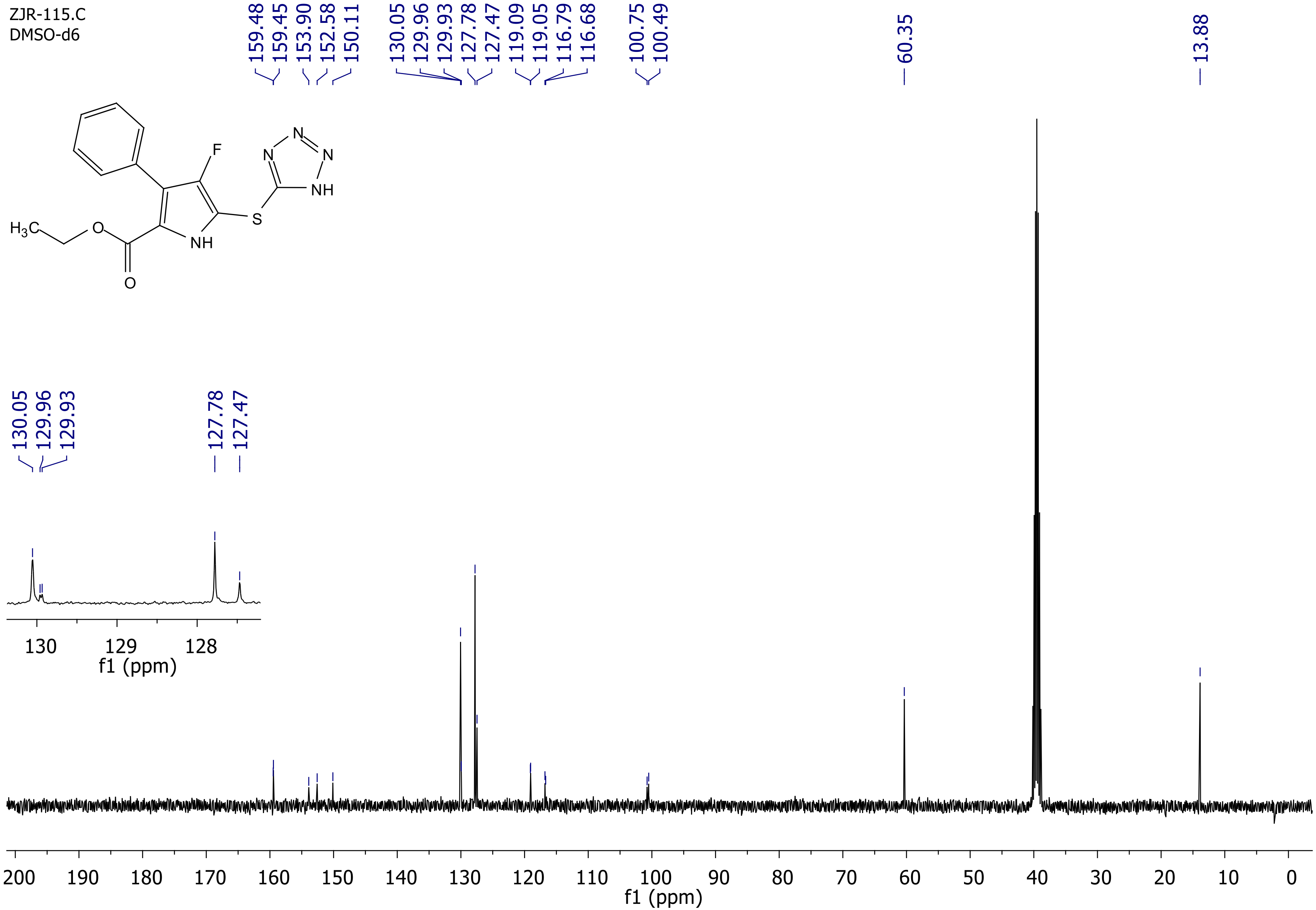

^{13}C NMR spectrum of ethyl 5-((1H-tetrazol-5-yl)thio)-4-fluoro-3-phenyl-1H-pyrrole-2-carboxylate (**6**) in DMSO-d6 at 100 MHz

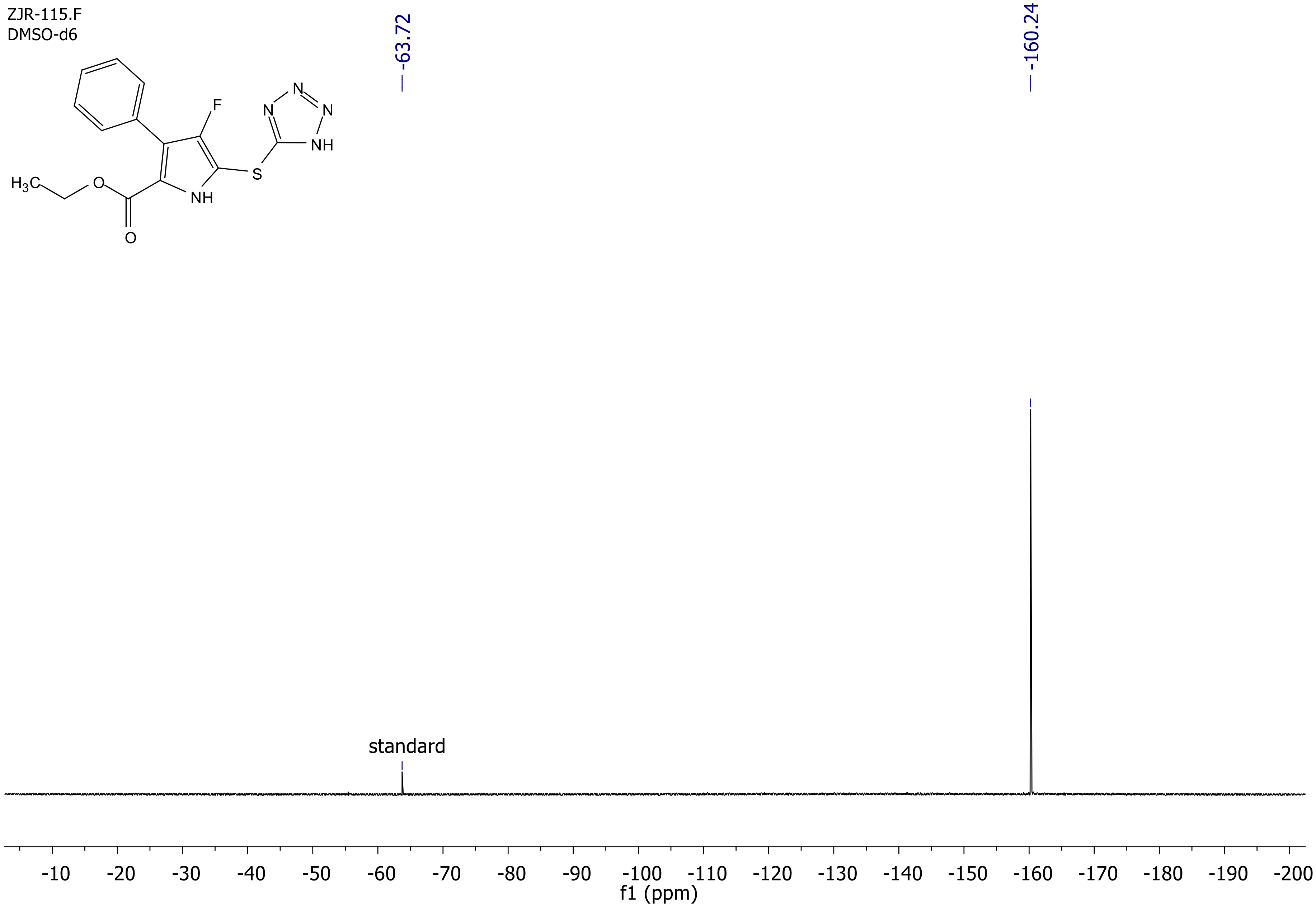

^{19}F NMR spectrum of ethyl 5-((1H-tetrazol-5-yl)thio)-4-fluoro-3-phenyl-1H-pyrrole-2-carboxylate (**6**) in DMSO-d6 at 376 MHz

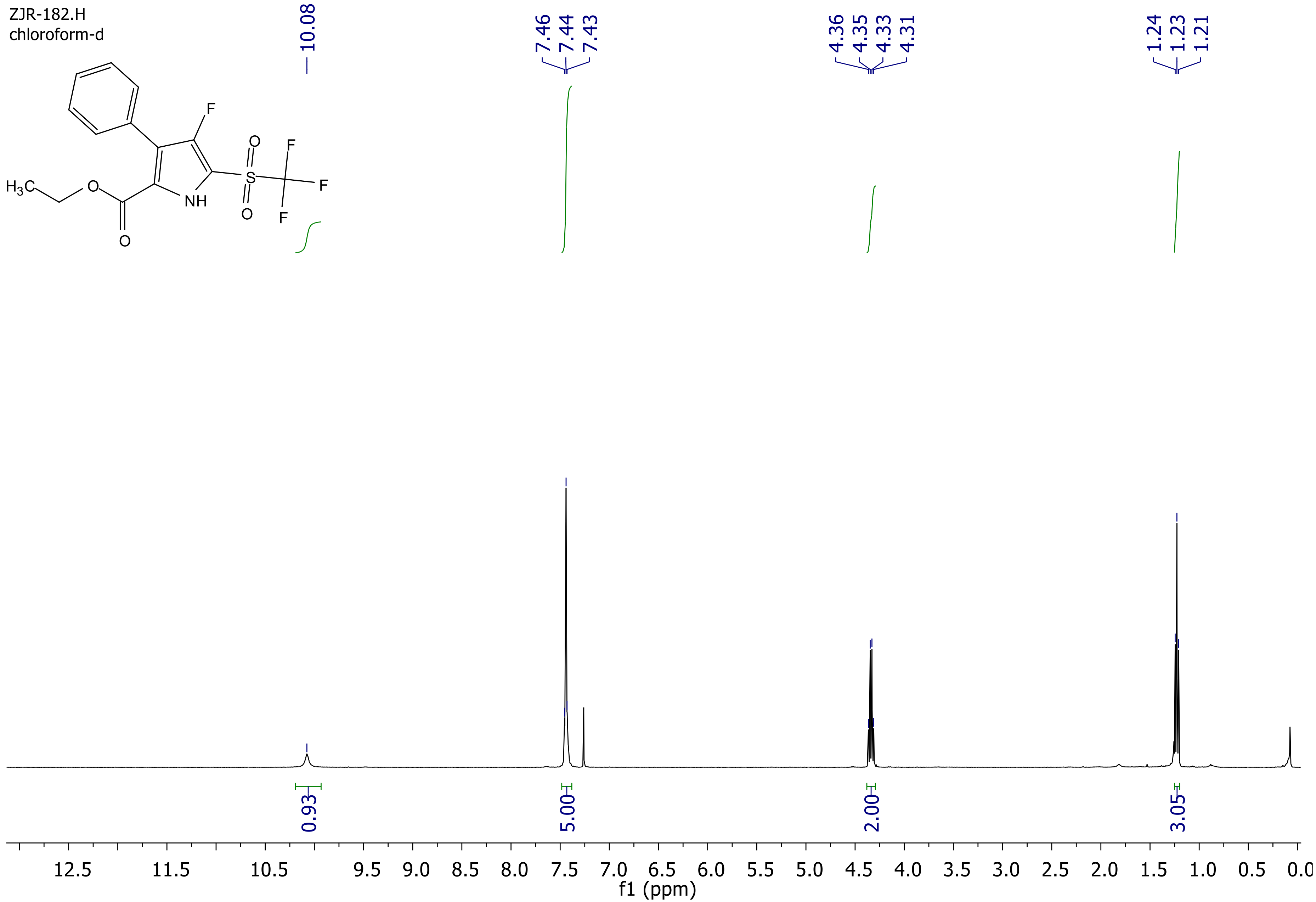

^{1}H NMR spectrum of ethyl 4-fluoro-3-phenyl-5-((trifluoromethyl)sulfonyl)-1H-pyrrole-2-carboxylate (**7**) in $CDCl_3$ at 400 MHz

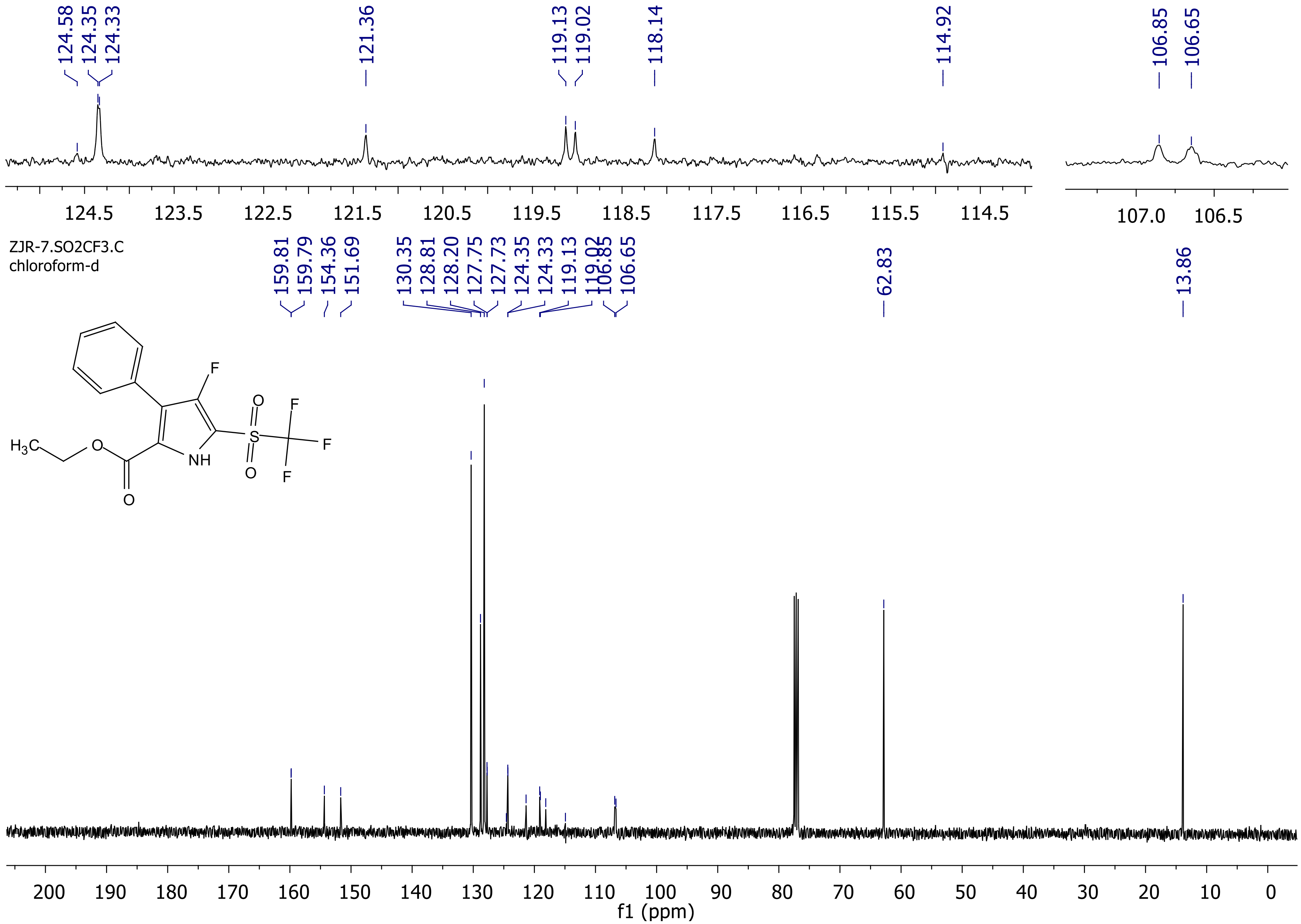

^{13}C NMR spectrum of ethyl 4-fluoro-3-phenyl-5-((trifluoromethyl)sulfonyl)-1H-pyrrole-2-carboxylate (**7**) in $CDCl_3$ at 100 MHz

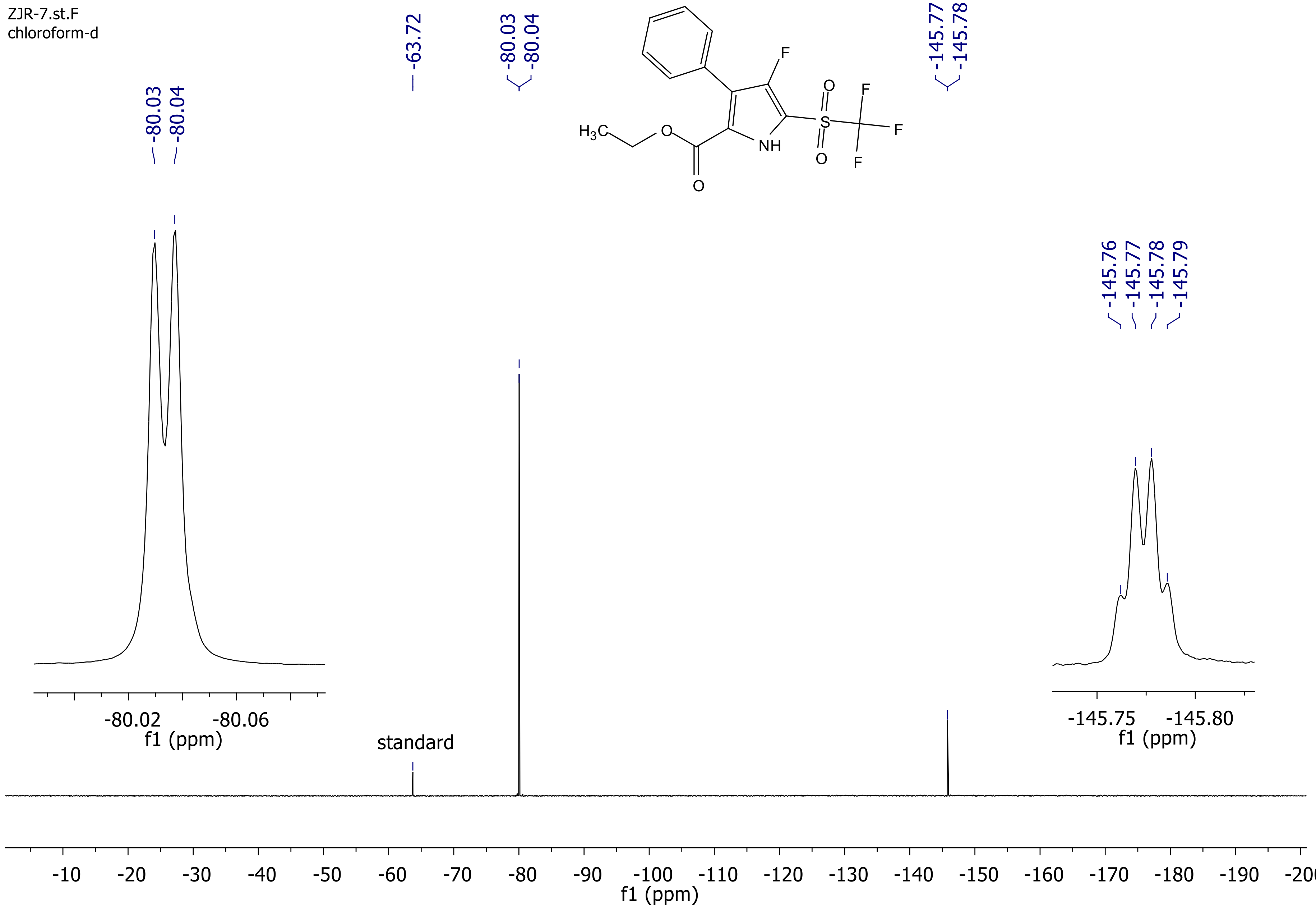

^{19}F NMR spectrum of ethyl 4-fluoro-3-phenyl-5-((trifluoromethyl)sulfonyl)-1H-pyrrole-2-carboxylate (**7**) in $CDCl_3$ at 376 MHz

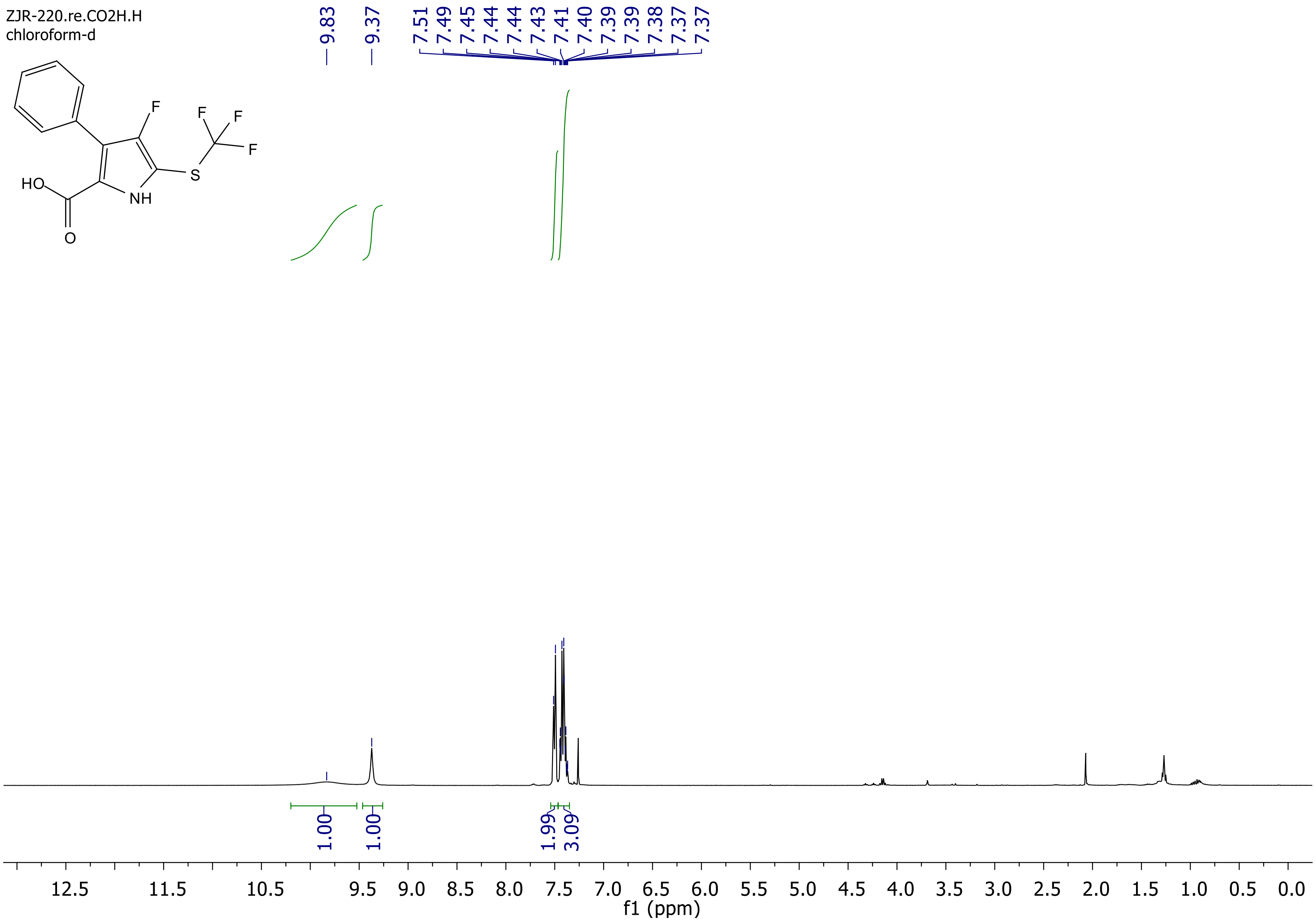

^1H NMR spectrum of 4-fluoro-3-phenyl-5-((trifluoromethyl)thio)-1H-pyrrole-2-carboxylic acid (**8**) in $CDCl_3$ at 400 MHz

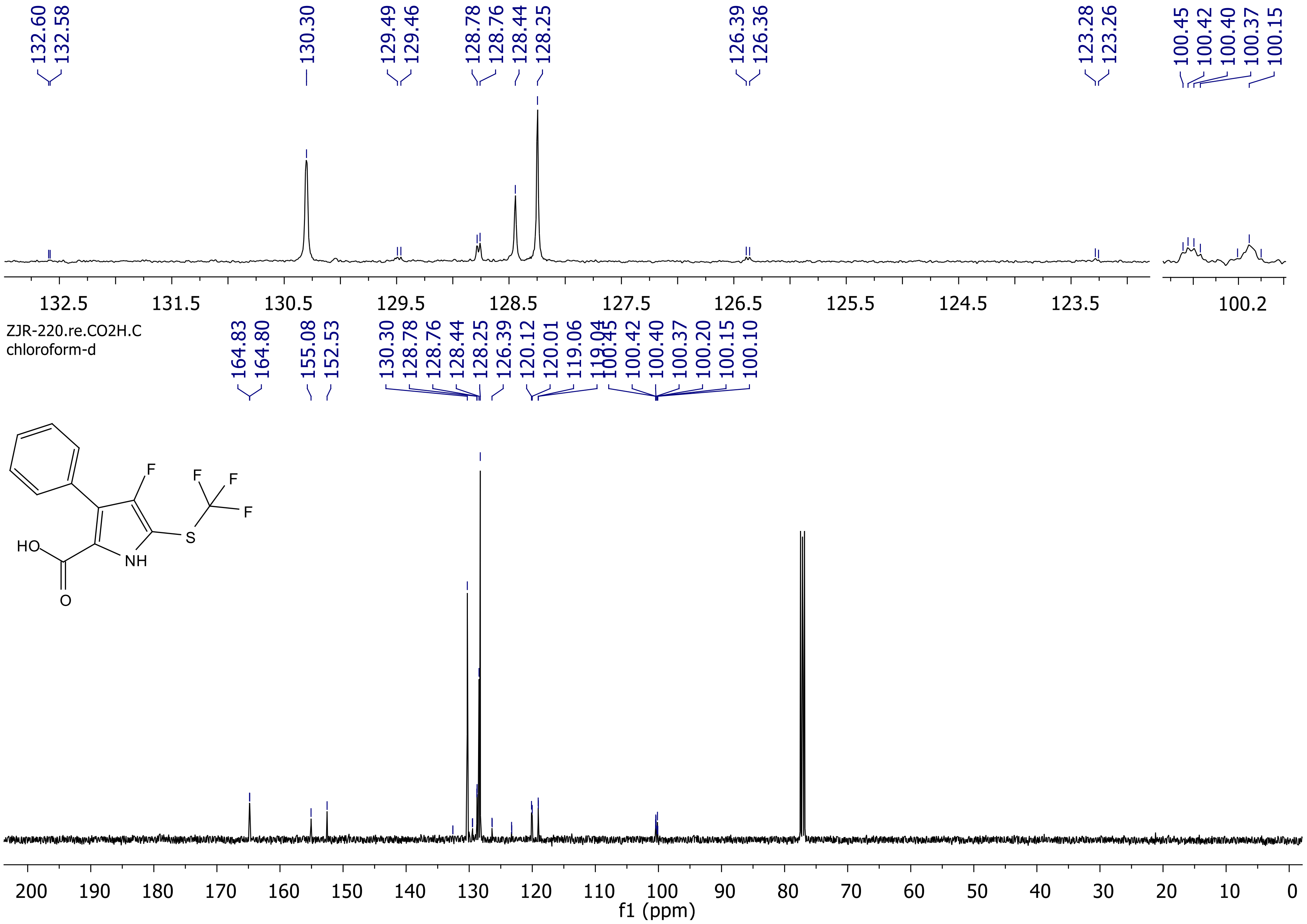

^{13}C NMR spectrum of 4-fluoro-3-phenyl-5-((trifluoromethyl)thio)-1H-pyrrole-2-carboxylic acid (**8**) in $CDCl_3$ at 100 MHz

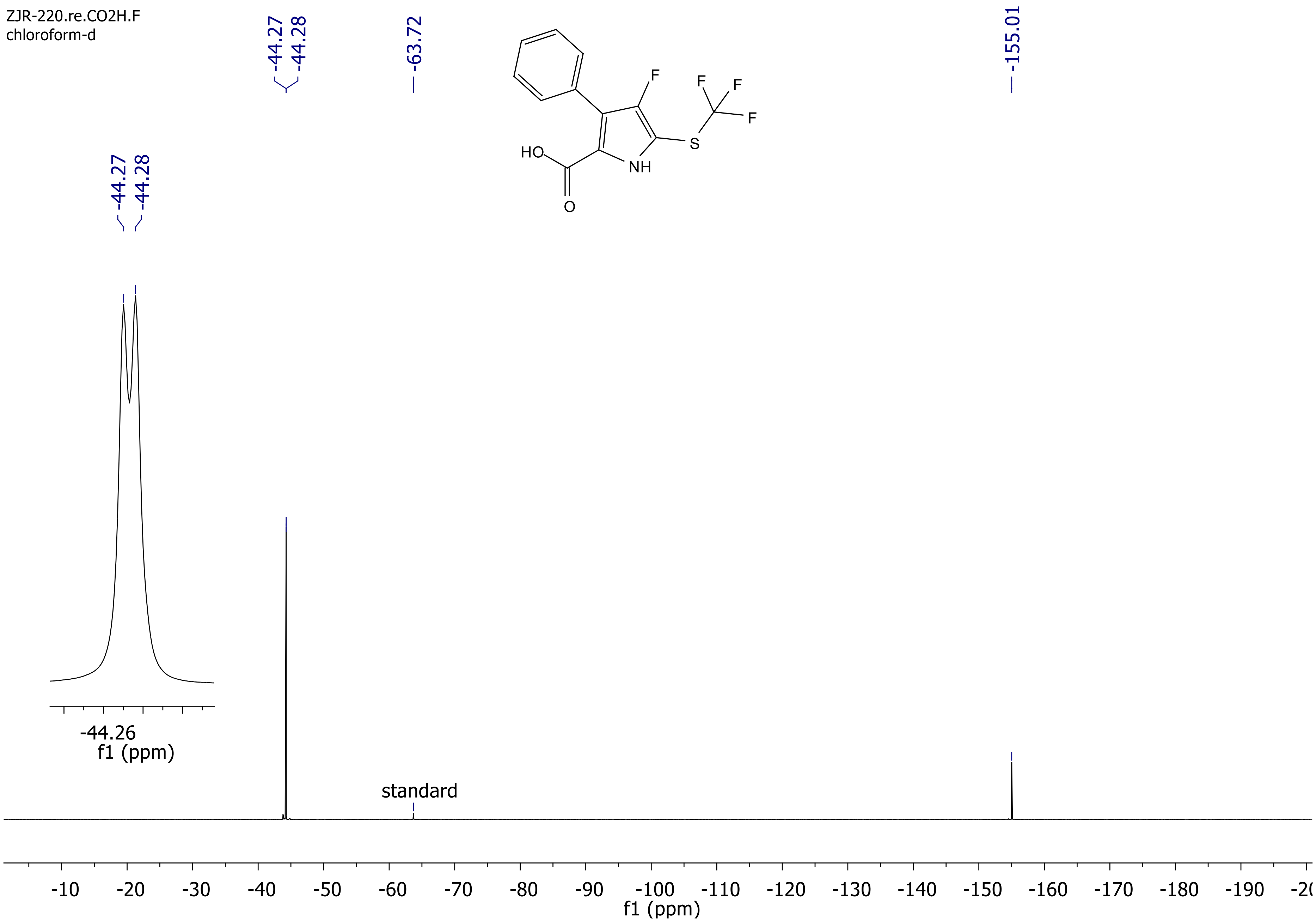

^{19}F NMR spectrum of 4-fluoro-3-phenyl-5-((trifluoromethyl)thio)-1H-pyrrole-2-carboxylic acid (**8**) in $CDCl_3$ at 376 MHz
